# Single-cell Raman-activated sorting and cultivation (scRACS-Culture) for assessing and mining in situ phosphate-solubilizing microbes from nature

**DOI:** 10.1038/s43705-022-00188-3

**Published:** 2022-10-30

**Authors:** Xiaoyan Jing, Yanhai Gong, Huihui Pan, Yu Meng, Yishang Ren, Zhidian Diao, Runzhi Mu, Teng Xu, Jia Zhang, Yuetong Ji, Yuandong Li, Chen Wang, Lingyun Qu, Li Cui, Bo Ma, Jian Xu

**Affiliations:** 1 Single-Cell Center, CAS Key Laboratory of Biofuels, Shandong Key Laboratory of Energy Genetics, Qingdao Institute of BioEnergy and Bioprocess Technology, Chinese Academy of Sciences, Qingdao, Shandong China; 2 University of Chinese Academy of Sciences, Beijing, China; 3 Shandong Energy Institute, Qingdao, Shandong China; 4 Qingdao New Energy Shandong Laboratory, Qingdao, Shandong China; 5 Qingdao Zhang Cun River Water Co., Ltd, Qingdao, Shandong China; 6 Qingdao Single-Cell Biotechnology Co., Ltd, Qingdao, Shandong China; 7 The First Institute of Oceanography, Ministry of Natural Resources, Qingdao, Shandong China; 8 Key Laboratory of Urban Environment and Health, Institute of Urban Environment, Chinese Academy of Sciences, Xiamen, Fujian, China

**Keywords:** Molecular biology, Environmental sciences

## Abstract

Due to the challenges in detecting in situ activity and cultivating the not-yet-cultured, functional assessment and mining of living microbes from nature has typically followed a ‘culture-first’ paradigm. Here, employing phosphate-solubilizing microbes (PSM) as model, we introduce a ‘screen-first’ strategy that is underpinned by a precisely one-cell-resolution, complete workflow of single-cell Raman-activated Sorting and Cultivation (scRACS-Culture). Directly from domestic sewage, individual cells were screened for in-situ organic-phosphate-solubilizing activity via D_2_O intake rate, sorted by the function via Raman-activated Gravity-driven Encapsulation (RAGE), and then cultivated from precisely one cell. By scRACS-Culture, pure cultures of strong organic PSM including *Comamonas* spp., *Acinetobacter* spp., *Enterobacter* spp. and *Citrobacter* spp., were derived, whose phosphate-solubilizing activities in situ are 90–200% higher than in pure culture, underscoring the importance of ‘screen-first’ strategy. Moreover, employing scRACS-Seq for post-RACS cells that remain uncultured, we discovered a previously unknown, low-abundance, strong organic-PSM of *Cutibacterium* spp. that employs secretary metallophosphoesterase (MPP), cell-wall-anchored 5′-nucleotidase (encoded by *ush*A) and periplasmic-membrane located PstSCAB-PhoU transporter system for efficient solubilization and scavenging of extracellular phosphate in sewage. Therefore, scRACS-Culture and scRACS-Seq provide an in situ function-based, ‘screen-first’ approach for assessing and mining microbes directly from the environment.

## Introduction

Function-based mining of microbes from nature has traditionally employed a ‘culture-first, screen-second’ strategy: cells are exposed to a particular culture condition until individual colonies (each consisting of 10^8-9^ cells) are formed, and then metabolic phenome or genome is screened via a pure culture, i.e., at the resolution of an isogenic population [1]. This results in several limitations. (i) Most cells in nature are yet to be cultured, thus such ‘culture-first’ strategy would have excluded those functional but not-yet-cultured cells before even the screening starts [2, 3]. (ii) Even for those cells that can be cultured, their activity as assessed in pure culture (i.e., in-tube) is frequently distinct or even irrelevant from their in-situ function, thus in-tube-activity-based assessment or screening can be misleading [4, 5]. (iii) Targeting all cells for cultivation from the very start, regardless of their metabolic functions, would result in inefficient investment of bulk of consumables on growing non-target cells.

**Fig. 1:**
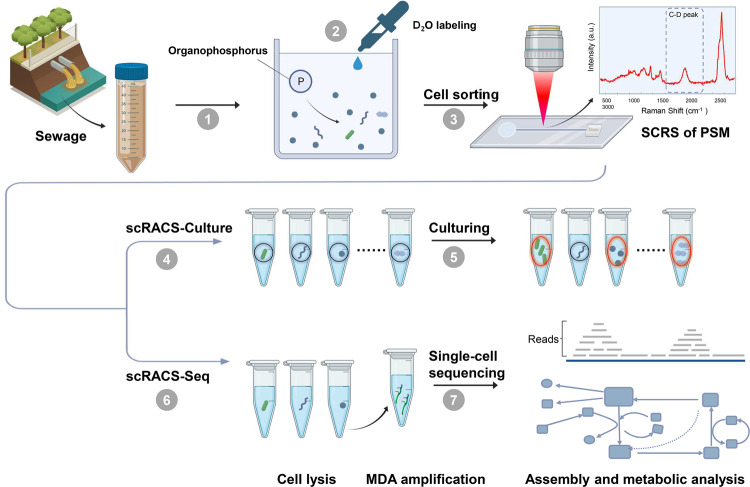
Workflow of scRACS-Seq/Culture for revealing organic-PSM in the sewage that links genotype to phenotype (metabolic activity). ① Cells in the sewage were extracted by Nycodenz density gradient separation (NDGS). ② For sorting organic-PSM, the sewage extract was incubated under the conditions of adding lecithin as organic phosphorus in dephosphorization sewage in 50%-D_2_O for 24 h. ③ Organic-PSM were identified based on the preset C-D ratio threshold (metabolically active) in SCRS, and then sorted out in the RAGE chip, as a one-cell-encapsulated droplet, in an one-cell-one-tube manner. ④ For scRACS-Culture, pL-volume culture was employed for seven days, following by μL volumes for another seven days in broth medium. ⑤ Single-cell-derived organic-PSM cultures were obtained for further validation. ⑥ For scRAGE-Seq, the RAGE-sorted cells were lysed, and then the genomic DNA was amplified by MDA and then processed for 16S rRNA sequencing and whole-genome shotgun sequencing. ⑦ Assembly and annotation of the single-cell shotgun sequencing reads, and then the metabolic pathways were reconstructed to establish the link between genotype (sequencing based) and the metabolic phenotype (SCRS based) at precisely one-cell resolution.

To address these limitations, we envision a ‘screen-first, culture-second’ strategy instead, called single-cell Raman-activated Cell Sorting and Culturing (scRACS-Culture), where, at resolution of precisely one cell and directly from the environmental sample, cells of targeted function are screened via the in situ metabolic phenome based on single-cell Raman spectrum (SCRS) and then cultured (Fig. 1). (*i*) At the SCRS-based functional-cell detection step, a SCRS which is an intrinsic biochemical fingerprint of a cell can identify or classify the cell based on a wide range of metabolic functions [6–8] or via its taxonomy (e.g., for microalgae [9] or for bacteria [10]); in particular, the shifts of specific Raman bands in SCRS can quantitatively measure its substrate-intake activity, when substrates with stable isotopes (e.g. ^13^C, ^15^N, ^2^H, ^18^O) are assimilated by the cell [6, 8]. (*ii*) At the RACS step, live cells of targeted metabolic function as recognized by SCRS can be obtained by various RACS technologies [6], either in an indexed, one-cell-per-tube manner [11] or via high-throughput systems such as FlowRACS [12]. (*iii*) At the sorted-cell cultivation step, pure cultures is derived from either the pool of sorted cells or each cell individually.

As theoretically every cell in a natural microbiota is distinct in its genotypes and phenotypes, sorting and culturing at precisely one-cell resolution is a necessity. However, scRACS-Culture has not been reported for environmental or human microbiota. The challenges here are rooted in the manipulation of individual live bacterial cells of very small size, specifically: (*i*) the complexity of environmental samples where many unknown factors can hinder efficient treatment and collection of cells at their proper metabolic state prior to the SCRS acquisition, (*ii*) the detrimental effects on cell vitality by laser irradiation during SCRS acquisition, particularly for non-resonance-Raman-peak based screening which demands longer laser exposure [13], and (*iii*) the enormous diversity in genome and exceedingly complexity in metabolic phenome for all the cells in a microbiota.

Eutrophication is an ecological disaster where an entire or partial body of water becomes enriched with minerals and nutrients, such as the phosphorus (P) which is present in inorganic or organic molecules and can be either soluble or insoluble [14]. To prevent or tackle eutrophication, sewage treatment that reduces inorganic and organic P is necessary, usually via a microbe-mediated P-removal process (as it produces minimal sludge and avoids secondary pollution [15]). As soluble P can be rapidly utilized by nearly all life forms, key to the sewage treatment process are the inorganic-P-solubilizing microbes (inorganic-PSM) or organic-P-solubilizing microbes (organic-PSM), which dissolve insoluble P to maintain vitality when soluble P is unavailable [15, 16]. Unfortunately, most microbes can utilize only soluble P, thus efficient PSM strains are highly sought-after resources, for reducing pollution in water bodies and soil, promoting nutrient absorption of crops, improving fertilizer efficiency, and reducing the use of chemical fertilizers [17]. Traditionally, PSM are isolated via a ‘culture-first’ strategy, by plating environmental samples on the Pikovskaya’s or yolk media that contain insoluble inorganic- or organic-P as the only P source [18–22]. However, the P-solubilizing activity of artificial-medium-cultured PSM is not necessarily the in situ activity [23, 24]. As a result, it is usually not clear whether those pure-cultured PSM that exhibit high activity in artificial media are the highly active PSM in the natural environments, and vice versa [5]. Therefore, identifying the in-situ functioning PSM, unraveling the underlying P-solubilizing mechanisms, and obtaining pure cultures of those PSM with high P-solubilizing activity in-situ have been of keen interest.

Here, employing the mining of organic-PSM that function in situ as a model, we establish a single-cell-resolution, complete workflow of scRACS-Culture for environmental microbiomes. Directly from domestic sewage, organic PSM are detected and quantitatively assessed based on in-situ activity via its SCRS, sorted accordingly by Raman-activated Gravity-driven Encapsulation (RAGE), and then pure cultures derived via precisely one-cell pL-microdroplets. Moreover, one-cell RACS-Seq revealed a previously unknown organic-PSM of *Cutibacterium* spp. that employs secretary metallophosphoesterase (MPP, metallo-dependent phosphatase) and 5’-nucleotidase (*ush*A) for efficient phosphate dissolution. Notably, our scRACS-Culture derived organic-PSM such as *Comamonas* spp., *Acinetobacter* spp., *Enterobacter* spp. and *Citrobacter* spp. all exhibit much higher CDR in situ than in pure cultures, potentially suggesting these bacteria are more active in P-solubilization in situ than in pure cultures. Thus scRACS-Culture can serve as a valuable and generally applicable approach for in situ-function-based microbial assessment and mining from nature.

## Results

### Benchmarking C-D band in SCRS as biomarker for identifying and characterizing PSM when insoluble P is the only P source

When just insoluble P is present, only PSM (but not non-PSM) are metabolically active and can assimilate the deuterium (D) from D_2_O to synthesize intracellular P-harboring molecules such as adenosine triphosphate (ATP), phospholipids and nucleic acids [25], resulting in the emergence of a C-D band in SCRS [26, 27]. Therefore, intensity of the C-D band can distinguish PSM from non-PSM under insoluble P [27].

To establish the ‘screen-first’ strategy of scRACS-Culture for PSM, we first probed whether the C-D band can serve as quantitative biomarker for the in situ P-solubilizing activity. Six bacterial species with well-characterized P-solubilizing activity were chosen as benchmarks (“Methods”), including three inorganic-PSM (*Bacillus subtilis* H6, *Escherichia coli* BL21 and *Bacillus megaterium* ACCC02970), two inorganic- and organic-PSM (*Aeromonas hydrophila* ATCC7966 and *Burkholderia cepacian* BN337012), and one non-PSM (*Enterococcus faecium* PH07). Metabolic activities of the six bacteria incubated in MM-sP (minimal medium with soluble P), MM-iiP (with the inorganic insoluble substrate of tricalcium phosphate as the only P source), MM-oiP (with the organic insoluble substrate of lecithin as the only P source) or MM-P_free (without P) were probed via the SCRS, respectively.

**Fig. 2:**
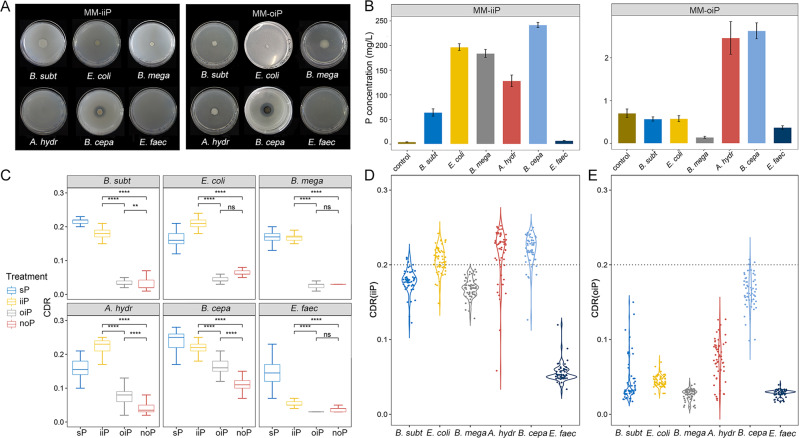
Determination of phosphorus-releasing capacity of pure-cultured bacteria that include *B. subtilis* H6, *E. coli* BL21, *B. megaterium* ACCC02970, *A. hydrophila* ATCC7966, *B. cepacian* BN337012, and *E. faecium* PH07. **A** Halo zones produced by these strains on the solid medium of MM-iiP and MM-oiP, respectively; **B** Concentrations of soluble P in supernatant via the molybdenum-antimony resistance colorimetric method in the liquid medium of MM-iiP and MM-oiP, respectively. Error bars represent the standard deviation of three measurements from biological triplicates; **C** C-D ratios of the six bacteria strains incubated in minimum media containing 50%-D_2_O without P (P_free), with dissolvable P (sP), Ca_3_(PO_4_)_2_ (iiP) and lecithin (oiP), respectively. CDR distribution patterns of the six species are compared between MM-iiP (**D**) and MM-oiP (**E**). CDR = 0.20 was chosen as the threshold. Sixty individual cells from each bacteria sample were measured by Raman spectroscopy.

As a control, in a conventional, ‘culture-first’ approach, the P-solubilizing activities were measured based on the size of halo zones around bacterial colony on solid medium containing either organic or inorganic P (“Methods”). For the PSM activities towards inorganic insoluble P (i.e., on the MM-iiP plates), *B. subtilis* H6, *E. coli* BL21, *B. megaterium* ACCC02970, *A. hydrophila* ATCC7966 and *B. cepacian* BN337012 all produce the halo zones, consistent with their known talents as inorganic PSM (Fig. 2A). Similarly, for the PSM of organic insoluble P (i.e., on the MM-oiP plates), only *A. hydrophila* ATCC7966 and *B. cepacian* BN337012 show such halo zones on the MM-oiP plates, supporting the two bacteria as organic-PSM (Fig. 2A).

As a second control which is also ‘culture-first’, the P-solubilizing ability was profiled by culturing each of the strains in liquid medium with tricalcium phosphate (i.e., inorganic P) or lecithin (i.e., organic P) as the insoluble P source and then quantifying microbe-released soluble P in medium (after shaking at 180 r/min and 30 °C, in triplicates; “Methods”). In the inorganic P group, the medium soluble P content of *B. subtilis* H6, *E. coli* BL21, *B. megaterium* ACCC02970, *A. hydrophila* ATCC7966 and *B. cepacian* BN337012 is 3.07- to 4.02-fold of the control, indicating their inorganic P solvation activity (Fig. 2B). In the organic P group, the medium soluble P content is higher than the control group for only *A. hydrophila* ATCC7966 and *B. cepacian* BN337012, indicating their organic-P mineralizing activity (Fig. 2B). In contrast, the negative control *E. faecium* PH07 shows no solvation activities for either inorganic or organic P, in either solid or the liquid medium (Fig. 2A, B). These results validate the respective inorganic- or organic-P solubilizing activities of these strains.

To test whether the microbial P-solubilizing activity can be quantitatively measured via SCRS, the D assimilating rates of the six bacteria incubated in MM-sP, MM-iiP, MM-oiP, and MM-P_free containing 50% D_2_O were probed respectively. SCRS from 60 individual bacterial cells were acquired for each sampling point, and the C-D ratio (CDR) calculated to measure the metabolic activity of each cell [28, 29]. In contrast to bacteria incubated in MM-P_free, all the six strains in MM-sP exhibit a prominent C-D band in the 2040–2300 cm^−1^ region (Fig. 2C). For *B. subtilis* H6, *E. coli* BL21 and *B. megaterium* ACCC02970, the CDR from MM-iiP (mean of 0.18, 0.21, 0.17 after 24 h incubation; *P* < 0.01 for all three pairs; Wilcoxon test) are much higher than MM-oiP (mean of 0.04, 0.04, 0.02; *P* < 0.01 for all) and MM-P_free (mean of 0.03, 0.06, 0.03; *P* < 0.01 for all) which stay at a low, MM-sP-like level (Fig. 2C). The elevation of CDR suggests metabolic activation of inorganic-PSM only when inorganic P is present. In contrast, for *A. hydrophila* ATCC7966 and *B. cepacian* BN337012, the CDR from MM-iiP (mean of 0.22 for both) and MM-oiP (mean of 0.07 and 0.16) are both higher than MM-P_free (mean of 0.04 and 0.11; *P* < 0.01 for both; Fig. 2C), indicating the two strains are metabolically active under both inorganic and organic P. These SCRS-based results are consistent with the two culture-first approaches (Fig. 2A, B), demonstrating the ability of CDR to not just distinguish but quantitatively assess a cell’s P solvation activity in a microbiome.

Notably, under MM-iiP, the CDR of individual cells from all the tested species spread over a wide range (from 0.03 to 0.25; Fig. 2D). In fact, no numeric thresholds of CDR can unambiguously distinguish the inorganic-PSM from the organic-PSM strains (Fig. 2D). In contrast, with MM-oiP, only a subpopulation of the organic-PSM of *B. cepacian* shows CDR of > 0.20 (5% of all cells; Fig. 2E). Therefore, in scRACS-Culture of sewage for mining PSM, we propose the CDR value of 0.20 as a threshold to screen for high-efficiency organic-PSM, either from pure cultures or directly from environmental microbiota.

### Detecting efficient in situ PSM directly from environmental microbiota via SCRS

**Fig. 3:**
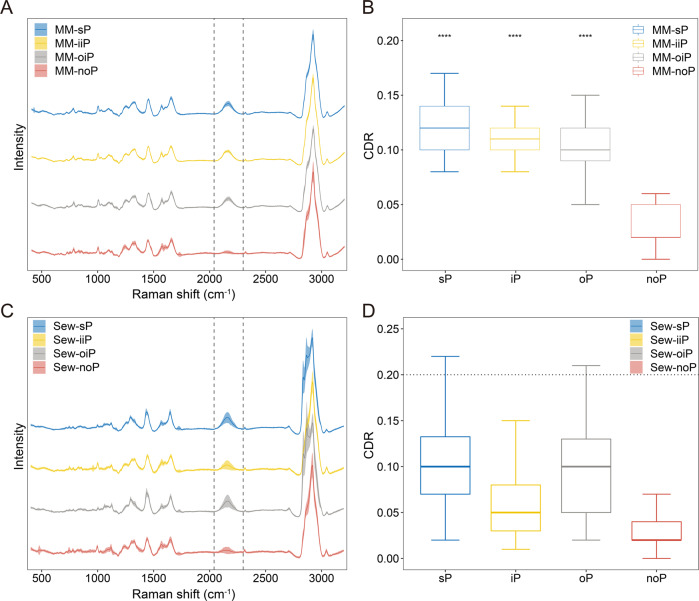
The P-solubilizing activity of indigenous sewage bacteria as quantified by Single-Cell Raman Spectra. SCRS (**A**) and the corresponding C-D ratio (**B**) of sewage bacteria incubated in the artificial culture media of MM-sP, MM-iiP, MM-oiP or MM-P_free with 50% D_2_O for 24 h, respectively. For comparison, also shown are SCRS (**C**) and the corresponding C-D ratio (**D**) of sewage bacteria incubated in dephosphorization sewage with 50%-D_2_O, either with no P (Sew-noP; as negative control) or supplemented with soluble P (Sew-sP), Ca_3_(PO_4_)_2_ (Sew-iiP) or lecithin (Sew-oiP) for 24 h, respectively. Sixty SCRS were acquired (one per cell) per condition, and all data points are presented here.

The in-situ microbial activity in the environment can be distinct from that in pure cultures. To test the ability of scRACS-Culture to assess in situ activity, we collected microbiome samples from the inlet sewage of the Zhangcun River Water Purification Plant, which is after aeration precipitation and grid filtration but prior to entering the anaerobic tank (“Methods”; Fig. S1). We started by inoculating the extracted sewage microbiota into the various minimal media of MM-P_free, MM-sP, MM-iiP or MM-oiP (amended with D_2_O at 50% final conc.), and incubating at room temperature (which simulates the in-situ condition) for 24 h respectively (“Methods”). (*i*) For MM-P_free, no sewage cells show detectable C-D bands in SCRS (Fig. 3A), indicating very low P solubilization activities in the absence of either soluble or insoluble P. (*ii*) For MM-sP, all the 60 microbial cells randomly sampled from each of the samples (in triplicates, with 20 cells for each biological replicate) exhibit high metabolic activity, as evident from their greatly elevated CDR (ranging between 0.08 and 0.21; Fig. 3B). (*iii*) For MM-iiP, the CDR observed are nearly identical to MM-sP, with both being relatively high (ranging between 0.08 and 0.15; Fig. 3B), indicating that inorganic-PSM account for a significant proportion of cells sampled. This is probably because in MM-iiP, inorganic-PSM are enriched in large quantities due to their dominant growth. (*iv*) For MM-oiP, the CDR are highly heterogeneous (ranging from 0.05 to 0.15; Fig. 3B), which suggests the presence of organic-PSM with both weak and strong P-solubilizing activities.

To confirm the presence of inorganic- or organic-PSM as detected by SCRS in these artificial media, the *pqq*C gene and *pho*X gene, which are widely used as the marker genes for inorganic-PSM [30] and organic-PSM [31] respectively, were amplified from the samples via PCR (Fig. S2A; Table S1). Bright bands specific to *pqq*C and *pho*X were observed from the sewage incubated under MM-sP (Fig. S2A), confirming the presence of both inorganic- and organic-PSM in this microbiota.

Next, to identify the cells with in situ PSM activity, we profiled the microbiota directly under the native condition of the sewage sample via SCRS. To avoid the interference by soluble organic-P (Fig. S2B; “Methods”), an aliquot of the sample was dephosphorized and sterilized, by employing FeSO_4_^·^7H_2_O to deplete such bioavailable P (reducing soluble P from 3 mg/L to <0.02 mg/L; Fig. S2C and Fig. S2D) and then removing all cells via a 0.22 μm filter. From another aliquot of this sewage sample, cells were extracted (“Methods”), added into the above dephosphorized and sterilized aliquot (containing 50% D_2_O) and incubated for 24 h under no P (P_free), soluble phosphate (sP), tricalcium phosphate (iiP) and lecithin (oiP), respectively (“Methods”).

For each of the substrate-specific conditions, 100 randomly sampled cells were analyzed for SCRS (Fig. 3C, D). In the P_free group, CDR are all very low (<0.08), but much higher upon the presence of soluble P (ranging from 0.02 to 0.22 in the sP group; Fig. 3D). In the iiP group, CDR of the 100 cells sampled are all < 0.15 (Fig. 3D), indicating the presence of inorganic-PSM yet few with strong P-solubilizing activity (as judged by the CDR threshold of 0.20). In contrast, in the oiP group, CDR span from 0.02 to 0.21, indicating the presence of both weak organic-PSM and strong organic-PSM (similarly, CDR threshold of 0.20 as criterion for strong P-solubilizing activity). Specifically, in the oiP group, cells with CDR > 0.20 are present at 9% frequency (Fig. 3D). These organic-PSM with efficient in situ activity in sewage are thus the target cells for our ‘screen-first culture-second’ strategy via scRACS-Culture.

### Deriving pure cultures of organic-PSM from functionally sorted single cells by scRACS-Culture

**Fig. 4:**
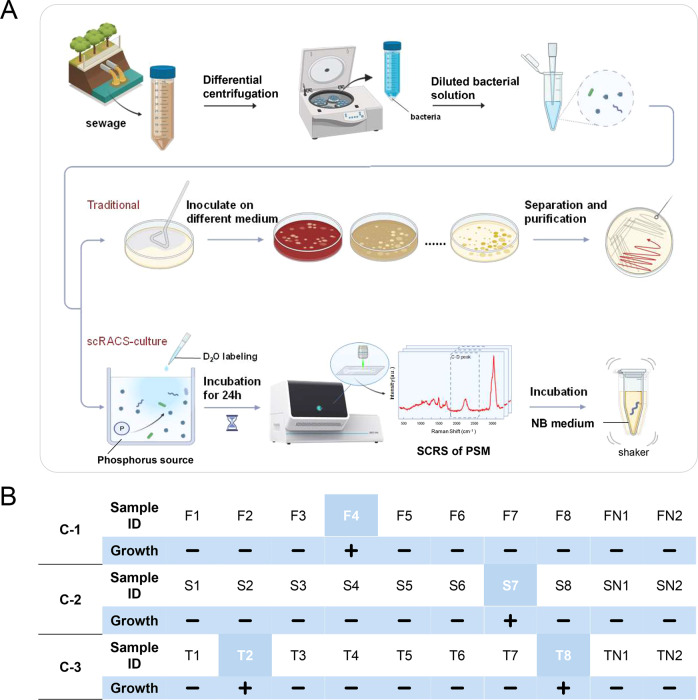
One-cell RACS-Culture of metabolically active organic-PSM cells from sewage. **A** The workflow of scRACS-Culture that identifies and isolates inorganic PSM from the sewage samples. **B** Results from culturing the isolated single organic-PSM cells in each receiving tube of 20 μL broth medium, respectively. In Tube FN1, FN2, SN1, SN2, TN1 and TN2, empty droplets (i.e., without cells) were sorted and cultured. In Tube F1-F8, S1-S8, T1-T8, the targeted organic-PSM cells were sorted and cultured in a one-cell-one-tube manner.

To test the feasibility of scRACS-Culture directly from the environment, extracted cells were incubated for 24 h, in the sewage which was first dephosphorized and then supplemented by lecithin as the only P source (with 50% D_2_O; Fig. 4A; “Methods”). Employing a RACS-Seq instrument equipped with a Raman-activated Gravity-driven microdroplet Encapsulation (RAGE) module (“Methods”), the cells were analyzed individually via SCRS (“Methods” [11, 32]). Then, cells recognized as strong organic-PSM in situ by SCRS (i.e., with CDR > 0.20) were sorted with a laser tweezer to form droplets in a one-cell-per-droplet manner respectively. In each scRACS-Culture run (each with triplicates), 8 individual cells were sorted from the D_2_O-labeled sewage microbiota in a one-cell-one-tube manner, respectively (Fig. 4B; Fig. S3). Two blank samples (with an empty droplet in each) were included as negative controls in each batch of experiments (Fig. 4B; Fig. S3).

A total of 24 target P-solubilizing cells (CDR > 0.20) and six negative controls (empty droplets without any cells) were obtained from the three scRACS-Culture runs (Fig. 4B; Fig. S3). To validate the cultivation for each of the scRACS-derived cells, pL-volume culture was employed for seven days, following by μL volumes for another seven days in broth medium (“Methods”). This resulted in four single-cell-derived organic-PSM cultures, which were identified by 16S rRNA gene, including *Comamonas* spp. (Tube F4), *Acinetobacter* spp. (Tube S7), *Enterobacter* spp. (Tube T2) and *Citrobacter* spp. (Tube T8; Fig. 4B; Fig. S3; File S2).

**Fig. 5:**
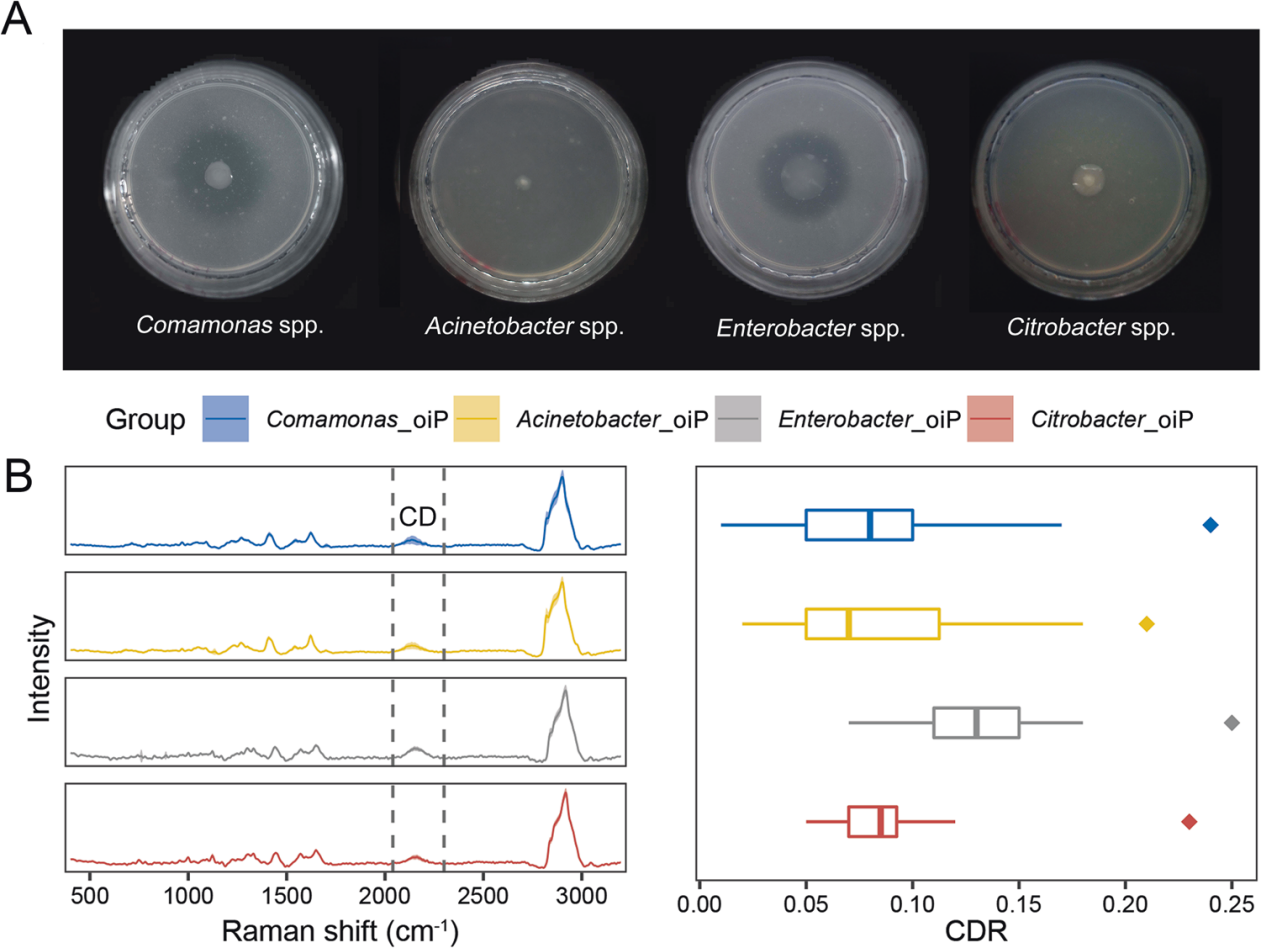
The phosphorus-solubilizing activity of the scRACS-Culture-derived bacterial strains of *Comamonas* spp., *Acinetobacter* spp., *Enterobacter* spp. and *Citrobacter* spp. **A** Halo zones produced on the solid medium of MM-iiP and MM-oiP for those four strains, respectively. **B** The SCRS and C-D ratios of the four strains, which were incubated in broth media containing 50% D_2_O with Ca_3_(PO_4_)_2_ (iiP) and lecithin (oiP), respectively. Sixty individual cells from each biological replicate sample were measured.

When plated on solid medium that contains only organic P, these four strains all produce highly visible halo zones around their colony, confirming organic-P solubilizing activities (Fig. 5A). Intriguingly, for each strain, the organic-P solubilizing activity in pure culture is much lower than that in situ. The former was measured by assessed the cultured cells in MM-oiP with 50% D_2_O (“Methods”), where SCRS from 60 cells randomly acquired for each strain (in triplicates, with 20 cells for each biological replicate) revealed prominent C-D bands for all cells (Fig. 5B). However, the mean CDR are much lower, i.e., only 33–52% of those in situ (0.08 vs 0.24, 0.09 vs 0.21, 0.13 vs 0.25, and 0.09 vs 0.23 for *Comamonas* spp., *Acinetobacter* spp., *Enterobacter* spp. and *Citrobacter* spp. respectively; Fig. 5A; Fig. S4). As the PSM’s in situ P-solubilizing activities appear to be different (as suggested by the 90–200% higher CDR) from those in pure culture, these results underscore the necessity of screening cells based on in-situ instead of pure-culture-based measurements, and suggest scRACS-Culture as a feasible approach for directly identifying and then mining in situ functioning cells from the environment.

### One-cell, in-situ-function-driven, high-genome-coverage sequencing of efficient in situ organic-PSM via scRACS-Seq

**Fig. 6:**
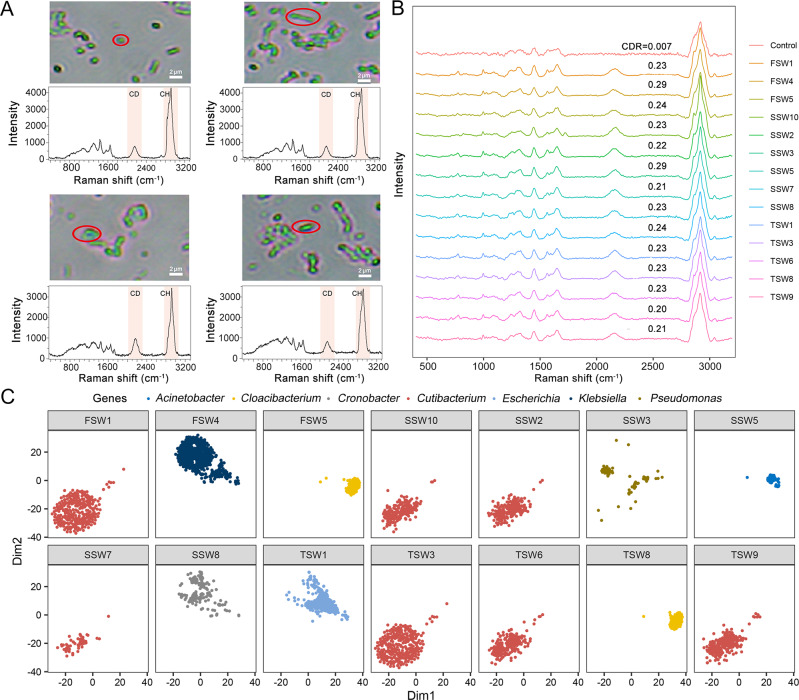
One-cell RACS-Seq of metabolically active organic-PSM cells from sewage. **A** Organic-PSM cells, with varying morphological features, as identified in the sewage based on SCRS. **B** SCRS of the C-D band-containing cells, which were treated with D_2_O for 24 h and sorted via the threshold of CDR > 0.20 for single-cell genomes. SCRS of ‘FSW1, FSW4, FSW5, SSW2, SSW3, SSW5, SSW7, SSW8, SSW10, TSW1, TSW3, TSW6, TSW8 and TSW9’ represent cells with the C-D band. SCRS of ‘control’ correspond to cells without CD band. CDR = Area (C-D)/(Area(C-D) + Area(C-H)). **C** Taxonomical origin of the one-cell scRACS-Seq derived organic-PSM genomes based on the t-SNE projection of binned contigs. Contigs are visualized based on 4-mer frequency features. Each contig is colored based on its taxonomic annotation at the family level.

To probe whether there are cells playing important function in situ yet recalcitrant to the growth in scRACS-Culture, we employed our recently established scRACS-Seq [11, 32] to establish one-cell-resolution links between in situ organic P-solubilizing activity and the genome sequence, directly from the sewage (Fig. 1; “Methods”). Cells from the D_2_O-probed sewage microbiota were analyzed individually via SCRS (Fig. 6A; “Methods”). Those cells that correspond to strong organic-PSM activity in situ (i.e., with CDR > 0.20; Fig. 6B) were sorted with a 1 064 nm laser tweezer, encapsulated, and exported in a one-cell-per-droplet manner respectively. In the scRACS-Seq process for sewage, we have added the “NCS” samples (“Negative Control of Sewage”; i.e., an empty droplet that harbors no cell) as a negative control in each batch of experiment (as shown in Fig. S5). No 16S rRNA genes were amplified from the NCS samples (Fig. S5), suggesting the low likelihood of introducing polluting cells or DNA in our workflow (Additional details on contamination assessment and control during scRACS-Seq were described in our former study [32, 33]). Each of the one-cell-encapsulated droplets was then transferred to a PCR-tube for single-cell lysis and Multiple Displacement Amplification (MDA). To assess the quality of MDA for each RAGE-derived cell, the 16S rRNA gene was amplified by PCR using the MDA product as the template (Fig. S5). Successful one-cell MDA products were then shotgun-sequenced in a one-cell-per-tube manner. Finally, for each cell that carries the SCRS-based P-solubilizing phenotype, the corresponding genome sequence was derived via *de novo* assembly of the shotgun reads and then interrogated via in silico metabolic reconstruction.

For each sewage sample (in three biological replicates of S-1, S-2, and S-3), among the hundreds of cells screened via SCRS, 10 environmental cells that correspond to strong organic-PSM (based on the criterion of CDR > 0.20) were sorted and sequenced respectively (i.e., 30 cells in total; Fig. 6B). Then, 15 one-cell MDA products each with both clear MDA bands and positive 16S rRNA PCR results were chosen for subsequent 16S rRNA and whole-genome sequencing (Fig. S5A–C), producing ~3 Gb of raw sequencing data for each cell: FSW1, FSW4, FSW5, SSW2, SSW3, SSW5, SSW7, SSW8, SSW10, TSW1, TSW3, TSW6, TSW8 and TSW9 (except FSW7 which failed to yield sequencing library due to severe degradation; Table 1). For S-1, S-2, and S-3, the success rate of one-cell RAGE-Seq (i.e., number of successful experiments divided by total number of attempts, with ‘success’ defined as the ability to produce from MDA product the target 16S-rRNA via PCR plus the validation by single-cell whole-genome sequencing) was 30, 60 and 50%, respectively (average of 46.7%).

**Table 1:** Predicted genome completeness and species abundance of RAGE-sorted bacteria in the sewage sample.

Type	Experiment series	RAGE-sorted organic-PSM samples	Class (Genus)	Estimated genome completeness by CheckM (%)	Contamination	Abundance in the community based on 16S rRNA sequencing	References supporting phosphorus dissolution function
scRACS-Seq	S-1	FSW1	*Cutibacterium*	90.48	3.47	0.01%	–
		FSW4	*Klebsiella*	97.44	105.5	0.14%	Kim et al. [94]
		FSW5	*Cloacibacterium*	40.85	5.17	1.58%	Gay et al. [95]
	S-2	SSW2	*Cutibacterium*	33.07	0.86	0.01%	–
		SSW3	*Pseudomonas*	9.59	0.68	1.27%	Cai et al. [96, 97]
		SSW5	*Acinetobacter*	15.52	1.72	1.86%	Chen et al. [98, 99]
		SSW7	*Cutibacterium*	96.94	0.92	0.01%	–
		SSW8	*Cronobacter*	50.12	1.85	–	Liang et al. [100, 101]
		SSW10	*Cutibacterium*	30.27	2.66	0.01%	–
	S-3	TSW1	*Escherichia*	39.24	4.31	0.27%	Choi et al. [102, 103]
		TSW3	*Cutibacterium*	61.53	3.67	0.01%	–
		TSW6	*Cutibacterium*	66.48	1.96	0.01%	–
		TSW8	*Cloacibacterium*	77.74	2.59	1.58%	Gay et al. [95]
		TSW9	*Cutibacterium*	86.39	4.44	0.01%	–
scRACS-Culture	C-1	F4	*Comamonas*	–	–	1.42%	Yaghoubi Khanghahi et al. [104]
	C-2	S7	*Acinetobacter*	–	–	1.86%	Chen et al. [98, 99]
	C-3	T2	*Enterobacter*	–	–	–	Adhikari et al. [105]
		T8	*Citrobacter*	–	–	–	Patel et al. [106]

For each of these efficient in situ organic-PSM cells, a single-cell amplified genome (SAG) was assembled (“Methods”), in which GC% of the assembled contigs (>200 bp; after decontamination) exhibits a normal distribution (Fig. S6). The taxonomy of each SAG was determined by the top contig bin, which was in turn assigned based on BLASTN of each contig to the NCBI NT database (Table S2). Accordingly, the SAGs were pinpointed as *Cutibacterium* spp., *Klebsiella* spp., *Cloacibacterium* spp., *Pseudomonas* spp., *Acinetobacter* spp., *Cronobacter* spp., and *Escherichia* spp., respectively (Table 1). Taxonomy assignment via the GTDB database yield essentially identical results (Table S2). Moreover, t-SNE projection of contigs from each cell (>1500 bp) via their 4-mer signatures revealed distinct clustering patterns that are characteristic to the respective taxa assigned (Fig. 6C). These results support the accuracy and integrity of the assembled precisely one-cell genomes of these efficient in situ organic-PSM.

CheckM [34], which is based on lineage-specific marker genes, revealed that genome completeness of these precisely one-cell SAGs is up to 97.4%. The average one-cell-derived genome completeness at each sewage-microbiota RAGE run is 76.3, 78.5 and 66.3% respectively (Table 1), which is approximately three-fold of that in the recent report for the high-throughput single-cell genome sequencing of gut microbiota via Microbe-Seq (<25% for each cell; [35]). Thus, the one-cell RAGE-Seq can recover high-completeness one-cell genomes of targeted in situ function directly from complex microbiota.

Notably, other than *Cutibacterium* spp. which is new, each of the six phyla associated with the SAGs (*Klebsiella*, *Cloacibacterium*, *Pseudomonas*, *Acinetobacter*, *Cronobacter* and *Escherichia*) include strains that were previously shown to dissolve organic insoluble P (Table 1). For example, *Pseudomonas stutzeri* DSM4166 and *Pseudomonas fluorescein* SBW25 employ secreted glycerophosphodiesterases, GlpQI and GlpQII, respectively, to rapidly degrade the phospholipids in organic P fractions of soil, resulting the release of bioavailable P [36]. For *Acinetobacter*, batch experiments with pure cultures showed that P release was dependent on the organic substrate, growth phase and strain used [37].

Interestingly, of the 14 sequenced organic-PSM cells, nine are from low-abundance genera of this sewage sample (seven from *Cutibacterium* spp., one from *Klebsiella* spp., and one from *Escherichia* spp.), as suggested by the relative abundance of their 16S rRNA genes in the corresponding metagenome (<0.3% at the OTU level for each genus; Table 1; Table S3; File S1). Therefore, our SCRS-based approach can effectively identify efficient in situ organic-PSM in a culture-independent manner, even for low-abundance taxa.

### Mechanism-based discovery of *Cutibacterium* spp. as a new in situ organic-PSM via its one-cell-derived genome and metabolic phenome

*Cutibacterium* spp., which was not previously known as P-solubilizing, accounts for seven of the 14 SAGs (SW1, SSW2, SSW7, SSW10, TSW3, TSW6, and TSW9; with pairwise 16S similarities between them all > 97%), and each of the seven cells exhibit strong organic-P solubilizing activity in situ (showing CDR of >0.20 with lecithin as the only P source). These observations suggest this species’ dominating contribution to lecithin mineralization in this sewage sample. To probe how this occurs, we focused on the SSW7 cell which features the highest genome completeness (96.64%, vs. the average of ~74.21%; Table 1).

To verify SSW7’s phenotype, we first tested a cultured strain of *Cutibacterium acnes* BNCC 336443 for its P-solubilizing ability, by SCRS-based profiling of metabolic activity in MM-iiP and MM-oiP containing 50% D_2_O (“Methods”). Under MM-iiP and at 24 h, the SCRS from 60 individual bacterial cells (in triplicates, with 20 cells for each biological replicate) exhibited high metabolic activity as revealed via CDR (from 0.11 to 0.23; Fig. S7). Under MM-oiP and at 24 h, the CDR observed are nearly identical to MM-iiP (from 0.12 to 0.21; Fig. S7). which is slightly lower than that of SSW7 under the in situ condition in sewage (CDR = 0.23; Fig. 6B). These results lend additional support to the strong P-solubilizing activity of SSW7.

The 2.4 Mb genome of SSW7 which encodes 2297 protein-coding genes (Table S4) is classified as *C. acnes*, which can trigger acne inflammation in human skin by secreting degrading enzymes such as lipases, sialidases, neuraminidases, pore-forming factors, etc [38], yet organic P-solubilizing activity is not previously known for this taxon. Searches via the Microbiome Search Engine (http://mse.ac.cn; [33, 39] suggested that, based on 16S rDNA similarity, SSW7 (OTU4315338) are mainly present in soil, human or animals (e.g., skin and gut), yet with very low relative abundance (all < 2‰; “Methods”). Moreover, pan-genome analysis of the existing *C. acnes* genomes plus representative genomes from other members of the *Cutibacterium* genus (from NCBI RefSeq database; all with animal origin) indicates the clustering of SSW7 with *C. acnes* genomes (“Methods”), which further supports the soil, human or animal origin of SSW7.

For the solubilization of organic P, soil PSM’s toolbox includes three groups of enzymes [40]: (*i*) phosphatases (phosphohydrolase), which dephosphorylate phospho-esters or phospho-anhydride bonds, (*ii*) phytases, which specifically release P from phytic acid, and (*iii*) phosphonatases (and C-P lyases), which cleave C-P in organophosphates. Interestingly, no genes encoding the phytase or phosphonatase groups of enzymes were identified in the SSW7 genome. However, four genes from the phosphatase group were found (Table S5): *ushA* (5′-nucleotidase and UDP-sugar hydrolase; HCONNHDO_00279), *PPE1* (phosphoesterase; HCONNHDO_00716), *PPE2* (phosphoesterase; HCONNHDO_01140), and *MPP* (metallo-dependent phosphatase; HCONNHDO_01231). The *ushA* and *MPP* harbor signal peptides (TAT signal peptide; Tat/SPI) that indicate secreted proteins [41], while *ushA*, *PPE1* and *PPE2* carry features (the sortase motif of LPxTG) consistent with potential substrates of sortase F, which suggests their covalent attachment to cell surface [42].

**Fig. 7:**
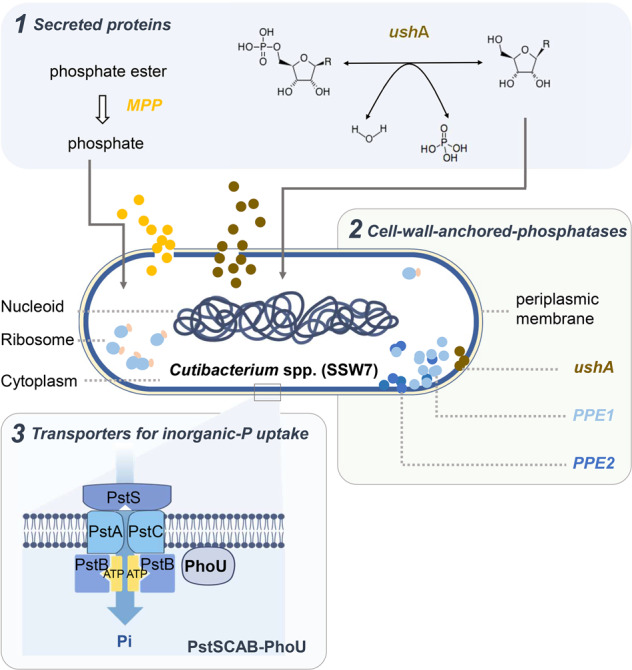
Phosphorus dissolution mechanisms derived from the one-cell organic-PSM genome of SSW7 (annotated as *Cutibacterium* spp.). Two secreted phosphatases (*ushA* and *MPP*), three phosphatases proteins (*ushA*, *PPE1* and *PPE2*), and the PstSCAB-PhoU gene cluster are key components that underpin the strong organic-P solubilizing phenotype of SSW7 and related strains in the sewage.

The *ushA* can be secreted extracellularly and anchored to the cell wall [41, 42]. Specifically, in *E. coli*, *ushA* is a periplasmic enzyme that can degrade nicotinamide adenine dinucleotide (NAD) [43]. In *Corynebacterium glutamicum*, *ushA* encodes a secretary enzyme that possesses 5′ nucleotidase and UDP-sugar hydrolase activities [44], which is induced under P starvation to use nucleotides as the sole P source [44]. Therefore, it is likely that nucleic acids are a vital source of organic-P for SSW7 (Table S5; Fig. 7).

The two potential phosphoesterases of *PPE1* and *PPE2* found in SSW7 are also likely anchored to the cell wall [42], which can cleavage the phosphate group from phospho-esters close to the cell (Table S5; Fig. 7). Therefore, these enzymes likely endow *C. acnes* the ability to scavenge the P nutrient from surface of particles that contain organic insoluble P substrates.

The MPPs are a group of secretory metallophosphoesterases involved in organic-P solubilization [45–47]. It harbors a conserved domain with an active site consisting of two metal ions (manganese, iron, or zinc) coordinated by an octahedral cage of histidine, aspartate, and asparagine residues [41, 48, 49]. Interestingly, the SSW7-encoded MPP gene belongs to a small collection of probable metallophosphoresterases (NCBI HMM accession: TIGR03767) that are widespread among the class of *Actinomycetia*, including *Cutibacterium* spp. (Fig. S8 red column) and *Streptomyces* spp. (Fig. S8; “Methods”).

To reuse the P liberated from organic-P, transportation systems of inorganic P are required. Bacteria often employ the high-affinity P transport systems PstSCAB-PhoU and PhnCDE, and the low-affinity PitH transporters [50]. In SSW7, we discovered a complete PstSCAB-PhoU gene cassette that copes with the Pho regulon to regulate intracellular inorganic P [51–53]. Besides, the P-binding protein PstS, which resides in the periplasm, is among the components of *C. acnes*-derived extracellular vesicles (EVs) [54]. Therefore, it is likely that SSW7 transports inorganic P not only through passively contacts but via active transport by the EVs.

Collectively, metabolic reconstruction from the one-cell derived genome suggested a mechanistic model for the strong organic-P solubilizing phenotype by SSW7 and related strains in the sewage (Fig. 7): (*i*) two secreted phosphatases (*ushA* and *MPP*) produce phosphate around the cells by cleaving the phosphate group from free nucleotides and esters such as phosphodiester and phosphotriester; (*ii*) three cell-wall-anchored phosphatases (*ushA*, *PPE1* and *PPE2*) possibly form biofilm on organic particles and produce phosphate from such particles near cell surface; (*iii*) the PstSCAB-PhoU gene cluster is probably responsible for inorganic-P uptake. Considering that SSW7-related cells represent a major portion of strong PSM cells as identified by scRACS-Seq in this sewage sample (7 out of the 14 SAGs of PSM), these genetic features likely underpin the efficient in situ organic-P solubilizing activity of SSW7, and can explain this low-abundance species’ significant ecological role in sewage.

## Discussion

At present, strategies for function-based screening of live single cells directly from microbiomes are quite limited. For example, in fluorescence-activated cell sorting (FACS), individual cells are encapsulated in microdroplets, incubated to derive pure cultures, and then assays designed to detect and screen the culture’s metabolic activity via fluorescent probes [3, 55, 56]. However, the need for fluorescence probes, the detection of function based on pure culture instead of in situ condition, and the low cell survival rate after fluorescence labeling has hindered broader application of this strategy.

Here we introduced a SCRS-based ‘screen first’ approach: scRACS-Culture does not require fluorescence labeling, and screens individual cells based on the in situ activity before deriving for pure cultures; moreover, the scope of metabolic activities assessable via a SCRS, which is much broader than the typical fluorescence signal, is still rapidly expanding [6, 57]. Thus, such fluorescence-probe free, in-situ-phenome-based screening of live single cells is more versatile and more widely applicable for microbiome mining. However, key to the RACS-based recovery of live cells from microbiota is the ability to maintain their vitality during SCRS acquisition and sorting, since laser radiation during SCRS acquisition can cause damage to vitality [13, 58]. In fact, post-RACS cultivation was described only for Raman-tweezer-based approaches that sort cells in the liquid phase. Specifically, for either pure *Marinobacter adhaerens* cultures [59] or mouse colon microbiota [60], the pool of sorted bacterial cells was cultivated on agar plates or in lipid medium. However, when culturing the sorted cells as a mixture instead of in a “one-cell per-tube” manner, it is difficult to gauge whether each of the sorted cells is actually successfully cultivated; this is however an important question because the cultivability of each sorted cell is theoretically unique in a microbiota. Thus, the feasibility of single-cell-resolution RACS-Culture (i.e., scRACS-Culture) remains an open question. This is particularly challenging when non-resonance Raman peaks are used as marker, since detection of non-resonance Raman peaks would require much longer laser exposure time than resonance peaks. We have tackled this problem by designing a RAGE chip which integrates SCRS acquisition, cell sorting, target-cell encapsulation microdroplet, and export of one-cell microdroplets into an aqueous on-chip process, where the water phase protects vitality of cells from laser energy yet allows acquisition of high-quality SCRS for detecting signal of non-resonance Raman peaks such as that of the C-D peak. We have previously showed that the RAGE process can fully preserves cell viability even for those sorting based on non-resonance Raman peaks (e.g., the C-D peak), since no apparent change in the viability of *E. coli* cells was found between laser-exposed and non-exposed cells [11].

Via scRACS-Culture, we derived pure cultures of organic PSM based on their efficient in situ P-solubilizing activities such as *Comamonas* spp., *Acinetobacter* spp., *Enterobacter* spp. and *Citrobacter* spp. Surprisingly, their in situ activities are all 90–200% higher than in pure culture (direct comparison of the activity between the two media is not possible as CDR is medium-dependent). This suggests that the scRACS-Culture-based assessment of in situ activity are more accurate and reliable, while those based on pure cultures can be distorted or even misleading. These advantages are clearly important to functional evaluation and mining of cells or their products from a microbiota.

On the other hand, for those post-RACS functional cells that are recalcitrant to cultivation, we have used scRACS-Seq to unveil the genomic foundation of the targeted metabolic activity. A major challenge for scRACS-Seq has been obtaining high-genome-wide coverage from the post-RACS one cell, which is pivotal to the quality of metabolic reconstruction. This key parameter, which reaches up to 96.94% here in sewage microbiome for phenotypic screening based on non-resonance Raman peaks, should be generally applicable to all phenotypes that a SCRS can model, since resonance-peak-based screening would require much lower laser power in SCRS acquisition and higher genome coverage is expected [13]. Notably, the performance is much higher than recent studies via Raman-activated Cell Ejection (RACE; [11, 61], either for pure cultures (e.g., ~16.43% for 5-*E. coli*-cell mixtures; [13], for Red Sea microbiomes (<10% for one-cell reactions; 8.95 and 19.29% for 8-cell pools; [62], or for Yellow Sea microbiomes (max. of 13.66% for 30-cell pools [63]). Together with our recent demonstration of deriving one-cell high-coverage genomes after RACS, such as 99.48% from urine [11], 99.70% from stomach [10] and 92.62% from soil [32], our findings further support the high genomic data quality and robustness of scRACS-Seq for various kinds of microbiome samples.

Using scRACS-Seq, we found that one group of Actinobacteria that is phylogenetically very close to the predominant skin symbiont of *C. acnes*, despite their very low abundance in the sewage sample, makes a disproportionally large and perhaps the most important contribution to lecithin solubilization in this particular industrial water purification process. Such a role is supported by both in situ activity-based phenotypic measurement as well as genetic evidence. As suggested by the high-coverage one-cell genome assembly, we hypothesis that this group of organisms might employ secretary metallophosphoesterase (MPP), cell-wall-anchored 5′-nucleotidase (*ush*A) and periplasmic-membrane located PstSCAB-PhoU transporter system for efficient solubilization and scavenging of extracellular phosphate in sewage (further experimental evidences are required to test the hypothesis). *Cutibacterium* spp. are not previously known for P-solubilization activities, thus our findings here suggest the potential uses of this group of bacteria as live-cell stimulants to accelerate P-solubilization in water purification plants. Moreover, based on these findings, we speculate that there may be an internal *C. acenes*-mediated link between P metabolism on the skin and in the sewage. As P-containing compounds are applied in many cosmetics [64], it would be intriguing to probe whether *C. acenes* or related species play a role in P metabolism on the human skin.

Notably, although we have obtained an array of strong PSMs in live pure cultures via scRACS-Culture, the sewage *Cutibacterium* spp. cells that we sequenced via scRACS-Seq remain uncultured. Clearly, despite its ‘screen-first’ feature, scRACS-Culture is still a cultivation-dependent method, and its potential is constrained by the cultivability of the sorted cells in an artificial medium. To improve the success rate of scRACS-Culture, we envision a strategy that unlocks the target-cell’s nutrient needs via metabolic reconstruction of its genome from scRACS-Seq; the knowledge can then be exploited to optimize the culture medium [65, 66]. Moreover, the throughput and automation of the scRACS-Culture workflow should be elevated. For example, flow-mode scRACS-Culture can be designed that couples microdroplet-based massive parallel one-cell cultivation (via advanced microfluidics [1, 67]) to the high-throughput RACS technologies (e.g., the pDEP-RADS based FlowRACS [12]).

Finally, it is important to note that, in a complex microbiota, CDR might not be directly correlated with P-solubilizing activity across different taxa. This is because CDR depends on how much deuterium is incorporated from heavy water into a cell’s biomass. Different taxa have different metabolisms which can lead to distinct levels of CDR, even if they are equally efficient at P solubilization. For example, as deuterium is known to be preferentially incorporated into lipids [28], cells with distinct lipidic contents may naturally differ in CDR value even when they show identical P-solubilizing activities. Therefore, by setting the CDR threshold of 0.20 to screen for high-efficiency organic PSM, our scRACS-Culture procedure might be selecting not only high-efficiency organic PSM but taxa that incorporate high-levels of deuterium, and similarly, we might have left out taxa that are also efficient at solubilizing P but do not display high CDR. As a result, the P-solubilizing efficiency of those cultures obtained by scRACS-Culture should be confirmed via a CDR-independent approach when possible.

In summary, the combined use of scRACS-Culture and scRACS-Seq provides an in situ function-based, ‘screen-first’ approach for directly assessing and mining from the environment those functioning microbes that are either culturable or not-yet culturable. As SCRS can measure a wide range of metabolic phenotypes in a fluorescence-probe-free, non-invasive manner, this approach is generally applicable, and should greatly expand the use of function-driven single-cell technologies in microbiome science and industries.

## Materials and methods

### Strains, media, and culture conditions

Bacterial strains used for benchmarking include three inorganic-PSM (*B. subtilis* H6, *E. coli* BL21 and *B. megaterium* ACCC02970), two both inorganic- and organic- PSM (*A. hydrophila* ATCC7966 and *B. cepacian* BN337012), and one non-PSM (*E. faecium* PH07). Among them, *B. subtilis* H6 and *E. faecium* PH07 were isolated in our laboratory; *Bacillus megaterium* ACCC02970 was purchased from the Agricultural Culture Collection of China; *A. hydrophila* ATCC7966 was from the First Institute of Oceanography, Ministry of Natural Resources; *B. cepacian* BN337012 and *Cutibacterium acnes* BNCC 336443 were from the Bena Culture Collection, China. Pure cultures of organic-PSM derived from those post-RACS single cells included *Comamonas spp*., *Acinetobacter spp*., *Enterobacter spp*. and *Citrobacter spp*., which were cultivated in broth medium.

A series of minimal media (MM) supplemented with different forms of P were used in this study. MM without P (MM-P_free) was prepared by adding 10.0 g of glucose, 0.5 g of (NH_4_)_2_SO_4_, 0.3 g of NaCl, 0.3 g of KCl, 0.15 g MgSO_4_, 0.03 g of FeSO_4_·7H_2_O, 0.03 g MnSO_4_·H_2_O into 1000 ml of ultrapure water. MM with dissoluble P (MM-sP) was prepared by dissolving 0.5 g/L of KH_2_PO_4_ and 1.5 g/L of Na_2_HPO_4_. MM with tricalcium phosphate (which represents inorganic insoluble P; MM-iiP) was prepared by adding 2.5 g/L Ca_3_(PO_4_)_2_ as the only P source into MM-P_free. Lecithin is a frequently used model organophosphorus compound, a widely present organic form of insoluble P, and among the most difficult organophosphorus compounds to break down [24]. The excessive accumulation of lecithin in soil and water can stimulate microalgal overgrowth and increase eutrophication risk [68–70]. Lecithin is decomposed only under extreme physical/chemical conditions, e.g., high temperature, extreme pH, dehydration, freezing, chilling, and high mineral concentrations [71, 72], thus biological strategies to reduce lecithin levels are of interest. Therefore, we employed minimal media containing lecithin (MM-ioP) as the only P source (amended with D_2_O) for bacterial incubation. In this study, MM with lecithin (which represents organic insoluble phosphorus; MM-oiP) was prepared by amending 0.5 g/L lecithin as the only P source into MM-P_free.

For deuterium-isotope based probing, 50% D_2_O (vol/vol; 99.9 atom% D; Sigma-Aldrich, Canada) was used. The various bacterial strains were incubated in a culture plate at 30 °C for different time periods for the D_2_O labeling prior to the acquisition of single-cell Raman spectra.

### Determination of phosphorus-solubilizing activity for pure cultures of PSM

Tricalcium phosphate (iiP) and lecithin (oiP) were used as the sole phosphorus sources to test the microbial ability to dissolve organic or inorganic P respectively. For solid medium, the P-solubilizing ability of a strain was determined via the method of dissolved P transparency circle [73]. Specifically, the pure cultures were inoculated on inorganic- or organic-P solid medium plates at 30 °C, respectively, and the dissolved P transparency circles were examined after ~7 days.

For liquid medium, the P-solubilizing ability of a strain was determined by the P content in supernatant via the molybdenum-antimony resistance colorimetric method [73]. *E. faecium* PH07, *E. coli* BL21 and *A. hydrophila* ATCC7966 were inoculated in the LB medium. *B. subtilis* H6, *B. megaterium* ACCC02970 and *B. cepacian* BN337012 were inoculated in the nutrient broth culture medium. All bacteria were activated and cultured by shaking at 30 °C. When OD_600_ reached 0.6~0.8, the samples were washed with ultrapure water for three times at 10,000 rpm to obtain bacterial suspension, respectively. Then 200 μL bacterial solution was added into a centrifuge tube that contains 30 mL liquid media for inorganic- or organic-P, respectively, and an identical volume of sterile water was added as negative control. Then, each sample was cultured by shaking at 180 rpm and 30 °C with three replicates. After five days, the culture collection was centrifuged at 4 °C at 8000 r/min for 10 min, and the P content in the supernatant was measured by molybdenum-antimony resistance colorimetry.

### Sample preparation for profiling P-solubilizing activity of sewage microbes in artificial media

The FastCell Extraction Kit for sewage (Lot: SCR013020, Qingdao Single-cell Biotechnology, Qingdao, China) was used for extracting the microbial cells from sewage samples. The cells were inoculated into the medium of MM-P_free/sP/iiP/oiP (with 50% D_2_O). After 24 h incubation at room temperature, the samples were washed three times at 8000 rpm for 3 min each time. Finally, the cell pellets were resuspended in 0.2 mL ddH_2_O and then pointed onto the chip for downstream acquisition of single-cell Raman spectra.

### Removal of phosphorus and microbial cells from sewage samples to create the in situ condition

Based on the soluble P content as measured by molybdenum-antimony resistance colorimetry (based on a P-content standard curve; Fig. S2B) [73], a proper concentration of FeSO_4_·7H_2_O ([P]:[FeSO_4_·7H_2_O] = 1:2 mol) was added into the sewage sample. The sample was mixed by shaking for 20 min and centrifugated at 8000 rpm for 5 min. Then the supernatant was filtered by a 0.22 μm filter to obtain the dephosphorized and sterilized solution. In the end, the soluble P content of the solution was measured to verify the efficacy of dephosphorization.

### Sample preparation for testing the in-situ activities of microbial cells in sewage

One mL of the dephosphorized and sterilized sewage was added into a centrifugal tube for the four groups of ‘P_free’, ‘sP, ‘iiP’ and ‘oiP’, respectively, and the volume of per tube was reduced to 300 μL with a centrifugal concentrator. Then, 500 μL D_2_O and 100 μL of the sewage solution (with the microbial cells extracted from another 1 mL sewage samples by the FastCell Extraction Kit for Sewage (Lot: SCR013020, Qingdao Single-cell Biotechnology, China)) were added into each of the four groups, respectively. Subsequently, 100 μL H_2_O was added into the tube of ‘P_free group’; 100 μL sP storage solution (0.5 g/L KH_2_PO_4_ and 1.5 g/L Na_2_HPO_4_) into the tube of ‘sP group’; 100 μL Ca_3_(PO_4_)_2_ storage solution (25 g/L Ca_3_(PO_4_)_2_) into the tube of ‘iiP group’; 100 μL lecithin (5 g/L lecithin) into the tube of ‘oiP group’. Then, the sewage microbial cells were added into the four groups of liquid substrates for room temperature incubation at 200 rpm for 24 h. The resulting samples then proceeded to the downstream SCRS acquisition and Raman-activated single-cell sorting.

### Acquisition of Single-cell Raman spectra of sewage microbiome in scRACS-Culture

The scRACS-Seq/Culture procedure was performed on a RACS-Seq system (Qingdao Single-cell Biotech, China), and all the SCRS were also acquired via the platform. For scRACS-Seq, the ‘sewage cell extracts’ were washed to remove residual media before the SCRS acquisition, and then resuspended by adding deionized water to dilute for performing the subsequent Raman microspectroscopy and sorting. For scRACS-Culture, ‘sewage cell extracts’ of the oiP group was resuspended in broth medium and underwent the subsequent single-cell SCRS acquisition, sorting and cultivation.

Specifically, the prepared bacterial cell suspensitions (~1 mL) was hanged on the sample holder and then loaded into the RAGE chip. Raman spectra were acquired with a confocal Raman microscope in the RACS-Seq system, where 50× dry objective was used for sample signal acquisition and optical tweezers, while a 10× dry objective for observing droplet generation and exportation. Cells with C-D band in SCRS were isolated using a RAGE chip [11, 32]. Selection of post-SCRS-acquisition cells to be sorted was via a customized criterion of CDR > 0.20 (derived by dividing C-D peak area from 2040 to 2300 cm^−1^ by the sum of C-D area and C-H peak area from 2800 to 3100 cm^−1^). The computational pipeline used here is available on github (https://github.com/gongyh/RamanD2O).

### Isolation of individual organic-PSM cells from the sewage sample by RAGE

As explained in Fig. S1, Microbiome cells in aqueous medium were injected into the RAGE chip with the height adjustable sample holder. The well was filled with mineral oil (2% wt EM90) when the cell phase reached the open well. The sample holder height was adjusted to obtain a balance between the water phase and oil phase. Cells located statically in the detection window were trapped and analyzed with the 532 nm laser to identify the cells with targeted metabolic activity based on SCRS. Then, the laser was switched to 1064 nm, to trap and move a single target cell to the edge of the aqueous phase, while clear other cells near the tip to ensure single-cell encapsulation. The sample holder was elevated to generate only one droplet, then lowered to the original height, so a single target cell is isolated and encapsulated within the droplet. The density of oil used is lower than water, so the droplet stays at bottom of the open well. Finally, the droplet with the target cell was readily removed via a pipette or capillary tube for the downstream cell lysis (for MDA) or cultivation.

### Multiple displacement amplification (MDA) for single-cell genome sequencing in scRACS-Seq

Lysis of the post-RAGE cells was individually carried out at 65 °C for 15 min with 1.5 µl lysis buffer for each of the one-cell samples, followed by addition of 1.5 µl stop solution to neutralize the lysis buffer. Reaction Buffer and DNA Polymerase were added and the mixture was incubated at 30 °C for 8 h with 70 °C hot-lid temperature for MDA reactions. Blank control (without any cells) was included to detect and quantify potential contamination. After that, the MDA products were processed for 16S rRNA gene PCR analysis using the 27F and 1492R primers (Table S1). Once the MDA products of single-cell genomic DNA were confirmed to contain single-species 16S-rRNA, they would undergo high-throughput sequencing.

### Library construction and sequencing of the sewage microbiota or the single cells

#### 16S-rRNA gene sequencing

Total genome DNA from the sewage sample was extracted using TIANamp Bacteria DNA Kit (Tiangen, China). It was processed for the detection of marker genes inorganic-PSM (*pqq* C) and organic-PSM (*pho* X; Table S1). In addition, 16S rRNA genes of the V4 regions were amplified using barcoded 515F and 806R. PCR reactions were carried out with 15 μl of Phusion® High-Fidelity PCR Master Mix (New England Biolabs), 2 µM of forward and reverse primers, and 10 ng template DNA. Thermal cycling consists of initial denaturation at 98 °C for 1 min, followed by 30 cycles of denaturation at 98 °C for 10 s, annealing at 50 °C for 30 s, and elongation at 72 °C for 30 s, and eventually 72 °C for 5 min. Sequencing libraries were generated using TruSeq® DNA PCR-Free Sample Preparation Kit (Illumina, USA). The library quality was assessed on a Qubit® 2.0 Fluorometer (Thermo Scientific) and an Agilent Bioanalyzer 2100 system. The library was sequenced on an Illumina NovaSeq platform which produced 250 bp paired-end reads.

#### Bacterial one-cell genome sequencing via scRACS-Seq

The MDA products from post-RACS single cells were treated with S1 Nuclease (Thermo Fisher Scientific, USA) to degrade the single-stranded nucleic acids, and then purified by Agencourt AMPure XP Beads (Beckman Coulter, USA). A total of 0.2 μg DNA per sample was used as input material for DNA library preparations. Sequencing libraries were generated using NEB Next® Ultra™ DNA Library Prep Kit for Illumina (NEB, USA), with index codes added to each sample. Briefly, genomic DNA sample was fragmented by sonication to a size of 350 bp. Then DNA fragments were end polished, A-tailed, and ligated with the full-length adapter for Illumina sequencing, followed by further PCR amplification. After the purification of PCR products by AMPure XP (Beckman Coulter, USA), DNA concentration was measured by Qubit® 3.0 Flurometer (Invitrogen, USA), and libraries analyzed for size distribution and quantified by real-time PCR. Clustering of the index-coded samples was performed on a cBot Cluster Generation System using Illumina PE Cluster Kit (Illumina, USA), and then the DNA libraries sequenced via 150 bp paired-end reads.

### Sequencing data analysis

#### Analysis of  16S-rRNA gene amplicons

QIIME version 1.9.1 [74] was used for 16S rRNA gene data analysis. The forward and reverse read pairs with minimum overlapping bases of 20 bp were joined and assigned to samples based on barcode and truncated by cutting off the barcode, primer sequence and low-quality bases (5’-end; quality score <20). Quality filtering on joined sequences was performed and sequences which did not fulfill the following criteria were discarded: sequence length > 200 bp and withno ambiguous bases. Then the chimeric sequences were removed using the UCHIME algorithm [75]. Putative contaminants were removed from datasets, as were singletons. The remaining high-quality reads were grouped into operational taxonomic units (OTUs) using the Vsearch algorithm [76], and aligned using default parameters against the Silva_132 database [77]. Representative sequences for the shared OTUs, as defined by 97% similarity, were obtained. Relative abundance of the bacterial taxa at the phylum, class, order, family, genus, and species levels was calculated and compared, respectively.

#### Analysis of bacterial one-cell genoms derived from scRACS-Seq

A computational pipeline for single-microbial-cell genome was applied (https://github.com/gongyh/nf-core-scgs) to efficiently analyze single-cell amplified bacterial genomes (SAGs) datasets, by integrating various tools with Nextflow [78]. Briefly, reads that passed illumina’s chastity filter were first quality checked using FastQC (https://www.bioinformatics.babraham.ac.uk/projects/fastqc/), and then quality trimmed using Trim Galore (https://www.bioinformatics.babraham.ac.uk/projects/trim_galore/) in the paired-end mode for each sample. To detect contaminated DNA fragments, clean reads were phylogenetically classified using Kraken [79]. Clean reads were then assembled into contigs using SPAdes [80] in single-cell mode. Taxonomic composition of assembled contigs (longer than 200 bp) was visualized using BlobTools [81]. Assembled genomes were annotated using Prokka [82], KofamKOALA [83] and eggNOG-mapper [84]. Considering the possibility of DNA contamination for environmental samples, assembled contigs were further split into bins by taxonomic annotations (in the genus level) for each SAG, followed by estimation of genome completeness using CheckM [34]. The identity of target cell was determined based on that of the top contig bin (which consists of contigs binned to the same taxonomical unit) [32].

#### Metabolic reconstruction based on one-cell-derived genomes

Protein-coding genes encoded in SSW7 were predicted based on Prokka v1.12 [85]. The secretary enzymes were screened based on literature mining and validated using SignalP v6.0 [86] and PSORTb v3.0 [87]. For functional annotation, the protein-coding genes in SSW7 were compared to the reference genome of *Cutibacterium acnes* strain KPA171202 (Accession AE017283.1). For the metallophosphoesterase (MPP) genes, a model of TIGR03767 family was retrieved from NCBI (Accession number: TIGR03767.1) and then the HMM model was used to find homologous genes from SAGs and UniRef50 database [88] using HMMER [89]. For phylogenetic analysis of MPPs, fragmented proteins were filtered, multiple sequence alignment and tree generation were performed using MAFFT [90], then the generated tree was visualized using iTOL [91].

### Single-cell cultivation of sewage-derived organic PSM via scRACS-Culture

The ‘sewage cell extracts’ of oiP group was resuspended in nutrient broth medium (NB medium; 10 g·L^−1^ peptones, 3 g·L^−1^ beef extract, 5 g·L^−1^ sodium chloride, PH = 7.2) for SCRS acquisition and sorting (criterion: CDR > 0.2) for subsequent single-cell cultivation. The NB medium was originally designed for use in the book of Standard Method for Examination of Water and Wastewater [92]. It is one of the several non-selective media commonly used in routine cultivation of microorganisms [92, 93]). Specifically, the sorted target cells were trapped in droplet and then exported into PCR tubes respectively in one-cell-one-tube manner using the RACS-Seq instrument. The first round of culture was conducted at 200 rpm in a 30° incubator in pL volume. After seven days of culture, 20 μL broth medium was added into PCR tubes for a second round of expansion under the same culture conditions, also for 7 days. If the single-cell culture is successful, the bacterial precipitates would be visually apparent in the 20 μL culture units.

## Supplementary information

Supplementary information

## Data Availability

The raw sequence data reported in this study have been deposited to NCBI SRA database (BioProject ID: PRJNA801117).
