# Supplementary material for: Single-cell Raman-activated sorting and cultivation (scRACS-Culture) for assessing and mining in situ phosphate-solubilizing microbes from nature
Source: ISME Commun. 2022 Oct 30;2:106. doi: 10.1038/s43705-022-00188-3 (PMC9723661; doi:10.1038/s43705-022-00188-3)
Supplement: Supplementary file 1 — Supplementary information [file 43705_2022_188_MOESM1_ESM.pdf]

Supplemental Information

Supplementary Figures

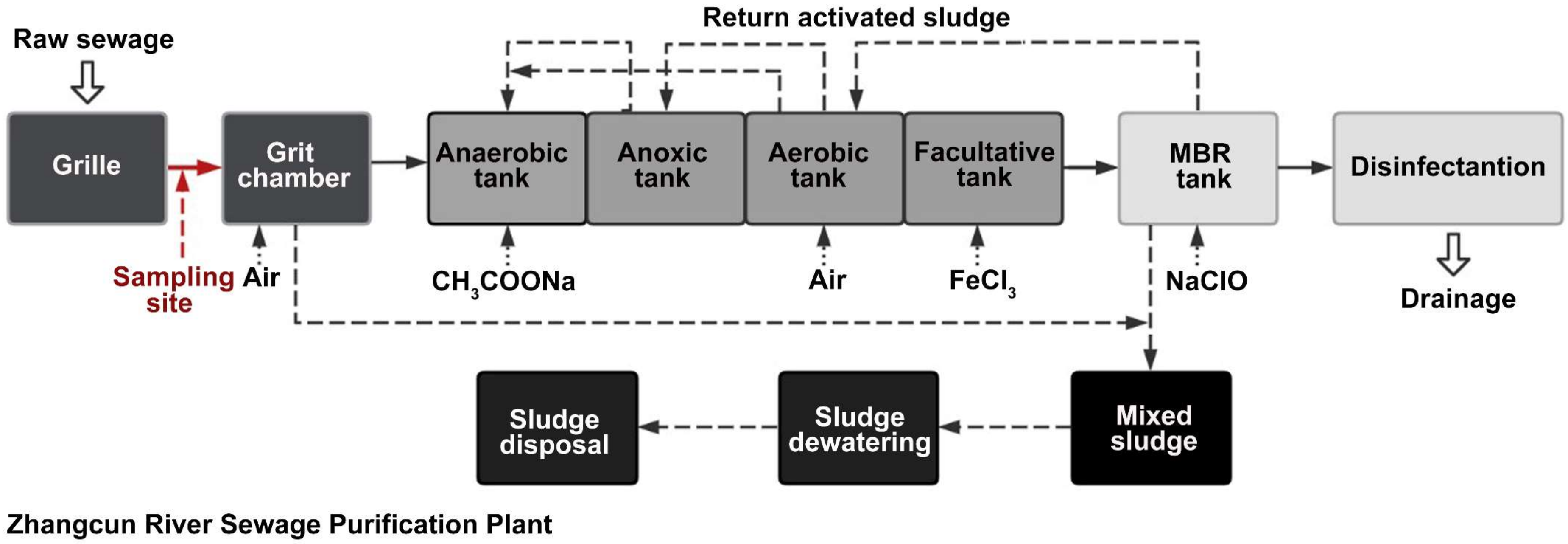

Figure S1. The flow diagram for sewage processing in the Zhangcun River Sewage Purification Plant.

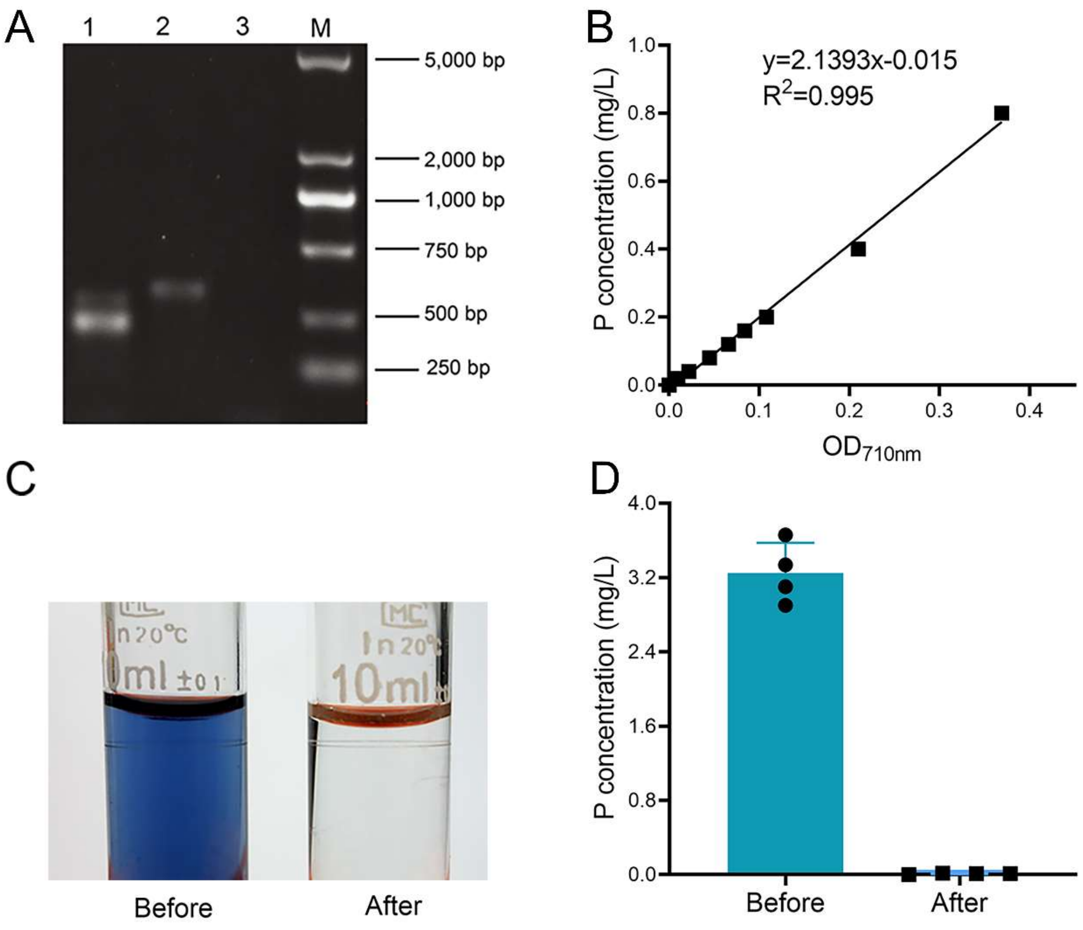

Figure S2. The process of removing phosphorus from sewage to generate the solution that models the *in situ* incubation condition. (**A**) Agarose gel images for the *pqq*C and *pho*X based validation of the sewage-derived organic- and inorganic-PSM. (**B**) Standard curve for the determination of P content in sewage. (**C**) The color reaction of sewage before and after P removal, as determined by the molybdenum-antimony resistance colorimetric method. (**D**) Concentrations of P in sewage as quantified via the molybdenum-antimony resistance colorimetric method before and after the P removal of sewage. Error bars represent the standard deviation of four measurements from biological quadruplicates.

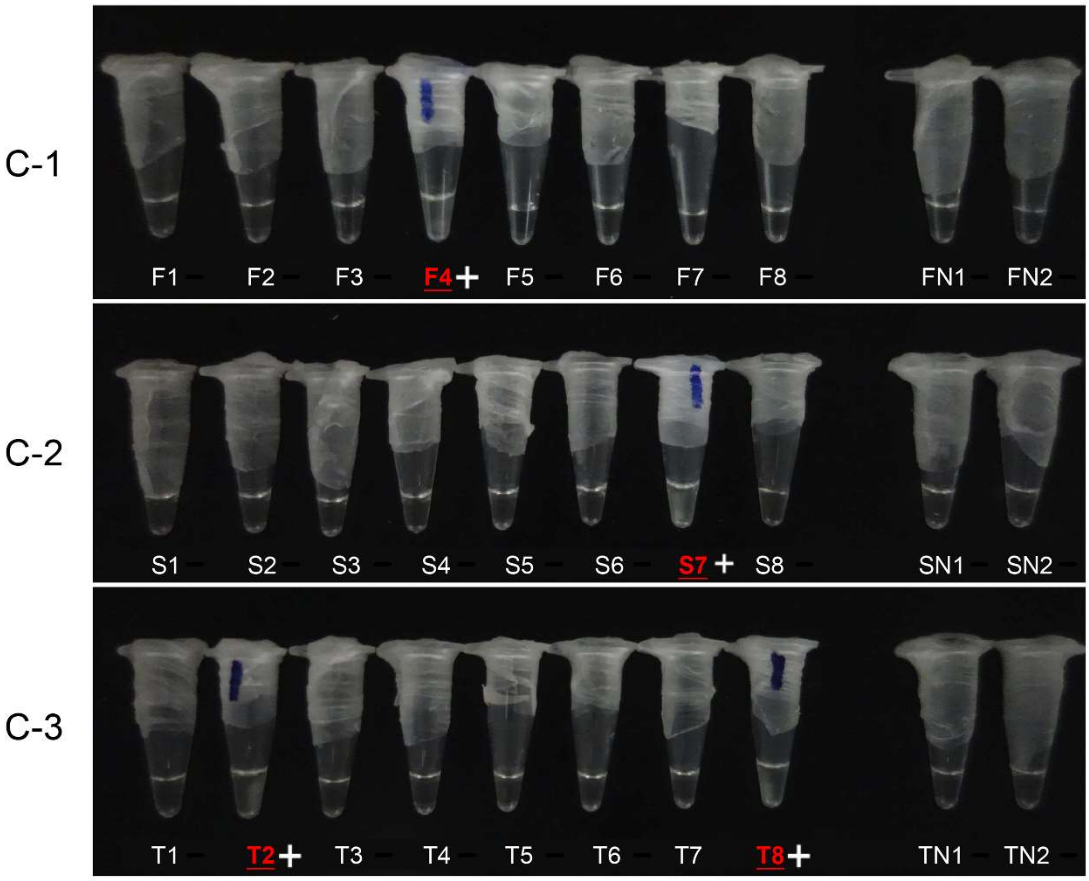

Figure S3. One-cell RACS-Culture of metabolically active organic-PSM cells from sewage. The isolated single organic-PSM cells were cultured respectively in each receiving tube of 20 µL broth medium, in an 'one-cell one-tube' manner. In Tube FN1, FN2, SN1, SN2, TN1 and TN2, empty droplets (i.e., without cells) were sorted and cultured. In Tube F1-F8, S1-S8, T1-T8, the targeted organic-PSM cells were sorted and cultured in a one-cell-one-tube manner.

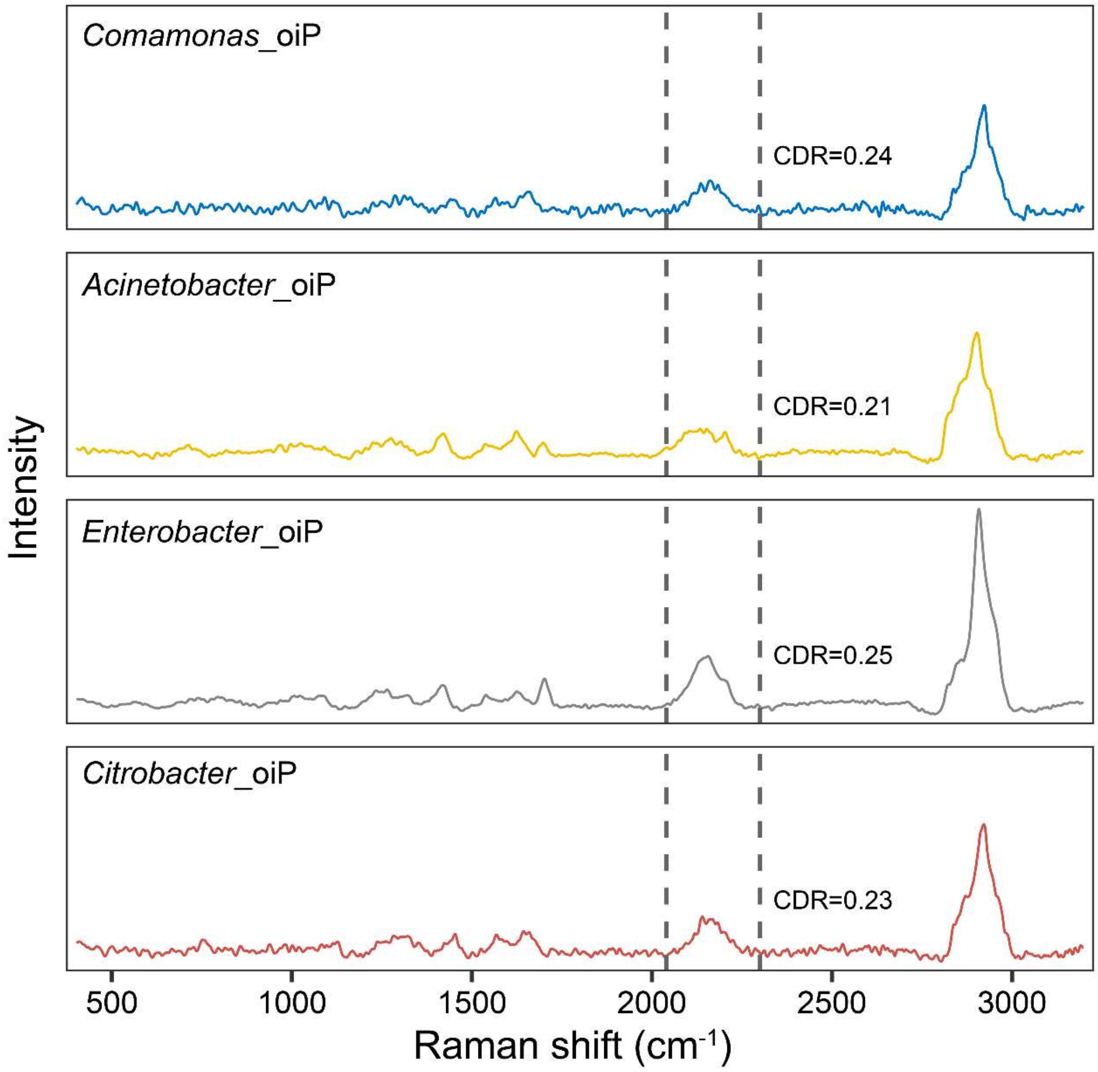

Figure S4. C-D ratios of the scRACS-Culture derived strains of *Comamonas* spp., *Acinetobacter* spp., *Enterobacter* spp. and *Citrobacter* spp.. The cells were incubated in broth media containing 50% D_2O with lecithin (oiP), respectively. Sixty individual cells from each bacteria sample were measured by Raman spectroscopy.

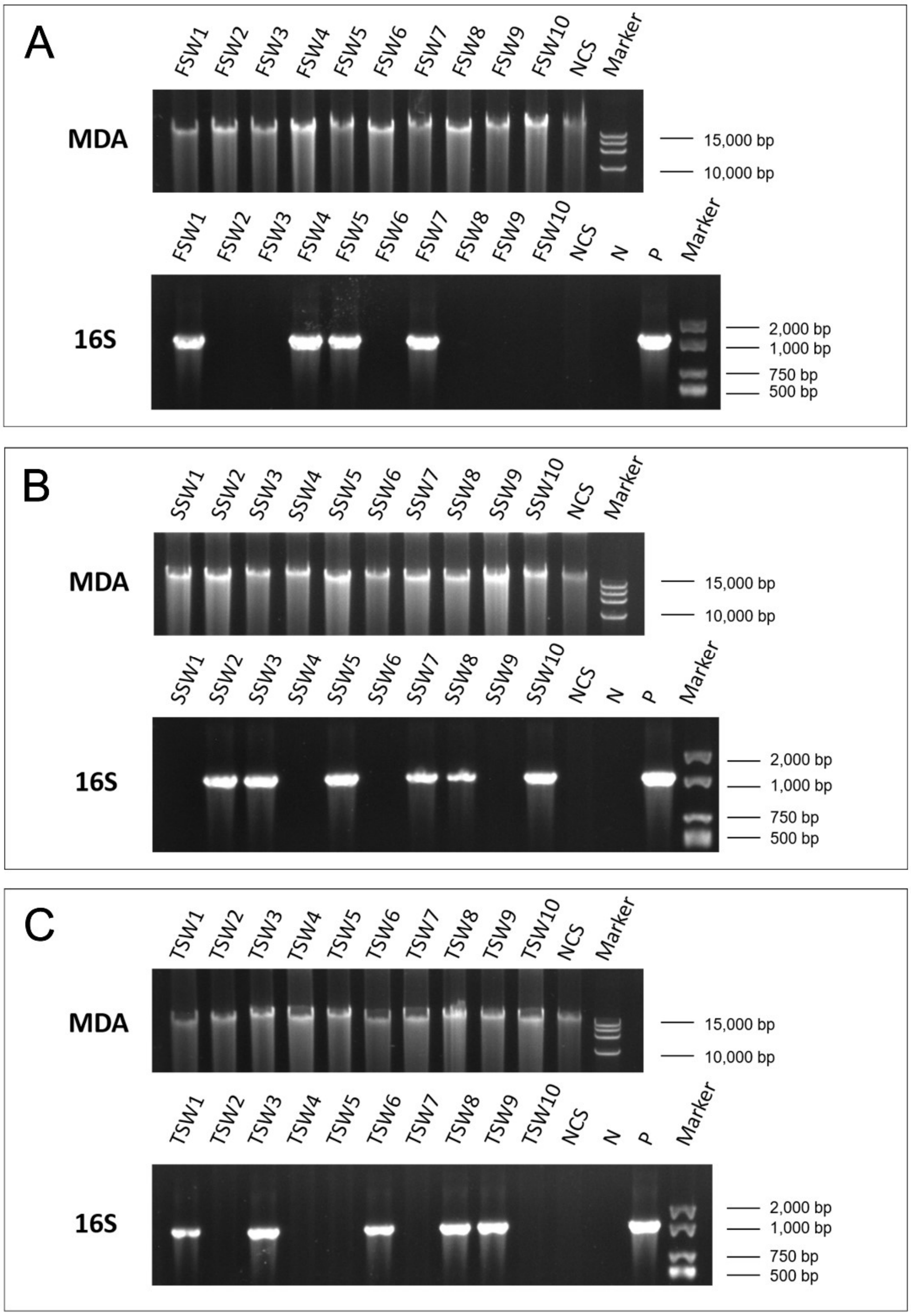

Figure S5. Agarose gel images of the multiple displacement amplifications (MDAs) and 16S rRNA gene validation processes for the organic-PSM obtained from the domestic sewage sample. MDA products and PCR products of the 16S rRNA genes from the first (**A**), second (**B**) and third (**C**) runs of high C-D-peak-marked organic-PSM cells were shown. Lane NCS, empty droplet (i.e., without cells); Lane N, negative control for PCR (i.e., without adding template); Lane P, positive control for PCR.

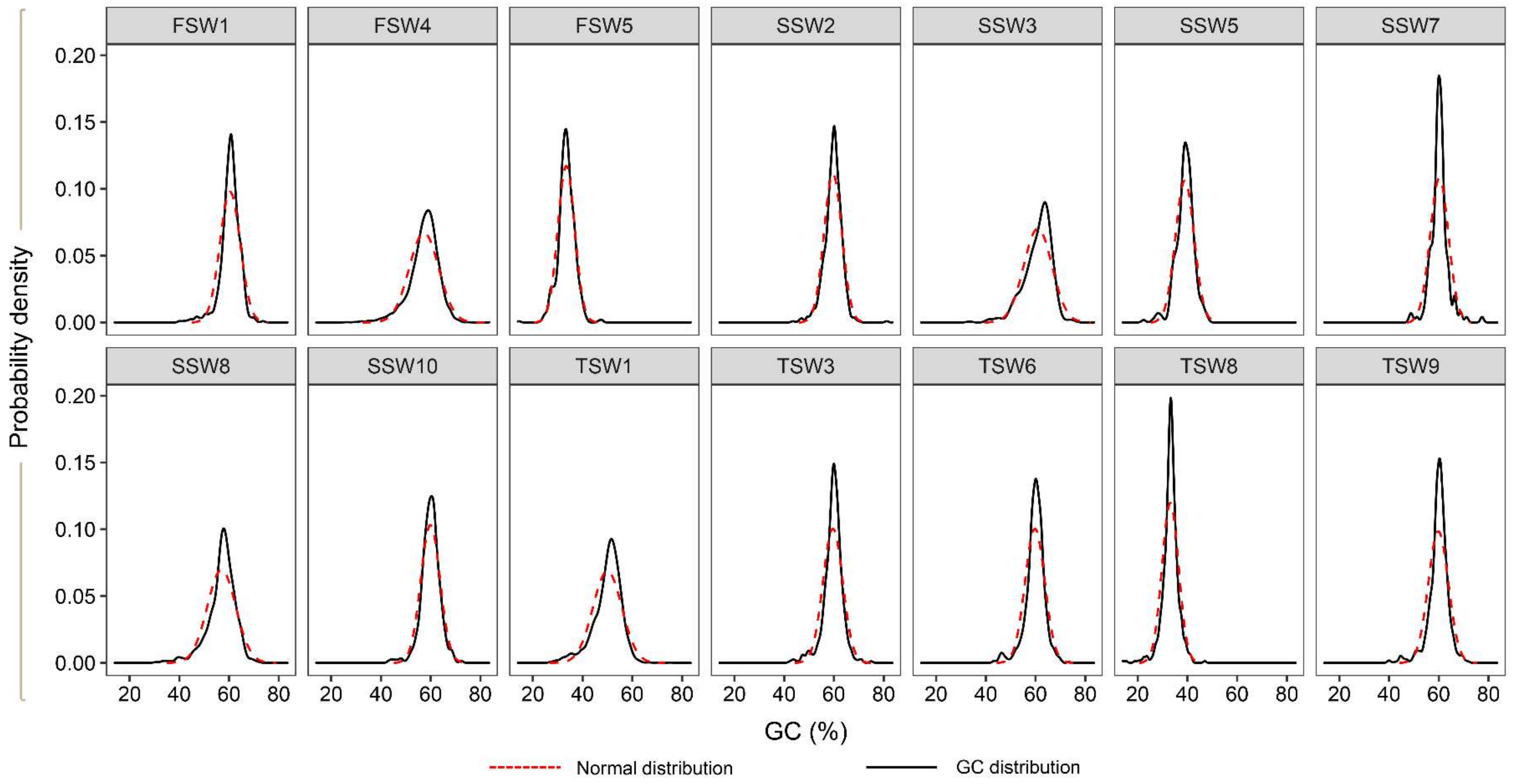

Figure S6. GC distributions of the contigs from the 14 one-cell SAGs that correspond to RAGE-sorted organic-PSMs in sewage. Black curves represent GC distribution of recovered draft genomes. A sliding window of 200 bp along each contig was used to extract sequence fragments and then calculate GC contents. Red curves show theoretical normal distribution with similar mean and standard deviation to the corresponding GC distribution. The GC contents of these sets of contigs exhibit normal distribution, supporting the integrity of the one-cell assemblies.

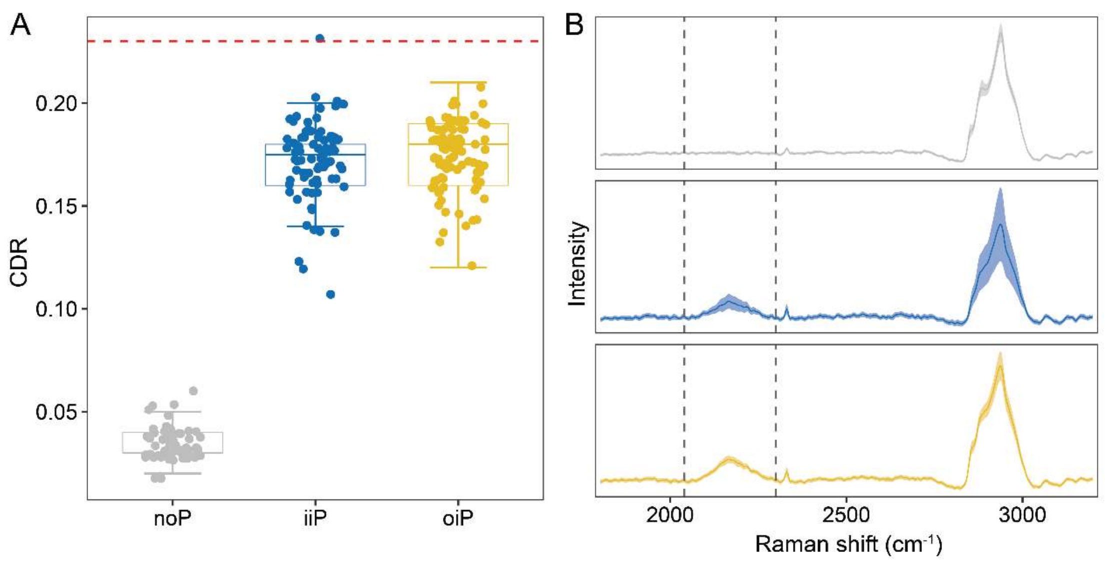

Figure S7. C-D ratios of the commercial strain of *Cutibacterium acnes* BNCC 336443. The strain was incubated in minimum media containing 50% D_2O without P (noP), with $Ca_3(PO_4)_2$ (iiP) and lecithin (oiP), respectively. The CDR distribution patterns (**A**), and the corresponding Raman spectra (**B**) are compared among the group of noP, iiP and oiP. Each spectrum shown represents an average of SCRS from 60 randomly selected cells, and the shadow represents standard deviation of SCRS.

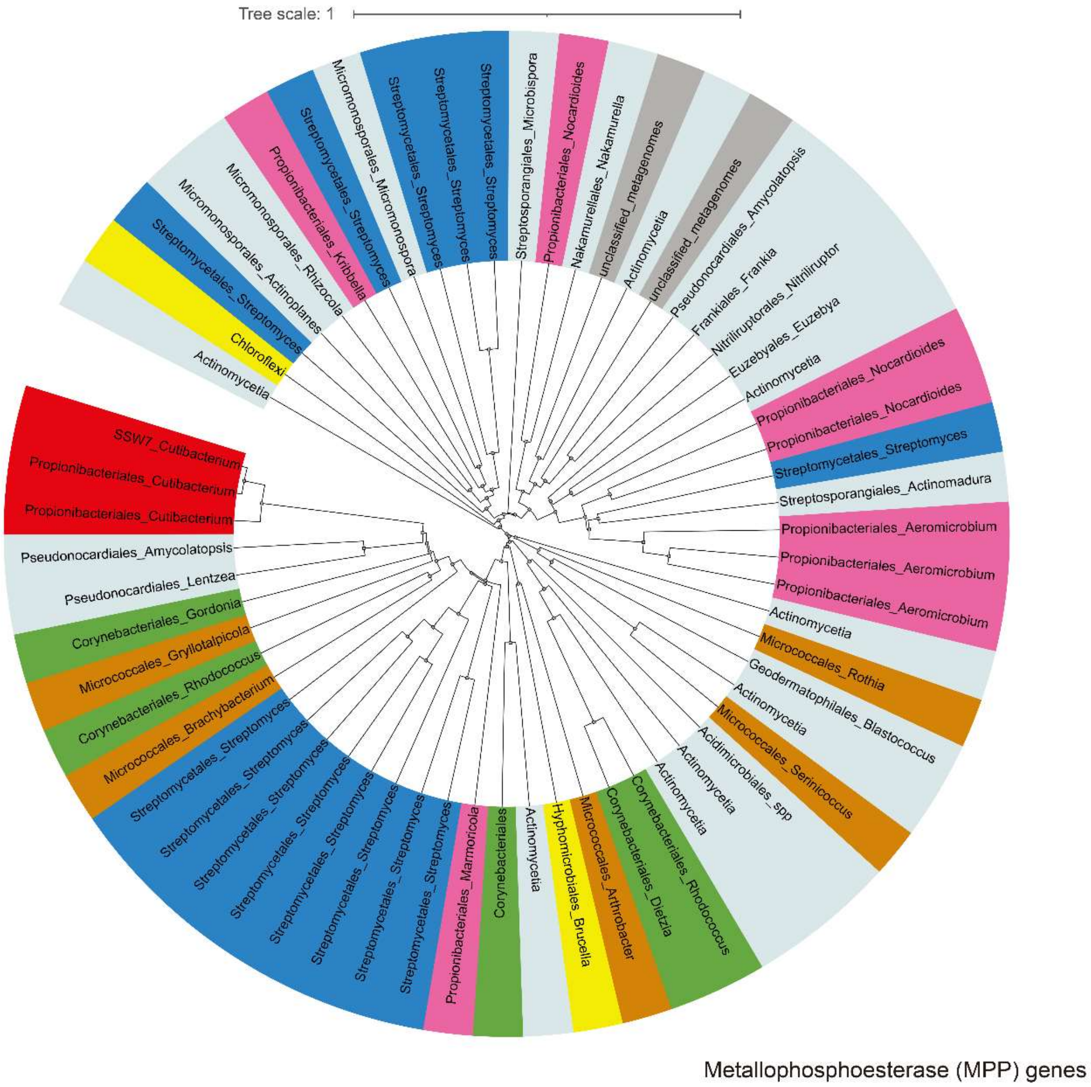

Figure S8. Homology analysis of MPPs which are from the TIGR03767 family.

Supplementary Tables

Table S1. Primers used for PCR amplification of 16S rRNA gene and phosphorus-solubilizing microbiome marker genes.

Description	Name	Primer Sequence (5'→ 3')
**16S rRNA, full length**	27-F	AGAGTTTGATCCTGGCTCAG
	1492-R	GGTTACCTTGTTACGACTT
***pqq*C gene**	*pqq*C-F	AACCGCTTCTACTACCAG
	*pqq*C-R	GCGAACAGCTCGGTCAG
***pho*X gene**	*pho*X-F	GGCAAAACGCCNTGGGGNAC
	*pho*X-R	GGGTCGACCTCGACNAYVYAGCC

Table S2. Taxonomy assignment of the targeted organic-PSM cells based on NCBI/NT or GTDB.

SAGs ID of organic-PBS cells	Class (Genus) assignment based on BLASTN of NCBI/NT	Taxonomical assignment based on GTDB-tk (Database release 95)
**FSW1**	*Cutibacterium*	*Cutibacterium*
**FSW4**	*Klebsiella*	-
**FSW5**	*Cloacibacterium*	*Cloacibacterium*
**SSW2**	*Cutibacterium*	*Cutibacterium*
**SSW3**	*Pseudomonas*	-
**SSW5**	*Acinetobacter*	*Acinetobacter*
**SSW7**	*Cutibacterium*	*Cutibacterium*
**SSW8**	*Cronobacter*	*Cronobacter*
**SSW10**	*Cutibacterium*	*Cutibacterium*
**TSW1**	*Escherichia*	-
**TSW3**	*Cutibacterium*	*Cutibacterium*
**TSW6**	*Cutibacterium*	*Cutibacterium*
**TSW8**	*Cloacibacterium*	*Cloacibacterium*
**TSW9**	*Cutibacterium*	*Cutibacterium*

Table S3. Numbers of 16S rRNA sequencing reads for microbial diversity analysis of sewage sample.

Sample	Raw reads	Processed reads	Pielou's evenness	Observed OTUs	Phylogenetic Diversity	Shanno
**sewage-01**	105862	51159	0.919	753	27.068	8.787
**sewage-02**	127586	61739	0.915	738	27.964	8.721
**sewage-03**	116038	55463	0.926	719	27.189	8.791

Table S4. Statistics of the predicted genes in single-cell genomes of the organic-PSMs in this study.

Sample	Contigs	Bases	Gene	tmRNA	tRNA	CDS	rRNA
**FSW1**	575	2,401,139	2,336	2	45	2,284	5
**FSW4**	5614	11,039,900	10,360	1	57	10,300	2
**FSW5**	644	1,041,361	948	0	18	927	3
**SSW2**	632	1,127,705	1,151	0	21	1,127	3
**SSW3**	570	669,282	695	0	10	681	4
**SSW5**	270	519,948	485	0	9	476	0
**SSW7**	146	2,445,871	2,353	2	45	2,297	9
**SSW8**	766	1,966,581	1,892	1	28	1,860	3
**SSW10**	697	968,806	1,008	0	25	977	6
**TSW1**	2,016	3,099,951	2,929	1	30	2,895	3
**TSW3**	989	1,741,726	1,816	1	35	1,773	7
**TSW6**	970	1,736,898	1,743	1	34	1,702	6
**TSW8**	548	2,075,725	1,925	0	40	1,882	3
**TSW9**	929	2,302,794	2,305	2	45	2,253	5

Table S5. The genes encoding secretary enzymes and cell-wall-anchored phosphoesterases for SSW7. The secretome of the *P. acnes* strain KPA171202 is mapped to the SAG assembly of SSW7. Genes potentially involved in dissolving organic-PSM are highlighted in bold.

Reference gene in KPA171202	Genomic locations in SSW7	Nucleotide similarity	Gene(s) in SSW7	Note
PPA0532	NODE_30:470-1747	100.00%	HCONNHDO_02159	
PPA0533	NODE_30:1783-2352	100.00%	HCONNHDO_02160	
PPA0534	NODE_30:2372-2968	100.00%	HCONNHDO_02161	
PPA0598	NODE_8:45411-43939	99.93%	HCONNHDO_01111	
PPA0644	NODE_110:1661-87	100.00%	HCONNHDO_02301	
PPA0687	NODE_6:21474-20671	99.63%	HCONNHDO_00866	
PPA0721	NODE_6:57368-58525	97.32%	HCONNHDO_00901	
PPA0816	NODE_7:84008-83001	99.90%	HCONNHDO_01046	
PPA1310	NODE_18:38080-39258	99.92%	HCONNHDO_01804	
**PPA1498**	**NODE_9:76869-78623**	**99.87%**	**HCONNHDO_01231**	**metallophosphoesterase**
PPA1662	NODE_32:2307-1430	98.75%	HCONNHDO_02197	
PPA1715	NODE_13:47679-48658	98.27%	HCONNHDO_01531	
PPA1939	NODE_22:30602-31087	99.79%	HCONNHDO_01965	
**PPA2097**	**NODE_2:85112-83003**	**96.07%**	**HCONNHDO_00279***	***ush*A (5'-nucleotidase)**
PPA2105	NODE_2:78785-79804	99.22%	HCONNHDO_00271	
PPA2106	NODE_2:78191-76689	99.00%	HCONNHDO_00270	
PPA2142	NODE_2:36737-35616	99.91%	HCONNHDO_00229*	
PPA2164	NODE_2:12861-14831	99.85%	HCONNHDO_00207	
PPA2175	NODE_2:1245-214	100.00%	HCONNHDO_00196	
PPA2239	NODE_209:610-15	100.00%*	HCONNHDO_02326	
**PPA1745**	**NODE_5: 134806-132251**	**99.92%**	**HCONNHDO_00716**	**phosphoesterase**
**PPA0570**	**NODE_8: 76609-78135**	**99.94%**	**HCONNHDO_01140**	**phosphoesterase**

Supplementary Files

Supplementary file 1. OTU abundance table for the sewage samples (sewage-01, sewage-02 and sewage-03 represent the three biological replicates).

Supplementary file 2. The 16S rRNA genes of the successfully cultured organic-PSM cells derived via scRACS-Culture.

Supplementary file 1: OTU abundance table for the sewage samples (sewage-01, sewage-02 and sewage-03 represent the three biological replicates).

OTU_ID	sewage-01	sewage-02	sewage-03	Abundance	Taxonomy
OTU_1	15856	17608	15692	24.95%	k__Bacteria;p__Campylobacterota;c__Campylobacteria;o__Campylobacterales;f__Arcobacteraceae;g__Arcobacter
OTU_2	4077	1761	3910	4.95%	k__Bacteria;p__Proteobacteria;c__Gammaproteobacteria;o__Burkholderiales;f__Comamonadaceae;g__Hydrogenophaga;s__
OTU_3	2951	1178	1410	2.81%	k__Bacteria;p__Cyanobacteria;c__Cyanobacteriia;o__Chloroplast;f__unidentified_Chloroplast;g__unidentified_Chloroplast
OTU_4	1993	1266	1930	2.63%	k__Bacteria;p__Proteobacteria;c__Gammaproteobacteria;o__Burkholderiales;f__Comamonadaceae;g__Acidovorax;s__Acidovorax_defluvii
OTU_5	619	827	649	1.06%	k__Bacteria;p__Proteobacteria;c__Alphaproteobacteria;o__Rhodobacterales;f__Rhodobacteraceae;g__Pseudorhodobacter;s__
OTU_6	2456	3029	2892	4.25%	k__Bacteria;p__Bacteroidota;c__Bacteroidia;o__Bacteroidales;f__Prevotellaceae;g__Prevotella_9;s__
OTU_7	666	1215	1227	1.58%	k__Bacteria;p__Bacteroidota;c__Bacteroidia;o__Flavobacteriales;f__Weeksellaceae;g__Cloacibacterium;s__
OTU_8	892	996	850	1.39%	k__Bacteria;p__Proteobacteria;c__Gammaproteobacteria;o__Pseudomonadales;f__Moraxellaceae;g__[Agitococcus]_lubricus_group;s__
OTU_9	728	391	798	0.97%	k__Bacteria;p__Proteobacteria;c__Gammaproteobacteria;o__Burkholderiales;f__Aquaspirillaceae;g__Rivicola;s__
OTU_10	2757	1587	2786	3.62%	k__Bacteria;p__Proteobacteria;c__Gammaproteobacteria;o__Pseudomonadales;f__Moraxellaceae;g__Acinetobacter;s__Acinetobacter_johnsonii
OTU_11	1012	867	920	1.42%	k__Bacteria;p__Proteobacteria;c__Gammaproteobacteria;o__Burkholderiales;f__Comamonadaceae;g__Comamonas;s__Comamonas_denitrificans
OTU_12	476	590	511	0.80%	k__Bacteria;p__Firmicutes;c__Bacilli;o__Lactobacillales;f__Carnobacteriaceae;g__Trichococcus;s__
OTU_13	1089	1300	1065	1.75%	k__Bacteria;p__Campylobacterota;c__Campylobacteria;o__Campylobacterales;f__Arcobacteraceae;g__Pseudarcobacter;s__
OTU_14	792	961	897	1.35%	k__Bacteria;p__Proteobacteria;c__Gammaproteobacteria;o__Enterobacterales;f__Aeromonadaceae;g__Aeromonas;s__Aeromonas_caviae
OTU_15	928	877	841	1.34%	k__Bacteria;p__unidentified_Bacteria;c__Gammaproteobacteria;o__Burkholderiales;f__Neisseriaceae;g__unidentified_Neisseriaceae;s__Neisseriaceae_bacterium_DSM_100970
OTU_16	413	186	343	0.48%	k__Bacteria;p__Proteobacteria;c__Gammaproteobacteria;o__Burkholderiales;f__Comamonadaceae
OTU_17	469	252	455	0.60%	k__Bacteria;p__Proteobacteria;c__Gammaproteobacteria;o__Burkholderiales;f__Comamonadaceae;g__Limnohabitans;s__
OTU_18	387	478	378	0.63%	k__Bacteria;p__Firmicutes;c__Clostridia;o__Lachnospirales;f__Lachnospiraceae;g__[Ruminococcus]_gnavus_group;s__
OTU_19	385	561	527	0.75%	k__Bacteria;p__Firmicutes;c__Bacilli;o__Lactobacillales;f__Streptococcaceae;g__Streptococcus
OTU_20	449	227	360	0.53%	k__Bacteria;p__Proteobacteria;c__Gammaproteobacteria;o__Pseudomonadales;f__Pseudomonadaceae;g__Pseudomonas
OTU_21	290	175	288	0.38%	k__Bacteria;p__Proteobacteria;c__Gammaproteobacteria;o__Burkholderiales;f__Comamonadaceae
OTU_22	484	260	507	0.64%	k__Bacteria;p__Fusobacteriota;c__Fusobacteriia;o__Fusobacteriales;f__Leptotrichiaceae;g__Hypnocyclicus;s__
OTU_23	426	460	400	0.65%	k__Bacteria;p__Proteobacteria;c__Gammaproteobacteria;o__Pseudomonadales;f__Moraxellaceae;g__Acinetobacter;s__Acinetobacter_sp_SWBY1
OTU_24	796	450	505	0.89%	k__Bacteria;p__Proteobacteria;c__Gammaproteobacteria;o__Pseudomonadales;f__Moraxellaceae;g__Acinetobacter
OTU_25	284	323	202	0.41%	k__Bacteria;p__Actinobacteria;c__unidentified_Actinobacteria;o__Bifidobacteriales;f__Bifidobacteriaceae;g__Bifidobacterium
OTU_26	288	355	308	0.48%	k__Bacteria;p__Firmicutes;c__Clostridia;o__Lachnospirales;f__Lachnospiraceae;g__[Ruminococcus]_torques_group;s__
OTU_27	275	287	254	0.41%	k__Bacteria;p__Proteobacteria;c__Gammaproteobacteria;o__Pseudomonadales;f__Moraxellaceae;g__Enhydrobacter;s__Moraxella_osloensis
OTU_28	362	346	306	0.51%	k__Bacteria;p__Firmicutes;c__Clostridia;o__Lachnospirales;f__Lachnospiraceae;g__Agathobacter;s__
OTU_29	252	297	283	0.42%	k__Bacteria;p__Bacteroidota;c__Bacteroidia;o__Bacteroidales;f__Bacteroidaceae;g__Bacteroides;s__Bacteroides_coprocola
OTU_30	388	669	506	0.79%	k__Bacteria;p__Firmicutes;c__Clostridia;o__Oscillospirales;f__Ruminococcaceae;g__Faecalibacterium;s__Faecalibacterium_prausnitzii
OTU_31	5	1	4	0.01%	k__Bacteria;p__Proteobacteria;c__Alphaproteobacteria;o__Rhizobiales;f__Rhizobiaceae;g__Bartonella
OTU_32	246	331	297	0.44%	k__Bacteria;p__Bacteroidota;c__Bacteroidia;o__Flavobacteriales;f__Flavobacteriaceae;g__Flavobacterium;s__Flavobacterium_sasangense
OTU_33	168	193	163	0.27%	k__Bacteria;p__Proteobacteria;c__Gammaproteobacteria;o__Enterobacterales;f__Enterobacteriaceae;g__Escherichia-Shigella;s__Escherichia_coli
OTU_34	152	219	189	0.28%	k__Bacteria;p__Bacteroidota;c__Bacteroidia;o__Bacteroidales;f__Paludibacteraceae;g__Paludibacter;s__
OTU_35	169	179	181	0.27%	k__Bacteria;p__Firmicutes;c__Clostridia;o__Lachnospirales;f__Lachnospiraceae;g__Roseburia
OTU_36	375	538	450	0.69%	k__Bacteria;p__Firmicutes;c__Negativicutes;o__Veillonellales-Selenomonadales;f__Selenomonadaceae;g__Megamonas;s__
OTU_37	166	88	157	0.21%	k__Bacteria;p__Proteobacteria;c__Gammaproteobacteria;o__Burkholderiales;f__Comamonadaceae;g__Sphaerotilus;s__Sphaerotilus_natans
OTU_38	188	107	167	0.23%	k__Bacteria;p__Proteobacteria;c__Gammaproteobacteria;o__Pseudomonadales;f__Moraxellaceae;g__Cavicella;s__
OTU_39	0	0	0	0.00%	k__Bacteria;p__Proteobacteria;c__Gammaproteobacteria;o__Enterobacterales;f__Orbaceae;g__Gilliamella
OTU_40	193	171	211	0.29%	k__Bacteria;p__Proteobacteria;c__Gammaproteobacteria;o__Pseudomonadales;f__Moraxellaceae;g__[Agitococcus]_lubricus_group;s__
OTU_41	560	271	363	0.61%	k__Bacteria;p__Proteobacteria;c__Alphaproteobacteria;o__Rickettsiales;f__Mitochondria;g__unidentified_Mitochondria
OTU_42	214	224	227	0.34%	k__Bacteria;p__Bacteroidota;c__Bacteroidia;o__Bacteroidales;f__Bacteroidaceae;g__Bacteroides;s__Bacteroides_plebeius
OTU_43	266	93	247	0.31%	k__Bacteria;p__Proteobacteria;c__Gammaproteobacteria;o__Burkholderiales;f__Comamonadaceae;g__Aquabacterium;s__
OTU_44	135	87	124	0.18%	k__Bacteria;p__Proteobacteria;c__Gammaproteobacteria;o__Enterobacterales;f__Alteromonadaceae;g__Rheinheimera;s__
OTU_45	175	152	163	0.25%	k__Bacteria;p__Campylobacterota;c__Campylobacteria;o__Campylobacterales;f__Sulfurospirillaceae;g__Sulfurospirillum;s__Sulfurospirillum_sp_UCH001
OTU_46	135	155	117	0.21%	k__Bacteria;p__Proteobacteria;c__Gammaproteobacteria;o__Burkholderiales;f__Rhodocyclaceae;g__Dechloromonas;s__
OTU_47	150	104	160	0.21%	k__Bacteria;p__Bacteroidota;c__Bacteroidia;o__Flavobacteriales;f__Flavobacteriaceae;g__Flavobacterium
OTU_48	1	0	0	0.00%	k__Bacteria;p__Proteobacteria;c__Gammaproteobacteria;o__Burkholderiales;f__Neisseriaceae;g__Snodgrassella;s__
OTU_49	212	203	271	0.35%	k__Bacteria;p__Bacteroidota;c__Bacteroidia;o__Bacteroidales;f__Bacteroidaceae;g__Bacteroides;s__Bacteroides_graminisolvens
OTU_50	117	118	101	0.17%	k__Bacteria;p__Firmicutes;c__Negativicutes;o__Veillonellales-Selenomonadales;f__Veillonellaceae;g__Dialister;s__Dialister_sp_Marseille-P5638
OTU_51	292	353	363	0.51%	k__Bacteria;p__Bacteroidota;c__Bacteroidia;o__Bacteroidales;f__Bacteroidaceae;g__Bacteroides;s__Bacteroides_vulgatus
OTU_52	121	101	67	0.15%	k__Bacteria;p__Firmicutes;c__Clostridia;o__Lachnospirales;f__Lachnospiraceae;g__Blautia
OTU_53	0	0	0	0.00%	k__Bacteria;p__Proteobacteria;c__Alphaproteobacteria;o__Acetobacterales;f__Acetobacteraceae;g__Commensalibacter;s__Commensalibacter_sp_AMU001

OTU_54	80	113	82	0.14%	k__Bacteria;p__Bacteroidota;c__Bacteroidia;o__Sphingobacteriales;f__Sphingobacteriaceae;g__Pedobacter;s__
OTU_55	208	206	200	0.31%	k__Bacteria;p__Proteobacteria;c__Gammaproteobacteria;o__Enterobacterales
OTU_56	110	113	129	0.18%	k__Bacteria;p__Bacteroidota;c__Bacteroidia;o__Bacteroidales;f__Paludibacteraceae;g__Paludibacter
OTU_57	108	258	216	0.30%	k__Bacteria;p__Proteobacteria;c__Alphaproteobacteria;o__Sphingomonadales;f__Sphingomonadaceae;g__Sphingomonas;s__Sphingomonas_faeni
OTU_58	73	85	88	0.12%	k__Bacteria;p__Bacteroidota;c__Bacteroidia;o__Bacteroidales;f__Prevotellaceae;g__Prevotella_9;s__Prevotella_paludivivens
OTU_59	111	133	96	0.17%	k__Bacteria;p__Actinobacteria;c__unidentified_Actinobacteria;o__Bifidobacteriales;f__Bifidobacteriaceae;g__Bifidobacterium;s__Bifidobacterium_longum
OTU_60	100	111	110	0.16%	k__Bacteria;p__Firmicutes;c__Bacilli;o__Lactobacillales;f__Enterococcaceae;g__Enterococcus;s__Enterococcus_aquimarinus
OTU_61	171	214	175	0.28%	k__Bacteria;p__Proteobacteria;c__Gammaproteobacteria;o__Enterobacterales;f__Aeromonadaceae;g__Tolumonas;s__
OTU_62	110	104	115	0.17%	k__Bacteria;p__Firmicutes;c__Clostridia;o__Lachnospirales;f__Lachnospiraceae;g__Dorea;s__
OTU_63	86	80	81	0.13%	k__Bacteria;p__Proteobacteria;c__Gammaproteobacteria;o__Burkholderiales;f__Oxalobacteraceae;g__Undibacterium;s__
OTU_64	145	60	140	0.18%	k__Bacteria;p__Proteobacteria;c__Gammaproteobacteria;o__Pseudomonadales;f__Pseudomonadaceae;g__Pseudomonas
OTU_65	379	563	530	0.75%	k__Bacteria;p__Proteobacteria;c__Gammaproteobacteria;o__Burkholderiales;f__Oxalobacteraceae;g__Massilia
OTU_66	82	79	66	0.12%	k__Bacteria;p__Firmicutes;c__Clostridia;o__Oscillospirales;f__Ruminococcaceae;g__Subdoligranulum;s__
OTU_67	134	169	136	0.22%	k__Bacteria;p__Bacteroidota;c__Bacteroidia;o__Bacteroidales;f__Tannerellaceae;g__Macellibacteroides;s__
OTU_68	141	172	160	0.24%	k__Bacteria;p__Firmicutes;c__Clostridia;o__Oscillospirales;f__Ruminococcaceae;g__Ruminococcus;s__Ruminococcus_sp_N15.MGS-57
OTU_69	108	172	98	0.19%	k__Bacteria;p__Proteobacteria;c__Gammaproteobacteria;o__Pseudomonadales;f__Moraxellaceae;g__Psychrobacter;s__Psychrobacter_urativorans
OTU_70	56	64	43	0.08%	k__Bacteria;p__Proteobacteria;c__Alphaproteobacteria;o__Rhodobacterales;f__Rhodobacteraceae
OTU_71	150	174	165	0.25%	k__Bacteria;p__Proteobacteria;c__Gammaproteobacteria;o__Pseudomonadales;f__Pseudomonadaceae;g__Pseudomonas
OTU_72	87	137	134	0.18%	k__Bacteria;p__Bacteroidota;c__Bacteroidia;o__Bacteroidales;f__Bacteroidaceae;g__Bacteroides;s__Bacteroides_stercoris
OTU_73	39	82	59	0.09%	k__Bacteria;p__Proteobacteria;c__Gammaproteobacteria;o__Burkholderiales;f__Oxalobacteraceae;g__Undibacterium
OTU_74	56	77	53	0.09%	k__Bacteria
OTU_75	4	1	7	0.01%	k__Bacteria;p__Actinobacteria;c__unidentified_Actinobacteria;o__Bifidobacteriales;f__Bifidobacteriaceae;g__Bifidobacterium;s__Bifidobacterium_asteroides
OTU_76	107	57	112	0.14%	k__Bacteria;p__Proteobacteria;c__Gammaproteobacteria;o__Pseudomonadales;f__Moraxellaceae;g__[Agitococcus]_lubricus_group;s__
OTU_77	50	71	37	0.08%	k__Bacteria;p__Proteobacteria;c__Gammaproteobacteria;o__Burkholderiales;f__Burkholderiaceae;g__Polynucleobacter;s__Polynucleobacter_asymbioticus
OTU_78	80	59	68	0.11%	k__Bacteria;p__Firmicutes;c__Clostridia;o__Oscillospirales;f__Ruminococcaceae;g__Subdoligranulum;s__
OTU_79	72	71	53	0.10%	k__Bacteria;p__Campylobacterota;c__Campylobacteria;o__Campylobacterales;f__Sulfurimonadaceae;g__Sulfurimonas;s__
OTU_80	0	0	0	0.00%	k__Bacteria;p__Proteobacteria;c__Alphaproteobacteria;o__Acetobacterales;f__Acetobacteraceae;g__Bombella
OTU_81	71	117	110	0.15%	k__Bacteria;p__Bacteroidota;c__Bacteroidia;o__Sphingobacteriales;f__Sphingobacteriaceae;g__Pedobacter
OTU_82	97	80	76	0.13%	k__Bacteria;p__Bacteroidota;c__Bacteroidia;o__Bacteroidales;f__Bacteroidaceae;g__Bacteroides;s__Bacteroides_uniformis
OTU_83	48	65	42	0.08%	k__Bacteria;p__Firmicutes;c__Clostridia;o__Lachnospirales;f__Lachnospiraceae;g__Lachnospira;s__
OTU_84	139	71	123	0.17%	k__Bacteria;p__Proteobacteria;c__Gammaproteobacteria;o__Burkholderiales;f__Comamonadaceae;g__Polaromonas
OTU_85	98	51	94	0.12%	k__Bacteria;p__Proteobacteria;c__Gammaproteobacteria;o__Burkholderiales;f__Comamonadaceae
OTU_86	97	117	71	0.14%	k__Bacteria;p__unidentified_Bacteria;c__Campylobacteria;o__Campylobacterales;f__;g__;s__
OTU_87	44	26	52	0.06%	k__Bacteria;p__Bacteroidota;c__Bacteroidia;o__Flavobacteriales;f__Flavobacteriaceae;g__Flavobacterium
OTU_88	0	0	0	0.00%	k__Bacteria;p__Firmicutes;c__Bacilli;o__Lactobacillales;f__Lactobacillaceae;g__Lactobacillus
OTU_89	41	39	39	0.06%	k__Bacteria;p__Actinobacteriota;c__Coriobacteriia;o__Coriobacteriales;f__Coriobacteriaceae;g__Collinsella;s__Collinsella_aerofaciens
OTU_90	132	85	115	0.17%	k__Bacteria;p__Proteobacteria;c__Gammaproteobacteria;o__Burkholderiales;f__Comamonadaceae;g__Comamonas
OTU_91	33	43	34	0.06%	k__Bacteria;p__Bacteroidota;c__Bacteroidia;o__Bacteroidales;f__Rikenellaceae;g__Alistipes
OTU_92	41	47	27	0.06%	k__Bacteria
OTU_93	0	0	15	0.01%	k__Bacteria;p__Firmicutes;c__Bacilli;o__Erysipelotrichales;f__Erysipelotrichaceae;g__Turicibacter;s__
OTU_94	33	52	42	0.06%	k__Bacteria;p__Firmicutes;c__Clostridia;o__Lachnospirales;f__Lachnospiraceae;g__Tyzzerella;s__
OTU_95	52	43	36	0.07%	k__Bacteria;p__Firmicutes;c__Negativicutes;o__Veillonellales-Selenomonadales;f__Veillonellaceae;g__Veillonella
OTU_96	49	52	39	0.07%	k__Bacteria;p__Firmicutes;c__Clostridia;o__Lachnospirales;f__Lachnospiraceae;g__Lachnoclostridium;s__
OTU_97	110	100	96	0.16%	k__Bacteria;p__Proteobacteria;c__Gammaproteobacteria;o__Pseudomonadales;f__Moraxellaceae;g__Acinetobacter
OTU_98	34	24	36	0.05%	k__Bacteria
OTU_99	44	67	40	0.08%	k__Bacteria;p__Gracilibacteria
OTU_100	51	52	35	0.07%	k__Bacteria;p__Proteobacteria;c__Alphaproteobacteria;o__Rhodobacterales;f__Rhodobacteraceae;g__Rhodobacter;s__
OTU_101	15	22	20	0.03%	k__Bacteria;p__Firmicutes;c__Clostridia;o__Lachnospirales;f__Lachnospiraceae;g__[Eubacterium]_hallii_group;s__
OTU_102	99	55	70	0.11%	k__Bacteria;p__Proteobacteria;c__Gammaproteobacteria;o__Burkholderiales;f__Alcaligenaceae;g__unidentified_Alcaligenaceae;s__Achromobacter_xylosoxidans
OTU_103	76	57	44	0.09%	k__Bacteria;p__Firmicutes;c__Bacilli;o__Erysipelotrichales;f__Erysipelotrichaceae;g__Holdemanella;s__
OTU_104	43	35	20	0.05%	k__Bacteria;p__Firmicutes;c__Clostridia;o__Lachnospirales;f__Lachnospiraceae;g__[Eubacterium]_ventriosum_group;s__
OTU_105	60	80	59	0.10%	k__Bacteria;p__Firmicutes;c__Clostridia;o__Lachnospirales;f__Lachnospiraceae;g__Lachnoclostridium;s__
OTU_106	39	31	61	0.07%	k__Bacteria;p__Fusobacteriota;c__Fusobacteriia;o__Fusobacteriales;f__Fusobacteriaceae;g__Fusobacterium;s__Fusobacterium_mortiferum
OTU_107	42	61	55	0.08%	k__Bacteria;p__unidentified_Bacteria;c__Campylobacteria;o__Campylobacterales;f__;g__;s__
OTU_108	80	89	70	0.12%	k__Bacteria;p__Firmicutes;c__Clostridia;o__Lachnospirales;f__Lachnospiraceae
OTU_109	57	69	45	0.09%	k__Bacteria;p__Proteobacteria;c__Gammaproteobacteria;o__Pseudomonadales;f__Moraxellaceae;g__Acinetobacter

OTU_110	37	48	66	0.08%	k__Bacteria;p__Bacteroidota;c__Bacteroidia;o__Bacteroidales;f__Bacteroidaceae;g__Bacteroides
OTU_111	33	24	33	0.05%	k__Bacteria;p__Proteobacteria;c__Gammaproteobacteria;o__Burkholderiales;f__Rhodocyclaceae;g__Sterolibacterium;s__
OTU_112	37	28	36	0.05%	k__Bacteria;p__Proteobacteria;c__Gammaproteobacteria;o__Burkholderiales;f__Rhodocyclaceae;g__Zoogloea;s__
OTU_113	27	19	35	0.04%	k__Bacteria;p__Proteobacteria;c__Gammaproteobacteria;o__Pseudomonadales;f__Saccharospirillaceae;g__Oceanobacter;s__
OTU_114	22	43	48	0.06%	k__Bacteria;p__Gracilibacteria;c__unidentified_Gracilibacteria;o__JGI_0000069-P22;f__;g__;s__
OTU_115	28	40	35	0.05%	k__Bacteria;p__Proteobacteria;c__Gammaproteobacteria;o__Pseudomonadales;f__Pseudohongiellaceae;g__Pseudohongiella;s__
OTU_116	35	43	32	0.06%	k__Bacteria;p__Firmicutes;c__Clostridia;o__Peptostreptococcales-Tissierellales;f__Peptostreptococcaceae;g__Proteocatella;s__
OTU_117	28	27	44	0.05%	k__Bacteria;p__Firmicutes;c__Negativicutes;o__Acidaminococcales;f__Acidaminococcaceae;g__Phascolarctobacterium;s__Phascolarctobacterium_faecium
OTU_118	0	0	0	0.00%	k__Bacteria;p__Proteobacteria;c__Gammaproteobacteria;o__Enterobacterales;f__Orbaceae;g__Frischella;s__Frischella_perrara
OTU_119	15	25	15	0.03%	k__Bacteria;p__Firmicutes;c__Clostridia;o__Oscillospirales;f__Oscillospiraceae;g__UCG-002;s__
OTU_120	2	4	3	0.00%	k__Bacteria;p__Bacteroidota;c__Bacteroidia;o__Bacteroidales;f__Rikenellaceae;g__Alistipes
OTU_121	35	45	45	0.06%	k__Bacteria;p__Proteobacteria;c__Gammaproteobacteria;o__Burkholderiales;f__Rhodocyclaceae;g__Zoogloea;s__Zoogloea_ramigera
OTU_122	57	27	32	0.06%	k__Bacteria;p__Proteobacteria;c__Gammaproteobacteria;o__Burkholderiales;f__Comamonadaceae
OTU_123	25	40	27	0.05%	k__Bacteria;p__Firmicutes;c__Clostridia;o__Lachnospirales;f__Lachnospiraceae;g__Anaerostipes;s__Anaerostipes_hadrus
OTU_124	94	109	82	0.14%	k__Bacteria;p__Proteobacteria;c__Gammaproteobacteria;o__Enterobacterales;f__Enterobacteriaceae;g__Klebsiella
OTU_125	34	21	38	0.05%	k__Bacteria;p__Proteobacteria;c__Gammaproteobacteria;o__Burkholderiales;f__Aquaspirillaceae;g__Aquaspirillum;s__Aquaspirillum_serpens
OTU_126	41	47	43	0.07%	k__Bacteria;p__Bacteroidota;c__Bacteroidia;o__Flavobacteriales;f__Flavobacteriaceae;g__Flavobacterium
OTU_127	4	28	28	0.03%	k__Bacteria;p__unidentified_Bacteria;c__Campylobacteria;o__Campylobacterales;f__Arcobacteraceae;g__;s__
OTU_128	16	26	31	0.04%	k__Bacteria;p__Firmicutes;c__Bacilli;o__Erysipelotrichales;f__Erysipelatoclostridiaceae;g__Erysipelotrichaceae_UCG-003
OTU_129	0	0	0	0.00%	k__Bacteria;p__Firmicutes;c__Bacilli;o__Lactobacillales;f__Lactobacillaceae;g__Lactobacillus;s__Lactobacillus_apis
OTU_130	24	24	34	0.04%	k__Bacteria
OTU_131	65	43	59	0.08%	k__Bacteria;p__Proteobacteria;c__Gammaproteobacteria;o__Burkholderiales;f__Sutterellaceae;g__Parasutterella;s__
OTU_132	55	61	43	0.08%	k__Bacteria;p__Firmicutes;c__Negativicutes;o__Acidaminococcales;f__Acidaminococcaceae;g__Phascolarctobacterium;s__
OTU_133	36	54	54	0.07%	k__Bacteria;p__Proteobacteria;c__Gammaproteobacteria;o__Pseudomonadales;f__Moraxellaceae;g__Acinetobacter
OTU_134	29	10	44	0.04%	k__Bacteria;p__Proteobacteria;c__Gammaproteobacteria;o__Burkholderiales;f__Oxalobacteraceae
OTU_135	27	29	31	0.04%	k__Bacteria;p__Firmicutes;c__Clostridia;o__Monoglobales;f__Monoglobaceae;g__Monoglobus;s__
OTU_136	44	52	33	0.07%	k__Bacteria;p__unidentified_Bacteria;c__Bacteroidia;o__Bacteroidales;f__Rikenellaceae;g__;s__
OTU_137	21	14	31	0.03%	k__Bacteria
OTU_138	23	38	25	0.04%	k__Bacteria;p__Firmicutes;c__Clostridia;o__Lachnospirales;f__Lachnospiraceae;g__Lachnospiraceae_NK4A136_group;s__
OTU_139	22	30	21	0.04%	k__Bacteria;p__Firmicutes;c__Clostridia;o__Peptostreptococcales-Tissierellales;f__Peptostreptococcaceae;g__Acetoanaerobium;s__
OTU_140	1	0	69	0.04%	k__Bacteria;p__Proteobacteria;c__Gammaproteobacteria;o__Burkholderiales;f__Sutterellaceae;g__Parasutterella;s__
OTU_141	0	0	0	0.00%	k__Bacteria;p__Bacteroidota;c__Bacteroidia;o__Bacteroidales;f__Prevotellaceae;g__Prevotella_7;s__Prevotella_melaninogenica
OTU_142	27	11	23	0.03%	k__Bacteria;p__Proteobacteria;c__Gammaproteobacteria;o__Xanthomonadales;f__Rhodanobacteraceae;g__Ahniella;s__
OTU_143	15	18	24	0.03%	k__Bacteria;p__Proteobacteria;c__Gammaproteobacteria;o__Burkholderiales
OTU_144	25	35	23	0.04%	k__Bacteria;p__Firmicutes;c__Bacilli;o__Lactobacillales;f__Carnobacteriaceae;g__Jeotgalibaca;s__
OTU_145	32	33	34	0.05%	k__Bacteria;p__Bdellovibrionota;c__Bdellovibrionia;o__Bacteriovoracales;f__Bacteriovoracaceae;g__Peredibacter;s__
OTU_146	62	33	44	0.07%	k__Bacteria;p__Proteobacteria;c__Gammaproteobacteria;o__Thiotrichales;f__Thiotrichaceae;g__Thiothrix;s__
OTU_147	1	2	0	0.00%	k__Bacteria;p__Firmicutes;c__Clostridia;o__Lachnospirales;f__Lachnospiraceae;g__Lachnospiraceae_NK4A136_group;s__
OTU_148	30	21	34	0.04%	k__Bacteria;p__Bdellovibrionota;c__Bdellovibrionia;o__Bacteriovoracales;f__Bacteriovoracaceae;g__Bacteriovorax;s__Bacteriovorax_sp_F2
OTU_149	37	18	14	0.04%	k__Bacteria;p__unidentified_Bacteria;c__Clostridia;o__Eubacteriales;f__Eubacteriaceae;g__;s__
OTU_150	0	0	0	0.00%	k__Bacteria;p__Campylobacterota;c__Campylobacteria;o__Campylobacterales;f__Helicobacteraceae;g__Helicobacter
OTU_151	2	0	3	0.00%	k__Bacteria;p__Fusobacteriota;c__Fusobacteriia;o__Fusobacteriales;f__Fusobacteriaceae;g__Fusobacterium;s__Fusobacterium_periodonticum
OTU_152	21	25	23	0.04%	k__Bacteria;p__Gracilibacteria;c__unidentified_Gracilibacteria;o__unidentified_Gracilibacteria;f__unidentified_Gracilibacteria;g__unidentified_Gracilibacteria
OTU_153	23	26	26	0.04%	k__Bacteria;p__Firmicutes;c__Negativicutes;o__Veillonellales-Selenomonadales;f__Sporomusaceae;g__Anaerosinus;s__
OTU_154	23	7	70	0.05%	k__Bacteria;p__Firmicutes;c__Bacilli;o__Lactobacillales;f__Lactobacillaceae;g__Ligilactobacillus;s__Lactobacillus_murinus
OTU_155	17	16	33	0.03%	k__Bacteria;p__Firmicutes;c__Clostridia;o__Oscillospirales;f__[Eubacterium]_coprostanoligenes_group;g__;s__
OTU_156	17	8	26	0.03%	k__Bacteria;p__Proteobacteria;c__Alphaproteobacteria;o__Rickettsiales;f__bac2nit3;g__;s__
OTU_157	51	55	36	0.07%	k__Bacteria;p__Firmicutes;c__Bacilli;o__Lactobacillales;f__Streptococcaceae;g__Lactococcus;s__Lactococcus_lactis
OTU_158	209	232	201	0.33%	k__Bacteria;p__Proteobacteria;c__Gammaproteobacteria;o__Enterobacterales;f__Aeromonadaceae;g__Aeromonas
OTU_159	30	22	10	0.03%	k__Bacteria;p__Firmicutes;c__Clostridia;o__Lachnospirales;f__Lachnospiraceae;g__Coprococcus
OTU_160	0	0	10	0.01%	k__Bacteria;p__Bacteroidota;c__Bacteroidia;o__Bacteroidales;f__Prevotellaceae;g__Prevotellaceae_UCG-001;s__
OTU_161	60	32	30	0.06%	k__Bacteria;p__Proteobacteria;c__Gammaproteobacteria;o__Burkholderiales;f__Burkholderiaceae;g__Ralstonia;s__Ralstonia_pickettii
OTU_162	40	38	43	0.06%	k__Bacteria;p__Campylobacterota;c__Campylobacteria;o__Campylobacterales;f__Sulfurospirillaceae;g__Sulfurospirillum;s__Sulfurospirillum_cavolei
OTU_163	23	39	29	0.05%	k__Bacteria;p__Proteobacteria;c__Gammaproteobacteria;o__Pseudomonadales;f__Spongiibacteraceae;g__;s__
OTU_164	56	72	62	0.10%	k__Bacteria;p__Bacteroidota;c__Bacteroidia;o__Bacteroidales;f__Bacteroidaceae;g__Bacteroides;s__Bacteroides_massiliensis
OTU_165	88	42	94	0.11%	k__Bacteria;p__Proteobacteria;c__Gammaproteobacteria;o__Burkholderiales;f__Comamonadaceae;g__Hydrogenophaga

OTU_166	22	17	39	0.04%	k__Bacteria;p__Proteobacteria;c__Gammaproteobacteria;o__Burkholderiales;f__Rhodocyclaceae;g__Dechlorobacter;s__
OTU_167	15	16	18	0.02%	k__Bacteria;p__Bacteroidota;c__Bacteroidia;o__Bacteroidales;f__Tannerellaceae;g__Parabacteroides;s__Parabacteroides_merdae
OTU_168	19	17	20	0.03%	k__Bacteria;p__Bacteroidota;c__Bacteroidia;o__Chitinophagales;f__Chitinophagaceae;g__Sediminibacterium;s__
OTU_169	21	142	7	0.09%	k__Bacteria;p__Bacteroidota;c__Bacteroidia;o__Cytophagales;f__Hymenobacteraceae;g__Hymenobacter;s__
OTU_170	61	65	79	0.10%	k__Bacteria;p__Bdellovibrionota;c__Bdellovibrionia;o__Bacteriovoracales;f__Bacteriovoracaceae;g__Bacteriovorax;s__
OTU_171	13	4	21	0.02%	k__Bacteria;p__unidentified_Bacteria;c__Fusobacteriia;o__Fusobacteriales;f__Leptotrichiaceae;g__;s__
OTU_172	31	18	34	0.04%	k__Bacteria;p__Proteobacteria;c__Gammaproteobacteria;o__Burkholderiales;f__Methylophilaceae;g__Methylotenera;s__
OTU_173	11	14	13	0.02%	k__Bacteria;p__Proteobacteria;c__Gammaproteobacteria;o__Pseudomonadales;f__Pseudohongiellaceae;g__Pseudohongiella;s__
OTU_174	19	22	19	0.03%	k__Bacteria;p__Firmicutes;c__Negativicutes;o__Acidaminococcales;f__Acidaminococcaceae;g__Acidaminococcus;s__
OTU_175	84	45	68	0.10%	k__Bacteria;p__Proteobacteria;c__Gammaproteobacteria;o__Pseudomonadales;f__Pseudomonadaceae;g__Pseudomonas
OTU_176	4	3	8	0.01%	k__Bacteria;p__Firmicutes;c__unidentified_Firmicutes;o__Oscillospirales;f__Ruminococcaceae;g__unidentified_Ruminococcaceae;s__[Clostridium]_leptum
OTU_177	51	20	64	0.07%	k__Bacteria;p__Proteobacteria;c__Gammaproteobacteria;o__Burkholderiales;f__Comamonadaceae;g__Hydrogenophaga;s__
OTU_178	24	12	3	0.02%	k__Bacteria;p__Verrucomicrobiota;c__Verrucomicrobiae;o__Verrucomicrobiales;f__Akkermansiaceae;g__Akkermansia;s__Akkermansia_muciniphila
OTU_179	27	25	13	0.03%	k__Bacteria;p__Proteobacteria;c__Gammaproteobacteria;o__Burkholderiales;f__Rhodocyclaceae;g__Dechloromonas;s__
OTU_180	32	38	28	0.05%	k__Bacteria;p__Firmicutes;c__Negativicutes;o__Veillonellales-Selenomonadales;f__Veillonellaceae;g__Megasphaera;s__Megasphaera_elsdenii
OTU_181	13	17	13	0.02%	k__Bacteria;p__Firmicutes;c__Clostridia;o__Peptostreptococcales-Tissierellales;f__Peptostreptococcaceae;g__Romboutsia;s__Romboutsia_ilealis
OTU_182	50	32	60	0.07%	k__Bacteria;p__Proteobacteria;c__Gammaproteobacteria;o__Enterobacterales;f__Alteromonadaceae;g__Rheinheimera
OTU_183	33	56	83	0.09%	k__Bacteria;p__Bacteroidota;c__Bacteroidia;o__Bacteroidales;f__Bacteroidaceae;g__Bacteroides;s__Bacteroides_xylanisolvens
OTU_184	17	28	35	0.04%	k__Bacteria;p__Firmicutes;c__Clostridia;o__Lachnospirales;f__Lachnospiraceae;g__Lachnospiraceae_UCG-010;s__
OTU_185	20	8	18	0.02%	k__Bacteria;p__Proteobacteria;c__Gammaproteobacteria;o__Pseudomonadales;f__Moraxellaceae;g__Alkanindiges;s__
OTU_186	30	31	27	0.04%	k__Bacteria;p__Firmicutes;c__Negativicutes;o__Veillonellales-Selenomonadales;f__Veillonellaceae;g__Dialister;s__
OTU_187	26	29	19	0.04%	k__Bacteria;p__Proteobacteria;c__Gammaproteobacteria;o__Pseudomonadales;f__Halomonadaceae;g__Halomonas;s__Candidatus_Halomonas_phosphatis
OTU_188	94	113	55	0.13%	k__Bacteria;p__Actinobacteria;c__unidentified_Actinobacteria;o__Micrococcales;f__Microbacteriaceae;g__Microterricola;s__Microterricola_viridarii
OTU_189	24	27	44	0.05%	k__Bacteria;p__Proteobacteria;c__Gammaproteobacteria;o__Burkholderiales;f__Rhodocyclaceae;g__Propionivibrio;s__
OTU_190	19	16	11	0.02%	k__Bacteria;p__Bacteroidota;c__Bacteroidia;o__Sphingobacteriales;f__Sphingobacteriaceae;g__Solitalea;s__
OTU_191	0	0	0	0.00%	k__Bacteria;p__Bacteroidota;c__Bacteroidia;o__Bacteroidales;f__Marinifilaceae;g__Odoribacter;s__
OTU_192	13	35	24	0.04%	k__Bacteria;p__Proteobacteria;c__Gammaproteobacteria;o__Pseudomonadales;f__Cellvibrionaceae;g__Cellvibrio
OTU_193	19	31	20	0.04%	k__Bacteria;p__Proteobacteria;c__Gammaproteobacteria;o__Burkholderiales;f__T34;g__;s__
OTU_194	25	28	33	0.04%	k__Bacteria;p__Proteobacteria;c__Gammaproteobacteria;o__Pseudomonadales;f__Moraxellaceae;g__Alkanindiges;s__
OTU_195	14	26	15	0.03%	k__Bacteria;p__Fusobacteriota;c__Fusobacteriia;o__Fusobacteriales;f__Leptotrichiaceae;g__Leptotrichia;s__
OTU_196	18	16	8	0.02%	k__Bacteria;p__Proteobacteria;c__Gammaproteobacteria;o__Xanthomonadales;f__Xanthomonadaceae;g__Thermomonas
OTU_197	23	41	29	0.05%	k__Bacteria;p__Firmicutes;c__Clostridia;o__Lachnospirales;f__Lachnospiraceae;g__Blautia
OTU_198	13	28	23	0.03%	k__Bacteria;p__Bacteroidota;c__Bacteroidia;o__Sphingobacteriales;f__Sphingobacteriaceae;g__Pedobacter
OTU_199	40	22	40	0.05%	k__Bacteria;p__Fusobacteriota;c__Fusobacteriia;o__Fusobacteriales;f__Leptotrichiaceae;g__Hypnocyclicus;s__
OTU_200	41	31	33	0.05%	k__Bacteria;p__Proteobacteria;c__Gammaproteobacteria;o__Pseudomonadales;f__Pseudomonadaceae;g__Pseudomonas
OTU_201	9	14	25	0.02%	k__Bacteria;p__Bacteroidota;c__Bacteroidia;o__Bacteroidales;f__Prevotellaceae;g__Prevotella_7;s__
OTU_202	0	0	0	0.00%	k__Bacteria;p__Bacteroidota;c__Bacteroidia;o__Bacteroidales;f__Prevotellaceae;g__Alloprevotella;s__
OTU_203	21	12	19	0.03%	k__Bacteria;p__unidentified_Bacteria;c__Bacteroidia;o__Bacteroidales;f__Paludibacteraceae;g__;s__
OTU_204	11	11	13	0.02%	k__Bacteria;p__Firmicutes;c__Clostridia;o__Oscillospirales;f__Ruminococcaceae;g__Ruminococcus
OTU_205	18	24	19	0.03%	k__Bacteria;p__Proteobacteria;c__Gammaproteobacteria;o__Enterobacterales;f__Aeromonadaceae
OTU_206	15	17	15	0.02%	k__Bacteria;p__Bdellovibrionota;c__Bdellovibrionia;o__Bacteriovoracales;f__Bacteriovoracaceae;g__Peredibacter
OTU_207	25	26	13	0.03%	k__Bacteria;p__Firmicutes;c__Bacilli;o__Lactobacillales;f__Aerococcaceae;g__Aerosphaera;s__
OTU_208	9	16	11	0.02%	k__Bacteria;p__Gracilibacteria;c__unidentified_Gracilibacteria;o__unidentified_Gracilibacteria;f__unidentified_Gracilibacteria;g__unidentified_Gracilibacteria
OTU_209	32	18	56	0.05%	k__Bacteria;p__Proteobacteria;c__Gammaproteobacteria;o__Burkholderiales;f__Neisseriaceae;g__Vitreoscilla;s__
OTU_210	9	14	19	0.02%	k__Bacteria;p__Proteobacteria;c__Gammaproteobacteria;o__Pseudomonadales;f__Pseudohongiellaceae;g__Pseudohongiella;s__
OTU_211	17	30	13	0.03%	k__Bacteria;p__Actinobacteria;c__unidentified_Actinobacteria;o__Micrococcales;f__Intrasporangiaceae;g__Knoellia;s__
OTU_212	42	72	84	0.10%	k__Bacteria;p__Bacteroidota;c__Bacteroidia;o__Bacteroidales;f__Paludibacteraceae;g__Paludibacter;s__
OTU_213	15	35	35	0.04%	k__Bacteria;p__Firmicutes;c__Negativicutes;o__Veillonellales-Selenomonadales;f__Sporomusaceae;g__Anaeromusa-Anaeroarcus;s__
OTU_214	0	0	0	0.00%	k__Bacteria;p__Firmicutes;c__Bacilli;o__Erysipelotrichales;f__Erysipelotrichaceae;g__Dubosiella;s__
OTU_215	11	8	16	0.02%	k__Bacteria;p__Gracilibacteria;c__;o__;f__;g__;s__
OTU_216	12	18	30	0.03%	k__Bacteria;p__Proteobacteria;c__Gammaproteobacteria;o__Thiotrichales;f__Thiotrichaceae;g__Thiothrix
OTU_217	17	17	15	0.02%	k__Bacteria;p__Firmicutes;c__Bacilli;o__Lactobacillales;f__Lactobacillaceae;g__Weissella;s__Weissella_cibaria
OTU_218	22	8	13	0.02%	k__Bacteria;p__Proteobacteria;c__Gammaproteobacteria;o__Burkholderiales;f__Chitinibacteraceae;g__Formivibrio;s__
OTU_219	14	27	27	0.03%	k__Bacteria;p__Firmicutes;c__Clostridia;o__Clostridiales;f__Clostridiaceae;g__Proteiniclasticum;s__
OTU_220	22	33	38	0.05%	k__Bacteria;p__Firmicutes;c__Clostridia;o__Lachnospirales;f__Lachnospiraceae;g__Lachnospira;s__
OTU_221	12	18	12	0.02%	k__Bacteria;p__Bacteroidota;c__Bacteroidia;o__Bacteroidales;f__Bacteroidaceae;g__Bacteroides;s__Bacteroides_paurosaccharolyticus

OTU_222	0	0	0	0.00%	k__Bacteria;p__Actinobacteriota;c__Coriobacteriia;o__Coriobacteriales;f__Atopobiaceae;g__Coriobacteriaceae_UCG-002;s__
OTU_223	0	0	48	0.02%	k__Bacteria;p__Bacteroidota;c__Bacteroidia;o__Bacteroidales;f__Muribaculaceae;g__;s__
OTU_224	10	16	12	0.02%	k__Bacteria;p__Firmicutes;c__Negativicutes;o__Veillonellales-Selenomonadales;f__Selenomonadaceae;g__Propionispira;s__Propionispira_arcuata
OTU_225	11	12	15	0.02%	k__Bacteria;p__Desulfobacterota;c__Desulfovibrionia;o__Desulfovibrionales;f__Desulfovibrionaceae;g__Desulfovibrio;s__Desulfovibrio_desulfuricans
OTU_226	21	11	16	0.02%	k__Bacteria;p__Proteobacteria;c__Alphaproteobacteria;o__Elsterales;f__Elsteraceae;g__Elstera;s__
OTU_227	51	88	42	0.09%	k__Bacteria;p__Proteobacteria;c__Alphaproteobacteria;o__Sphingomonadales;f__Sphingomonadaceae;g__Sphingomonas
OTU_228	22	26	16	0.03%	k__Bacteria;p__Firmicutes;c__Clostridia;o__Christensenellales;f__Christensenellaceae;g__Christensenellaceae_R-7_group;s__
OTU_229	39	51	20	0.06%	k__Bacteria;p__Proteobacteria;c__Alphaproteobacteria;o__Rhizobiales;f__Beijerinckiaceae;g__Methylobacterium-Methylorubrum
OTU_230	32	39	31	0.05%	k__Bacteria;p__Firmicutes;c__Clostridia;o__Lachnospirales;f__Lachnospiraceae;g__[Eubacterium]_eligens_group;s__
OTU_231	3	14	17	0.02%	k__Bacteria;p__Proteobacteria;c__Gammaproteobacteria;o__Burkholderiales;f__Comamonadaceae;g__Brachymonas;s__Brachymonas_denitrificans
OTU_232	16	25	20	0.03%	k__Bacteria;p__Proteobacteria;c__Gammaproteobacteria;o__Pseudomonadales;f__Moraxellaceae;g__Alkanindiges;s__
OTU_233	54	43	43	0.07%	k__Bacteria;p__Proteobacteria;c__Gammaproteobacteria;o__Pseudomonadales;f__Moraxellaceae;g__Acinetobacter
OTU_234	13	12	18	0.02%	k__Bacteria;p__Proteobacteria;c__Gammaproteobacteria;o__Burkholderiales;f__Comamonadaceae
OTU_235	11	17	20	0.02%	k__Bacteria;p__Firmicutes;c__Bacilli;o__Lactobacillales;f__Streptococcaceae;g__Lactococcus
OTU_236	12	20	13	0.02%	k__Bacteria
OTU_237	17	24	14	0.03%	k__Bacteria;p__Firmicutes;c__Bacilli;o__Erysipelotrichales;f__Erysipelotrichaceae;g__Erysipelothrix;s__
OTU_238	0	1	5	0.00%	k__Bacteria;p__Firmicutes;c__Clostridia;o__Lachnospirales;f__Lachnospiraceae;g__Lachnospiraceae_NK4A136_group
OTU_239	86	45	28	0.08%	k__Bacteria;p__Proteobacteria;c__Alphaproteobacteria;o__Rhodobacterales;f__Rhodobacteraceae;g__Paracoccus
OTU_240	18	17	13	0.02%	k__Bacteria;p__Firmicutes;c__Negativicutes;o__Veillonellales-Selenomonadales;f__Selenomonadaceae
OTU_241	1	0	45	0.02%	k__Bacteria;p__Bacteroidota;c__Bacteroidia;o__Bacteroidales;f__Muribaculaceae;g__;s__
OTU_242	12	12	8	0.02%	k__Bacteria;p__Proteobacteria;c__Gammaproteobacteria;o__Pseudomonadales;f__Moraxellaceae;g__Moraxella;s__Moraxella_osloensis
OTU_243	9	16	10	0.02%	k__Bacteria;p__Gracilibacteria;c__;o__;f__;g__;s__
OTU_244	111	68	111	0.15%	k__Bacteria;p__Proteobacteria;c__Gammaproteobacteria;o__Burkholderiales;f__Comamonadaceae
OTU_245	12	9	15	0.02%	k__Bacteria;p__Bacteroidota;c__Bacteroidia;o__Bacteroidales;f__Prevotellaceae;g__Alloprevotella;s__Prevotellaceae_bacterium_Marseille-P2831
OTU_246	11	16	8	0.02%	k__Bacteria;p__Gracilibacteria;c__unidentified_Gracilibacteria;o__unidentified_Gracilibacteria;f__unidentified_Gracilibacteria;g__unidentified_Gracilibacteria
OTU_247	12	3	15	0.02%	k__Bacteria;p__Proteobacteria;c__Gammaproteobacteria;o__Enterobacterales;f__Aeromonadaceae;g__Aeromonas;s__Pseudaeromonas_sharmana
OTU_248	28	29	27	0.04%	k__Bacteria;p__Bacteroidota;c__Bacteroidia;o__Bacteroidales;f__Tannerellaceae;g__Parabacteroides;s__Parabacteroides_sp_CT06
OTU_249	34	35	48	0.06%	k__Bacteria;p__Proteobacteria;c__Gammaproteobacteria;o__Burkholderiales;f__Comamonadaceae
OTU_250	12	47	7	0.03%	k__Bacteria;p__Actinobacteria;c__unidentified_Actinobacteria;o__Kineosporiales;f__Kineosporiaceae;g__Pseudokineococcus
OTU_251	11	14	30	0.03%	k__Bacteria;p__Bacteroidota;c__Bacteroidia;o__Bacteroidales;f__Bacteroidaceae;g__Bacteroides;s__
OTU_252	6	12	13	0.02%	k__Bacteria;p__Proteobacteria;c__Gammaproteobacteria;o__Burkholderiales;f__Sutterellaceae;g__Sutterella;s__
OTU_253	0	0	19	0.01%	k__Bacteria;p__Bacteroidota;c__Bacteroidia;o__Bacteroidales;f__Rikenellaceae;g__Alistipes;s__
OTU_254	15	17	13	0.02%	k__Bacteria;p__Proteobacteria;c__Alphaproteobacteria;o__Caulobacterales;f__Caulobacteraceae;g__Brevundimonas
OTU_255	12	5	15	0.02%	k__Bacteria
OTU_256	0	7	0	0.00%	k__Bacteria;p__Firmicutes;c__Clostridia;o__Oscillospirales;f__Ruminococcaceae;g__[Eubacterium]_siraeum_group;s__
OTU_257	6	12	10	0.01%	k__Bacteria;p__Proteobacteria;c__Gammaproteobacteria;o__Burkholderiales;f__Rhodocyclaceae;g__Dechloromonas;s__
OTU_258	129	67	142	0.17%	k__Bacteria;p__Proteobacteria;c__Gammaproteobacteria;o__Burkholderiales;f__Comamonadaceae;g__Rhodoferax;s__
OTU_259	13	6	26	0.02%	k__Bacteria;p__Bacteroidota;c__Bacteroidia;o__Flavobacteriales;f__Weeksellaceae;g__Chryseobacterium;s__
OTU_260	2	18	25	0.02%	k__Bacteria;p__Firmicutes;c__Bacilli;o__Lactobacillales;f__Lactobacillaceae;g__Lactobacillus;s__Lactobacillus_johnsonii
OTU_261	14	14	13	0.02%	k__Bacteria;p__Proteobacteria;c__Gammaproteobacteria;o__Burkholderiales;f__Sutterellaceae;g__Sutterella;s__Sutterella_wadsworthensis
OTU_262	13	2	9	0.01%	k__Bacteria;p__Proteobacteria;c__Gammaproteobacteria;o__Pseudomonadales;f__Moraxellaceae;g__Fluviicoccus;s__
OTU_263	0	0	0	0.00%	k__Bacteria;p__Bacteroidota;c__Bacteroidia;o__Bacteroidales;f__Muribaculaceae;g__;s__
OTU_264	0	0	0	0.00%	k__Bacteria;p__Bacteroidota;c__Bacteroidia;o__Bacteroidales;f__Prevotellaceae
OTU_265	38	35	54	0.06%	k__Bacteria;p__Proteobacteria;c__Gammaproteobacteria;o__Pseudomonadales;f__Moraxellaceae;g__Acinetobacter;s__
OTU_266	0	0	0	0.00%	k__Bacteria;p__unidentified_Bacteria;c__Clostridia;o__Oscillospirales;f__Oscillospiraceae;g__;s__
OTU_267	4	15	13	0.02%	k__Bacteria;p__Firmicutes;c__Clostridia;o__Lachnospirales;f__Lachnospiraceae;g__Butyrivibrio;s__
OTU_268	71	32	71	0.09%	k__Bacteria;p__Proteobacteria;c__Gammaproteobacteria;o__Burkholderiales;f__Comamonadaceae
OTU_269	1	5	4	0.01%	k__Bacteria;p__Firmicutes;c__Clostridia;o__Christensenellales;f__Christensenellaceae;g__Christensenellaceae_R-7_group;s__
OTU_270	10	14	14	0.02%	k__Bacteria;p__Cyanobacteria;c__Sericytochromatia
OTU_271	0	1	1	0.00%	k__Bacteria
OTU_272	12	8	7	0.01%	k__Bacteria;p__Firmicutes;c__Clostridia;o__Lachnospirales;f__Lachnospiraceae;g__Lachnospiraceae_ND3007_group;s__
OTU_273	12	11	16	0.02%	k__Bacteria;p__Firmicutes;c__Bacilli;o__Lactobacillales;f__Lactobacillaceae;g__Companilactobacillus;s__Lactobacillus_crustorum
OTU_274	10	18	13	0.02%	k__Bacteria;p__unidentified_Bacteria;c__Alphaproteobacteria;o__Paracaedibacterales;f__Paracaedibacteraceae;g__;s__
OTU_275	21	18	5	0.02%	k__Bacteria;p__Firmicutes;c__Clostridia;o__Clostridiales;f__Clostridiaceae;g__Clostridium_sensu_stricto_1;s__
OTU_276	36	36	34	0.05%	k__Bacteria;p__Firmicutes;c__Negativicutes;o__Veillonellales-Selenomonadales;f__Selenomonadaceae;g__Selenomonas;s__Selenomonas_lacticifex
OTU_277	11	21	9	0.02%	k__Bacteria;p__Bacteroidota;c__Bacteroidia;o__Flavobacteriales;f__Flavobacteriaceae;g__Flavobacterium

OTU_278	0	0	0	0.00%	k__Bacteria;p__Bacteroidota;c__Bacteroidia;o__Bacteroidales;f__Muribaculaceae;g__;s__
OTU_279	0	0	9	0.00%	k__Bacteria;p__unidentified_Bacteria;c__Saccharimonadia;o__Saccharimonadales;f__Saccharimonadaceae;g__Candidatus_Saccharimonas;s__
OTU_280	0	0	0	0.00%	k__Bacteria;p__Bacteroidota;c__Bacteroidia;o__Bacteroidales;f__Rikenellaceae;g__Alistipes;s__
OTU_281	0	0	0	0.00%	k__Bacteria;p__Bacteroidota;c__Bacteroidia;o__Bacteroidales;f__Muribaculaceae;g__;s__
OTU_282	24	22	14	0.03%	k__Bacteria;p__Firmicutes;c__Clostridia;o__Lachnospirales;f__Lachnospiraceae;g__Lachnospiraceae_UCG-004;s__
OTU_283	17	21	13	0.03%	k__Bacteria;p__Proteobacteria;c__Gammaproteobacteria;o__Pseudomonadales;f__Halomonadaceae;g__Halomonas;s__Candidatus_Halomonas_phosphatis
OTU_284	9	14	19	0.02%	k__Bacteria;p__Firmicutes;c__Clostridia;o__Oscillospirales;f__Oscillospiraceae;g__NK4A214_group;s__
OTU_285	0	0	0	0.00%	k__Bacteria;p__Firmicutes;c__Clostridia;o__Clostridia_UCG-014;f__;g__;s__
OTU_286	5	5	14	0.01%	k__Bacteria;p__Proteobacteria;c__Gammaproteobacteria;o__Pseudomonadales;f__Pseudomonadaceae;g__Pseudomonas
OTU_287	10	25	11	0.02%	k__Bacteria;p__Firmicutes;c__unidentified_Firmicutes;o__Lachnospirales;f__Lachnospiraceae;g__;s__
OTU_288	7	13	0	0.01%	k__Bacteria;p__Firmicutes;c__Clostridia;o__Oscillospirales;f__[Eubacterium]_coprostanoligenes_group;g__;s__
OTU_289	10	16	12	0.02%	k__Bacteria;p__Firmicutes;c__Clostridia;o__Oscillospirales;f__Butyricicoccaceae;g__Butyricicoccus;s__
OTU_290	25	45	17	0.04%	k__Bacteria;p__Actinobacteria;c__unidentified_Actinobacteria;o__Micrococcales;f__Micrococcaceae;g__Arthrobacter;s__Arthrobacter_agilis
OTU_291	0	0	0	0.00%	k__Bacteria;p__Bacteroidota;c__Bacteroidia;o__Bacteroidales;f__Rikenellaceae;g__Alistipes;s__
OTU_292	7	8	5	0.01%	k__Bacteria;p__Firmicutes;c__Clostridia;o__Oscillospirales;f__Ruminococcaceae;g__Ruminococcus;s__Ruminococcus_bicirculans
OTU_293	6	1	13	0.01%	k__Bacteria;p__unidentified_Bacteria;c__Fusobacteriia;o__Fusobacteriales;f__Leptotrichiaceae;g__;s__
OTU_294	5	5	4	0.01%	k__Bacteria;p__Proteobacteria;c__Gammaproteobacteria;o__Pseudomonadales;f__Moraxellaceae;g__Moraxella
OTU_295	41	18	25	0.04%	k__Bacteria;p__Proteobacteria;c__Gammaproteobacteria;o__Burkholderiales;f__Rhodocyclaceae;g__Thauera;s__
OTU_296	3	4	7	0.01%	k__Bacteria;p__Proteobacteria;c__Gammaproteobacteria;o__Xanthomonadales;f__Rhodanobacteraceae;g__Ahniella;s__
OTU_297	15	3	7	0.01%	k__Bacteria;p__Firmicutes;c__Clostridia;o__Oscillospirales;f__Hungateiclostridiaceae;g__Saccharofermentans;s__
OTU_298	6	7	12	0.01%	k__Bacteria;p__unidentified_Bacteria;c__Bacteroidia;o__Bacteroidales;f__Dysgonomonadaceae;g__;s__
OTU_299	0	0	0	0.00%	k__Bacteria;p__Bacteroidota;c__Bacteroidia;o__Bacteroidales;f__Marinifilaceae;g__Odoribacter;s__
OTU_300	52	9	2	0.03%	k__Bacteria;p__Actinobacteria;c__unidentified_Actinobacteria;o__Kineosporiales;f__Kineosporiaceae;g__Kineococcus;s__
OTU_301	7	16	7	0.02%	k__Bacteria;p__unidentified_Bacteria;c__Negativicutes;o__Veillonellales-Selenomonadales;f__Sporomusaceae;g__;s__
OTU_302	14	15	21	0.03%	k__Bacteria;p__Bacteroidota;c__Bacteroidia;o__Bacteroidales;f__Bacteroidaceae;g__Bacteroides;s__Bacteriodetes_bacterium_enrichment_culture_clone_AP-FeEnrich3
OTU_303	18	4	23	0.02%	k__Bacteria;p__Proteobacteria;c__Gammaproteobacteria;o__Burkholderiales;f__Neisseriaceae;g__Uruburuella
OTU_304	6	9	10	0.01%	k__Bacteria;p__Firmicutes;c__Bacilli;o__Lactobacillales;f__Lactobacillaceae;g__Ligilactobacillus;s__Lactobacillus_salivarius
OTU_305	33	23	4	0.03%	k__Bacteria;p__Proteobacteria;c__Alphaproteobacteria;o__Rhodobacterales;f__Rhodobacteraceae;g__Rubellimicrobium;s__
OTU_306	5	9	15	0.01%	k__Bacteria;p__Firmicutes;c__Clostridia;o__Oscillospirales;f__Ruminococcaceae;g__Ruminococcus;s__
OTU_307	8	5	7	0.01%	k__Bacteria;p__Firmicutes;c__Bacilli;o__Lactobacillales;f__Lactobacillaceae;g__Fructilactobacillus;s__
OTU_308	9	2	9	0.01%	k__Bacteria;p__Proteobacteria;c__Gammaproteobacteria;o__Halothiobacillales;f__Halothiobacillaceae;g__Thiovirga;s__
OTU_309	10	7	20	0.02%	k__Bacteria;p__Bacteroidota;c__Bacteroidia;o__Flavobacteriales;f__Flavobacteriaceae;g__Flavobacterium
OTU_310	13	8	20	0.02%	k__Bacteria;p__Bacteroidota;c__Bacteroidia;o__Bacteroidales;f__Bacteroidaceae;g__Bacteroides;s__Bacteroides_fragilis
OTU_311	7	4	4	0.01%	k__Bacteria;p__unidentified_Bacteria;c__Clostridia;o__Oscillospirales;f__Oscillospiraceae;g__;s__
OTU_312	0	0	0	0.00%	k__Bacteria;p__Proteobacteria;c__Gammaproteobacteria;o__Burkholderiales;f__Neisseriaceae;g__Neisseria
OTU_313	10	8	14	0.02%	k__Bacteria;p__Spirochaetota;c__MVP-15;o__;f__;g__;s__
OTU_314	7	9	16	0.02%	k__Bacteria;p__Gracilibacteria;c__unidentified_Gracilibacteria;o__JGI_0000069-P22;f__;g__;s__
OTU_315	8	15	11	0.02%	k__Bacteria;p__Proteobacteria;c__Gammaproteobacteria;o__Enterobacterales;f__Shewanellaceae;g__Shewanella;s__Shewanella_baltica
OTU_316	0	0	53	0.03%	k__Bacteria;p__Bacteroidota;c__Bacteroidia;o__Bacteroidales;f__Muribaculaceae;g__;s__
OTU_317	5	5	4	0.01%	k__Bacteria;p__Proteobacteria;c__Gammaproteobacteria;o__Enterobacterales;f__Pasteurellaceae;g__Haemophilus;s__Haemophilus_parainfluenzae
OTU_318	22	9	16	0.02%	k__Bacteria;p__Proteobacteria;c__Gammaproteobacteria;o__Burkholderiales;f__Aquaspirillaceae;g__Laribacter;s__Laribacter_hongkongensis
OTU_319	7	4	6	0.01%	k__Bacteria;p__Proteobacteria;c__Gammaproteobacteria;o__Burkholderiales;f__Rhodocyclaceae;g__C39;s__
OTU_320	12	9	14	0.02%	k__Bacteria;p__Firmicutes;c__Bacilli;o__Lactobacillales;f__Lactobacillaceae;g__Lactiplantibacillus;s__Lactobacillus_plantarum
OTU_321	14	21	8	0.02%	k__Bacteria;p__Bacteroidota;c__Bacteroidia;o__Bacteroidales;f__Bacteroidaceae;g__Bacteroides;s__Bacteroides_cellulosilyticus
OTU_322	25	11	13	0.02%	k__Bacteria;p__Proteobacteria;c__Gammaproteobacteria;o__Burkholderiales;f__Rhodocyclaceae;g__Sterolibacterium;s__
OTU_323	0	0	45	0.02%	k__Bacteria;p__Bacteroidota;c__Bacteroidia;o__Bacteroidales;f__Muribaculaceae;g__;s__
OTU_324	61	44	44	0.08%	k__Bacteria;p__Proteobacteria;c__Gammaproteobacteria;o__Enterobacterales;f__Enterobacteriaceae;g__Raoultella;s__Raoultella_ornithinolytica
OTU_325	6	10	4	0.01%	k__Bacteria;p__Bacteroidota;c__Bacteroidia;o__Bacteroidales;f__Paludibacteraceae;g__Paludibacter;s__
OTU_326	9	22	20	0.03%	k__Bacteria;p__Bacteroidota;c__Bacteroidia;o__Flavobacteriales;f__Flavobacteriaceae;g__Flavobacterium;s__Flavobacterium_sp_CSF321-10
OTU_327	17	19	15	0.03%	k__Bacteria;p__Firmicutes;c__Clostridia;o__Lachnospirales;f__Lachnospiraceae;g__Agathobacter;s__Eubacterium_ramulus
OTU_328	10	12	13	0.02%	k__Bacteria;p__Proteobacteria;c__Gammaproteobacteria;o__Burkholderiales;f__Sutterellaceae;g__Sutterella;s__
OTU_329	8	9	17	0.02%	k__Bacteria;p__Bacteroidota;c__Bacteroidia;o__Bacteroidales;f__Bacteroidaceae;g__Bacteroides;s__Bacteroides_coprophilus
OTU_330	11	21	16	0.02%	k__Bacteria;p__Proteobacteria;c__Gammaproteobacteria;o__Enterobacterales;f__Shewanellaceae;g__Shewanella;s__Shewanella_sp_FDAARGOS_354
OTU_331	34	13	56	0.05%	k__Bacteria;p__Proteobacteria;c__Gammaproteobacteria;o__Pseudomonadales;f__Pseudomonadaceae;g__Pseudomonas;s__Pseudomonas_psychrotolerans
OTU_332	6	20	20	0.02%	k__Bacteria;p__Proteobacteria;c__Gammaproteobacteria;o__Burkholderiales;f__Comamonadaceae;g__Alicycliphilus
OTU_333	9	15	21	0.02%	k__Bacteria;p__Proteobacteria;c__Gammaproteobacteria;o__Pseudomonadales;f__Moraxellaceae;g__Acinetobacter;s__Acinetobacter_celticus

OTU_334	15	13	8	0.02%	k__Bacteria;p__Bacteroidota;c__Bacteroidia;o__Bacteroidales;f__Prevotellaceae;g__Prevotellaceae_NK3B31_group
OTU_335	5	10	5	0.01%	k__Bacteria;p__Desulfobacterota;c__Desulfovibrionia;o__Desulfovibrionales;f__Desulfomicrobiaceae;g__Desulfomicrobium;s__
OTU_336	8	1	9	0.01%	k__Bacteria;p__Bacteroidota;c__Bacteroidia;o__Flavobacteriales;f__Weeksellaceae
OTU_337	0	0	0	0.00%	k__Bacteria;p__Firmicutes;c__Clostridia;o__Clostridia_UCG-014;f__;g__;s__
OTU_338	18	15	17	0.03%	k__Bacteria;p__Firmicutes;c__Clostridia;o__Lachnospirales;f__Lachnospiraceae;g__Coprococcus
OTU_339	5	7	8	0.01%	k__Bacteria;p__Firmicutes;c__unidentified_Firmicutes;o__Oscillospirales;f__Ruminococcaceae;g__;s__
OTU_340	0	0	0	0.00%	k__Bacteria;p__Firmicutes;c__Clostridia;o__Lachnospirales;f__Lachnospiraceae;g__Lachnospiraceae_NK4A136_group;s__
OTU_341	10	11	7	0.01%	k__Bacteria;p__Proteobacteria;c__Gammaproteobacteria;o__Burkholderiales;f__Rhodocyclaceae;g__Sulfuritalea;s__
OTU_342	37	46	26	0.06%	k__Bacteria;p__Firmicutes;c__Negativicutes;o__Veillonellales-Selenomonadales;f__Veillonellaceae;g__Dialister;s__
OTU_343	19	7	9	0.02%	k__Bacteria;p__Firmicutes;c__Negativicutes;o__Veillonellales-Selenomonadales;f__Selenomonadaceae;g__Anaerovibrio;s__
OTU_344	13	32	18	0.03%	k__Bacteria;p__Firmicutes;c__Bacilli;o__Lactobacillales;f__Streptococcaceae;g__Streptococcus
OTU_345	7	5	4	0.01%	k__Bacteria
OTU_346	12	10	5	0.01%	k__Bacteria;p__Proteobacteria;c__Gammaproteobacteria;o__Enterobacterales
OTU_347	7	9	8	0.01%	k__Bacteria;p__Firmicutes;c__Bacilli;o__Lactobacillales;f__Lactobacillaceae;g__Loigolactobacillus
OTU_348	0	0	0	0.00%	k__Bacteria;p__Firmicutes;c__Clostridia;o__Lachnospirales;f__Lachnospiraceae;g__Lachnospiraceae_NK4A136_group;s__
OTU_349	5	1	4	0.01%	k__Bacteria;p__Bdellovibrionota;c__Bdellovibrionia;o__Bacteriovoracales;f__Bacteriovoracaceae;g__Peredibacter
OTU_350	0	0	0	0.00%	k__Bacteria;p__Actinobacteriota;c__Coriobacteriia;o__Coriobacteriales;f__Atopobiaceae;g__Atopobium;s__
OTU_351	9	14	15	0.02%	k__Bacteria;p__unidentified_Bacteria;c__Gammaproteobacteria;o__Enterobacterales;f__unidentified_Enterobacterales;g__unidentified_Enterobacterales;s__Idiomarina_sp
OTU_352	5	11	9	0.01%	k__Bacteria;p__Actinobacteria;c__unidentified_Actinobacteria;o__Propionibacteriales;f__Propionibacteriaceae;g__Propioniciclava;s__
OTU_353	10	7	2	0.01%	k__Bacteria;p__Firmicutes;c__Clostridia;o__Lachnospirales;f__Lachnospiraceae;g__[Eubacterium]_ruminantium_group;s__
OTU_354	15	18	18	0.03%	k__Bacteria;p__Campylobacterota;c__Campylobacteria;o__Campylobacterales;f__Sulfurimonadaceae;g__Sulfuricurvum;s__
OTU_355	13	6	8	0.01%	k__Bacteria;p__Firmicutes;c__Clostridia;o__Oscillospirales;f__Oscillospiraceae;g__Flavonifractor;s__
OTU_356	3	10	2	0.01%	k__Bacteria;p__Bacteroidota;c__Bacteroidia
OTU_357	5	10	5	0.01%	k__Bacteria;p__Proteobacteria;c__Gammaproteobacteria;o__Enterobacterales;f__Shewanellaceae;g__Shewanella;s__Shewanella_morhuae
OTU_358	0	10	7	0.01%	k__Bacteria;p__Proteobacteria;c__Gammaproteobacteria;o__Burkholderiales;f__Chitinimonadaceae;g__Chitinivorax;s__
OTU_359	3	6	8	0.01%	k__Bacteria;p__Proteobacteria;c__Gammaproteobacteria;o__Xanthomonadales;f__Xanthomonadaceae;g__Arenimonas;s__
OTU_360	14	12	28	0.03%	k__Bacteria;p__Bacteroidota;c__Bacteroidia;o__Cytophagales;f__Bernardetiaceae;g__Bernardetia;s__
OTU_361	30	51	19	0.05%	k__Bacteria;p__Proteobacteria;c__Alphaproteobacteria;o__Rhizobiales;f__Rhizobiaceae;g__Aureimonas;s__
OTU_362	5	6	1	0.01%	k__Bacteria;p__Proteobacteria;c__Gammaproteobacteria;o__Burkholderiales;f__Aquaspirillaceae;g__Aquaspirillum;s__Aquaspirillum_putridiconchylium
OTU_363	8	9	16	0.02%	k__Bacteria;p__Bacteroidota;c__Bacteroidia;o__Bacteroidales;f__Prevotellaceae;g__Prevotella;s__Prevotella_stercorea
OTU_364	0	1	24	0.01%	k__Bacteria;p__Bacteroidota;c__Bacteroidia;o__Bacteroidales;f__Muribaculaceae;g__;s__
OTU_365	4	9	1	0.01%	k__Bacteria;p__Gracilibacteria;c__unidentified_Gracilibacteria;o__JGI_0000069-P22;f__;g__;s__
OTU_366	8	2	5	0.01%	k__Bacteria;p__Firmicutes;c__Clostridia;o__Oscillospirales;f__[Eubacterium]_coprostanoligenes_group;g__;s__
OTU_367	5	7	8	0.01%	k__Bacteria;p__Bacteroidota;c__Bacteroidia;o__Bacteroidales;f__Bacteroidaceae;g__Bacteroides;s__Bacteroides_salyersiae
OTU_368	45	26	60	0.07%	k__Bacteria;p__Proteobacteria;c__Gammaproteobacteria;o__Pseudomonadales;f__Moraxellaceae;g__Fluviicoccus;s__
OTU_369	0	0	0	0.00%	k__Bacteria;p__Firmicutes;c__Clostridia;o__Peptostreptococcales-Tissierellales;f__Family_XI;g__Parvimonas;s__
OTU_370	8	5	14	0.01%	k__Bacteria;p__Firmicutes;c__Clostridia;o__Peptostreptococcales-Tissierellales;f__Peptostreptococcaceae;g__Proteocatella;s__Frigovirgula_sp_canine_oral_taxon_032
OTU_371	1	12	6	0.01%	k__Bacteria;p__Kapabacteria;c__unidentified_Kapabacteria;o__Kapabacteriales;f__;g__;s__
OTU_372	20	13	5	0.02%	k__Bacteria;p__Proteobacteria;c__Alphaproteobacteria;o__Rhizobiales;f__Beijerinckiaceae;g__Methylobacterium-Methylorubrum;s__Methylorubrum_extorquens
OTU_373	14	3	13	0.02%	k__Bacteria;p__unidentified_Bacteria;c__Fusobacteriia;o__Fusobacteriales;f__Leptotrichiaceae;g__;s__
OTU_374	6	6	5	0.01%	k__Bacteria;p__Firmicutes;c__Bacilli;o__Lactobacillales;f__Lactobacillaceae;g__Limosilactobacillus;s__Lactobacillus_mucosae
OTU_375	6	6	6	0.01%	k__Bacteria;p__Firmicutes;c__Clostridia;o__Oscillospirales;f__Ruminococcaceae;g__Ruminococcus;s__
OTU_376	8	10	15	0.02%	k__Bacteria
OTU_377	0	1	3	0.00%	k__Bacteria;p__Bacteroidota;c__Bacteroidia;o__Bacteroidales;f__Muribaculaceae;g__;s__
OTU_378	1	2	0	0.00%	k__Bacteria;p__Firmicutes;c__Clostridia;o__Lachnospirales;f__Lachnospiraceae
OTU_379	3	4	4	0.01%	k__Bacteria
OTU_380	0	0	0	0.00%	k__Bacteria;p__Bacteroidota;c__Bacteroidia;o__Bacteroidales;f__Muribaculaceae;g__;s__
OTU_381	91	106	77	0.14%	k__Bacteria;p__Firmicutes;c__Clostridia;o__Lachnospirales;f__Lachnospiraceae;g__Blautia;s__
OTU_382	2	5	0	0.00%	k__Bacteria
OTU_383	7	17	10	0.02%	k__Bacteria;p__Firmicutes;c__Clostridia;o__Oscillospirales;f__Oscillospiraceae;g__UCG-005;s__
OTU_384	29	4	3	0.02%	k__Bacteria;p__Firmicutes;c__Bacilli;o__Erysipelotrichales;f__Erysipelotrichaceae;g__Turicibacter
OTU_385	7	2	4	0.01%	k__Bacteria;p__unidentified_Bacteria;c__Alphaproteobacteria;o__Rickettsiales;f__Rickettsiaceae;g__;s__
OTU_386	25	16	20	0.03%	k__Bacteria;p__Proteobacteria;c__Gammaproteobacteria;o__Burkholderiales;f__Comamonadaceae
OTU_387	6	9	12	0.01%	k__Bacteria;p__Proteobacteria;c__Gammaproteobacteria;o__Burkholderiales;f__Rhodocyclaceae;g__Zoogloea;s__
OTU_388	8	2	4	0.01%	k__Bacteria
OTU_389	9	17	6	0.02%	k__Bacteria;p__Firmicutes;c__BRH-c20a;o__;f__;g__;s__

OTU_390	49	21	28	0.05%	k__Bacteria;p__Firmicutes;c__Clostridia;o__Lachnospirales;f__Lachnospiraceae;g__Lachnoclostridium;s__Lachnoclostridium_sp_YL32
OTU_391	0	0	2	0.00%	k__Bacteria;p__Firmicutes;c__Clostridia;o__Monoglobales;f__Monoglobaceae;g__Monoglobus;s__
OTU_392	23	10	13	0.02%	k__Bacteria;p__Proteobacteria;c__Gammaproteobacteria;o__Enterobacterales;f__Alteromonadaceae;g__Rheinheimera;s__
OTU_393	0	0	0	0.00%	k__Bacteria;p__Bacteroidota;c__Bacteroidia;o__Bacteroidales;f__Muribaculaceae;g__;s__
OTU_394	15	15	10	0.02%	k__Bacteria;p__Firmicutes;c__Clostridia;o__Oscillospirales;f__Ruminococcaceae;g__Ruminococcus;s__Ruminococcus_callidus
OTU_395	0	0	0	0.00%	k__Bacteria;p__Campylobacterota;c__Campylobacteria;o__Campylobacterales;f__Campylobacteraceae;g__Campylobacter
OTU_396	9	10	6	0.01%	k__Bacteria;p__Actinobacteria;c__unidentified_Actinobacteria;o__Propionibacteriales;f__Propionibacteriaceae;g__Cutibacterium;s__Cutibacterium_acnes
OTU_397	4	5	6	0.01%	k__Bacteria;p__Proteobacteria;c__Gammaproteobacteria;o__Pseudomonadales;f__Spongiibacteraceae;g__;s__
OTU_398	2	9	8	0.01%	k__Bacteria;p__Firmicutes;c__Clostridia;o__Oscillospirales;f__Oscillospiraceae;g__UCG-003;s__
OTU_399	9	5	8	0.01%	k__Bacteria;p__Firmicutes;c__Negativicutes;o__Veillonellales-Selenomonadales;f__Veillonellaceae;g__Veillonella
OTU_400	7	9	15	0.02%	k__Bacteria
OTU_401	3	0	22	0.01%	k__Bacteria;p__unidentified_Bacteria;c__Desulfovibrionia;o__Desulfovibrionales;f__Desulfovibrionaceae;g__;s__
OTU_402	12	9	14	0.02%	k__Bacteria;p__Firmicutes;c__Negativicutes;o__Acidaminococcales;f__Acidaminococcaceae;g__Succinispira;s__
OTU_403	6	7	4	0.01%	k__Bacteria;p__unidentified_Bacteria;c__Gammaproteobacteria;o__Burkholderiales;f__Alcaligenaceae;g__;s__
OTU_404	0	0	0	0.00%	k__Bacteria;p__Firmicutes;c__Clostridia;o__Clostridia_UCG-014;f__;g__;s__
OTU_405	7	15	7	0.01%	k__Bacteria;p__Bacteroidota;c__Bacteroidia;o__Cytophagales;f__Spirosomaceae;g__Flectobacillus;s__
OTU_406	11	5	11	0.01%	k__Bacteria;p__unidentified_Bacteria;c__Alphaproteobacteria;o__Elsterales;f__Elsteraceae;g__;s__
OTU_407	8	13	14	0.02%	k__Bacteria;p__Bacteroidota;c__Bacteroidia;o__Sphingobacteriales;f__env.OPS_17;g__;s__
OTU_408	0	0	5	0.00%	k__Bacteria;p__Bacteroidota;c__Bacteroidia;o__Bacteroidales;f__Muribaculaceae;g__;s__
OTU_409	0	0	0	0.00%	k__Bacteria;p__Bacteroidota;c__Bacteroidia;o__Bacteroidales;f__Muribaculaceae;g__;s__
OTU_410	3	2	8	0.01%	k__Bacteria;p__Bacteroidota;c__Bacteroidia;o__Flavobacteriales;f__Flavobacteriaceae;g__Flavobacterium;s__Flavobacterium_weaverense
OTU_411	4	9	9	0.01%	k__Bacteria;p__Bacteroidota;c__Bacteroidia;o__Bacteroidales;f__Marinifilaceae;g__Odoribacter;s__Odoribacter_splanchnicus
OTU_412	10	14	4	0.01%	k__Bacteria;p__unidentified_Bacteria;c__Synergistia;o__Synergistales;f__Synergistaceae
OTU_413	7	4	4	0.01%	k__Bacteria;p__Bdellovibrionota;c__Bdellovibrionia;o__Bacteriovoracales;f__Bacteriovoracaceae;g__Peredibacter;s__Bacteriovorax_sp_EPC3
OTU_414	0	0	0	0.00%	k__Bacteria;p__Bacteroidota;c__Bacteroidia;o__Bacteroidales;f__Prevotellaceae;g__Prevotella;s__Prevotella_pallens
OTU_415	3	5	10	0.01%	k__Bacteria;p__Gracilibacteria;c__unidentified_Gracilibacteria;o__Absconditabacteriales_(SR1);f__unidentified_Absconditabacteriales_(SR1);g__unidentified_Absconditabacteriales_(SR1);s__candidate_division_SR1_bacterium_Aalborg_AAW-1
OTU_416	1	0	19	0.01%	k__Bacteria;p__Bacteroidota;c__Bacteroidia;o__Bacteroidales;f__Muribaculaceae;g__;s__
OTU_417	0	0	8	0.00%	k__Bacteria;p__Bacteroidota;c__Bacteroidia;o__Bacteroidales;f__Muribaculaceae;g__;s__
OTU_418	6	2	7	0.01%	k__Bacteria;p__unidentified_Bacteria;c__Gammaproteobacteria;o__Xanthomonadales;f__Rhodanobacteraceae;g__;s__
OTU_419	2	1	3	0.00%	k__Bacteria;p__Actinobacteriota;c__Coriobacteriia;o__Coriobacteriales;f__Eggerthellaceae
OTU_420	8	6	5	0.01%	k__Bacteria;p__Firmicutes;c__Clostridia;o__Clostridiales;f__Clostridiaceae;g__Proteiniclasticum;s__
OTU_421	2	2	5	0.00%	k__Bacteria;p__Proteobacteria;c__Gammaproteobacteria;o__Burkholderiales;f__Rhodocyclaceae;g__Zoogloea
OTU_422	0	2	1	0.00%	k__Bacteria;p__Firmicutes;c__Clostridia;o__Oscillospirales;f__Oscillospiraceae;g__NK4A214_group;s__
OTU_423	4	2	11	0.01%	k__Bacteria;p__Bacteroidota;c__Bacteroidia;o__Bacteroidales;f__Dysgonomonadaceae;g__Petrimonas;s__
OTU_424	6	9	4	0.01%	k__Bacteria;p__Proteobacteria;c__Gammaproteobacteria;o__Enterobacterales;f__Succinivibrionaceae;g__Ruminobacter;s__
OTU_425	7	10	2	0.01%	k__Bacteria;p__Firmicutes;c__Clostridia;o__Oscillospirales;f__Ruminococcaceae;g__Ruminococcus
OTU_426	0	0	46	0.02%	k__Bacteria;p__Bacteroidota;c__Bacteroidia;o__Bacteroidales;f__Prevotellaceae;g__Alloprevotella
OTU_427	13	3	13	0.01%	k__Bacteria;p__Firmicutes;c__Negativicutes;o__Veillonellales-Selenomonadales;f__Veillonellaceae;g__Dialister;s__
OTU_428	9	3	9	0.01%	k__Bacteria;p__Proteobacteria;c__Gammaproteobacteria;o__Pseudomonadales;f__Moraxellaceae;g__Acinetobacter
OTU_429	23	3	4	0.02%	k__Bacteria;p__Firmicutes;c__Bacilli;o__Lactobacillales;f__Vagococcaceae;g__Vagococcus;s__Vagococcus_fluvialis
OTU_430	5	6	3	0.01%	k__Bacteria;p__Gracilibacteria;c__unidentified_Gracilibacteria;o__JGI_0000069-P22;f__;g__;s__
OTU_431	1	10	10	0.01%	k__Bacteria;p__Firmicutes;c__Bacilli;o__Lactobacillales;f__Lactobacillaceae;g__Lactobacillus
OTU_432	16	12	19	0.02%	k__Bacteria;p__Bacteroidota;c__Bacteroidia;o__Bacteroidales;f__Marinilabiliaceae;g__[Cytophaga]_xylanolytica_group;s__Cytophaga_xylanolytica
OTU_433	0	4	9	0.01%	k__Bacteria;p__Bacteroidota;c__Bacteroidia;o__Bacteroidales;f__Rikenellaceae;g__dgA-11_gut_group;s__
OTU_434	4	3	4	0.01%	k__Bacteria;p__unidentified_Bacteria;c__Bacteroidia;o__Bacteroidales;f__;g__;s__
OTU_435	4	1	2	0.00%	k__Bacteria;p__Firmicutes;c__Bacilli;o__Lactobacillales;f__Streptococcaceae;g__Lactococcus;s__Lactococcus_garvieae
OTU_436	3	4	7	0.01%	k__Bacteria;p__Proteobacteria;c__Gammaproteobacteria;o__Pseudomonadales;f__Moraxellaceae;g__Moraxella
OTU_437	16	8	0	0.01%	k__Bacteria;p__Proteobacteria;c__Gammaproteobacteria
OTU_438	11	16	8	0.02%	k__Bacteria;p__Firmicutes;c__Clostridia;o__Clostridia_UCG-014;f__;g__;s__
OTU_439	6	2	0	0.00%	k__Bacteria;p__Bacteroidota;c__Bacteroidia;o__Bacteroidales;f__Prevotellaceae;g__Prevotella_7;s__
OTU_440	47	11	15	0.04%	k__Bacteria;p__Proteobacteria;c__Alphaproteobacteria;o__Rhizobiales;f__Rhizobiaceae;g__Allorhizobium-Neorhizobium-Pararhizobium-Rhizobium
OTU_441	8	11	5	0.01%	k__Bacteria;p__unidentified_Bacteria;c__Clostridia;o__Oscillospirales;f__Oscillospiraceae;g__;s__
OTU_442	5	6	5	0.01%	k__Bacteria;p__unidentified_Bacteria;c__Fusobacteriia;o__Fusobacteriales;f__Leptotrichiaceae;g__;s__
OTU_443	9	2	7	0.01%	k__Bacteria;p__Proteobacteria;c__Gammaproteobacteria;o__Burkholderiales;f__Comamonadaceae;g__Comamonas;s__

OTU_444	6	8	16	0.02%	k__Bacteria;p__Firmicutes;c__Negativicutes;o__Acidaminococcales;f__Acidaminococcaceae;g__Succiniclasticum;s__
OTU_445	4	2	3	0.00%	k__Bacteria;p__Proteobacteria;c__Gammaproteobacteria;o__Pseudomonadales;f__Moraxellaceae;g__Alkanindiges;s__
OTU_446	2	8	3	0.01%	k__Bacteria;p__Bdellovibrionota;c__Bdellovibrionia;o__Bdellovibrionales;f__Bdellovibrionaceae;g__Bdellovibrio;s__Bdellovibrio_bacteriovorus
OTU_447	12	0	5	0.01%	k__Bacteria;p__Firmicutes;c__Clostridia;o__Peptostreptococcales-Tissierellales;f__Peptostreptococcaceae;g__Peptoclostridium;s__
OTU_448	0	0	26	0.01%	k__Bacteria;p__Bacteroidota;c__Bacteroidia;o__Bacteroidales;f__Muribaculaceae;g__;s__
OTU_449	10	4	7	0.01%	k__Bacteria;p__Firmicutes;c__Bacilli;o__Lactobacillales;f__Lactobacillaceae;g__Ligilactobacillus;s__Lactobacillus_ruminis
OTU_450	23	17	4	0.02%	k__Bacteria;p__Proteobacteria;c__Gammaproteobacteria;o__Enterobacterales;f__Vibrionaceae;g__Vibrio
OTU_451	138	134	167	0.22%	k__Bacteria;p__Proteobacteria;c__Gammaproteobacteria;o__Enterobacterales
OTU_452	10	25	9	0.02%	k__Bacteria;p__Firmicutes;c__Clostridia;o__Clostridiales;f__Clostridiaceae;g__Clostridium_sensu_stricto_1;s__Clostridium_beijerinckii
OTU_453	1	7	7	0.01%	k__Bacteria;p__Kapabacteria;c__unidentified_Kapabacteria;o__Kapabacteriales
OTU_454	3	11	6	0.01%	k__Bacteria;p__Firmicutes;c__Clostridia;o__Peptostreptococcales-Tissierellales;f__Anaerovoracaceae;g__Anaerovorax
OTU_455	7	6	6	0.01%	k__Bacteria;p__unidentified_Bacteria;c__Negativicutes;o__Acidaminococcales;f__Acidaminococcaceae;g__;s__
OTU_456	2	11	3	0.01%	k__Bacteria;p__Bacteroidota;c__Bacteroidia;o__Bacteroidales;f__Prolixibacteraceae;g__Roseimarinus;s__
OTU_457	0	0	2	0.00%	k__Bacteria;p__Desulfobacterota;c__Desulfovibrionia;o__Desulfovibrionales;f__Desulfovibrionaceae;g__Desulfovibrio;s__
OTU_458	3	6	6	0.01%	k__Bacteria;p__Firmicutes;c__Clostridia;o__Clostridia_UCG-014;f__;g__;s__
OTU_459	15	8	10	0.02%	k__Bacteria;p__Firmicutes;c__Clostridia;o__Oscillospirales;f__Oscillospiraceae;g__UCG-002;s__
OTU_460	8	9	7	0.01%	k__Bacteria;p__Proteobacteria;c__Gammaproteobacteria;o__Pseudomonadales;f__Moraxellaceae;g__Acinetobacter
OTU_461	2	5	4	0.01%	k__Bacteria;p__Bdellovibrionota;c__Bdellovibrionia;o__Bacteriovoracales;f__Bacteriovoracaceae;g__Peredibacter;s__
OTU_462	3	10	9	0.01%	k__Bacteria;p__Proteobacteria;c__Gammaproteobacteria;o__Burkholderiales;f__Sutterellaceae;g__Sutterella;s__
OTU_463	15	11	5	0.02%	k__Bacteria;p__Actinobacteria;c__unidentified_Actinobacteria;o__Frankiales;f__Geodermatophilaceae;g__Klenkia;s__
OTU_464	2	2	6	0.01%	k__Bacteria
OTU_465	15	8	5	0.01%	k__Bacteria;p__Bacteroidota;c__Bacteroidia;o__Flavobacteriales;f__Weeksellaceae;g__Chryseobacterium
OTU_466	0	0	0	0.00%	k__Bacteria;p__Fusobacteriota;c__Fusobacteriia;o__Fusobacteriales;f__Leptotrichiaceae;g__Leptotrichia
OTU_467	53	12	16	0.04%	k__Bacteria;p__Proteobacteria;c__Gammaproteobacteria;o__Enterobacterales;f__Erwiniaceae;g__Pantoea
OTU_468	3	3	3	0.00%	k__Bacteria;p__Bacteroidota;c__Bacteroidia;o__Bacteroidales;f__Prevotellaceae;g__Prevotella_7;s__
OTU_469	5	10	8	0.01%	k__Bacteria;p__Proteobacteria;c__Gammaproteobacteria;o__Pseudomonadales;f__Moraxellaceae;g__Psychrobacter
OTU_470	4	17	36	0.03%	k__Bacteria;p__Firmicutes;c__Bacilli;o__Lactobacillales;f__Lactobacillaceae;g__Limosilactobacillus;s__Lactobacillus_reuteri
OTU_471	4	0	0	0.00%	k__Bacteria;p__unidentified_Bacteria;c__Gammaproteobacteria;o__Salinisphaerales;f__Solimonadaceae;g__;s__
OTU_472	16	15	13	0.02%	k__Bacteria;p__unidentified_Bacteria;c__Campylobacteria;o__Campylobacterales;f__Arcobacteraceae;g__;s__
OTU_473	0	0	0	0.00%	k__Bacteria;p__Bacteroidota;c__Bacteroidia;o__Bacteroidales;f__Muribaculaceae;g__;s__
OTU_474	8	2	7	0.01%	k__Bacteria;p__Cyanobacteria;c__Sericytochromatia;o__;f__;g__;s__
OTU_475	0	0	16	0.01%	k__Bacteria;p__Bacteroidota;c__Bacteroidia;o__Bacteroidales;f__Muribaculaceae;g__;s__
OTU_476	13	5	2	0.01%	k__Bacteria;p__Proteobacteria;c__Alphaproteobacteria;o__Sphingomonadales;f__Sphingomonadaceae;g__Altererythrobacter;s__Porphyrobacter_mercurialis
OTU_477	3	3	4	0.01%	k__Bacteria;p__Proteobacteria;c__Alphaproteobacteria;o__Rhizobiales;f__Devosiaceae;g__Devosia;s__
OTU_478	53	39	35	0.06%	k__Bacteria;p__Proteobacteria;c__Gammaproteobacteria;o__Burkholderiales;f__Comamonadaceae;g__Simplicispira;s__
OTU_479	4	4	8	0.01%	k__Bacteria;p__Synergistota;c__Synergistia;o__Synergistales;f__Synergistaceae;g__Cloacibacillus;s__
OTU_480	2	6	5	0.01%	k__Bacteria;p__Actinobacteriota;c__Coriobacteriia;o__Coriobacteriales;f__Atopobiaceae;g__Olsenella;s__
OTU_481	11	19	7	0.02%	k__Bacteria
OTU_482	3	4	2	0.00%	k__Bacteria;p__Bacteroidota;c__Bacteroidia;o__Bacteroidales;f__Prolixibacteraceae;g__Roseimarinus;s__
OTU_483	6	5	5	0.01%	k__Bacteria;p__Desulfobacterota;c__Desulfuromonadia;o__;f__;g__;s__
OTU_484	1	5	6	0.01%	k__Bacteria;p__Bacteroidota;c__Bacteroidia;o__Bacteroidales;f__Dysgonomonadaceae;g__Proteiniphilum;s__
OTU_485	7	7	10	0.01%	k__Bacteria;p__Kapabacteria;c__unidentified_Kapabacteria;o__Kapabacteriales;f__;g__;s__
OTU_486	0	0	36	0.02%	k__Bacteria;p__Bacteroidota;c__Bacteroidia;o__Bacteroidales;f__Muribaculaceae;g__;s__
OTU_487	0	0	0	0.00%	k__Bacteria;p__Firmicutes;c__Bacilli;o__Erysipelotrichales;f__Erysipelatoclostridiaceae;g__Erysipelatoclostridium;s__
OTU_488	6	11	4	0.01%	k__Bacteria;p__Firmicutes;c__Negativicutes;o__Veillonellales-Selenomonadales;f__Veillonellaceae;g__Megasphaera;s__
OTU_489	16	13	7	0.02%	k__Bacteria;p__Firmicutes;c__Clostridia;o__Oscillospirales;f__Oscillospiraceae;g__NK4A214_group;s__
OTU_490	1	1	2	0.00%	k__Bacteria;p__Firmicutes;c__Clostridia;o__Lachnospirales;f__Lachnospiraceae;g__Lachnospiraceae_NK4A136_group;s__
OTU_491	0	0	0	0.00%	k__Bacteria;p__Actinobacteriota;c__Coriobacteriia;o__Coriobacteriales;f__Eggerthellaceae
OTU_492	4	2	3	0.00%	k__Bacteria;p__Proteobacteria;c__Gammaproteobacteria;o__Burkholderiales;f__Chitinibacteraceae;g__Formivibrio;s__
OTU_493	10	7	9	0.01%	k__Bacteria;p__Bacteroidota;c__Bacteroidia;o__Bacteroidales;f__Rikenellaceae;g__Alistipes;s__
OTU_494	49	61	49	0.08%	k__Bacteria;p__Firmicutes;c__Bacilli;o__Lactobacillales;f__Enterococcaceae;g__Enterococcus;s__Enterococcus_faecalis
OTU_495	13	17	10	0.02%	k__Bacteria;p__Firmicutes;c__Bacilli;o__Entomoplasmatales;f__unidentified_Entomoplasmatales;g__Candidatus_Spiroplasma;s__
OTU_496	1	3	7	0.01%	k__Bacteria;p__Firmicutes;c__Clostridia;o__Lachnospirales;f__Lachnospiraceae
OTU_497	7	24	24	0.03%	k__Bacteria;p__Desulfobacterota;c__Desulfovibrionia;o__Desulfovibrionales;f__Desulfomicrobiaceae;g__Desulfomicrobium
OTU_498	0	0	24	0.01%	k__Bacteria;p__Bacteroidota;c__Bacteroidia;o__Bacteroidales;f__Muribaculaceae;g__;s__
OTU_499	6	10	7	0.01%	k__Bacteria;p__Firmicutes;c__Bacilli;o__Staphylococcales;f__Staphylococcaceae;g__Staphylococcus;s__Staphylococcus_aureus

OTU_500	3	1	5	0.00%	k__Bacteria;p__Bacteroidota;c__Bacteroidia;o__Bacteroidales;f__Dysgonomonadaceae
OTU_501	6	8	0	0.01%	k__Bacteria;p__Proteobacteria;c__Alphaproteobacteria;o__Dongiales;f__Dongiaceae;g__Dongia
OTU_502	3	11	5	0.01%	k__Bacteria;p__Spirochaetota;c__Leptospirae;o__Leptospirales;f__Leptospiraceae;g__Leptospira;s__
OTU_503	0	0	0	0.00%	k__Bacteria;p__Actinobacteriota;c__Coriobacteriia;o__Coriobacteriales;f__Eggerthellaceae;g__Enterorhabdus;s__
OTU_504	15	13	18	0.02%	k__Bacteria;p__Bacteroidota;c__Bacteroidia;o__Bacteroidales;f__Prevotellaceae;g__Prevotella_9;s__
OTU_505	13	8	4	0.01%	k__Bacteria;p__Firmicutes;c__Clostridia;o__Lachnospirales;f__Lachnospiraceae;g__Lachnospiraceae_NK4A136_group;s__
OTU_506	10	8	11	0.01%	k__Bacteria;p__Bacteroidota;c__Bacteroidia;o__Bacteroidales;f__Prevotellaceae;g__Paraprevotella
OTU_507	10	8	12	0.02%	k__Bacteria;p__Firmicutes;c__Negativicutes;o__Veillonellales-Selenomonadales;f__Veillonellaceae;g__Megasphaera;s__
OTU_508	1	2	2	0.00%	k__Bacteria;p__Gracilibacteria
OTU_509	6	6	8	0.01%	k__Bacteria;p__Bacteroidota;c__Bacteroidia
OTU_510	0	0	4	0.00%	k__Bacteria;p__Bacteroidota;c__Bacteroidia;o__Bacteroidales;f__Rikenellaceae;g__Alistipes;s__
OTU_511	0	4	2	0.00%	k__Bacteria;p__Gracilibacteria;c__unidentified_Gracilibacteria;o__unidentified_Gracilibacteria;f__unidentified_Gracilibacteria; g__unidentified_Gracilibacteria;s__Gracilibacteria_bacterium_canine_oral_taxon_291
OTU_512	1	6	3	0.01%	k__Bacteria;p__Bacteroidota;c__Bacteroidia;o__Bacteroidales;f__Bacteroidaceae;g__Bacteroides;s__
OTU_513	7	5	1	0.01%	k__Bacteria;p__Firmicutes;c__Negativicutes;o__Veillonellales-Selenomonadales;f__Selenomonadaceae;g__Propionispira;s__
OTU_514	8	2	1	0.01%	k__Bacteria;p__Kapabacteria;c__unidentified_Kapabacteria;o__Kapabacteriales;f__;g__;s__
OTU_515	1	2	0	0.00%	k__Bacteria;p__Firmicutes;c__Clostridia;o__Oscillospirales;f__Oscillospiraceae;g__Colidextribacter;s__
OTU_516	8	7	2	0.01%	k__Bacteria;p__Firmicutes;c__Clostridia;o__Lachnospirales;f__Lachnospiraceae
OTU_517	6	7	3	0.01%	k__Bacteria;p__Bacteroidota;c__Bacteroidia;o__Sphingobacteriales;f__NS11-12_marine_group
OTU_518	6	0	7	0.01%	k__Bacteria;p__Proteobacteria;c__Gammaproteobacteria;o__Xanthomonadales;f__Xanthomonadaceae;g__Arenimonas;s__
OTU_519	0	2	5	0.00%	k__Bacteria;p__Desulfobacterota;c__Desulfovibrionia;o__Desulfovibrionales;f__Desulfovibrionaceae;g__Desulfovibrio;s__Desulfovibrio_piger
OTU_520	6	0	7	0.01%	k__Bacteria;p__Firmicutes;c__Clostridia;o__Peptostreptococcales-Tissierellales;f__unidentified_Peptostreptococcales-Tissierellales;g__Gottschalkia;s__
OTU_521	16	5	1	0.01%	k__Bacteria;p__Actinobacteriota;c__Coriobacteriia;o__Coriobacteriales;f__Coriobacteriaceae;g__Collinsella;s__Collinsella_stercoris
OTU_522	0	2	1	0.00%	k__Bacteria;p__unidentified_Bacteria;c__Gammaproteobacteria;o__Burkholderiales;f__Rhodocyclaceae;g__;s__
OTU_523	9	5	2	0.01%	k__Bacteria;p__Firmicutes;c__Clostridia;o__Clostridiales;f__Clostridiaceae;g__Clostridium_sensu_stricto_1;s__Clostridium_perfringens
OTU_524	3	2	5	0.01%	k__Bacteria;p__Bacteroidota;c__Bacteroidia;o__Cytophagales
OTU_525	225	88	199	0.26%	k__Bacteria;p__Proteobacteria;c__Gammaproteobacteria;o__Burkholderiales;f__Comamonadaceae;g__Rhodoferax;s__
OTU_526	9	8	2	0.01%	k__Bacteria;p__Bacteroidota;c__Bacteroidia;o__Flavobacteriales;f__Flavobacteriaceae;g__Flavobacterium
OTU_527	9	11	1	0.01%	k__Bacteria;p__Firmicutes;c__Clostridia;o__Lachnospirales;f__Lachnospiraceae
OTU_528	5	4	7	0.01%	k__Bacteria;p__Proteobacteria;c__Gammaproteobacteria;o__Burkholderiales
OTU_529	12	1	6	0.01%	k__Bacteria;p__Bacteroidota;c__Bacteroidia;o__Chitinophagales;f__Chitinophagaceae;g__Edaphobaculum;s__
OTU_530	33	4	2	0.02%	k__Bacteria;p__Firmicutes;c__Bacilli;o__Alicyclobacillales;f__Alicyclobacillaceae;g__Tumebacillus;s__
OTU_531	0	0	0	0.00%	k__Bacteria;p__Firmicutes;c__Clostridia;o__Peptostreptococcales-Tissierellales;f__Peptostreptococcaceae;g__Peptostreptococcus;s__
OTU_532	6	3	3	0.01%	k__Bacteria;p__Firmicutes;c__Clostridia;o__Lachnospirales;f__Lachnospiraceae;g__Roseburia;s__
OTU_533	0	0	14	0.01%	k__Bacteria;p__Bacteroidota;c__Bacteroidia;o__Bacteroidales;f__Muribaculaceae;g__;s__
OTU_534	18	5	1	0.01%	k__Bacteria;p__Proteobacteria;c__Alphaproteobacteria;o__Rhizobiales;f__Beijerinckiaceae;g__Microvirga;s__
OTU_535	8	10	8	0.01%	k__Bacteria;p__Proteobacteria;c__Gammaproteobacteria;o__Pseudomonadales;f__Moraxellaceae;g__Alkanindiges;s__
OTU_536	2	5	4	0.01%	k__Bacteria;p__Desulfobacterota;c__Desulfuromonadia;o__Bradymonadales;f__;g__;s__
OTU_537	3	13	2	0.01%	k__Bacteria;p__Proteobacteria;c__Alphaproteobacteria;o__Sphingomonadales;f__Sphingomonadaceae;g__Erythrobacter;s__Erythrobacter_sp
OTU_538	0	0	0	0.00%	k__Bacteria;p__Bacteroidota;c__Bacteroidia;o__Bacteroidales;f__Rikenellaceae;g__Rikenella;s__
OTU_539	0	1	5	0.00%	k__Bacteria;p__unidentified_Bacteria;c__Bacilli;o__Erysipelotrichales;f__Erysipelotrichaceae;g__;s__
OTU_540	1	3	0	0.00%	k__Bacteria;p__Firmicutes;c__Clostridia;o__Oscillospirales;f__Oscillospiraceae;g__Oscillibacter;s__
OTU_541	5	5	7	0.01%	k__Bacteria;p__Firmicutes;c__Bacilli;o__Lactobacillales;f__Lactobacillaceae;g__Latilactobacillus;s__Lactobacillus_sakei
OTU_542	0	6	1	0.00%	k__Bacteria;p__Proteobacteria;c__Gammaproteobacteria;o__Pseudomonadales;f__Spongiibacteraceae;g__;s__
OTU_543	3	1	7	0.01%	k__Bacteria;p__Proteobacteria;c__Gammaproteobacteria;o__Pseudomonadales;f__Cellvibrionaceae;g__Cellvibrio
OTU_544	5	4	5	0.01%	k__Bacteria;p__Firmicutes;c__Clostridia;o__Oscillospirales;f__Ruminococcaceae;g__Ruminococcus
OTU_545	0	0	0	0.00%	k__Bacteria;p__Actinobacteriota;c__Coriobacteriia;o__Coriobacteriales;f__Eggerthellaceae;g__Parvibacter;s__
OTU_546	0	8	0	0.00%	k__Bacteria;p__Bacteroidota;c__Bacteroidia;o__Flavobacteriales;f__Crocinitomicaceae;g__Salinirepens;s__
OTU_547	2	2	2	0.00%	k__Bacteria;p__Firmicutes;c__Clostridia;o__Lachnospirales;f__Lachnospiraceae
OTU_548	1	1	4	0.00%	k__Bacteria;p__Bdellovibrionota;c__Bdellovibrionia;o__Bdellovibrionales;f__Bdellovibrionaceae;g__Bdellovibrio
OTU_549	4	3	7	0.01%	k__Bacteria;p__Desulfobacterota;c__Desulfobacteria;o__Desulfobacterales;f__Desulforegulaceae;g__Desulforegula;s__
OTU_550	3	1	5	0.00%	k__Bacteria;p__Fusobacteriota;c__Fusobacteriia;o__Fusobacteriales;f__Fusobacteriaceae;g__Cetobacterium;s__
OTU_551	5	3	7	0.01%	k__Bacteria;p__Gracilibacteria;c__unidentified_Gracilibacteria;o__JGI_0000069-P22;f__;g__;s__
OTU_552	3	0	4	0.00%	k__Bacteria;p__Proteobacteria;c__Gammaproteobacteria;o__Xanthomonadales;f__Xanthomonadaceae;g__Stenotrophomonas
OTU_553	4	0	6	0.01%	k__Bacteria;p__Proteobacteria;c__Gammaproteobacteria;o__unidentified_Gammaproteobacteria;f__unidentified_Gammaproteobacteria;g__;s__

OTU_554	0	0	0	0.00%	k__Bacteria;p__Bacteroidota;c__Bacteroidia;o__Bacteroidales;f__Bacteroidaceae;g__Bacteroides;s__Bacteroides_caecimuris
OTU_555	14	11	10	0.02%	k__Bacteria;p__Firmicutes;c__Bacilli;o__Erysipelotrichales;f__Erysipelatoclostridiaceae;g__Catenibacterium;s__
OTU_556	10	12	13	0.02%	k__Bacteria;p__Desulfobacterota;c__Desulfuromonadia;o__Geobacterales;f__Geobacteraceae;g__Trichlorobacter;s__
OTU_557	68	85	55	0.11%	k__Bacteria;p__Proteobacteria;c__Gammaproteobacteria;o__Burkholderiales;f__Oxalobacteraceae
OTU_558	2	5	0	0.00%	k__Bacteria;p__Bacteroidota;c__Bacteroidia;o__Cytophagales;f__Spirosomaceae;g__Dyadobacter;s__
OTU_559	5	3	9	0.01%	k__Bacteria;p__Firmicutes;c__Clostridia;o__Oscillospirales;f__Oscillospiraceae;g__UCG-005;s__
OTU_560	4	1	6	0.01%	k__Bacteria;p__Bacteroidota;c__Bacteroidia;o__Flavobacteriales;f__Weeksellaceae;g__Empedobacter;s__
OTU_561	0	1	9	0.01%	k__Bacteria;p__unidentified_Bacteria;c__Bacteroidia;o__Bacteroidales;f__Rikenellaceae;g__unidentified_Rikenellaceae;s__iron-reducing_enrichment_clone_Cl-A7
OTU_562	0	3	5	0.00%	k__Bacteria;p__unidentified_Bacteria;c__Gammaproteobacteria;o__Burkholderiales;f__Neisseriaceae;g__;s__
OTU_563	1	3	0	0.00%	k__Bacteria;p__Bacteroidota;c__Bacteroidia;o__Cytophagales;f__Spirosomaceae;g__Runella;s__
OTU_564	5	2	4	0.01%	k__Bacteria;p__Actinobacteria;c__unidentified_Actinobacteria;o__Propionibacteriales;f__Propionibacteriaceae;g__Tessaracoccus;s__
OTU_565	9	20	12	0.02%	k__Bacteria;p__Proteobacteria;c__Gammaproteobacteria;o__Burkholderiales;f__Rhodocyclaceae;g__Dechloromonas;s__
OTU_566	7	9	4	0.01%	k__Bacteria;p__Firmicutes;c__Clostridia;o__Peptostreptococcales-Tissierellales;f__Peptostreptococcaceae;g__Paeniclostridium
OTU_567	0	0	16	0.01%	k__Bacteria;p__Bacteroidota;c__Bacteroidia;o__Bacteroidales;f__Tannerellaceae;g__Parabacteroides
OTU_568	0	0	0	0.00%	k__Bacteria;p__Bacteroidota;c__Bacteroidia;o__Bacteroidales;f__Muribaculaceae;g__;s__
OTU_569	2	4	2	0.00%	k__Bacteria;p__unidentified_Bacteria;c__Bacteroidia;o__Bacteroidales;f__Marinifilaceae;g__;s__
OTU_570	2	2	4	0.00%	k__Bacteria;p__Firmicutes;c__Clostridia;o__Oscillospirales;f__Hungateiclostridiaceae;g__Ercella
OTU_571	0	0	0	0.00%	k__Bacteria;p__Firmicutes;c__Clostridia;o__Lachnospirales;f__Lachnospiraceae;g__Stomatobaculum;s__
OTU_572	2	1	1	0.00%	k__Bacteria;p__Bacteroidota;c__Bacteroidia;o__Sphingobacteriales;f__NS11-12_marine_group;g__;s__
OTU_573	3	3	3	0.00%	k__Bacteria;p__Firmicutes;c__Negativicutes;o__Veillonellales-Selenomonadales;f__Sporomusaceae;g__Acetonema;s__
OTU_574	0	0	0	0.00%	k__Bacteria;p__Firmicutes;c__Clostridia;o__Clostridia_vadinBB60_group;f__;g__;s__
OTU_575	5	2	2	0.00%	k__Bacteria;p__Proteobacteria;c__Gammaproteobacteria;o__Burkholderiales;f__Chromobacteriaceae;g__Vogesella
OTU_576	12	9	7	0.01%	k__Bacteria;p__Firmicutes;c__Clostridia;o__Clostridiales;f__Clostridiaceae;g__Clostridium_sensu_stricto_1
OTU_577	0	1	10	0.01%	k__Bacteria;p__Firmicutes;c__Bacilli;o__Lactobacillales;f__Streptococcaceae;g__Streptococcus;s__Streptococcus_anginosus
OTU_578	2	5	2	0.00%	k__Bacteria;p__Firmicutes;c__Clostridia;o__Lachnospirales;f__Lachnospiraceae
OTU_579	0	0	0	0.00%	k__Bacteria;p__Bacteroidota;c__Bacteroidia;o__Bacteroidales;f__Prevotellaceae;g__Alloprevotella;s__
OTU_580	4	1	2	0.00%	k__Bacteria;p__Proteobacteria;c__Gammaproteobacteria;o__Burkholderiales;f__Rhodocyclaceae;g__Uliginosibacterium;s__
OTU_581	15	0	0	0.01%	k__Bacteria;p__Firmicutes;c__Clostridia;o__Peptococcales;f__Peptococcaceae;g__Peptococcus;s__
OTU_582	4	1	0	0.00%	k__Bacteria;p__Bacteroidota;c__Bacteroidia;o__Bacteroidales;f__Barnesiellaceae;g__Barnesiella;s__
OTU_583	8	5	5	0.01%	k__Bacteria;p__Proteobacteria;c__Gammaproteobacteria;o__Burkholderiales;f__Rhodocyclaceae;g__Uliginosibacterium;s__
OTU_584	3	0	0	0.00%	k__Bacteria;p__Proteobacteria;c__Gammaproteobacteria;o__Burkholderiales;f__Rhodocyclaceae;g__Azospira
OTU_585	21	14	6	0.02%	k__Bacteria;p__Proteobacteria;c__Alphaproteobacteria;o__Rhizobiales;f__Devosiaceae;g__Devosia;s__
OTU_586	3	5	6	0.01%	k__Bacteria;p__Synergistota;c__Synergistia;o__Synergistales;f__Synergistaceae;g__Lactivibrio;s__
OTU_587	3	2	1	0.00%	k__Bacteria;p__unidentified_Bacteria;c__Synergistia;o__Synergistales;f__Synergistaceae;g__;s__
OTU_588	0	1	1	0.00%	k__Bacteria;p__Firmicutes;c__Clostridia;o__Clostridia_UCG-014;f__;g__;s__
OTU_589	8	17	1	0.01%	k__Bacteria;p__Proteobacteria;c__Gammaproteobacteria;o__Enterobacterales;f__Pseudoalteromonadaceae;g__Pseudoalteromonas
OTU_590	1	6	4	0.01%	k__Bacteria;p__Myxococcota;c__Polyangia;o__Nannocystales;f__Nannocystaceae;g__Nannocystis;s__
OTU_591	0	2	2	0.00%	k__Bacteria;p__Firmicutes;c__Clostridia;o__Oscillospirales;f__Ruminococcaceae;g__UBA1819;s__
OTU_592	65	32	62	0.08%	k__Bacteria;p__Proteobacteria;c__Gammaproteobacteria;o__Burkholderiales;f__Comamonadaceae
OTU_593	2	4	2	0.00%	k__Bacteria;p__Proteobacteria;c__Gammaproteobacteria;o__Burkholderiales;f__Rhodocyclaceae;g__Sterolibacterium;s__
OTU_594	2	6	0	0.00%	k__Bacteria;p__unidentified_Bacteria;c__Clostridia;o__Lachnospirales;f__Lachnospiraceae
OTU_595	1	7	11	0.01%	k__Bacteria;p__Bacteroidota;c__Bacteroidia;o__Bacteroidales;f__Marinilabiliaceae;g__[Cytophaga]_xylanolytica_group;s__
OTU_596	5	2	7	0.01%	k__Bacteria;p__Proteobacteria;c__Gammaproteobacteria;o__Pseudomonadales;f__Moraxellaceae;g__Alkanindiges;s__Moraxellaceae_bacterium_HYN0046
OTU_597	5	2	1	0.00%	k__Bacteria;p__Bdellovibrionota;c__Bdellovibrionia;o__Bdellovibrionales;f__Bdellovibrionaceae;g__;s__
OTU_598	5	4	2	0.01%	k__Bacteria;p__Desulfobacterota;c__Desulfovibrionia;o__Desulfovibrionales;f__Desulfovibrionaceae;g__Bilophila;s__
OTU_599	13	13	2	0.01%	k__Bacteria;p__Proteobacteria;c__Gammaproteobacteria;o__Xanthomonadales;f__Xanthomonadaceae;g__Stenotrophomonas;s__Stenotrophomonas_chelatiphaga
OTU_600	2	34	10	0.02%	k__Bacteria;p__Bacteroidota;c__Bacteroidia;o__Cytophagales;f__Hymenobacteraceae;g__Hymenobacter;s__
OTU_601	3	2	4	0.00%	k__Bacteria;p__Spirochaetota;c__Leptospirae;o__Leptospirales;f__Leptospiraceae;g__Leptospira;s__Leptospira_biflexa
OTU_602	0	0	5	0.00%	k__Bacteria;p__Bacteroidota;c__Bacteroidia;o__Bacteroidales;f__Muribaculaceae;g__;s__
OTU_603	7	4	4	0.01%	k__Bacteria;p__Proteobacteria;c__Alphaproteobacteria;o__Rhizobiales;f__unidentified_Rhizobiales;g__Phreatobacter
OTU_604	0	0	15	0.01%	k__Bacteria;p__Bacteroidota;c__Bacteroidia;o__Bacteroidales;f__Muribaculaceae;g__Muribaculum;s__
OTU_605	4	3	3	0.01%	k__Bacteria;p__Firmicutes;c__Clostridia;o__Peptostreptococcales-Tissierellales;f__Fusibacteraceae;g__Fusibacter
OTU_606	6	2	10	0.01%	k__Bacteria;p__Bacteroidota;c__Bacteroidia;o__Bacteroidales;f__Marinilabiliaceae;g__[Cytophaga]_xylanolytica_group
OTU_607	2	1	1	0.00%	k__Bacteria;p__Bacteroidota;c__Bacteroidia;o__Bacteroidales;f__Tannerellaceae;g__Parabacteroides;s__
OTU_608	0	11	0	0.01%	k__Bacteria;p__Firmicutes;c__Bacilli;o__Lactobacillales;f__Aerococcaceae;g__Globicatella;s__
OTU_609	2	3	3	0.00%	k__Bacteria;p__Proteobacteria;c__Gammaproteobacteria;o__Burkholderiales;f__Rhodocyclaceae

OTU_610	4	1	1	0.00%	k__Bacteria;p__Actinobacteria;c__unidentified_Actinobacteria;o__Bifidobacteriales;f__Bifidobacteriaceae;g__Bifidobacterium;s__Bifidobacterium_animalis
OTU_611	0	0	18	0.01%	k__Bacteria;p__Bacteroidota;c__Bacteroidia;o__Bacteroidales;f__Rikenellaceae;g__Rikenellaceae_RC9_gut_group;s__
OTU_612	32	23	42	0.05%	k__Bacteria;p__Bacteroidota;c__Bacteroidia;o__Flavobacteriales;f__Flavobacteriaceae;g__Flavobacterium;s__Flavobacterium_sp_HME6125
OTU_613	3	9	2	0.01%	k__Bacteria;p__Proteobacteria;c__Alphaproteobacteria;o__Caulobacterales;f__Caulobacteraceae;g__Brevundimonas;s__Brevundimonas_vesicularis
OTU_614	74	47	94	0.11%	k__Bacteria;p__Proteobacteria;c__Gammaproteobacteria;o__Burkholderiales;f__Comamonadaceae;g__Limnohabitans;s__
OTU_615	3	9	3	0.01%	k__Bacteria;p__Actinobacteria;c__unidentified_Actinobacteria;o__Kineosporiales;f__Kineosporiaceae;g__Quadrisphaera;s__Quadrisphaera_granulorum
OTU_616	3	9	6	0.01%	k__Bacteria;p__Firmicutes;c__Negativicutes;o__Veillonellales-Selenomonadales;f__Selenomonadaceae;g__Anaerovibrio;s__
OTU_617	2	0	4	0.00%	k__Bacteria;p__Firmicutes;c__Bacilli;o__Erysipelotrichales;f__Erysipelatoclostridiaceae;g__Erysipelatoclostridium;s__Erysipelatoclostridium_ramosum
OTU_618	10	2	0	0.01%	k__Bacteria;p__Proteobacteria;c__Gammaproteobacteria;o__Pseudomonadales;f__Halomonadaceae;g__Halomonas
OTU_619	0	0	8	0.00%	k__Bacteria;p__Proteobacteria;c__Gammaproteobacteria;o__Burkholderiales;f__Neisseriaceae;g__Conchiformibius;s__Neisseria_canis
OTU_620	1	2	1	0.00%	k__Bacteria;p__Firmicutes;c__Clostridia;o__Oscillospirales;f__Ruminococcaceae;g__Ruminococcus;s__Clostridiaceae_bacterium_DJF_VR76
OTU_621	57	51	42	0.08%	k__Bacteria;p__Proteobacteria;c__Gammaproteobacteria;o__Pseudomonadales;f__Pseudomonadaceae;g__Pseudomonas
OTU_622	2	0	1	0.00%	k__Bacteria;p__Proteobacteria;c__Gammaproteobacteria
OTU_623	5	1	6	0.01%	k__Bacteria;p__unidentified_Bacteria;c__Bacteroidia;o__Chitinophagales;f__Saprospiraceae;g__;s__
OTU_624	3	4	1	0.00%	k__Bacteria;p__unidentified_Bacteria;c__Clostridia;o__Lachnospirales;f__Lachnospiraceae;g__unidentified_Lachnospiraceae;s__bacterium_enrichment_culture_clone_Ecwsrb026
OTU_625	0	13	0	0.01%	k__Bacteria;p__Proteobacteria;c__Gammaproteobacteria;o__Burkholderiales;f__Comamonadaceae;g__Schlegelella;s__
OTU_626	19	7	13	0.02%	k__Bacteria
OTU_627	6	3	1	0.01%	k__Bacteria;p__Firmicutes;c__Clostridia;o__Lachnospirales;f__Lachnospiraceae;g__Shuttleworthia;s__
OTU_628	6	4	3	0.01%	k__Bacteria;p__Proteobacteria;c__Gammaproteobacteria;o__Pseudomonadales;f__Cellvibrionaceae;g__Cellvibrio;s__
OTU_629	0	0	0	0.00%	k__Bacteria;p__Cyanobacteria;c__Vampirivibrionia;o__Gastranaerophilales;f__;g__;s__
OTU_630	4	2	5	0.01%	k__Bacteria;p__Desulfobacterota;c__Desulfovibrionia;o__Desulfovibrionales;f__Desulfovibrionaceae;g__Desulfovibrio;s__bacterium_endosymbiont_of_Onthophagus_Taurus
OTU_631	1	0	7	0.00%	k__Bacteria;p__Cyanobacteria
OTU_632	0	2	0	0.00%	k__Bacteria;p__Firmicutes;c__Clostridia;o__Oscillospirales;f__Ruminococcaceae;g__Faecalibacterium;s__
OTU_633	1	1	0	0.00%	k__Bacteria;p__unidentified_Bacteria;c__Alphaproteobacteria;o__Paracaedibacterales;f__Paracaedibacteraceae;g__;s__
OTU_634	0	6	1	0.00%	k__Bacteria;p__Firmicutes;c__Clostridia;o__Christensenellales;f__Christensenellaceae;g__Christensenellaceae_R-7_group;s__
OTU_635	3	5	4	0.01%	k__Bacteria;p__Proteobacteria;c__Gammaproteobacteria;o__Competibacterales;f__Competibacteraceae;g__Plasticicumulans;s__
OTU_636	14	0	1	0.01%	k__Bacteria;p__Proteobacteria;c__Gammaproteobacteria;o__Steroidobacterales;f__Steroidobacteraceae;g__Steroidobacter;s__
OTU_637	2	1	1	0.00%	k__Bacteria;p__Firmicutes;c__Clostridia;o__Peptostreptococcales-Tissierellales
OTU_638	4	1	0	0.00%	k__Bacteria;p__unidentified_Bacteria;c__Bacteroidia;o__Bacteroidales;f__;g__;s__
OTU_639	3	3	0	0.00%	k__Bacteria;p__Fibrobacterota;c__Fibrobacteria;o__Fibrobacterales;f__Fibrobacteraceae;g__Fibrobacter;s__
OTU_640	0	0	0	0.00%	k__Bacteria;p__Bacteroidota;c__Bacteroidia;o__Bacteroidales;f__Prevotellaceae;g__Prevotella;s__Prevotella_salivae
OTU_641	2	3	4	0.00%	k__Bacteria;p__Bacteroidota;c__Bacteroidia;o__Bacteroidales;f__Prevotellaceae
OTU_642	6	6	1	0.01%	k__Bacteria;p__Proteobacteria;c__Alphaproteobacteria;o__Sphingomonadales;f__Sphingomonadaceae;g__Sphingomonas;s__Rhizorhabdus_dicambivorans
OTU_643	0	0	0	0.00%	k__Bacteria;p__Bacteroidota;c__Bacteroidia;o__Bacteroidales;f__Porphyromonadaceae;g__Porphyromonas;s__Porphyromonas_endodontalis
OTU_644	9	0	0	0.00%	k__Bacteria;p__Firmicutes;c__Bacilli;o__Bacillales;f__Planococcaceae;g__Lysinibacillus
OTU_645	20	16	0	0.02%	k__Bacteria;p__Firmicutes;c__Clostridia;o__Lachnospirales;f__Lachnospiraceae;g__Lachnospiraceae_NK4A136_group;s__
OTU_646	14	39	10	0.03%	k__Bacteria;p__Proteobacteria;c__Gammaproteobacteria;o__Pseudomonadales;f__Moraxellaceae;g__Acinetobacter
OTU_647	15	2	5	0.01%	k__Bacteria;p__Proteobacteria;c__Gammaproteobacteria;o__Pseudomonadales;f__Moraxellaceae;g__Acinetobacter
OTU_648	3	9	3	0.01%	k__Bacteria;p__Proteobacteria;c__Alphaproteobacteria;o__Rhizobiales;f__Beijerinckiaceae;g__Methylobacterium-Methylorubrum;s__Methylobacterium_brachiatum
OTU_649	6	1	0	0.00%	k__Bacteria;p__Bacteroidota;c__Bacteroidia;o__Bacteroidales;f__Paludibacteraceae;g__Paludibacter;s__
OTU_650	2	3	1	0.00%	k__Bacteria;p__Firmicutes;c__Clostridia;o__Peptostreptococcales-Tissierellales;f__Anaerovoracaceae
OTU_651	4	2	2	0.00%	k__Bacteria;p__Bacteroidota;c__Bacteroidia;o__Bacteroidales;f__Bacteroidaceae;g__Bacteroides
OTU_652	98	132	124	0.18%	k__Bacteria;p__Campylobacterota;c__Campylobacteria;o__Campylobacterales;f__Arcobacteraceae;g__Arcobacter
OTU_653	5	3	3	0.01%	k__Bacteria;p__Actinobacteria;c__unidentified_Actinobacteria;o__Actinomycetales;f__Actinomycetaceae;g__Flaviflexus
OTU_654	10	18	5	0.02%	k__Bacteria;p__Proteobacteria;c__Gammaproteobacteria;o__Enterobacterales;f__Vibrionaceae;g__Vibrio
OTU_655	0	0	10	0.01%	k__Bacteria;p__Proteobacteria;c__Gammaproteobacteria;o__Xanthomonadales;f__Xanthomonadaceae;g__Thermomonas
OTU_656	2	1	2	0.00%	k__Bacteria;p__Bacteroidota;c__Bacteroidia;o__Bacteroidales;f__Dysgonomonadaceae;g__Dysgonomonas;s__Dysgonomonas_mossii
OTU_657	0	5	1	0.00%	k__Bacteria;p__Bacteroidota;c__Bacteroidia;o__Flavobacteriales;f__Crocinitomicaceae;g__Fluviicola;s__
OTU_658	2	2	5	0.00%	k__Bacteria
OTU_659	2	0	31	0.02%	k__Bacteria;p__Firmicutes;c__Clostridia;o__Clostridia_UCG-014;f__;g__;s__
OTU_660	37	24	66	0.06%	k__Bacteria;p__Actinobacteria;c__unidentified_Actinobacteria;o__Micrococcales;f__Microbacteriaceae;g__Microbacterium;s__Microbacterium_testaceum
OTU_661	7	8	10	0.01%	k__Bacteria;p__Proteobacteria;c__Gammaproteobacteria;o__Burkholderiales;f__Rhodocyclaceae;g__Sulfuritalea;s__
OTU_662	0	4	0	0.00%	k__Bacteria;p__Bacteroidota;c__Bacteroidia;o__Cytophagales;f__MWH-CFBk5;g__unidentified_MWH-CFBk5;s__Bacteroidetes_bacterium_MWH-CFBk5
OTU_663	3	0	7	0.01%	k__Bacteria;p__Actinobacteria;c__unidentified_Actinobacteria;o__Micrococcales;f__Bogoriellaceae;g__Georgenia;s__
OTU_664	0	0	1	0.00%	k__Bacteria;p__Firmicutes;c__Clostridia;o__Peptostreptococcales-Tissierellales;f__Anaerovoracaceae;g__Family_XIII_AD3011_group;s__
OTU_665	3	3	2	0.00%	k__Bacteria;p__Proteobacteria;c__Gammaproteobacteria;o__Xanthomonadales;f__Xanthomonadaceae;g__Arenimonas

OTU_666	3	3	1	0.00%	k__Bacteria;p__Synergistota;c__Synergistia;o__Synergistales;f__Synergistaceae;g__Syner-01;s__
OTU_667	2	3	1	0.00%	k__Bacteria;p__Bacteroidota;c__OC31;o__;f__;g__;s__
OTU_668	20	4	8	0.02%	k__Bacteria;p__Proteobacteria;c__Alphaproteobacteria;o__Sphingomonadales;f__Sphingomonadaceae;g__Novosphingobium
OTU_669	5	5	4	0.01%	k__Bacteria;p__Bacteroidota;c__Bacteroidia;o__Bacteroidales;f__Prevotellaceae;g__Prevotella;s__
OTU_670	6	5	4	0.01%	k__Bacteria;p__Proteobacteria;c__Gammaproteobacteria;o__Burkholderiales;f__Comamonadaceae;g__Caenimonas;s__
OTU_671	4	2	2	0.00%	k__Bacteria;p__Bdellovibrionota;c__Bdellovibrionia;o__Bacteriovoracales;f__Bacteriovoracaceae;g__Bacteriovorax;s__
OTU_672	14	18	13	0.02%	k__Bacteria;p__Proteobacteria;c__Alphaproteobacteria;o__Rhodobacterales;f__Rhodobacteraceae
OTU_673	1	0	5	0.00%	k__Bacteria
OTU_674	0	0	0	0.00%	k__Bacteria;p__Firmicutes;c__Bacilli;o__Erysipelotrichales;f__Erysipelatoclostridiaceae
OTU_675	0	0	0	0.00%	k__Bacteria;p__Campylobacterota;c__Campylobacteria;o__Campylobacterales;f__Campylobacteraceae;g__Campylobacter;s__Campylobacter_concisus
OTU_676	2	1	0	0.00%	k__Bacteria;p__Actinobacteria;c__unidentified_Actinobacteria;o__Actinomycetales;f__Actinomycetaceae;g__Actinomyces;s__Actinomyces_minihominis
OTU_677	2	0	3	0.00%	k__Bacteria;p__Proteobacteria;c__Gammaproteobacteria;o__Enterobacterales;f__Morganellaceae;g__Providencia;s__
OTU_678	0	0	0	0.00%	k__Bacteria;p__Deferribacteres;c__unidentified_Deferribacteres;o__Deferribacterales;f__Deferribacteraceae;g__Mucispirillum;s__Mucispirillum_schaedleri
OTU_679	0	0	6	0.00%	k__Bacteria;p__Proteobacteria;c__Gammaproteobacteria;o__Pseudomonadales;f__Moraxellaceae;g__Acinetobacter
OTU_680	0	0	0	0.00%	k__Bacteria;p__Firmicutes;c__Bacilli;o__Acholeplasmatales;f__Acholeplasmataceae;g__Anaeroplasma;s__
OTU_681	2	3	0	0.00%	k__Bacteria;p__Proteobacteria;c__Alphaproteobacteria;o__Rhodospirillales;f__Rhodospirillaceae;g__Insolitispirillum;s__
OTU_682	1	3	0	0.00%	k__Bacteria;p__Proteobacteria;c__Alphaproteobacteria;o__Zavarziniales;f__Zavarziniaceae;g__Zavarzinia;s__Zavarzinia_compransoris
OTU_683	0	3	1	0.00%	k__Bacteria;p__Bacteroidota;c__Bacteroidia
OTU_684	0	1	1	0.00%	k__Bacteria;p__Verrucomicrobiota;c__Verrucomicrobiae;o__Verrucomicrobiales;f__Verrucomicrobiaceae;g__Prosthecobacter;s__
OTU_685	2	3	4	0.00%	k__Bacteria;p__Bacteroidota;c__Bacteroidia;o__Cytophagales;f__Spirosomaceae;g__Flectobacillus
OTU_686	2	0	0	0.00%	k__Bacteria;p__Firmicutes;c__Bacilli;o__Lactobacillales;f__Carnobacteriaceae;g__Lacticigenium;s__
OTU_687	5	3	0	0.00%	k__Bacteria;p__Myxococcota;c__Polyangia;o__Haliangiales;f__Haliangiaceae;g__Haliangium;s__
OTU_688	6	3	0	0.00%	k__Bacteria;p__Bacteroidota;c__Bacteroidia;o__Bacteroidales;f__Rikenellaceae;g__Anaerocella;s__
OTU_689	2	1	7	0.01%	k__Bacteria;p__Proteobacteria;c__Gammaproteobacteria;o__Burkholderiales;f__Chromobacteriaceae;g__Vogesella;s__Vogesella_amnigena
OTU_690	1	3	0	0.00%	k__Bacteria;p__Bacteroidota;c__Bacteroidia;o__Bacteroidales;f__Rikenellaceae;g__Alistipes;s__Alistipes_obesi
OTU_691	1	3	3	0.00%	k__Bacteria;p__Bacteroidota;c__Bacteroidia;o__Flavobacteriales;f__Crocinitomicaceae;g__Fluviicola;s__
OTU_692	8	0	1	0.00%	k__Bacteria;p__Proteobacteria;c__Alphaproteobacteria;o__Rhodobacterales;f__Rhodobacteraceae;g__Rubellimicrobium;s__
OTU_693	0	5	1	0.00%	k__Bacteria;p__Bacteroidota;c__Bacteroidia;o__Flavobacteriales;f__Crocinitomicaceae;g__Fluviicola;s__
OTU_694	10	12	0	0.01%	k__Bacteria;p__Actinobacteria;c__unidentified_Actinobacteria;o__Micrococcales;f__Cellulomonadaceae;g__Cellulomonas;s__
OTU_695	10	0	0	0.01%	k__Bacteria;p__Firmicutes;c__Bacilli;o__Thermoactinomycetales;f__Thermoactinomycetaceae;g__Thermoactinomyces;s__Thermoactinomyces_vulgaris
OTU_696	2	9	3	0.01%	k__Bacteria;p__Bacteroidota;c__Bacteroidia;o__Bacteroidales;f__Paludibacteraceae;g__Paludibacter;s__
OTU_697	1	1	2	0.00%	k__Bacteria;p__Proteobacteria;c__Gammaproteobacteria;o__Burkholderiales;f__Rhodocyclaceae
OTU_698	11	0	3	0.01%	k__Bacteria;p__Bacteroidota;c__Bacteroidia;o__Flavobacteriales;f__Weeksellaceae;g__Chryseobacterium
OTU_699	1	2	6	0.00%	k__Bacteria;p__Bacteroidota;c__Bacteroidia;o__Bacteroidales;f__Dysgonomonadaceae;g__Proteiniphilum;s__
OTU_700	4	1	3	0.00%	k__Bacteria;p__Bacteroidota;c__Bacteroidia;o__Sphingobacteriales;f__NS11-12_marine_group;g__;s__
OTU_701	0	0	4	0.00%	k__Bacteria;p__unidentified_Bacteria;c__Alphaproteobacteria;o__Rhizobiales;f__Pleomorphomonadaceae;g__;s__
OTU_702	4	5	6	0.01%	k__Bacteria;p__Desulfobacterota;c__Desulfobacteria;o__Desulfobacterales;f__Desulforegulaceae;g__Desulforegula;s__
OTU_703	10	7	10	0.01%	k__Bacteria;p__Proteobacteria;c__Gammaproteobacteria;o__Pseudomonadales;f__Pseudomonadaceae;g__Thiopseudomonas;s__
OTU_704	0	0	0	0.00%	k__Bacteria;p__Firmicutes;c__Clostridia;o__Lachnospirales;f__Lachnospiraceae
OTU_705	0	1	8	0.00%	k__Bacteria;p__Campylobacterota;c__Campylobacteria;o__Campylobacterales;f__Helicobacteraceae;g__Helicobacter
OTU_706	13	9	12	0.02%	k__Bacteria;p__Firmicutes;c__Clostridia;o__Lachnospirales;f__Lachnospiraceae;g__Lachnospiraceae_FCS020_group;s__
OTU_707	5	8	2	0.01%	k__Bacteria;p__Firmicutes;c__Negativicutes;o__Veillonellales-Selenomonadales;f__Selenomonadaceae;g__Mitsuokella;s__
OTU_708	7	2	8	0.01%	k__Bacteria;p__unidentified_Bacteria;c__Saccharimonadia;o__Saccharimonadales;f__Saccharimonadaceae;g__TM7a;s__
OTU_709	4	0	1	0.00%	k__Bacteria;p__Firmicutes;c__Clostridia;o__Lachnospirales;f__Lachnospiraceae;g__Oribacterium;s__
OTU_710	12	9	4	0.01%	k__Bacteria;p__Desulfobacterota;c__Desulfobulbia;o__Desulfobulbales;f__Desulfobulbaceae;g__Desulfobulbus;s__
OTU_711	4	4	1	0.00%	k__Bacteria;p__Proteobacteria;c__Gammaproteobacteria;o__Pseudomonadales;f__Pseudohongiellaceae;g__Pseudohongiella;s__
OTU_712	2	2	1	0.00%	k__Bacteria;p__unidentified_Bacteria;c__Bacteroidia;o__Bacteroidales;f__;g__;s__
OTU_713	0	0	0	0.00%	k__Bacteria;p__Actinobacteriota;c__Coriobacteriia;o__Coriobacteriales;f__Eggerthellaceae;g__Enterorhabdus;s__
OTU_714	2	1	0	0.00%	k__Bacteria;p__Bacteroidota;c__Bacteroidia;o__Bacteroidales;f__Prolixibacteraceae;g__Roseimarinus;s__
OTU_715	6	3	0	0.00%	k__Bacteria;p__Bacteroidota;c__Bacteroidia;o__Bacteroidales;f__Bacteroidaceae;g__Bacteroides;s__
OTU_716	0	0	0	0.00%	k__Bacteria;p__Proteobacteria;c__Gammaproteobacteria;o__Burkholderiales;f__Neisseriaceae;g__Alysiella;s__
OTU_717	3	16	5	0.01%	k__Bacteria;p__Actinobacteria;c__unidentified_Actinobacteria;o__Frankiales;f__Geodermatophilaceae;g__Modestobacter
OTU_718	1	1	3	0.00%	k__Bacteria;p__Verrucomicrobiota;c__Verrucomicrobiae;o__Pedosphaerales;f__Pedosphaeraceae;g__DEV114;s__
OTU_719	13	13	13	0.02%	k__Bacteria;p__Actinobacteria;c__unidentified_Actinobacteria;o__Micrococcales;f__Microbacteriaceae;g__Leucobacter;s__
OTU_720	4	3	5	0.01%	k__Bacteria;p__unidentified_Bacteria;c__Gammaproteobacteria;o__Pseudomonadales;f__Moraxellaceae;g__;s__
OTU_721	0	0	2	0.00%	k__Bacteria;p__Spirochaetota;c__MVP-15

OTU_722	0	7	0	0.00%	k__Bacteria;p__Actinobacteria;c__unidentified_Actinobacteria;o__Propionibacteriales;f__Propionibacteriaceae;g__Friedmanniella;s__
OTU_723	2	4	1	0.00%	k__Bacteria;p__Gracilibacteria;c__unidentified_Gracilibacteria;o__unidentified_Gracilibacteria;f__unidentified_Gracilibacteria;g__unidentified_Gracilibacteria
OTU_724	2	6	6	0.01%	k__Bacteria;p__Spirochaetota;c__Spirochaetia;o__Spirochaetales;f__Spirochaetaceae;g__Treponema;s__Treponema_zuelzerae
OTU_725	3	1	2	0.00%	k__Bacteria;p__Proteobacteria;c__Gammaproteobacteria;o__Burkholderiales;f__Chitinibacteraceae;g__Chitinibacter;s__
OTU_726	15	5	0	0.01%	k__Bacteria;p__Actinobacteria;c__unidentified_Actinobacteria;o__Propionibacteriales;f__Nocardioidaceae;g__Marmoricola
OTU_727	0	0	10	0.01%	k__Bacteria;p__Firmicutes;c__Clostridia;o__Lachnospirales;f__Lachnospiraceae;g__Lachnospiraceae_NK4A136_group;s__
OTU_728	1	0	0	0.00%	k__Bacteria;p__Firmicutes;c__Clostridia;o__Christensenellales;f__Christensenellaceae;g__Christensenellaceae_R-7_group
OTU_729	2	2	5	0.00%	k__Bacteria;p__Bacteroidota;c__Bacteroidia;o__Bacteroidales;f__Paludibacteraceae;g__Paludibacter;s__
OTU_730	1	3	1	0.00%	k__Bacteria;p__Campylobacterota;c__Campylobacteria;o__Campylobacterales;f__Sulfurovaceae;g__Sulfurovum
OTU_731	0	4	3	0.00%	k__Bacteria;p__Bacteroidota;c__Bacteroidia;o__Bacteroidales;f__Tannerellaceae;g__Parabacteroides;s__
OTU_732	1	0	1	0.00%	k__Bacteria;p__Bacteroidota;c__Bacteroidia;o__Bacteroidales;f__Rikenellaceae;g__Anaerocella;s__
OTU_733	3	4	5	0.01%	k__Bacteria;p__Gracilibacteria;c__unidentified_Gracilibacteria;o__JGI_0000069-P22;f__;g__;s__
OTU_734	3	3	2	0.00%	k__Bacteria;p__Myxococcota;c__Polyangia;o__Polyangiales;f__Sandaracinaceae;g__Sandaracinus;s__
OTU_735	3	1	3	0.00%	k__Bacteria;p__Actinobacteriota;c__Coriobacteriia;o__Coriobacteriales;f__Eggerthellaceae;g__Senegalimassilia;s__
OTU_736	1	0	1	0.00%	k__Bacteria;p__Firmicutes;c__Desulfitobacteriia;o__Desulfitobacteriales;f__unidentified_Desulfitobacteriales;g__TC1;s__
OTU_737	3	6	6	0.01%	k__Bacteria;p__Desulfobacterota;c__Desulfovibrionia;o__Desulfovibrionales;f__Desulfomicrobiaceae;g__Desulfomicrobium;s__
OTU_738	0	0	0	0.00%	k__Bacteria;p__Bacteroidota;c__Bacteroidia;o__Bacteroidales;f__Barnesiellaceae;g__Coprobacter;s__
OTU_739	2	3	0	0.00%	k__Bacteria;p__Bacteroidota;c__Bacteroidia;o__Flavobacteriales;f__Crocinitomicaceae;g__Fluviicola;s__
OTU_740	4	0	2	0.00%	k__Bacteria;p__Proteobacteria;c__Gammaproteobacteria;o__Beggiatoales;f__Beggiatoaceae;g__Beggiatoa
OTU_741	2	1	6	0.00%	k__Bacteria;p__Firmicutes;c__Bacilli;o__Lactobacillales;f__Lactobacillaceae;g__Paucilactobacillus;s__
OTU_742	0	0	2	0.00%	k__Bacteria;p__Proteobacteria;c__Gammaproteobacteria;o__Burkholderiales;f__Nitrosomonadaceae;g__Nitrosomonas;s__
OTU_743	2	0	5	0.00%	k__Bacteria;p__Proteobacteria;c__Gammaproteobacteria;o__unidentified_Gammaproteobacteria;f__unidentified_Gammaproteobacteria;g__;s__
OTU_744	1	6	2	0.00%	k__Bacteria;p__Firmicutes;c__Negativicutes;o__Acidaminococcales;f__Acidaminococcaceae;g__Acidaminococcus;s__
OTU_745	3	2	3	0.00%	k__Bacteria;p__unidentified_Bacteria;c__Negativicutes;o__Veillonellales-Selenomonadales;f__Sporomusaceae;g__;s__
OTU_746	2	3	2	0.00%	k__Bacteria;p__unidentified_Bacteria;c__Gammaproteobacteria;o__Diplorickettsiales;f__Diplorickettsiaceae;g__;s__
OTU_747	0	2	0	0.00%	k__Bacteria;p__Firmicutes;c__Bacilli;o__Lactobacillales;f__Lactobacillaceae;g__Levilactobacillus;s__Lactobacillus_brevis
OTU_748	0	0	5	0.00%	k__Bacteria;p__Firmicutes;c__Clostridia;o__Oscillospirales;f__Ruminococcaceae;g__Anaerotruncus;s__
OTU_749	2	1	0	0.00%	k__Bacteria;p__Bacteroidota;c__Bacteroidia;o__Bacteroidales;f__Prevotellaceae;g__Paraprevotella;s__Paraprevotella_xylaniphila
OTU_750	1	1	2	0.00%	k__Bacteria;p__unidentified_Bacteria;c__Clostridia;o__Oscillospirales;f__Ruminococcaceae;g__;s__
OTU_751	0	2	2	0.00%	k__Bacteria;p__Acidobacteriota;c__Blastocatellia
OTU_752	8	2	0	0.01%	k__Bacteria;p__unidentified_Bacteria;c__Vicinamibacteria;o__Vicinamibacterales;f__;g__;s__
OTU_753	0	3	2	0.00%	k__Bacteria;p__Firmicutes;c__Bacilli;o__Lactobacillales;f__Carnobacteriaceae;g__Atopostipes;s__
OTU_754	5	0	1	0.00%	k__Bacteria;p__Firmicutes;c__Bacilli;o__Bacillales;f__Bacillaceae
OTU_755	3	3	1	0.00%	k__Bacteria;p__Firmicutes;c__Clostridia;o__Lachnospirales;f__Lachnospiraceae;g__Tyzzerella;s__Anaerotignum_lactatifermentans
OTU_756	4	2	0	0.00%	k__Bacteria;p__Bacteroidota;c__Bacteroidia;o__Chitinophagales;f__Chitinophagaceae;g__Cnuella
OTU_757	3	1	3	0.00%	k__Bacteria;p__Bacteroidota;c__Bacteroidia;o__Sphingobacteriales;f__env.OPS_17;g__;s__
OTU_758	4	5	3	0.01%	k__Bacteria;p__Bacteroidota;c__Bacteroidia;o__Chitinophagales;f__Chitinophagaceae;g__Flaviaesturariibacter;s__
OTU_759	0	2	1	0.00%	k__Bacteria;p__Proteobacteria;c__Gammaproteobacteria;o__Beggiatoales;f__Beggiatoaceae;g__Beggiatoa;s__Beggiatoa_sp_AA5A
OTU_760	2	7	2	0.01%	k__Bacteria;p__Firmicutes;c__Clostridia;o__Eubacteriales;f__Eubacteriaceae;g__Acetobacterium;s__
OTU_761	6	0	0	0.00%	k__Bacteria;p__Proteobacteria;c__Gammaproteobacteria;o__Burkholderiales;f__Oxalobacteraceae;g__Massilia
OTU_762	3	2	0	0.00%	k__Bacteria;p__unidentified_Bacteria;c__Gammaproteobacteria;o__Burkholderiales;f__Neisseriaceae;g__unidentified_Neisseriaceae;s__Neisseriaceae_bacterium_DSM_100970
OTU_763	0	0	14	0.01%	k__Bacteria;p__Bacteroidota;c__Bacteroidia;o__Bacteroidales;f__Muribaculaceae;g__;s__
OTU_764	1	6	4	0.01%	k__Bacteria;p__Firmicutes;c__Clostridia;o__Eubacteriales;f__Eubacteriaceae
OTU_765	0	0	0	0.00%	k__Bacteria;p__Bacteroidota;c__Bacteroidia;o__Bacteroidales;f__Muribaculaceae;g__;s__
OTU_766	1	5	4	0.01%	k__Bacteria;p__Bacteroidota;c__Bacteroidia;o__Bacteroidales;f__Prevotellaceae;g__Alloprevotella;s__
OTU_767	0	1	2	0.00%	k__Bacteria;p__Firmicutes;c__Bacilli;o__Lactobacillales;f__Streptococcaceae;g__Lactococcus
OTU_768	1	3	0	0.00%	k__Bacteria;p__Cyanobacteria;c__Cyanobacteriia;o__Chloroplast
OTU_769	2	1	3	0.00%	k__Bacteria;p__Bacteroidota;c__Bacteroidia;o__Bacteroidales;f__Prolixibacteraceae;g__Roseimarinus;s__
OTU_770	6	3	14	0.01%	k__Bacteria;p__Proteobacteria;c__Gammaproteobacteria;o__Enterobacterales;f__Alteromonadaceae;g__Rheinheimera;s__Pararheinheimera_soli
OTU_771	3	5	4	0.01%	k__Bacteria;p__Bacteroidota;c__Bacteroidia;o__Flavobacteriales;f__Flavobacteriaceae;g__Flavobacterium;s__Flavobacterium_aquatile
OTU_772	1	0	0	0.00%	k__Bacteria;p__Proteobacteria;c__Gammaproteobacteria;o__Burkholderiales;f__Comamonadaceae;g__Inhella;s__
OTU_773	11	9	18	0.02%	k__Bacteria;p__Bacteroidota;c__Bacteroidia;o__Bacteroidales;f__Prevotellaceae;g__Prevotella_9;s__
OTU_774	1	1	0	0.00%	k__Bacteria;p__Firmicutes;c__Clostridia;o__Oscillospirales;f__[Eubacterium]_coprostanoligenes_group;g__;s__
OTU_775	8	0	6	0.01%	k__Bacteria;p__Proteobacteria;c__Gammaproteobacteria;o__Burkholderiales;f__Comamonadaceae
OTU_776	2	3	1	0.00%	k__Bacteria;p__Firmicutes;c__Clostridia;o__Peptostreptococcales-Tissierellales;f__Family_XI;g__Gallicola
OTU_777	0	0	0	0.00%	k__Bacteria;p__unidentified_Bacteria;c__Saccharimonadia;o__Saccharimonadales;f__Saccharimonadaceae;g__TM7x;s__

OTU_778	1	2	0	0.00%	k__Bacteria;p__Proteobacteria;c__Gammaproteobacteria;o__Xanthomonadales;f__Rhodanobacteraceae;g__Ahniella;s__
OTU_779	35	47	22	0.05%	k__Bacteria;p__Proteobacteria;c__Alphaproteobacteria;o__Sphingomonadales;f__Sphingomonadaceae;g__Sphingomonas;s__Sphingomonas_phyllosphaerae
OTU_780	8	0	0	0.00%	k__Bacteria;p__Proteobacteria;c__Gammaproteobacteria;o__Burkholderiales;f__Alcaligenaceae;g__Verticiella
OTU_781	3	1	2	0.00%	k__Bacteria;p__Actinobacteria;c__unidentified_Actinobacteria;o__Micrococcales;f__Demequinaceae;g__Demequina;s__
OTU_782	0	7	0	0.00%	k__Bacteria;p__Proteobacteria;c__Alphaproteobacteria;o__Rhizobiales
OTU_783	2	5	3	0.01%	k__Bacteria;p__unidentified_Bacteria;c__Acidimicrobiia;o__Microtrichales;f__;g__;s__
OTU_784	0	3	2	0.00%	k__Bacteria;p__Gracilibacteria
OTU_785	1	7	3	0.01%	k__Bacteria;p__Firmicutes
OTU_786	2	0	8	0.01%	k__Bacteria;p__Proteobacteria;c__Gammaproteobacteria;o__Enterobacterales;f__Alteromonadaceae;g__Alishewanella;s__
OTU_787	1	5	0	0.00%	k__Bacteria;p__Bacteroidota;c__Bacteroidia;o__Bacteroidales;f__Rikenellaceae;g__;s__
OTU_788	3	2	0	0.00%	k__Bacteria
OTU_789	2	0	3	0.00%	k__Bacteria;p__Bacteroidota;c__Bacteroidia;o__Sphingobacteriales;f__Sphingobacteriaceae;g__Sphingobacterium;s__Bacteroidetes_bacterium_RBE2CD-54
OTU_790	6	1	0	0.00%	k__Bacteria;p__Proteobacteria;c__Gammaproteobacteria;o__Burkholderiales;f__Sutterellaceae;g__AAP99;s__
OTU_791	2	4	2	0.00%	k__Bacteria;p__Bacteroidota;c__Bacteroidia;o__Flavobacteriales;f__Crocinitomicaceae;g__Fluviicola;s__
OTU_792	0	0	0	0.00%	k__Bacteria
OTU_793	4	2	0	0.00%	k__Bacteria;p__Firmicutes;c__Desulfitobacteriia;o__Desulfitobacteriales;f__unidentified_Desulfitobacteriales;g__TC1;s__
OTU_794	0	3	4	0.00%	k__Bacteria;p__Firmicutes;c__Negativicutes;o__Acidaminococcales;f__Acidaminococcaceae
OTU_795	2	0	1	0.00%	k__Bacteria;p__Proteobacteria;c__Gammaproteobacteria;o__Xanthomonadales;f__Xanthomonadaceae;g__Pseudoxanthomonas;s__
OTU_796	0	0	0	0.00%	k__Bacteria;p__unidentified_Bacteria;c__Clostridia;o__Peptococcales;f__Peptococcaceae;g__;s__
OTU_797	2	2	0	0.00%	k__Bacteria;p__unidentified_Bacteria;c__Bacilli;o__Erysipelotrichales;f__Erysipelotrichaceae;g__;s__
OTU_798	1	0	2	0.00%	k__Bacteria;p__Campylobacterota;c__Campylobacteria;o__Campylobacterales
OTU_799	0	0	4	0.00%	k__Bacteria
OTU_800	5	2	2	0.00%	k__Bacteria;p__Bacteroidota;c__Bacteroidia;o__Bacteroidales;f__Tannerellaceae;g__Parabacteroides;s__Parabacteroides_goldsteinii
OTU_801	3	8	3	0.01%	k__Bacteria;p__Actinobacteria;c__unidentified_Actinobacteria;o__Corynebacteriales;f__Nocardiaceae;g__Rhodococcus;s__Rhodococcus_fascians
OTU_802	0	1	0	0.00%	k__Bacteria;p__Firmicutes;c__Clostridia;o__Clostridia_vadinBB60_group;f__;g__;s__
OTU_803	0	0	2	0.00%	k__Bacteria;p__Acidobacteriota;c__Vicinamibacteria;o__Subgroup_17;f__;g__;s__
OTU_804	0	0	0	0.00%	k__Bacteria;p__Firmicutes;c__Clostridia;o__Oscillospirales;f__Ruminococcaceae;g__[Eubacterium]_siraeum_group;s__
OTU_805	0	0	0	0.00%	k__Bacteria;p__Bacteroidota;c__Bacteroidia;o__Bacteroidales;f__Rikenellaceae;g__Alistipes;s__Alistipes_sp_cv1
OTU_806	5	7	11	0.01%	k__Bacteria;p__Firmicutes;c__Clostridia;o__Oscillospirales;f__Oscillospiraceae;g__Colidextribacter;s__Clostridiales_bacterium_CCNA10
OTU_807	0	0	5	0.00%	k__Bacteria;p__Verrucomicrobiota;c__Verrucomicrobiae;o__Chthoniobacterales;f__Terrimicrobiaceae;g__Terrimicrobium;s__
OTU_808	1	4	0	0.00%	k__Bacteria;p__Actinobacteria;c__unidentified_Actinobacteria;o__Micrococcales;f__Bogoriellaceae;g__Georgenia;s__
OTU_809	1	1	2	0.00%	k__Bacteria;p__Proteobacteria;c__Gammaproteobacteria;o__Burkholderiales;f__Sutterellaceae;g__Sutterella;s__
OTU_810	14	7	10	0.02%	k__Bacteria;p__Firmicutes
OTU_811	5	1	1	0.00%	k__Bacteria;p__Verrucomicrobiota;c__Verrucomicrobiae;o__Verrucomicrobiales;f__Verrucomicrobiaceae;g__Prosthecobacter;s__
OTU_812	165	174	191	0.27%	k__Bacteria;p__Firmicutes;c__Clostridia;o__Lachnospirales;f__Lachnospiraceae;g__CAG-56;s__
OTU_813	0	0	1	0.00%	k__Bacteria;p__Firmicutes;c__Clostridia;o__Oscillospirales;f__Ruminococcaceae;g__CAG-352;s__
OTU_814	0	3	0	0.00%	k__Bacteria;p__unidentified_Bacteria;c__Synergistia;o__Synergistales;f__Synergistaceae
OTU_815	1	2	9	0.01%	k__Bacteria;p__Firmicutes;c__Clostridia;o__Oscillospirales;f__Hungateiclostridiaceae;g__Fastidiosipila
OTU_816	0	0	10	0.01%	k__Bacteria;p__Firmicutes;c__Bacilli;o__Erysipelotrichales;f__Erysipelatoclostridiaceae;g__Candidatus_Stoquefichus;s__
OTU_817	4	2	4	0.01%	k__Bacteria;p__Bacteroidota;c__Bacteroidia;o__Cytophagales;f__Spirosomaceae
OTU_818	8	5	1	0.01%	k__Bacteria;p__Spirochaetota;c__MVP-15;o__;f__;g__;s__
OTU_819	0	0	1	0.00%	k__Bacteria;p__Bacteroidota;c__Bacteroidia;o__Bacteroidales;f__Muribaculaceae;g__;s__
OTU_820	0	0	5	0.00%	k__Bacteria;p__Proteobacteria;c__Alphaproteobacteria;o__Rhodobacterales;f__Rhodobacteraceae
OTU_821	7	2	3	0.01%	k__Bacteria;p__Proteobacteria;c__Alphaproteobacteria;o__Acetobacterales;f__Acetobacteraceae;g__Roseomonas
OTU_822	4	2	0	0.00%	k__Bacteria;p__Proteobacteria;c__Gammaproteobacteria;o__Enterobacterales;f__Pasteurellaceae
OTU_823	2	0	5	0.00%	k__Bacteria
OTU_824	8	12	21	0.02%	k__Bacteria;p__Bacteroidota;c__Bacteroidia;o__Bacteroidales;f__Prevotellaceae;g__Alloprevotella;s__
OTU_825	0	0	0	0.00%	k__Bacteria;p__Firmicutes;c__Clostridia;o__Oscillospirales;f__Ruminococcaceae;g__Ruminococcus;s__
OTU_826	2	1	2	0.00%	k__Bacteria;p__Cyanobacteria;c__Cyanobacteriia;o__Chloroplast;f__unidentified_Chloroplast;g__unidentified_Chloroplast
OTU_827	5	1	1	0.00%	k__Bacteria
OTU_828	1	0	0	0.00%	k__Bacteria;p__Proteobacteria;c__Gammaproteobacteria;o__Burkholderiales;f__Sutterellaceae;g__Parasutterella;s__Burkholderiales_bacterium_YL45
OTU_829	4	3	4	0.01%	k__Bacteria;p__unidentified_Bacteria;c__Gammaproteobacteria;o__Xanthomonadales;f__Rhodanobacteraceae;g__;s__
OTU_830	0	0	0	0.00%	k__Bacteria;p__Firmicutes;c__Clostridia;o__Clostridia_vadinBB60_group;f__;g__;s__
OTU_831	1	0	5	0.00%	k__Bacteria;p__Proteobacteria;c__Gammaproteobacteria;o__Cardiobacteriales;f__Wohlfahrtiimonadaceae;g__Koukoulia;s__Koukoulia_aurantiaca
OTU_832	4	1	3	0.00%	k__Bacteria;p__Actinobacteriota;c__Coriobacteriia;o__Coriobacteriales;f__Eggerthellaceae;g__Eggerthella;s__
OTU_833	2	4	1	0.00%	k__Bacteria;p__Bacteroidota;c__Bacteroidia;o__Chitinophagales;f__Saprospiraceae;g__Haliscomenobacter;s__

OTU_834	4	2	0	0.00%	k__Bacteria;p__Firmicutes;c__unidentified_Firmicutes;o__Oscillospirales;f__Ruminococcaceae;g__;s__
OTU_835	42	39	49	0.07%	k__Bacteria;p__Firmicutes;c__Bacilli;o__Lactobacillales;f__Streptococcaceae;g__Streptococcus;s__Streptococcus_sp_FF10
OTU_836	5	3	4	0.01%	k__Bacteria;p__Bdellovibrionota;c__Bdellovibrionia;o__Bacteriovoracales;f__Bacteriovoracaceae;g__Peredibacter
OTU_837	5	1	0	0.00%	k__Bacteria;p__Proteobacteria;c__Alphaproteobacteria;o__Rhizobiales;f__Stappiaceae;g__Agaricicola;s__Agaricicola_taiwanensis
OTU_838	1	0	5	0.00%	k__Bacteria;p__Firmicutes;c__Clostridia;o__Oscillospirales;f__Ruminococcaceae;g__[Eubacterium]_siraeum_group;s__
OTU_839	0	0	0	0.00%	k__Bacteria;p__Firmicutes;c__Clostridia;o__Clostridia_UCG-014;f__;g__;s__
OTU_840	1	0	1	0.00%	k__Bacteria;p__Proteobacteria;c__Gammaproteobacteria;o__Burkholderiales;f__Comamonadaceae;g__Rhizobacter;s__
OTU_841	3	0	0	0.00%	k__Bacteria;p__Proteobacteria;c__Gammaproteobacteria;o__Burkholderiales;f__Chitinimonadaceae;g__Chitinivorax;s__
OTU_842	2	0	1	0.00%	k__Bacteria;p__Proteobacteria;c__Gammaproteobacteria;o__Burkholderiales;f__Chitinibacteraceae;g__Formivibrio;s__
OTU_843	2	2	2	0.00%	k__Bacteria;p__Bacteroidota;c__Bacteroidia;o__Flavobacteriales;f__Flavobacteriaceae;g__Flavobacterium;s__Flavobacterium_columnare
OTU_844	5	3	2	0.01%	k__Bacteria;p__Desulfobacterota;c__Desulfobulbia;o__Desulfobulbales;f__Desulfobulbaceae;g__Desulfobulbus;s__
OTU_845	3	0	2	0.00%	k__Bacteria;p__Bacteroidota;c__Bacteroidia;o__Bacteroidales;f__Bacteroidaceae;g__Bacteroides;s__Bacteroides_nordii
OTU_846	0	0	0	0.00%	k__Bacteria;p__Actinobacteriota;c__Coriobacteriia;o__Coriobacteriales;f__Eggerthellaceae;g__Enterorhabdus;s__
OTU_847	0	2	1	0.00%	k__Bacteria;p__unidentified_Bacteria;c__Verrucomicrobiae;o__;f__;g__;s__
OTU_848	12	3	1	0.01%	k__Bacteria;p__Proteobacteria;c__Gammaproteobacteria;o__Pseudomonadales;f__Moraxellaceae;g__Acinetobacter;s__Acinetobacter_xiamenensis
OTU_849	0	1	2	0.00%	k__Bacteria;p__Bacteroidota;c__Bacteroidia;o__Bacteroidales;f__Muribaculaceae;g__;s__
OTU_850	4	9	4	0.01%	k__Bacteria;p__Bacteroidota;c__Bacteroidia;o__Cytophagales;f__Hymenobacteraceae;g__Hymenobacter
OTU_851	4	3	0	0.00%	k__Bacteria;p__Proteobacteria;c__Gammaproteobacteria;o__Burkholderiales;f__Alcaligenaceae;g__Parapusillimonas
OTU_852	0	0	0	0.00%	k__Bacteria;p__Firmicutes;c__Bacilli;o__Erysipelotrichales;f__Erysipelatoclostridiaceae;g__Candidatus_Stoquefichus;s__
OTU_853	2	0	0	0.00%	k__Bacteria;p__Proteobacteria;c__Gammaproteobacteria;o__Burkholderiales;f__Burkholderiaceae;g__Limnobacter;s__Limnobacter_thiooxidans
OTU_854	2	0	4	0.00%	k__Bacteria;p__Proteobacteria;c__Gammaproteobacteria;o__Burkholderiales;f__Chitinibacteraceae;g__Formivibrio;s__
OTU_855	2	3	2	0.00%	k__Bacteria;p__Gracilibacteria;c__unidentified_Gracilibacteria;o__unidentified_Gracilibacteria;f__unidentified_Gracilibacteria;g__unidentified_Gracilibacteria
OTU_856	2	2	3	0.00%	k__Bacteria
OTU_857	6	14	6	0.01%	k__Bacteria;p__Actinobacteria;c__unidentified_Actinobacteria;o__Frankiales;f__Geodermatophilaceae;g__Blastococcus
OTU_858	1	6	1	0.00%	k__Bacteria;p__unidentified_Bacteria;c__Bacteroidia;o__Bacteroidales;f__Dysgonomonadaceae;g__;s__
OTU_859	0	5	1	0.00%	k__Bacteria;p__Proteobacteria;c__Gammaproteobacteria;o__CHAB-XI-27;f__;g__;s__
OTU_860	0	1	0	0.00%	k__Bacteria;p__Firmicutes;c__Clostridia;o__Clostridia_vadinBB60_group;f__;g__;s__
OTU_861	0	0	5	0.00%	k__Bacteria;p__Spirochaetota;c__MVP-15;o__;f__;g__;s__
OTU_862	3	4	7	0.01%	k__Bacteria;p__Bacteroidota;c__Bacteroidia;o__Bacteroidales;f__Rikenellaceae;g__Bact-08
OTU_863	0	0	2	0.00%	k__Bacteria;p__Proteobacteria;c__Gammaproteobacteria;o__unidentified_Gammaproteobacteria;f__unidentified_Gammaproteobacteria;g__;s__
OTU_864	2	0	0	0.00%	k__Bacteria;p__unidentified_Bacteria;c__Alphaproteobacteria;o__Micavibrionales;f__;g__;s__
OTU_865	0	0	0	0.00%	k__Bacteria;p__Firmicutes;c__Bacilli;o__Erysipelotrichales;f__Erysipelotrichaceae;g__Solobacterium;s__Solobacterium_moorei
OTU_866	0	0	0	0.00%	k__Bacteria;p__Gracilibacteria;c__unidentified_Gracilibacteria;o__Absconditabacteriales_(SR1);f__unidentified_Absconditabacteriales_(SR1);<br>g__unidentified_Absconditabacteriales_(SR1);s__SR1_bacterium_oral_taxon_875
OTU_867	0	2	4	0.00%	k__Bacteria;p__Proteobacteria;c__Gammaproteobacteria;o__Enterobacterales;f__Aeromonadaceae;g__Tolumonas
OTU_868	2	0	5	0.00%	k__Bacteria
OTU_869	0	1	1	0.00%	k__Bacteria;p__Firmicutes;c__Clostridia;o__Christensenellales;f__Christensenellaceae;g__Christensenellaceae_R-7_group;s__
OTU_870	7	0	0	0.00%	k__Bacteria;p__Firmicutes;c__Clostridia;o__Oscillospirales;f__Hungateiclostridiaceae;g__Saccharofermentans
OTU_871	4	0	1	0.00%	k__Bacteria;p__Bacteroidota;c__Bacteroidia;o__Cytophagales;f__Cytophagaceae;g__Cytophaga;s__
OTU_872	0	1	2	0.00%	k__Bacteria
OTU_873	1	1	4	0.00%	k__Bacteria;p__Bacteroidota;c__Bacteroidia;o__Bacteroidales;f__Prevotellaceae;g__Prevotella
OTU_874	2	1	1	0.00%	k__Bacteria;p__Firmicutes;c__Clostridia;o__Peptostreptococcales-Tissierellales;f__Anaerovoracaceae;g__Family_XIII_AD3011_group;s__
OTU_875	0	3	2	0.00%	k__Bacteria;p__Proteobacteria;c__Alphaproteobacteria;o__Rickettsiales;f__Mitochondria
OTU_876	0	2	0	0.00%	k__Bacteria;p__Firmicutes;c__Bacilli;o__Lactobacillales
OTU_877	0	2	2	0.00%	k__Bacteria
OTU_878	3	3	1	0.00%	k__Bacteria;p__Proteobacteria;c__Gammaproteobacteria;o__Xanthomonadales;f__Rhodanobacteraceae;g__Dokdonella;s__
OTU_879	15	15	7	0.02%	k__Bacteria;p__Bacteroidota;c__Bacteroidia;o__Cytophagales;f__Hymenobacteraceae;g__Hymenobacter;s__
OTU_880	0	3	2	0.00%	k__Bacteria;p__Synergistota;c__Synergistia;o__Synergistales;f__Synergistaceae;g__Fretibacterium;s__
OTU_881	0	5	0	0.00%	k__Bacteria;p__Proteobacteria;c__Gammaproteobacteria;o__Pseudomonadales;f__Cellvibrionaceae;g__Cellvibrio;s__
OTU_882	0	2	0	0.00%	k__Bacteria;p__Firmicutes;c__Bacilli;o__Lactobacillales;f__Streptococcaceae;g__Streptococcus;s__Streptococcus_henryi
OTU_883	0	0	0	0.00%	k__Bacteria;p__Firmicutes;c__Clostridia;o__Peptostreptococcales-Tissierellales;f__Anaerovoracaceae;g__[Eubacterium]_nodatum_group
OTU_884	4	0	0	0.00%	k__Bacteria;p__Proteobacteria;c__Gammaproteobacteria;o__Pseudomonadales;f__Moraxellaceae;g__Cavicella;s__
OTU_885	11	0	0	0.01%	k__Bacteria;p__Firmicutes;c__Clostridia;o__Lachnospirales;f__Lachnospiraceae;g__A2;s__
OTU_886	0	3	7	0.01%	k__Bacteria;p__unidentified_Bacteria;c__Bacteroidia;o__Chitinophagales;f__Saprospiraceae;g__;s__
OTU_887	2	0	1	0.00%	k__Bacteria;p__Proteobacteria;c__Gammaproteobacteria;o__Burkholderiales;f__Chitinibacteraceae;g__Formivibrio;s__

OTU_888	0	0	0	0.00%	k__Bacteria;p__Proteobacteria;c__Gammaproteobacteria;o__Cardiobacteriales;f__Wohlfahrtiimonadaceae;g__Ignatzschineria
OTU_889	0	0	0	0.00%	k__Bacteria
OTU_890	2	0	0	0.00%	k__Bacteria;p__unidentified_Bacteria;c__Saccharimonadia;o__Saccharimonadales;f__unidentified_Saccharimonadales;g__unidentified_Saccharimonadales; s__TM7_phylum_sp_oral_clone_FR058
OTU_891	1	0	0	0.00%	k__Bacteria;p__unidentified_Bacteria;c__Gammaproteobacteria;o__Burkholderiales;f__Neisseriaceae;g__;s__
OTU_892	0	0	5	0.00%	k__Bacteria
OTU_893	0	0	1	0.00%	k__Bacteria;p__Proteobacteria;c__Gammaproteobacteria;o__Burkholderiales;f__Alcaligenaceae;g__GKS98_freshwater_group;s__
OTU_894	8	0	0	0.00%	k__Bacteria;p__Firmicutes;c__Clostridia;o__Peptostreptococcales-Tissierellales;f__Sedimentibacteraceae;g__Sedimentibacter;s__
OTU_895	6	0	5	0.01%	k__Bacteria;p__Fusobacteriota;c__Fusobacteriia;o__Fusobacteriales;f__Leptotrichiaceae;g__Leptotrichia;s__
OTU_896	1	1	1	0.00%	k__Bacteria;p__Cyanobacteria;c__Vampirivibrionia;o__Obscuribacterales;f__Obscuribacteraceae;g__Candidatus_Obscuribacter;s__
OTU_897	2	2	1	0.00%	k__Bacteria
OTU_898	1	2	1	0.00%	k__Bacteria;p__Bacteroidota;c__Bacteroidia;o__Sphingobacteriales;f__Lentimicrobiaceae;g__Lentimicrobium;s__
OTU_899	9	2	0	0.01%	k__Bacteria;p__Actinobacteria;c__unidentified_Actinobacteria;o__Corynebacteriales;f__Mycobacteriaceae;g__Mycobacterium
OTU_900	0	0	0	0.00%	k__Bacteria;p__unidentified_Bacteria;c__Clostridia;o__Lachnospirales;f__Lachnospiraceae;g__;s__
OTU_901	0	0	0	0.00%	k__Bacteria;p__Bacteroidota;c__Bacteroidia;o__Bacteroidales;f__Prevotellaceae;g__Prevotella_7
OTU_902	0	1	1	0.00%	k__Bacteria;p__Firmicutes;c__Clostridia;o__Peptostreptococcales-Tissierellales;f__Anaerovoracaceae;g__[Eubacterium]_nodatum_group;s__
OTU_903	1	5	0	0.00%	k__Bacteria;p__Firmicutes;c__Clostridia;o__Lachnospirales;f__Lachnospiraceae;g__Lachnospiraceae_NK4A136_group;s__
OTU_904	0	5	0	0.00%	k__Bacteria;p__Firmicutes;c__Clostridia;o__Eubacteriales;f__Garciellaceae;g__Irregularibacter
OTU_905	0	1	7	0.00%	k__Bacteria;p__Proteobacteria;c__Gammaproteobacteria;o__Pseudomonadales;f__Moraxellaceae;g__[Agitococcus]_lubricus_group;s__
OTU_906	20	20	19	0.03%	k__Bacteria;p__Bacteroidota;c__Bacteroidia;o__Bacteroidales;f__Bacteroidaceae;g__Bacteroides;s__Bacteroides_caccae
OTU_907	0	1	2	0.00%	k__Bacteria;p__unidentified_Bacteria;c__Bacteroidia;o__Chitinophagales;f__Saprospiraceae
OTU_908	3	0	1	0.00%	k__Bacteria;p__Bacteroidota;c__Bacteroidia;o__Sphingobacteriales;f__NS11-12_marine_group;g__unidentified_NS11-12_marine_group;s__Bacteroidetes_bacterium_UKL13-3
OTU_909	3	0	0	0.00%	k__Bacteria;p__Proteobacteria;c__Gammaproteobacteria;o__Burkholderiales;f__Neisseriaceae
OTU_910	8	8	12	0.01%	k__Bacteria;p__Firmicutes;c__Clostridia;o__Clostridia_UCG-014;f__;g__;s__
OTU_911	0	0	5	0.00%	k__Bacteria
OTU_912	0	0	0	0.00%	k__Bacteria;p__Firmicutes;c__Clostridia;o__Monoglobales;f__Monoglobaceae;g__Monoglobus;s__
OTU_913	2	1	7	0.01%	k__Bacteria;p__Firmicutes;c__Clostridia;o__Oscillospirales;f__Ruminococcaceae;g__Negativibacillus;s__
OTU_914	1	3	0	0.00%	k__Bacteria;p__Firmicutes;c__Clostridia;o__Oscillospirales;f__Oscillospiraceae;g__NK4A214_group;s__
OTU_915	1	0	0	0.00%	k__Bacteria;p__Proteobacteria;c__Gammaproteobacteria;o__Burkholderiales;f__Sutterellaceae;g__Sutterella;s__Sutterellaceae_bacterium_Marseille-P2968
OTU_916	0	0	0	0.00%	k__Bacteria;p__Firmicutes;c__Clostridia;o__Peptostreptococcales-Tissierellales;f__Anaerovoracaceae;g__Family_XIII_UCG-001;s__Eubacterium_sp_oral_clone_CK047
OTU_917	0	1	1	0.00%	k__Bacteria;p__Proteobacteria;c__Gammaproteobacteria;o__Burkholderiales;f__Oxalobacteraceae
OTU_918	1	0	0	0.00%	k__Bacteria;p__unidentified_Bacteria;c__Bacteroidia;o__Chitinophagales;f__;g__;s__
OTU_919	0	6	0	0.00%	k__Bacteria;p__Proteobacteria;c__Gammaproteobacteria;o__Pseudomonadales;f__Alcanivoracaceae;g__Ketobacter
OTU_920	0	3	2	0.00%	k__Bacteria;p__Bacteroidota;c__Bacteroidia;o__Bacteroidales;f__Dysgonomonadaceae;g__Petrimonas;s__
OTU_921	0	0	0	0.00%	k__Bacteria;p__Actinobacteriota;c__Coriobacteriia;o__Coriobacteriales;f__Eggerthellaceae;g__Enterorhabdus;s__
OTU_922	2	5	0	0.00%	k__Bacteria;p__Proteobacteria;c__Alphaproteobacteria;o__Rhizobiales;f__Xanthobacteraceae;g__Rhodopseudomonas;s__Rhodopseudomonas_palustris
OTU_923	6	0	0	0.00%	k__Bacteria;p__Proteobacteria;c__Gammaproteobacteria;o__Xanthomonadales;f__Rhodanobacteraceae;g__Dokdonella;s__
OTU_924	10	0	1	0.01%	k__Bacteria;p__Actinobacteria;c__unidentified_Actinobacteria;o__Streptomycetales;f__Streptomycetaceae;g__Streptomyces;s__Streptomyces_scabiei
OTU_925	2	4	2	0.00%	k__Bacteria;p__Firmicutes;c__Bacilli;o__Bacillales;f__Planococcaceae;g__Solibacillus;s__Solibacillus_silvestris
OTU_926	0	3	3	0.00%	k__Bacteria;p__Proteobacteria;c__Gammaproteobacteria;o__Methylococcales;f__Methylomonadaceae
OTU_927	0	0	0	0.00%	k__Bacteria;p__Bacteroidota;c__Bacteroidia;o__Bacteroidales;f__Prevotellaceae;g__Prevotellaceae_UCG-001;s__
OTU_928	6	4	0	0.01%	k__Bacteria
OTU_929	0	0	0	0.00%	k__Bacteria;p__Bacteroidota;c__Bacteroidia;o__Flavobacteriales;f__Flavobacteriaceae
OTU_930	0	0	0	0.00%	k__Bacteria;p__Firmicutes;c__Bacilli;o__Lactobacillales;f__Lactobacillaceae;g__Bombilactobacillus;s__
OTU_931	0	4	0	0.00%	k__Bacteria;p__Bacteroidota;c__Bacteroidia;o__Bacteroidales;f__Dysgonomonadaceae;g__Dysgonomonas;s__
OTU_932	1	0	1	0.00%	k__Bacteria;p__unidentified_Bacteria;c__Clostridia;o__Lachnospirales;f__Lachnospiraceae
OTU_933	0	1	2	0.00%	k__Bacteria
OTU_934	0	2	1	0.00%	k__Bacteria;p__Firmicutes;c__Bacilli;o__Lactobacillales;f__Lactobacillaceae;g__Limosilactobacillus;s__Lactobacillus_fermentum
OTU_935	1	1	2	0.00%	k__Bacteria;p__Firmicutes;c__Clostridia;o__Lachnospirales;f__Lachnospiraceae;g__Blautia;s__
OTU_936	0	0	0	0.00%	k__Bacteria;p__Firmicutes;c__Negativicutes;o__Veillonellales-Selenomonadales;f__Veillonellaceae;g__Dialister;s__Dialister_pneumosintes
OTU_937	4	2	5	0.01%	k__Bacteria;p__Proteobacteria;c__Gammaproteobacteria;o__Enterobacterales
OTU_938	1	0	1	0.00%	k__Bacteria;p__Proteobacteria;c__Gammaproteobacteria;o__Burkholderiales;f__Methylophilaceae;g__MM1;s__
OTU_939	0	0	0	0.00%	k__Bacteria;p__Proteobacteria;c__Alphaproteobacteria;o__Paracaedibacterales;f__Paracaedibacteraceae;g__Candidatus_Paracaedibacter
OTU_940	0	1	0	0.00%	k__Bacteria;p__Firmicutes;c__Bacilli;o__Lactobacillales;f__Listeriaceae;g__Brochothrix;s__Brochothrix_thermosphacta
OTU_941	2	1	2	0.00%	k__Bacteria;p__Proteobacteria;c__Gammaproteobacteria;o__Pseudomonadales;f__Halomonadaceae;g__Marinospirillum;s__Marinospirillum_minutulum

OTU_942	1	3	0	0.00%	k__Bacteria;p__Firmicutes;c__Clostridia;o__Lachnospirales;f__Lachnospiraceae;g__Moryella;s__
OTU_943	1	1	0	0.00%	k__Bacteria;p__Sumerlaeota;c__Sumerlaeia;o__Sumerlaeales;f__Sumerlaeaceae;g__Sumerlaea;s__
OTU_944	0	0	0	0.00%	k__Bacteria
OTU_945	2	0	3	0.00%	k__Bacteria
OTU_946	4	1	0	0.00%	k__Bacteria;p__Firmicutes;c__Clostridia;o__Oscillospirales;f__Ruminococcaceae;g__Negativibacillus;s__
OTU_947	5	0	0	0.00%	k__Bacteria;p__Firmicutes;c__Clostridia;o__Peptostreptococcales-Tissierellales;f__unidentified_Peptostreptococcales-Tissierellales;g__Gottschalkia;s__
OTU_948	0	5	0	0.00%	k__Bacteria
OTU_949	0	4	0	0.00%	k__Bacteria;p__Firmicutes;c__Thermoanaerobacteria;o__Thermoanaerobacterales;f__SRB2;g__;s__
OTU_950	2	1	2	0.00%	k__Bacteria
OTU_951	1	4	4	0.00%	k__Bacteria;p__Proteobacteria;c__Gammaproteobacteria;o__Pseudomonadales;f__Halomonadaceae;g__Halomonas;s__Pseudomonas_sp_TN12
OTU_952	2	3	0	0.00%	k__Bacteria;p__Gemmatimonadota
OTU_953	10	2	12	0.01%	k__Bacteria;p__Proteobacteria;c__Gammaproteobacteria;o__Burkholderiales;f__Comamonadaceae
OTU_954	0	1	0	0.00%	k__Bacteria;p__Firmicutes;c__Clostridia;o__Oscillospirales;f__Oscillospiraceae;g__Colidextribacter;s__Colidextribacter_massiliensis
OTU_955	2	0	1	0.00%	k__Bacteria;p__Firmicutes;c__Clostridia;o__Lachnospirales;f__Lachnospiraceae;g__Lachnospiraceae_NK3A20_group
OTU_956	0	4	0	0.00%	k__Bacteria
OTU_957	4	0	0	0.00%	k__Bacteria;p__unidentified_Bacteria;c__Clostridia;o__Oscillospirales;f__Ruminococcaceae;g__;s__
OTU_958	0	2	5	0.00%	k__Bacteria;p__Spirochaetota;c__Leptospirae;o__Leptospirales;f__Leptospiraceae;g__RBG-16-49-21;s__
OTU_959	0	0	4	0.00%	k__Bacteria;p__unidentified_Bacteria;c__Alphaproteobacteria;o__Rickettsiales;f__Rickettsiaceae;g__;s__
OTU_960	2	1	1	0.00%	k__Bacteria;p__Fusobacteriota;c__Fusobacteriia;o__Fusobacteriales;f__Leptotrichiaceae;g__Sebaldella;s__
OTU_961	0	1	0	0.00%	k__Bacteria;p__Actinobacteriota;c__Coriobacteriia;o__Coriobacteriales;f__Atopobiaceae;g__Atopobium;s__
OTU_962	3	1	0	0.00%	k__Bacteria;p__Bacteroidota;c__Bacteroidia;o__Sphingobacteriales
OTU_963	0	0	0	0.00%	k__Bacteria;p__Actinobacteria;c__unidentified_Actinobacteria;o__Micrococcales;f__Micrococcaceae;g__Rothia;s__Rothia_mucilaginosa
OTU_964	0	3	0	0.00%	k__Bacteria;p__Firmicutes;c__Clostridia;o__Oscillospirales;f__Ruminococcaceae
OTU_965	2	1	5	0.00%	k__Bacteria;p__Proteobacteria;c__Gammaproteobacteria;o__Enterobacterales;f__Morganellaceae;g__Morganella;s__Morganella_morganii
OTU_966	1	0	4	0.00%	k__Bacteria;p__Proteobacteria;c__Gammaproteobacteria
OTU_967	0	1	0	0.00%	k__Bacteria;p__Firmicutes;c__Bacilli;o__Staphylococcales;f__Gemellaceae;g__Gemella;s__
OTU_968	2	1	6	0.00%	k__Bacteria;p__Proteobacteria;c__Gammaproteobacteria;o__Xanthomonadales;f__Rhodanobacteraceae;g__Ahniella;s__
OTU_969	1	0	0	0.00%	k__Bacteria;p__Bacteroidota;c__Bacteroidia;o__Chitinophagales;f__Chitinophagaceae
OTU_970	5	0	2	0.00%	k__Bacteria;p__Bacteroidota;c__Bacteroidia;o__Bacteroidales;f__Dysgonomonadaceae;g__Proteiniphilum;s__
OTU_971	5	1	0	0.00%	k__Bacteria;p__Gemmatimonadetes;c__unidentified_Gemmatimonadetes;o__Gemmatimonadales;f__Gemmatimonadaceae;g__Gemmatimonas;s__
OTU_972	1	2	3	0.00%	k__Bacteria
OTU_973	1	3	1	0.00%	k__Bacteria;p__unidentified_Bacteria;c__Alphaproteobacteria;o__Rhodospirillales;f__;g__;s__
OTU_974	4	0	0	0.00%	k__Bacteria;p__Actinobacteriota;c__Acidimicrobiia;o__IMCC26256;f__;g__;s__
OTU_975	5	7	6	0.01%	k__Bacteria;p__Firmicutes;c__Bacilli;o__Lactobacillales;f__Lactobacillaceae;g__Lacticaseibacillus
OTU_976	5	2	0	0.00%	k__Bacteria;p__Firmicutes;c__Bacilli;o__Erysipelotrichales;f__Erysipelatoclostridiaceae;g__Erysipelatoclostridium
OTU_977	0	4	0	0.00%	k__Bacteria;p__Firmicutes
OTU_978	3	1	0	0.00%	k__Bacteria;p__Bacteroidota;c__Bacteroidia
OTU_979	0	0	4	0.00%	k__Bacteria;p__Spirochaetota;c__MVP-15
OTU_980	7	1	3	0.01%	k__Bacteria;p__Proteobacteria;c__Gammaproteobacteria;o__Xanthomonadales;f__Xanthomonadaceae;g__Stenotrophomonas
OTU_981	1	2	3	0.00%	k__Bacteria;p__Proteobacteria;c__Gammaproteobacteria;o__Xanthomonadales;f__Xanthomonadaceae;g__Pseudoxanthomonas;s__
OTU_982	0	4	0	0.00%	k__Bacteria;p__Actinobacteria;c__unidentified_Actinobacteria;o__Micrococcales;f__Beutenbergiaceae;g__Salana;s__
OTU_983	72	48	45	0.08%	k__Bacteria;p__Proteobacteria;c__Gammaproteobacteria;o__Burkholderiales;f__Oxalobacteraceae;g__Massilia
OTU_984	4	0	1	0.00%	k__Bacteria;p__Fusobacteriota;c__Fusobacteriia;o__Fusobacteriales;f__Fusobacteriaceae
OTU_985	1	0	3	0.00%	k__Bacteria;p__Firmicutes;c__Clostridia;o__Oscillospirales;f__[Eubacterium]_coprostanoligenes_group;g__;s__
OTU_986	0	6	1	0.00%	k__Bacteria;p__Firmicutes;c__Bacilli;o__Lactobacillales;f__Lactobacillaceae;g__Lacticaseibacillus
OTU_987	1	2	1	0.00%	k__Bacteria;p__unidentified_Bacteria;c__unidentified_Bacteria;o__Actinomycetales;f__Actinomycetaceae;g__;s__
OTU_988	0	4	0	0.00%	k__Bacteria;p__Firmicutes;c__Bacilli;o__Lactobacillales;f__Lactobacillaceae;g__Weissella;s__Weissella_ceti
OTU_989	2	2	2	0.00%	k__Bacteria;p__Gracilibacteria;c__unidentified_Gracilibacteria;o__JGI_0000069-P22;f__;g__;s__
OTU_990	0	0	0	0.00%	k__Bacteria;p__Actinobacteria;c__unidentified_Actinobacteria;o__Actinomycetales;f__Actinomycetaceae;g__Actinomyces;s__Schaalia_odontolytica
OTU_991	1	0	4	0.00%	k__Bacteria;p__Acidobacteriota;c__Blastocatellia;o__11-24;f__;g__;s__
OTU_992	1	3	1	0.00%	k__Bacteria;p__Proteobacteria;c__Alphaproteobacteria;o__Caulobacterales;f__Caulobacteraceae
OTU_993	5	0	0	0.00%	k__Bacteria;p__Bacteroidota;c__Bacteroidia;o__Flavobacteriales;f__Flavobacteriaceae;g__Salegentibacter;s__
OTU_994	0	0	4	0.00%	k__Bacteria;p__unidentified_Bacteria;c__Bacteroidia;o__Bacteroidales;f__Barnesiellaceae;g__;s__
OTU_995	1	1	0	0.00%	k__Bacteria;p__unidentified_Bacteria;c__Saccharimonadia;o__Saccharimonadales;f__Saccharimonadaceae;g__TM7a;s__
OTU_996	1	0	1	0.00%	k__Bacteria;p__Proteobacteria;c__Gammaproteobacteria;o__unidentified_Gammaproteobacteria;f__unidentified_Gammaproteobacteria;g__;s__
OTU_997	0	0	0	0.00%	k__Bacteria;p__Firmicutes;c__Clostridia;o__Christensenellales;f__Christensenellaceae;g__Christensenellaceae_R-7_group;s__

OTU_998	0	4	0	0.00%	k__Bacteria;p__Proteobacteria;c__Gammaproteobacteria;o__Burkholderiales;f__Burkholderiaceae;g__Limnobacter;s__
OTU_999	0	0	1	0.00%	k__Bacteria;p__Spirochaetota;c__Leptospirae;o__Leptospirales;f__Leptospiraceae;g__;s__
OTU_1000	3	0	0	0.00%	k__Bacteria
OTU_1001	0	1	4	0.00%	k__Bacteria;p__Firmicutes;c__Clostridia;o__Christensenellales;f__Christensenellaceae;g__Christensenellaceae_R-7_group
OTU_1002	0	0	1	0.00%	k__Bacteria;p__Firmicutes;c__Negativicutes;o__Veillonellales-Selenomonadales;f__Sporomusaceae;g__Anaeromusa-Anaeroarcus;s__Sporomusaceae_bacterium
OTU_1003	3	0	3	0.00%	k__Bacteria;p__Gracilibacteria;c__unidentified_Gracilibacteria;o__JGI_0000069-P22;f__;g__;s__
OTU_1004	2	3	0	0.00%	k__Bacteria;p__Actinobacteria;c__unidentified_Actinobacteria;o__Actinomycetales;f__Actinomycetaceae;g__Actinomyces;s__Actinomyces_graevenitzii
OTU_1005	0	3	0	0.00%	k__Bacteria;p__Deinococcota;c__Deinococci;o__Deinococcales;f__Deinococcaceae;g__Deinococcus;s__
OTU_1006	0	0	0	0.00%	k__Bacteria;p__Actinobacteriota;c__Coriobacteriia;o__Coriobacteriales;f__Eggerthellaceae
OTU_1007	3	0	0	0.00%	k__Bacteria;p__Verrucomicrobiota;c__Verrucomicrobiae;o__Verrucomicrobiales;f__Verrucomicrobiaceae;g__Prosthecobacter;s__
OTU_1008	0	0	3	0.00%	k__Bacteria;p__Proteobacteria;c__Gammaproteobacteria;o__Xanthomonadales;f__Xanthomonadaceae;g__Pseudoxanthomonas;s__Pseudoxanthomonas_mexicana
OTU_1009	1	0	0	0.00%	k__Bacteria;p__Proteobacteria;c__Gammaproteobacteria;o__Methylococcales;f__Methylomonadaceae
OTU_1010	0	2	1	0.00%	k__Bacteria;p__unidentified_Bacteria;c__Gammaproteobacteria;o__Burkholderiales;f__Neisseriaceae;g__unidentified_Neisseriaceae; s__bacterium_enrichment_culture_clone_B214
OTU_1011	0	4	0	0.00%	k__Bacteria;p__Firmicutes;c__Clostridia;o__Peptostreptococcales-Tissierellales;f__Family_XI;g__Peptoniphilus
OTU_1012	0	7	5	0.01%	k__Bacteria;p__unidentified_Bacteria;c__Negativicutes;o__Acidaminococcales;f__Acidaminococcaceae;g__;s__
OTU_1013	4	0	0	0.00%	k__Bacteria;p__Proteobacteria;c__Alphaproteobacteria;o__Rhizobiales;f__unidentified_Rhizobiales;g__Phreatobacter;s__
OTU_1014	3	12	1	0.01%	k__Bacteria;p__Firmicutes;c__Bacilli;o__Bacillales;f__Bacillaceae;g__Bacillus
OTU_1015	3	7	2	0.01%	k__Bacteria;p__Firmicutes;c__Bacilli;o__Lactobacillales;f__Lactobacillaceae;g__Lactobacillus;s__Lactobacillus_delbrueckii
OTU_1016	0	0	3	0.00%	k__Bacteria;p__unidentified_Bacteria;c__Campylobacteria;o__Campylobacterales;f__;g__;s__
OTU_1017	1	0	0	0.00%	k__Bacteria;p__Proteobacteria;c__Gammaproteobacteria;o__Burkholderiales;f__Neisseriaceae
OTU_1018	0	0	0	0.00%	k__Bacteria;p__unidentified_Bacteria;c__Alphaproteobacteria;o__Rickettsiales;f__Rickettsiaceae;g__;s__
OTU_1019	0	1	1	0.00%	k__Bacteria;p__Proteobacteria;c__Gammaproteobacteria;o__Burkholderiales;f__Nitrosomonadaceae;g__MND1;s__
OTU_1020	0	0	1	0.00%	k__Bacteria;p__Cyanobacteria;c__Cyanobacteriia;o__Chloroplast;f__unidentified_Chloroplast;g__unidentified_Chloroplast
OTU_1021	3	0	3	0.00%	k__Bacteria;p__Proteobacteria;c__Gammaproteobacteria;o__Burkholderiales;f__Rhodocyclaceae
OTU_1022	0	0	5	0.00%	k__Bacteria;p__Firmicutes;c__Clostridia;o__Oscillospirales;f__Hungateiclostridiaceae;g__HN-HF0106;s__
OTU_1023	0	0	0	0.00%	k__Bacteria;p__unidentified_Bacteria;c__Bacilli;o__Erysipelotrichales;f__Erysipelotrichaceae;g__;s__
OTU_1024	0	4	0	0.00%	k__Bacteria;p__Firmicutes;c__Clostridia;o__Lachnospirales;f__Lachnospiraceae
OTU_1025	2	1	0	0.00%	k__Bacteria;p__Firmicutes;c__Negativicutes;o__Acidaminococcales;f__Acidaminococcaceae;g__Acidaminococcus
OTU_1026	4	2	0	0.00%	k__Bacteria;p__unidentified_Bacteria;c__Gammaproteobacteria;o__Salinisphaerales;f__Solimonadaceae
OTU_1027	4	0	0	0.00%	k__Bacteria;p__Bacteroidota;c__Bacteroidia;o__Chitinophagales;f__Chitinophagaceae;g__Flavisolibacter;s__
OTU_1028	1	5	0	0.00%	k__Bacteria;p__unidentified_Bacteria;c__Negativicutes;o__Acidaminococcales;f__Acidaminococcaceae;g__;s__
OTU_1029	4	0	0	0.00%	k__Bacteria;p__Cyanobacteria;c__Cyanobacteriia;o__unidentified_Cyanobacteriia;f__unidentified_Cyanobacteriia;g__Leptolyngbya_EcFYyyy-00;s__
OTU_1030	0	3	3	0.00%	k__Bacteria;p__Bacteroidota;c__Bacteroidia;o__Flavobacteriales;f__Flavobacteriaceae;g__Flavobacterium;s__Flavobacterium_cauense
OTU_1031	1	0	1	0.00%	k__Bacteria;p__Proteobacteria;c__Gammaproteobacteria;o__Enterobacterales;f__Yersiniaceae;g__Rahnella1;s__Rahnella_aquatilis
OTU_1032	0	2	0	0.00%	k__Bacteria
OTU_1033	0	1	1	0.00%	k__Bacteria;p__Proteobacteria;c__Alphaproteobacteria;o__Paracaedibacterales;f__Paracaedibacteraceae;g__Candidatus_Paracaedibacter;s__Candidatus_Paracaedibacter_symbiosus
OTU_1034	3	0	3	0.00%	k__Bacteria;p__Proteobacteria;c__Gammaproteobacteria;o__Cardiobacteriales;f__Wohlfahrtiimonadaceae;g__Ignatzschineria;s__
OTU_1035	0	0	4	0.00%	k__Bacteria;p__Bacteroidota;c__Bacteroidia;o__Bacteroidales;f__Dysgonomonadaceae;g__Proteiniphilum;s__
OTU_1036	0	0	1	0.00%	k__Bacteria;p__unidentified_Bacteria;c__Bacteroidia;o__Bacteroidales;f__Prevotellaceae;g__;s__
OTU_1037	5	1	0	0.00%	k__Bacteria;p__unidentified_Bacteria;c__Bacteroidia;o__Chitinophagales;f__Saprospiraceae;g__;s__
OTU_1038	0	4	0	0.00%	k__Bacteria;p__Firmicutes;c__Clostridia;o__Oscillospirales;f__Hungateiclostridiaceae;g__Saccharofermentans
OTU_1039	0	2	1	0.00%	k__Bacteria;p__unidentified_Bacteria;c__Alphaproteobacteria;o__Reyranellales;f__Reyranellaceae;g__;s__
OTU_1040	3	1	0	0.00%	k__Bacteria;p__Campylobacterota;c__Campylobacteria;o__Campylobacterales
OTU_1041	2	0	0	0.00%	k__Bacteria;p__Bacteroidota;c__Bacteroidia;o__Sphingobacteriales;f__FFCH9454;g__;s__
OTU_1042	0	3	0	0.00%	k__Bacteria;p__Proteobacteria;c__Gammaproteobacteria;o__Halothiobacillales;f__Halothiobacillaceae;g__Halothiobacillus;s__
OTU_1043	3	0	1	0.00%	k__Bacteria;p__Proteobacteria;c__Gammaproteobacteria;o__Burkholderiales;f__Rhodocyclaceae
OTU_1044	6	1	3	0.01%	k__Bacteria;p__Bacteroidota;c__Bacteroidia;o__Bacteroidales;f__Williamwhitmaniaceae;g__Williamwhitmania;s__
OTU_1045	1	1	2	0.00%	k__Bacteria;p__Proteobacteria;c__Gammaproteobacteria
OTU_1046	0	0	1	0.00%	k__Bacteria;p__Firmicutes;c__Clostridia;o__Peptostreptococcales-Tissierellales;f__Anaerovoracaceae;g__Family_XIII_AD3011_group;s__
OTU_1047	0	1	1	0.00%	k__Bacteria;p__unidentified_Bacteria;c__Gammaproteobacteria;o__Burkholderiales;f__Neisseriaceae
OTU_1048	0	4	3	0.00%	k__Bacteria;p__unidentified_Bacteria;c__Gammaproteobacteria;o__Burkholderiales;f__Alcaligenaceae;g__;s__
OTU_1049	0	0	1	0.00%	k__Bacteria;p__Bacteroidota;c__Bacteroidia;o__Bacteroidales;f__Marinifilaceae;g__Butyricimonas;s__
OTU_1050	10	7	11	0.01%	k__Bacteria;p__Firmicutes;c__Clostridia;o__Lachnospirales;f__Lachnospiraceae;g__Anaerostipes;s__

OTU_1051	1	1	3	0.00%	k__Bacteria;p__Firmicutes;c__Negativicutes;o__Veillonellales-Selenomonadales;f__Selenomonadaceae;g__Mitsuokella;s__
OTU_1052	3	0	1	0.00%	k__Bacteria;p__Bacteroidota;c__Bacteroidia;o__Bacteroidales;f__Prevotellaceae;g__Alloprevotella;s__
OTU_1053	3	0	1	0.00%	k__Bacteria;p__Firmicutes;c__Negativicutes;o__Veillonellales-Selenomonadales;f__Veillonellaceae;g__Allisonella;s__
OTU_1054	0	0	0	0.00%	k__Bacteria;p__Firmicutes;c__Negativicutes;o__Veillonellales-Selenomonadales;f__Veillonellaceae;g__Veillonella
OTU_1055	2	1	1	0.00%	k__Bacteria
OTU_1056	1	5	0	0.00%	k__Bacteria;p__Actinobacteria;c__unidentified_Actinobacteria;o__Streptosporangiales;f__Thermomonosporaceae;g__Thermobispora;s__Thermobispora_bispora
OTU_1057	0	0	0	0.00%	k__Bacteria;p__unidentified_Bacteria;c__Bacteroidia;o__Cytophagales;f__Spirosomaceae;g__;s__
OTU_1058	2	2	1	0.00%	k__Bacteria;p__Proteobacteria;c__Alphaproteobacteria;o__Rickettsiales;f__Rickettsiaceae;g__Candidatus_Megaira;s__
OTU_1059	3	6	1	0.01%	k__Bacteria;p__Firmicutes;c__Clostridia;o__Lachnospirales;f__Lachnospiraceae;g__Lachnospiraceae_UCG-006
OTU_1060	0	0	0	0.00%	k__Bacteria;p__unidentified_Bacteria;c__Alphaproteobacteria;o__Rhodospirillales;f__unidentified_Rhodospirillales;g__unidentified_Rhodospirillales;s__Azospirillum_sp_47_25
OTU_1061	1	0	0	0.00%	k__Bacteria;p__Proteobacteria;c__Alphaproteobacteria;o__Rhodospirillales;f__Magnetospirillaceae;g__Magnetospirillum;s__
OTU_1062	0	0	0	0.00%	k__Bacteria;p__Gracilibacteria;c__unidentified_Gracilibacteria;o__unidentified_Gracilibacteria;f__unidentified_Gracilibacteria;g__unidentified_Gracilibacteria
OTU_1063	3	0	0	0.00%	k__Bacteria;p__Firmicutes;c__Clostridia;o__Christensenellales;f__Christensenellaceae;g__Christensenellaceae_R-7_group
OTU_1064	4	0	0	0.00%	k__Bacteria;p__Bacteroidota;c__Bacteroidia;o__Chitinophagales;f__Chitinophagaceae;g__Flaviaesturariibacter;s__
OTU_1065	22	20	4	0.02%	k__Bacteria;p__Proteobacteria;c__Alphaproteobacteria;o__Sphingomonadales;f__Sphingomonadaceae;g__Sphingomonas
OTU_1066	0	1	0	0.00%	k__Bacteria;p__Myxococcota;c__Polyangia;o__Polyangiales;f__Polyangiaceae;g__Polyangium;s__
OTU_1067	0	0	0	0.00%	k__Bacteria;p__Bacteroidota;c__Bacteroidia;o__Bacteroidales;f__Bacteroidaceae;g__Bacteroides;s__Bacteroides_barnesiae
OTU_1068	0	1	0	0.00%	k__Bacteria;p__unidentified_Bacteria;c__Saccharimonadia;o__Saccharimonadales
OTU_1069	0	0	0	0.00%	k__Bacteria;p__Actinobacteriota;c__Coriobacteriia;o__Coriobacteriales;f__Eggerthellaceae;g__Enterorhabdus;s__
OTU_1070	5	2	2	0.00%	k__Bacteria;p__Proteobacteria;c__Alphaproteobacteria;o__Rhodobacterales;f__Rhodobacteraceae;g__Rubellimicrobium;s__
OTU_1071	2	3	0	0.00%	k__Bacteria;p__Firmicutes;c__Clostridia;o__Monoglobales;f__Monoglobaceae;g__Monoglobus;s__
OTU_1072	1	2	2	0.00%	k__Bacteria;p__Bacteroidota;c__Bacteroidia;o__Bacteroidales;f__Marinifilaceae;g__Butyricimonas
OTU_1073	1	1	1	0.00%	k__Bacteria;p__Bacteroidota;c__Bacteroidia;o__Flavobacteriales;f__Flavobacteriaceae;g__Flavobacterium;s__Flavobacterium_suncheonense
OTU_1074	1	0	0	0.00%	k__Bacteria;p__Chloroflexi;c__Anaerolineae;o__RBG-13-54-9;f__;g__;s__
OTU_1075	0	0	3	0.00%	k__Bacteria;p__Cyanobacteria;c__Cyanobacteriia;o__Cyanobacteriales;f__Coleofasciculaceae;g__Microcoleus_PCC-7113;s__Microcoleus_sp_PCC_7113
OTU_1076	0	0	3	0.00%	k__Bacteria;p__Spirochaetota;c__Spirochaetia;o__Spirochaetales;f__Spirochaetaceae;g__Treponema
OTU_1077	0	0	2	0.00%	k__Bacteria;p__Desulfobacterota;c__Desulfovibrionia;o__Desulfovibrionales;f__Desulfovibrionaceae;g__Desulfovibrio;s__
OTU_1078	0	3	0	0.00%	k__Bacteria
OTU_1079	230	251	230	0.36%	k__Bacteria;p__Proteobacteria;c__Gammaproteobacteria;o__Enterobacterales;f__Aeromonadaceae;g__Tolumonas;s__
OTU_1080	3	0	0	0.00%	k__Bacteria;p__Proteobacteria;c__Gammaproteobacteria;o__Pseudomonadales;f__Hahellaceae;g__Hahella;s__
OTU_1081	0	0	0	0.00%	k__Bacteria;p__Bacteroidota;c__Bacteroidia;o__Bacteroidales;f__Muribaculaceae;g__;s__
OTU_1082	1	0	2	0.00%	k__Bacteria;p__Kapabacteria;c__unidentified_Kapabacteria;o__Kapabacteriales
OTU_1083	5	1	0	0.00%	k__Bacteria;p__Desulfobacterota;c__Desulfovibrionia;o__Desulfovibrionales;f__Desulfovibrionaceae;g__Desulfovibrio
OTU_1084	0	2	0	0.00%	k__Bacteria;p__Proteobacteria;c__Gammaproteobacteria;o__Pseudomonadales;f__Moraxellaceae;g__Alkanindiges;s__
OTU_1085	3	2	1	0.00%	k__Bacteria;p__Firmicutes;c__Clostridia;o__Peptostreptococcales-Tissierellales;f__Family_XI;g__Tissierella
OTU_1086	0	0	3	0.00%	k__Bacteria;p__Firmicutes;c__Clostridia;o__Oscillospirales;f__Hungateiclostridiaceae;g__Ruminiclostridium;s__Ruminiclostridium_hungatei
OTU_1087	0	3	0	0.00%	k__Bacteria;p__Proteobacteria;c__Gammaproteobacteria;o__Methylococcales;f__Methylomonadaceae;g__Methylovulum;s__
OTU_1088	3	2	1	0.00%	k__Bacteria;p__Proteobacteria;c__Gammaproteobacteria;o__Pseudomonadales;f__Pseudomonadaceae;g__Pseudomonas
OTU_1089	1	1	0	0.00%	k__Bacteria;p__Bacteroidota;c__Bacteroidia;o__Chitinophagales;f__Chitinophagaceae;g__Taibaiella;s__
OTU_1090	0	2	1	0.00%	k__Bacteria;p__Bacteroidota;c__Bacteroidia;o__Bacteroidales;f__Rikenellaceae;g__DMER64
OTU_1091	7	4	6	0.01%	k__Bacteria;p__Proteobacteria;c__Gammaproteobacteria;o__Burkholderiales;f__Methylophilaceae;g__Methylotenera;s__
OTU_1092	0	0	1	0.00%	k__Bacteria;p__Proteobacteria;c__Alphaproteobacteria;o__Rhizobiales;f__Beijerinckiaceae;g__Microvirga;s__Microvirga_sp
OTU_1093	0	0	4	0.00%	k__Bacteria;p__Proteobacteria;c__Alphaproteobacteria;o__Paracaedibacterales;f__Paracaedibacteraceae;g__Candidatus_Paracaedibacter;s__
OTU_1094	1	0	1	0.00%	k__Bacteria;p__Gracilibacteria;c__;o__;f__;g__;s__
OTU_1095	0	3	0	0.00%	k__Bacteria;p__Bacteroidota;c__Bacteroidia;o__Bacteroidales
OTU_1096	0	1	1	0.00%	k__Bacteria;p__Proteobacteria;c__Gammaproteobacteria;o__Salinisphaerales;f__Solimonadaceae;g__Hydrocarboniphaga;s__Hydrocarboniphaga_daqingensis
OTU_1097	0	1	2	0.00%	k__Bacteria;p__unidentified_Bacteria;c__Alphaproteobacteria;o__Micavibrionales;f__unidentified_Micavibrionales;g__unidentified_ Micavibrionales;s__bacterium_enrichment_culture_clone_SRAO_28
OTU_1098	0	3	1	0.00%	k__Bacteria;p__unidentified_Bacteria;c__Saccharimonadia;o__Saccharimonadales;f__;g__;s__
OTU_1099	3	6	5	0.01%	k__Bacteria;p__Firmicutes;c__Negativicutes;o__Acidaminococcales;f__Acidaminococcaceae;g__Acidaminococcus
OTU_1100	3	0	1	0.00%	k__Bacteria;p__Firmicutes;c__Clostridia;o__Clostridia_vadinBB60_group;f__;g__;s__
OTU_1101	167	184	164	0.26%	k__Bacteria;p__Campylobacterota;c__Campylobacteria;o__Campylobacterales;f__Arcobacteraceae
OTU_1102	0	0	0	0.00%	k__Bacteria
OTU_1103	3	0	4	0.00%	k__Bacteria;p__Proteobacteria;c__Alphaproteobacteria;o__Caulobacterales;f__Caulobacteraceae;g__Brevundimonas;s__Brevundimonas_terrae
OTU_1104	6	4	3	0.01%	k__Bacteria;p__Firmicutes;c__Bacilli;o__Bacillales;f__Planococcaceae;g__Planococcus

OTU_1105	0	0	2	0.00%	k__Bacteria;p__Firmicutes;c__Negativicutes;o__Veillonellales-Selenomonadales;f__Veillonellaceae;g__Megasphaera;s__Megasphaera_micronuciformis
OTU_1106	270	289	271	0.42%	k__Bacteria;p__Actinobacteria;c__unidentified_Actinobacteria;o__Bifidobacteriales;f__Bifidobacteriaceae;g__Bifidobacterium;s__Bifidobacterium_adolescentis
OTU_1107	39	26	42	0.05%	k__Bacteria;p__Proteobacteria;c__Gammaproteobacteria;o__Burkholderiales;f__Rhodocyclaceae;g__Dechlorobacter;s__
OTU_1108	7	7	2	0.01%	k__Bacteria;p__Firmicutes;c__Negativicutes;o__Veillonellales-Selenomonadales;f__Veillonellaceae;g__Megasphaera
OTU_1109	0	0	2	0.00%	k__Bacteria;p__Proteobacteria;c__Alphaproteobacteria;o__Rhodospirillales;f__Rhodospirillaceae;g__Novispirillum
OTU_1110	2	0	1	0.00%	k__Bacteria;p__Firmicutes;c__Negativicutes;o__Veillonellales-Selenomonadales;f__Veillonellaceae;g__Dialister
OTU_1111	1	0	0	0.00%	k__Bacteria;p__Bacteroidota;c__Bacteroidia;o__Bacteroidales;f__Rikenellaceae;g__Alistipes;s__Alistipes_inops
OTU_1112	4	0	0	0.00%	k__Bacteria;p__Bacteroidota;c__Bacteroidia;o__Sphingobacteriales;f__AKYH767;g__;s__
OTU_1113	0	0	0	0.00%	k__Bacteria;p__Proteobacteria;c__Gammaproteobacteria;o__Enterobacterales;f__Morganellaceae;g__Proteus;s__Proteus_mirabilis
OTU_1114	3	2	2	0.00%	k__Bacteria;p__Desulfobacterota;c__Desulfovibrionia;o__Desulfovibrionales;f__Desulfovibrionaceae;g__Desulfovibrio
OTU_1115	4	3	0	0.00%	k__Bacteria;p__Firmicutes;c__Clostridia;o__Lachnospirales;f__Lachnospiraceae;g__Anaerosporobacter
OTU_1116	0	0	0	0.00%	k__Bacteria;p__Fusobacteriota;c__Fusobacteriia;o__Fusobacteriales;f__Leptotrichiaceae;g__Leptotrichia;s__
OTU_1117	0	0	1	0.00%	k__Bacteria;p__Firmicutes;c__Clostridia;o__Lachnospirales;f__Lachnospiraceae;g__[Bacteroides]_pectinophilus_group;s__
OTU_1118	0	0	3	0.00%	k__Bacteria;p__Firmicutes;c__Bacilli;o__Acholeplasmatales;f__Acholeplasmataceae;g__Acholeplasma;s__Acholeplasma_manati
OTU_1119	0	0	0	0.00%	k__Bacteria;p__Firmicutes;c__Bacilli;o__Erysipelotrichales;f__Erysipelatoclostridiaceae;g__UCG-004;s__bacterium_enrichment_culture_clone_DPHB07
OTU_1120	13	0	0	0.01%	k__Bacteria;p__Firmicutes;c__Bacilli;o__Bacillales;f__Bacillaceae;g__Bacillus
OTU_1121	0	0	3	0.00%	k__Bacteria;p__Proteobacteria;c__Gammaproteobacteria;o__Methylococcales;f__Methylomonadaceae
OTU_1122	2	0	0	0.00%	k__Bacteria;p__Proteobacteria;c__Gammaproteobacteria;o__Burkholderiales;f__Alcaligenaceae;g__Paenalcaligenes;s__
OTU_1123	0	2	3	0.00%	k__Bacteria;p__Bacteroidota;c__Bacteroidia;o__Chitinophagales;f__Chitinophagaceae;g__Sediminibacterium;s__
OTU_1124	3	0	0	0.00%	k__Bacteria
OTU_1125	0	3	1	0.00%	k__Bacteria;p__Desulfobacterota;c__Desulfobacteria;o__Desulfobacterales;f__unidentified_Desulfobacterales;g__Desulfatiferula
OTU_1126	1	0	1	0.00%	k__Bacteria;p__unidentified_Bacteria;c__Gammaproteobacteria;o__Xanthomonadales;f__Rhodanobacteraceae;g__;s__
OTU_1127	613	709	605	0.98%	k__Bacteria;p__Campylobacterota;c__Campylobacteria;o__Campylobacterales;f__Arcobacteraceae
OTU_1128	6	0	0	0.00%	k__Bacteria;p__Proteobacteria;c__Alphaproteobacteria;o__Sphingomonadales;f__Sphingomonadaceae;g__Altererythrobacter;s__
OTU_1129	2	1	3	0.00%	k__Bacteria;p__Bacteroidota;c__Bacteroidia;o__Cytophagales;f__Spirosomaceae
OTU_1130	1	3	0	0.00%	k__Bacteria;p__Synergistota;c__Synergistia;o__Synergistales;f__Synergistaceae;g__Syner-01;s__
OTU_1131	9	8	15	0.02%	k__Bacteria;p__Proteobacteria;c__Gammaproteobacteria;o__Burkholderiales;f__Comamonadaceae
OTU_1132	0	6	0	0.00%	k__Bacteria;p__Firmicutes;c__Negativicutes;o__Veillonellales-Selenomonadales;f__Veillonellaceae;g__Megasphaera
OTU_1133	30	23	30	0.04%	k__Bacteria;p__Campylobacterota;c__Campylobacteria;o__Campylobacterales;f__Arcobacteraceae;g__Arcobacter
OTU_1134	0	3	0	0.00%	k__Bacteria;p__Proteobacteria;c__Gammaproteobacteria;o__Burkholderiales;f__Rhodocyclaceae;g__Zoogloea;s__
OTU_1135	0	1	0	0.00%	k__Bacteria;p__Kapabacteria;c__unidentified_Kapabacteria;o__Kapabacteriales;f__;g__;s__
OTU_1136	0	2	0	0.00%	k__Bacteria;p__Proteobacteria;c__Alphaproteobacteria;o__Paracaedibacterales;f__Paracaedibacteraceae;g__Candidatus_Finniella;s__
OTU_1137	1	3	0	0.00%	k__Bacteria;p__unidentified_Bacteria;c__Bacteroidia;o__Chitinophagales;f__Saprospiraceae;g__;s__
OTU_1138	17	1	6	0.01%	k__Bacteria;p__Proteobacteria;c__Gammaproteobacteria;o__Xanthomonadales;f__Xanthomonadaceae;g__Stenotrophomonas
OTU_1139	0	2	0	0.00%	k__Bacteria;p__unidentified_Bacteria;c__Alphaproteobacteria;o__;f__;g__;s__
OTU_1140	1	2	0	0.00%	k__Bacteria
OTU_1141	3	0	3	0.00%	k__Bacteria
OTU_1142	3	2	0	0.00%	k__Bacteria;p__Bacteroidota;c__Bacteroidia;o__Bacteroidales;f__Prevotellaceae;g__Prevotella_7;s__
OTU_1143	0	6	1	0.00%	k__Bacteria;p__unidentified_Bacteria;c__Clostridia;o__Oscillospirales;f__Oscillospiraceae;g__;s__
OTU_1144	0	3	0	0.00%	k__Bacteria;p__Acidobacteriota;c__Vicinamibacteria;o__Vicinamibacterales;f__Vicinamibacteraceae;g__;s__
OTU_1145	1	0	1	0.00%	k__Bacteria;p__Bacteroidota;c__Bacteroidia;o__Bacteroidales;f__Prevotellaceae
OTU_1146	3	0	0	0.00%	k__Bacteria;p__Bacteroidota;c__Bacteroidia;o__Bacteroidales;f__Tannerellaceae;g__Parabacteroides;s__
OTU_1147	0	0	0	0.00%	k__Bacteria;p__Verrucomicrobiota;c__Verrucomicrobiae;o__Opitutales;f__Opitutaceae
OTU_1148	3	0	0	0.00%	k__Bacteria;p__unidentified_Bacteria;c__Alphaproteobacteria;o__Holosporales;f__Holosporaceae;g__;s__
OTU_1149	0	1	1	0.00%	k__Bacteria;p__Proteobacteria;c__Gammaproteobacteria;o__Pseudomonadales;f__Pseudomonadaceae;g__Pseudomonas;s__Pseudomonas_sp_108Z1
OTU_1150	0	1	0	0.00%	k__Bacteria;p__Proteobacteria;c__Gammaproteobacteria;o__Burkholderiales
OTU_1151	0	0	0	0.00%	k__Bacteria;p__Proteobacteria;c__Gammaproteobacteria;o__Burkholderiales;f__Rhodocyclaceae;g__Sulfuritalea;s__
OTU_1152	1	4	0	0.00%	k__Bacteria;p__Proteobacteria;c__Alphaproteobacteria;o__Azospirillales;f__Azospirillaceae;g__Skermanella;s__
OTU_1153	1	1	1	0.00%	k__Bacteria;p__unidentified_Bacteria;c__Bacteroidia;o__Chitinophagales;f__;g__;s__
OTU_1154	0	1	1	0.00%	k__Bacteria
OTU_1155	0	0	0	0.00%	k__Bacteria;p__Proteobacteria;c__Gammaproteobacteria;o__Burkholderiales
OTU_1156	0	2	0	0.00%	k__Bacteria;p__Bacteroidota;c__Bacteroidia;o__Bacteroidales;f__Muribaculaceae;g__;s__
OTU_1157	0	4	0	0.00%	k__Bacteria;p__Firmicutes;c__Clostridia;o__Christensenellales;f__Christensenellaceae;g__Christensenellaceae_R-7_group;s__bacterium_enrichment_culture_clone_DPF35
OTU_1158	0	0	3	0.00%	k__Bacteria;p__Actinobacteria;c__unidentified_Actinobacteria;o__Propionibacteriales;f__Propionibacteriaceae;g__Propioniciclava;s__
OTU_1159	4	6	4	0.01%	k__Bacteria;p__Proteobacteria;c__Gammaproteobacteria;o__Burkholderiales;f__Rhodocyclaceae
OTU_1160	4	5	0	0.00%	k__Bacteria;p__Bacteroidota;c__Bacteroidia;o__Bacteroidales;f__Rikenellaceae;g__Alistipes;s__Alistipes_indistinctus

OTU_1161	0	1	0	0.00%	k__Bacteria;p__Firmicutes;c__Desulfitobacteriia;o__Desulfitobacteriales;f__unidentified_Desulfitobacteriales;g__TC1;s__
OTU_1162	0	0	0	0.00%	k__Bacteria;p__Bacteroidota;c__Bacteroidia;o__Bacteroidales;f__Bacteroidaceae;g__Bacteroides;s__
OTU_1163	0	1	0	0.00%	k__Bacteria;p__Proteobacteria;c__Gammaproteobacteria;o__Enterobacterales
OTU_1164	0	0	0	0.00%	k__Bacteria;p__Bacteroidota;c__Bacteroidia;o__Bacteroidales;f__Muribaculaceae;g__;s__
OTU_1165	2	3	0	0.00%	k__Bacteria;p__Proteobacteria;c__Gammaproteobacteria;o__Legionellales;f__Legionellaceae;g__Legionella
OTU_1166	0	4	0	0.00%	k__Bacteria;p__Firmicutes;c__Clostridia;o__Clostridiales;f__Clostridiaceae;g__Clostridium_sensu_stricto_8;s__
OTU_1167	0	4	0	0.00%	k__Bacteria;p__Bacteroidota;c__Bacteroidia;o__Cytophagales;f__Spirosomaceae;g__Spirosoma;s__Spirosoma_oryzae
OTU_1168	0	0	1	0.00%	k__Bacteria;p__Elusimicrobia;c__unidentified_Elusimicrobia;o__Elusimicrobiales;f__Elusimicrobiaceae;g__Elusimicrobium;s__
OTU_1169	1	0	2	0.00%	k__Bacteria;p__Bacteroidota;c__Bacteroidia;o__Chitinophagales;f__Saprospiraceae;g__Aureispira;s__
OTU_1170	1	0	1	0.00%	k__Bacteria;p__unidentified_Bacteria;c__Coriobacteriia;o__Coriobacteriales;f__;g__;s__
OTU_1171	0	0	0	0.00%	k__Bacteria;p__Verrucomicrobiota;c__Verrucomicrobiae;o__Opitutales;f__Opitutaceae;g__Lacunisphaera;s__
OTU_1172	0	2	0	0.00%	k__Bacteria;p__Bacteroidota;c__Bacteroidia;o__Bacteroidales;f__PeH15;g__;s__
OTU_1173	0	0	0	0.00%	k__Bacteria
OTU_1174	18	11	11	0.02%	k__Bacteria
OTU_1175	6	1	2	0.00%	k__Bacteria;p__Firmicutes;c__Clostridia;o__Peptostreptococcales-Tissierellales;f__Family_XI;g__Tissierella;s__
OTU_1176	1	0	0	0.00%	k__Bacteria;p__Proteobacteria;c__Alphaproteobacteria;o__Caedibacterales;f__Caedibacteraceae;g__Caedibacter;s__
OTU_1177	0	0	0	0.00%	k__Bacteria;p__Bacteroidota;c__Bacteroidia;o__Bacteroidales;f__Muribaculaceae;g__;s__
OTU_1178	0	3	0	0.00%	k__Bacteria;p__unidentified_Bacteria;c__Alphaproteobacteria
OTU_1179	5	9	2	0.01%	k__Bacteria
OTU_1180	3	0	1	0.00%	k__Bacteria;p__Firmicutes;c__Bacilli;o__Lactobacillales;f__Carnobacteriaceae;g__Atopostipes;s__
OTU_1181	0	2	0	0.00%	k__Bacteria;p__Cyanobacteria;c__Sericytochromatia;o__;f__;g__;s__
OTU_1182	3	0	0	0.00%	k__Bacteria;p__Desulfobacterota;c__Desulfovibrionia;o__Desulfovibrionales;f__Desulfovibrionaceae;g__Desulfovibrio;s__toluene-degrading_methanogenic_consortium_bacterium
OTU_1183	2	1	0	0.00%	k__Bacteria;p__Actinobacteriota;c__Coriobacteriia;o__Coriobacteriales;f__Atopobiaceae;g__Olsenella;s__Olsenella_scatoligenes
OTU_1184	2	1	0	0.00%	k__Bacteria;p__unidentified_Bacteria;c__Bacteroidia;o__Bacteroidales;f__Dysgonomonadaceae;g__;s__
OTU_1185	0	0	0	0.00%	k__Bacteria;p__Bdellovibrionota;c__Bdellovibrionia;o__Bdellovibrionales;f__Bdellovibrionaceae;g__Bdellovibrio;s__
OTU_1186	2	0	0	0.00%	k__Bacteria;p__unidentified_Bacteria;c__Clostridia;o__Lachnospirales;f__Lachnospiraceae;g__unidentified_Lachnospiraceae;s__Clostridium_sp_Culture-54
OTU_1187	3	0	1	0.00%	k__Bacteria;p__Actinobacteria;c__unidentified_Actinobacteria;o__Corynebacteriales;f__Dietziaceae;g__Dietzia
OTU_1188	2	0	0	0.00%	k__Bacteria
OTU_1189	217	241	230	0.35%	k__Bacteria;p__Proteobacteria;c__Gammaproteobacteria;o__Pseudomonadales;f__Moraxellaceae;g__Acinetobacter;s__Acinetobacter_sp_WCHAc010034
OTU_1190	0	0	0	0.00%	k__Bacteria;p__Firmicutes;c__Bacilli;o__RF39;f__;g__;s__
OTU_1191	0	0	1	0.00%	k__Bacteria;p__unidentified_Bacteria;c__Anaerolineae;o__Anaerolineales;f__Anaerolineaceae;g__;s__
OTU_1192	0	8	1	0.00%	k__Bacteria;p__Bdellovibrionota;c__Bdellovibrionia;o__Bacteriovoracales;f__Bacteriovoracaceae;g__Bacteriovorax;s__
OTU_1193	0	1	1	0.00%	k__Bacteria;p__Actinobacteria;c__unidentified_Actinobacteria;o__Corynebacteriales;f__Mycobacteriaceae;g__Mycobacterium
OTU_1194	0	0	0	0.00%	k__Bacteria;p__Bacteroidota;c__Bacteroidia;o__Chitinophagales;f__Chitinophagaceae;g__Ferruginibacter
OTU_1195	1	3	0	0.00%	k__Bacteria;p__Firmicutes;c__Bacilli;o__Lactobacillales;f__Lactobacillaceae;g__Leuconostoc;s__Leuconostoc_pseudomesenteroides
OTU_1196	3	1	0	0.00%	k__Bacteria;p__Bacteroidota;c__Bacteroidia;o__Cytophagales;f__Hymenobacteraceae;g__Hymenobacter
OTU_1197	0	4	1	0.00%	k__Bacteria;p__Proteobacteria;c__Gammaproteobacteria;o__Pseudomonadales;f__Pseudomonadaceae;g__Pseudomonas;s__Pseudomonas_pohangensis
OTU_1198	3	0	4	0.00%	k__Bacteria;p__Desulfobacterota;c__Desulfuromonadia;o__Geobacterales
OTU_1199	0	2	0	0.00%	k__Bacteria;p__Acidobacteriota;c__Thermoanaerobaculia;o__Thermoanaerobaculales;f__Thermoanaerobaculaceae;g__Subgroup_10
OTU_1200	0	3	0	0.00%	k__Bacteria;p__Actinobacteria;c__unidentified_Actinobacteria;o__Pseudonocardiales;f__Pseudonocardiaceae;g__Pseudonocardia;s__
OTU_1201	6	4	1	0.01%	k__Bacteria;p__Proteobacteria;c__Alphaproteobacteria;o__Rhodobacterales;f__Rhodobacteraceae;g__Paracoccus
OTU_1202	0	0	0	0.00%	k__Bacteria;p__Proteobacteria;c__Gammaproteobacteria;o__Enterobacterales;f__Orbaceae;g__Gilliamella
OTU_1203	1	3	0	0.00%	k__Bacteria;p__Gemmatimonadetes;c__unidentified_Gemmatimonadetes;o__Gemmatimonadales;f__Gemmatimonadaceae;g__Roseisolibacter;s__
OTU_1204	2	2	3	0.00%	k__Bacteria;p__Synergistota;c__Synergistia;o__Synergistales;f__Synergistaceae;g__Syner-01;s__
OTU_1205	0	0	3	0.00%	k__Bacteria;p__unidentified_Bacteria;c__Saccharimonadia;o__Saccharimonadales;f__Saccharimonadaceae;g__TM7a;s__
OTU_1206	0	0	0	0.00%	k__Bacteria;p__Proteobacteria;c__Gammaproteobacteria;o__Enterobacterales;f__Yersiniaceae
OTU_1207	0	2	1	0.00%	k__Bacteria;p__Sumerlaeota;c__Sumerlaeia;o__Sumerlaeales;f__Sumerlaeaceae;g__Sumerlaea;s__
OTU_1208	2	4	1	0.00%	k__Bacteria;p__Actinobacteriota;c__Acidimicrobiia;o__Microtrichales;f__Iamiaceae;g__Iamia;s__
OTU_1209	0	0	0	0.00%	k__Bacteria
OTU_1210	0	1	0	0.00%	k__Bacteria
OTU_1211	0	3	0	0.00%	k__Bacteria;p__Spirochaetota;c__Spirochaetia;o__Spirochaetales;f__Spirochaetaceae;g__Treponema
OTU_1212	27	35	26	0.04%	k__Bacteria;p__Firmicutes;c__Clostridia;o__Lachnospirales;f__Lachnospiraceae;g__Roseburia;s__Roseburia_intestinalis
OTU_1213	0	2	1	0.00%	k__Bacteria;p__Firmicutes;c__Clostridia;o__Clostridia_UCG-014;f__;g__;s__
OTU_1214	7	0	0	0.00%	k__Bacteria;p__RCP2-54;c__;o__;f__;g__;s__
OTU_1215	0	0	1	0.00%	k__Bacteria;p__Firmicutes;c__Clostridia;o__Peptostreptococcales-Tissierellales;f__Family_XI;g__W5053;s__
OTU_1216	4	3	5	0.01%	k__Bacteria;p__Proteobacteria;c__Gammaproteobacteria;o__Pseudomonadales;f__Cellvibrionaceae;g__Cellvibrio

OTU_1217	1	2	0	0.00%	k__Bacteria;p__unidentified_Bacteria;c__Acidimicrobiia;o__Microtrichales;f__;g__;s__
OTU_1218	0	0	0	0.00%	k__Bacteria;p__Campylobacterota;c__Campylobacteria;o__Campylobacterales;f__Campylobacteraceae;g__Campylobacter;s__Campylobacter_showae
OTU_1219	1	0	2	0.00%	k__Bacteria;p__Bacteroidota;c__Bacteroidia;o__Cytophagales;f__Spirosomaceae;g__Emticicia;s__
OTU_1220	3	0	0	0.00%	k__Bacteria;p__Firmicutes;c__Clostridia;o__Oscillospirales;f__[Eubacterium]_coprostanoligenes_group;g__;s__
OTU_1221	3	0	0	0.00%	k__Bacteria;p__FCPU426;c__;o__;f__;g__;s__
OTU_1222	0	0	0	0.00%	k__Bacteria;p__unidentified_Bacteria;c__Clostridia;o__Oscillospirales;f__Ruminococcaceae;g__;s__
OTU_1223	0	0	0	0.00%	k__Bacteria;p__Bacteroidota;c__Bacteroidia;o__Flavobacteriales;f__Weeksellaceae;g__Bergeyella
OTU_1224	0	1	0	0.00%	k__Bacteria;p__unidentified_Bacteria;c__Saccharimonadia;o__Saccharimonadales
OTU_1225	0	0	0	0.00%	k__Bacteria;p__Firmicutes;c__Bacilli;o__Erysipelotrichales;f__Erysipelotrichaceae;g__;s__
OTU_1226	1	2	0	0.00%	k__Bacteria;p__Myxococcota;c__Polyangia;o__Nannocystales;f__Nannocystaceae;g__;s__
OTU_1227	8	4	3	0.01%	k__Bacteria;p__Firmicutes;c__Clostridia;o__Oscillospirales;f__Oscillospiraceae;g__UCG-005;s__
OTU_1228	0	1	3	0.00%	k__Bacteria;p__Campylobacterota;c__Campylobacteria;o__Campylobacterales;f__Sulfurospirillaceae;g__Sulfurospirillum;s__
OTU_1229	1	1	3	0.00%	k__Bacteria;p__Firmicutes;c__Clostridia;o__Oscillospirales;f__Oscillospiraceae
OTU_1230	0	0	0	0.00%	k__Bacteria
OTU_1231	1	1	2	0.00%	k__Bacteria;p__Campylobacterota;c__Campylobacteria;o__Campylobacterales;f__Sulfurimonadaceae;g__Sulfurimonas;s__
OTU_1232	0	0	1	0.00%	k__Bacteria;p__Synergistota;c__Synergistia;o__Synergistales;f__Synergistaceae;g__Aminomonas;s__
OTU_1233	0	13	2	0.01%	k__Bacteria;p__Actinobacteria;c__unidentified_Actinobacteria;o__Micrococcales;f__Intrasporangiaceae;g__Ornithinimicrobium;s__
OTU_1234	16	4	6	0.01%	k__Bacteria;p__Proteobacteria;c__Gammaproteobacteria;o__Pseudomonadales;f__Moraxellaceae;g__Acinetobacter;s__Acinetobacter_venetianus
OTU_1235	4	0	1	0.00%	k__Bacteria;p__unidentified_Bacteria;c__Gammaproteobacteria;o__Xanthomonadales;f__Rhodanobacteraceae;g__;s__
OTU_1236	0	2	2	0.00%	k__Bacteria;p__Actinobacteria;c__unidentified_Actinobacteria;o__Corynebacteriales;f__Corynebacteriaceae;g__Corynebacterium;s__Corynebacterium_tuberculostearicum
OTU_1237	0	0	1	0.00%	k__Bacteria;p__unidentified_Bacteria;c__Anaerolineae;o__Anaerolineales;f__Anaerolineaceae;g__;s__
OTU_1238	2	0	1	0.00%	k__Bacteria;p__unidentified_Bacteria;c__Bacteroidia;o__Chitinophagales;f__Chitinophagaceae
OTU_1239	0	0	7	0.00%	k__Bacteria;p__Bacteroidota;c__Bacteroidia;o__Bacteroidales;f__Muribaculaceae;g__;s__
OTU_1240	1	0	2	0.00%	k__Bacteria;p__Hydrogenedentes;c__Hydrogenedentia;o__Hydrogenedentiales;f__Hydrogenedensaceae;g__;s__
OTU_1241	0	1	0	0.00%	k__Bacteria;p__unidentified_Bacteria;c__Gammaproteobacteria;o__Salinisphaerales;f__Solimonadaceae;g__;s__
OTU_1242	0	0	0	0.00%	k__Bacteria;p__Proteobacteria;c__Gammaproteobacteria;o__unidentified_Gammaproteobacteria;f__unidentified_Gammaproteobacteria;g__;s__
OTU_1243	3	0	0	0.00%	k__Bacteria;p__Bacteroidota;c__Bacteroidia;o__Bacteroidales;f__Dysgonomonadaceae
OTU_1244	0	0	0	0.00%	k__Bacteria;p__unidentified_Bacteria;c__Desulfovibrionia;o__Desulfovibrionales;f__Desulfovibrionaceae;g__;s__
OTU_1245	0	0	0	0.00%	k__Bacteria;p__Bacteroidota;c__Bacteroidia;o__Bacteroidales;f__Prevotellaceae;g__Alloprevotella;s__Alloprevotella_tannerae
OTU_1246	0	2	0	0.00%	k__Bacteria;p__Firmicutes;c__Clostridia;o__Oscillospirales;f__Ruminococcaceae;g__Ruminococcus
OTU_1247	0	1	2	0.00%	k__Bacteria;p__unidentified_Bacteria;c__Alphaproteobacteria;o__Rhodospirillales;f__;g__;s__
OTU_1248	0	0	0	0.00%	k__Bacteria;p__Firmicutes;c__Bacilli;o__Erysipelotrichales;f__Erysipelotrichaceae;g__Erysipelothrix
OTU_1249	3	2	0	0.00%	k__Bacteria;p__Proteobacteria;c__Gammaproteobacteria;o__Burkholderiales
OTU_1250	2	0	2	0.00%	k__Bacteria;p__Firmicutes;c__Bacilli;o__Exiguobacterales;f__Exiguobacteraceae;g__Exiguobacterium
OTU_1251	3	0	0	0.00%	k__Bacteria;p__Myxococcota;c__Polyangia;o__Polyangiales;f__Phaselicystidaceae;g__Phaselicystis;s__
OTU_1252	1	1	0	0.00%	k__Bacteria;p__Nitrospirota;c__Nitrospira;o__Nitrospirales;f__Nitrospiraceae
OTU_1253	80	114	90	0.14%	k__Bacteria;p__Firmicutes;c__Bacilli;o__Lactobacillales;f__Streptococcaceae;g__Streptococcus
OTU_1254	0	0	0	0.00%	k__Bacteria
OTU_1255	0	0	2	0.00%	k__Bacteria;p__Bacteroidota;c__Bacteroidia;o__Cytophagales;f__Spirosomaceae;g__Persicitalea;s__
OTU_1256	7	0	1	0.00%	k__Bacteria;p__Firmicutes;c__Clostridia;o__Lachnospirales;f__Lachnospiraceae;g__Tyzzerella;s__
OTU_1257	0	0	0	0.00%	k__Bacteria;p__Proteobacteria;c__Gammaproteobacteria;o__Pseudomonadales;f__Cellvibrionaceae;g__Cellvibrio;s__
OTU_1258	0	1	0	0.00%	k__Bacteria;p__WPS-2;c__;o__;f__;g__;s__
OTU_1259	0	1	1	0.00%	k__Bacteria;p__Bacteroidota;c__Bacteroidia;o__Sphingobacteriales;f__NS11-12_marine_group;g__;s__
OTU_1260	0	0	3	0.00%	k__Bacteria;p__Bacteroidota;c__Bacteroidia;o__Chitinophagales;f__Chitinophagaceae;g__Taibaiella;s__
OTU_1261	3	0	0	0.00%	k__Bacteria
OTU_1262	0	0	0	0.00%	k__Bacteria;p__Firmicutes;c__Clostridia;o__Clostridia_vadinBB60_group;f__;g__;s__
OTU_1263	0	0	1	0.00%	k__Bacteria;p__Cyanobacteria;c__Cyanobacteriia;o__Chloroplast
OTU_1264	1	0	3	0.00%	k__Bacteria;p__Gracilibacteria;c__unidentified_Gracilibacteria;o__unidentified_Gracilibacteria;f__unidentified_Gracilibacteria;g__unidentified_Gracilibacteria
OTU_1265	0	0	3	0.00%	k__Bacteria;p__Proteobacteria;c__Gammaproteobacteria;o__Pseudomonadales;f__Pseudomonadaceae
OTU_1266	1	0	0	0.00%	k__Bacteria;p__unidentified_Bacteria;c__Clostridia;o__Oscillospirales;f__Ruminococcaceae;g__;s__
OTU_1267	2	0	0	0.00%	k__Bacteria;p__Proteobacteria;c__Alphaproteobacteria;o__unidentified_Alphaproteobacteria;f__unidentified_Alphaproteobacteria;g__Neomegalonema;s__Neomegalonema_perideroedes
OTU_1268	0	2	1	0.00%	k__Bacteria;p__Bacteroidota;c__Bacteroidia;o__Bacteroidales;f__Prevotellaceae;g__Prevotellaceae_UCG-004;s__
OTU_1269	3	0	0	0.00%	k__Bacteria;p__Firmicutes;c__Clostridia;o__Oscillospirales;f__Ruminococcaceae;g__Anaerotruncus;s__
OTU_1270	0	1	0	0.00%	k__Bacteria;p__Firmicutes;c__Clostridia;o__Clostridia_vadinBB60_group;f__;g__;s__
OTU_1271	0	3	0	0.00%	k__Bacteria;p__Firmicutes;c__Clostridia;o__Oscillospirales;f__Butyricicoccaceae
OTU_1272	0	0	3	0.00%	k__Bacteria;p__Proteobacteria;c__Alphaproteobacteria;o__Rickettsiales;f__Mitochondria

OTU_1273	7	1	1	0.00%	k__Bacteria;p__Bacteroidota;c__Bacteroidia;o__Cytophagales;f__Spirosomaceae;g__Lacihabitans;s__
OTU_1274	0	0	0	0.00%	k__Bacteria;p__Desulfobacterota;c__Desulfovibrionia;o__Desulfovibrionales;f__Desulfovibrionaceae;g__Bilophila;s__
OTU_1275	2	0	6	0.00%	k__Bacteria;p__Proteobacteria;c__Gammaproteobacteria;o__Burkholderiales;f__Chitinibacteraceae;g__Chitinibacter
OTU_1276	1	1	1	0.00%	k__Bacteria;p__Bacteroidota;c__Bacteroidia;o__Flavobacteriales;f__Crocinitomicaceae;g__Fluviicola;s__
OTU_1277	3	0	1	0.00%	k__Bacteria;p__Acidobacteriota;c__Blastocatellia;o__Blastocatellales;f__Blastocatellaceae;g__Stenotrophobacter;s__
OTU_1278	0	0	9	0.00%	k__Bacteria;p__Deferribacteres;c__unidentified_Deferribacteres;o__Deferribacterales;f__Deferribacteraceae;g__Mucispirillum;s__Mucispirillum_schaedleri
OTU_1279	2	5	0	0.00%	k__Bacteria;p__Acidobacteriota;c__Acidobacteriae;o__Bryobacterales;f__Bryobacteraceae;g__Bryobacter;s__
OTU_1280	1	1	0	0.00%	k__Bacteria;p__Bacteroidota;c__Bacteroidia;o__SM1A07;f__;g__;s__
OTU_1281	4	0	0	0.00%	k__Bacteria;p__Firmicutes;c__Clostridia;o__Christensenellales;f__Christensenellaceae;g__Christensenellaceae_R-7_group;s__
OTU_1282	1	0	0	0.00%	k__Bacteria;p__Firmicutes;c__Clostridia;o__Christensenellales;f__Christensenellaceae;g__Christensenellaceae_R-7_group;s__iron-reducing_bacterium_enrichment_culture_clone_HN70
OTU_1283	0	2	2	0.00%	k__Bacteria;p__Bacteroidota;c__Bacteroidia;o__Cytophagales;f__Spirosomaceae;g__Lacihabitans;s__
OTU_1284	0	3	0	0.00%	k__Bacteria;p__Acidobacteriota;c__Vicinamibacteria;o__Subgroup_17;f__;g__;s__
OTU_1285	113	43	102	0.13%	k__Bacteria;p__Proteobacteria;c__Gammaproteobacteria;o__Burkholderiales;f__Comamonadaceae;g__Macromonas;s__
OTU_1286	0	0	0	0.00%	k__Bacteria;p__Desulfobacterota;c__Desulfovibrionia;o__Desulfovibrionales;f__Desulfovibrionaceae;g__Desulfovibrio;s__Desulfovibrio_vulgaris
OTU_1287	0	0	0	0.00%	k__Bacteria;p__Cyanobacteria;c__Vampirivibrionia;o__Gastranaerophilales;f__;g__;s__
OTU_1288	4	0	0	0.00%	k__Bacteria;p__Actinobacteria;c__unidentified_Actinobacteria
OTU_1289	0	2	0	0.00%	k__Bacteria;p__Firmicutes;c__Bacilli;o__Lactobacillales;f__Lactobacillaceae;g__Lactobacillus;s__Lactobacillus_iners
OTU_1290	3	1	1	0.00%	k__Bacteria;p__Firmicutes;c__Clostridia;o__Lachnospirales;f__Lachnospiraceae;g__Marvinbryantia;s__
OTU_1291	2	1	4	0.00%	k__Bacteria;p__Bacteroidota;c__Bacteroidia;o__Bacteroidales;f__Williamwhitmaniaceae;g__Blvii28_wastewater-sludge_group;s__
OTU_1292	0	0	0	0.00%	k__Bacteria;p__Firmicutes;c__Clostridia;o__Clostridia_UCG-014;f__;g__;s__
OTU_1293	0	0	0	0.00%	k__Bacteria;p__Firmicutes;c__Clostridia;o__Clostridia_vadinBB60_group;f__;g__;s__
OTU_1294	3	3	2	0.00%	k__Bacteria;p__Proteobacteria;c__Gammaproteobacteria;o__Burkholderiales;f__Comamonadaceae;g__Methylibium;s__
OTU_1295	1	0	2	0.00%	k__Bacteria;p__unidentified_Bacteria;c__Gammaproteobacteria;o__Pseudomonadales;f__Moraxellaceae;g__;s__
OTU_1296	0	3	3	0.00%	k__Bacteria;p__Bacteroidota;c__Bacteroidia;o__Bacteroidales;f__Tannerellaceae;g__Parabacteroides;s__Parabacteroides_gordonii
OTU_1297	1	0	0	0.00%	k__Bacteria;p__Bacteroidota;c__Bacteroidia;o__Bacteroidales;f__Prevotellaceae
OTU_1298	0	0	0	0.00%	k__Bacteria;p__Firmicutes;c__Bacilli;o__Lactobacillales;f__Lactobacillaceae;g__Lactobacillus;s__Lactobacillus_apis
OTU_1299	0	0	0	0.00%	k__Bacteria;p__Proteobacteria;c__Alphaproteobacteria;o__Rhizobiales;f__Xanthobacteraceae;g__Xanthobacter;s__
OTU_1300	1	2	0	0.00%	k__Bacteria;p__Bacteroidota;c__Bacteroidia;o__Flavobacteriales;f__Flavobacteriaceae;g__Flavobacterium;s__Flavobacterium_sp_enrichment_culture_clone_SA_NR2_1
OTU_1301	0	2	0	0.00%	k__Bacteria;p__Firmicutes;c__Clostridia
OTU_1302	0	0	0	0.00%	k__Bacteria;p__Firmicutes;c__Clostridia;o__Clostridia_UCG-014;f__;g__;s__
OTU_1303	0	0	0	0.00%	k__Bacteria;p__Proteobacteria;c__Gammaproteobacteria;o__Enterobacterales;f__Pasteurellaceae;g__Aggregatibacter;s__Aggregatibacter_segnis
OTU_1304	0	0	0	0.00%	k__Bacteria
OTU_1305	3	0	0	0.00%	k__Bacteria;p__Proteobacteria;c__Gammaproteobacteria;o__Xanthomonadales;f__Xanthomonadaceae;g__Lysobacter;s__
OTU_1306	1	2	0	0.00%	k__Bacteria;p__Elusimicrobiota;c__Lineage_IIb;o__;f__;g__;s__
OTU_1307	0	2	1	0.00%	k__Bacteria;p__Firmicutes;c__Clostridia;o__Oscillospirales;f__Ruminococcaceae;g__Fournierella;s__
OTU_1308	1	0	0	0.00%	k__Bacteria;p__Bacteroidota;c__Bacteroidia;o__Flavobacteriales;f__Weeksellaceae;g__Chryseobacterium;s__Chryseobacterium_sp_A1-ST2
OTU_1309	3	3	1	0.00%	k__Bacteria;p__Firmicutes;c__Clostridia;o__Peptostreptococcales-Tissierellales;f__Acidaminobacteraceae;g__Acidaminobacter;s__
OTU_1310	1	2	0	0.00%	k__Bacteria;p__unidentified_Bacteria;c__Alphaproteobacteria;o__Micavibrionales;f__;g__;s__
OTU_1311	0	3	0	0.00%	k__Bacteria;p__Proteobacteria;c__Alphaproteobacteria;o__Rhizobiales;f__Beijerinckiaceae;g__Psychroglaciecola;s__
OTU_1312	7	5	0	0.01%	k__Bacteria;p__Proteobacteria;c__Alphaproteobacteria;o__Rhizobiales;f__Rhizobiaceae;g__Mesorhizobium
OTU_1313	5	0	0	0.00%	k__Bacteria;p__Firmicutes;c__Clostridia;o__Lachnospirales;f__Lachnospiraceae;g__Lachnospiraceae_UCG-001;s__
OTU_1314	0	1	0	0.00%	k__Bacteria;p__Spirochaetota;c__Leptospirae;o__Leptospirales;f__Leptospiraceae;g__Turneriella;s__
OTU_1315	0	0	0	0.00%	k__Bacteria;p__Proteobacteria;c__Gammaproteobacteria;o__Burkholderiales;f__Neisseriaceae;g__Neisseria;s__Neisseria_perflava
OTU_1316	2	0	0	0.00%	k__Bacteria;p__Proteobacteria;c__Alphaproteobacteria;o__Sphingomonadales;f__Sphingomonadaceae;g__Novosphingobium;s__
OTU_1317	2	7	1	0.01%	k__Bacteria;p__Actinobacteriota;c__Thermoleophilia;o__Solirubrobacterales;f__Solirubrobacteraceae;g__Solirubrobacter;s__
OTU_1318	0	0	0	0.00%	k__Bacteria;p__Firmicutes;c__Clostridia;o__Oscillospirales;f__UCG-010;g__;s__
OTU_1319	0	1	1	0.00%	k__Bacteria;p__Bdellovibrionota;c__Bdellovibrionia;o__Bdellovibrionales;f__Bdellovibrionaceae;g__Bdellovibrio
OTU_1320	10	8	1	0.01%	k__Bacteria;p__Firmicutes;c__Clostridia;o__Lachnospirales;f__Lachnospiraceae;g__[Eubacterium]_xylanophilum_group;s__
OTU_1321	0	4	2	0.00%	k__Bacteria;p__Bacteroidota;c__Bacteroidia;o__Bacteroidales;f__Prevotellaceae;g__Alloprevotella;s__
OTU_1322	6	6	6	0.01%	k__Bacteria;p__Firmicutes;c__Clostridia;o__Lachnospirales;f__Lachnospiraceae
OTU_1323	0	2	0	0.00%	k__Bacteria
OTU_1324	0	1	0	0.00%	k__Bacteria;p__Firmicutes;c__Clostridia;o__Peptostreptococcales-Tissierellales;f__unidentified_Peptostreptococcales-Tissierellales;g__Guggenheimella;s__
OTU_1325	0	0	0	0.00%	k__Bacteria;p__Firmicutes;c__Clostridia;o__Peptostreptococcales-Tissierellales;f__Anaerovoracaceae;g__[Eubacterium]_brachy_group;s__
OTU_1326	2	0	0	0.00%	k__Bacteria;p__Firmicutes;c__Clostridia;o__Clostridiales;f__Clostridiaceae;g__Candidatus_Arthromitus;s__Candidatus_Arthromitus_sp_SFB-mouse-NL
OTU_1327	0	2	0	0.00%	k__Bacteria;p__Proteobacteria;c__Gammaproteobacteria;o__Burkholderiales;f__Rhodocyclaceae;g__Sulfuritalea;s__
OTU_1328	4	0	0	0.00%	k__Bacteria;p__Proteobacteria;c__Gammaproteobacteria;o__Xanthomonadales;f__Xanthomonadaceae;g__Vulcaniibacterium;s__

OTU_1329	3	0	0	0.00%	k__Bacteria;p__Firmicutes;c__Clostridia;o__Oscillospirales;f__UCG-010;g__;s__
OTU_1330	0	1	1	0.00%	k__Bacteria;p__unidentified_Bacteria;c__Bacteroidia;o__Chitinophagales;f__Saprospiraceae;g__;s__
OTU_1331	0	0	0	0.00%	k__Bacteria;p__Firmicutes;c__Bacilli
OTU_1332	0	3	0	0.00%	k__Bacteria;p__unidentified_Bacteria;c__Bacteroidia;o__Chitinophagales;f__Saprospiraceae;g__;s__
OTU_1333	0	0	0	0.00%	k__Bacteria;p__Bacteroidota;c__Bacteroidia;o__Sphingobacteriales;f__Sphingobacteriaceae;g__Pedobacter
OTU_1334	0	0	0	0.00%	k__Bacteria;p__Bacteroidota;c__Bacteroidia;o__Bacteroidales;f__Muribaculaceae;g__;s__
OTU_1335	1	0	0	0.00%	k__Bacteria;p__Proteobacteria;c__Gammaproteobacteria;o__Pseudomonadales;f__Moraxellaceae
OTU_1336	0	0	0	0.00%	k__Bacteria;p__unidentified_Bacteria;c__Clostridia;o__Oscillospirales;f__Oscillospiraceae;g__;s__
OTU_1337	0	0	1	0.00%	k__Bacteria;p__unidentified_Bacteria;c__Babeliae;o__Babeliales;f__Vermiphilaceae;g__;s__
OTU_1338	1	1	2	0.00%	k__Bacteria;p__Proteobacteria;c__Gammaproteobacteria;o__Burkholderiales;f__Procabacteriaceae;g__Procabacter
OTU_1339	0	2	0	0.00%	k__Bacteria;p__Firmicutes;c__Clostridia;o__Lachnospirales;f__Lachnospiraceae;g__Lachnospiraceae_NK4A136_group
OTU_1340	0	2	0	0.00%	k__Bacteria
OTU_1341	2	0	0	0.00%	k__Bacteria;p__Firmicutes;c__Negativicutes;o__Veillonellales-Selenomonadales;f__Veillonellaceae;g__Megasphaera;s__Megasphaera_paucivorans
OTU_1342	0	1	0	0.00%	k__Bacteria;p__Bacteroidota;c__Bacteroidia;o__Flavobacteriales;f__Crocinitomicaceae;g__Fluviicola;s__
OTU_1343	2	0	0	0.00%	k__Bacteria;p__Proteobacteria;c__Gammaproteobacteria;o__Burkholderiales;f__Comamonadaceae
OTU_1344	0	0	0	0.00%	k__Bacteria;p__unidentified_Bacteria;c__Alphaproteobacteria;o__Kiloniellales;f__Fodinicurvataceae;g__;s__
OTU_1345	0	0	0	0.00%	k__Bacteria;p__Firmicutes;c__Clostridia;o__Oscillospirales;f__Ruminococcaceae;g__Harryflintia;s__
OTU_1346	0	0	0	0.00%	k__Bacteria;p__Firmicutes;c__Clostridia;o__Clostridia_vadinBB60_group;f__;g__;s__
OTU_1347	2	0	1	0.00%	k__Bacteria
OTU_1348	0	0	0	0.00%	k__Bacteria;p__Bdellovibrionota;c__Bdellovibrionia;o__Bdellovibrionales;f__Bdellovibrionaceae;g__Bdellovibrio
OTU_1349	2	1	1	0.00%	k__Bacteria;p__Bacteroidota;c__Bacteroidia;o__Cytophagales;f__Cytophagaceae;g__Cytophaga;s__Cytophaga_hutchinsonii
OTU_1350	3	0	0	0.00%	k__Bacteria;p__Bacteroidota;c__Bacteroidia;o__Bacteroidales;f__Williamwhitmaniaceae;g__Acetobacteroides;s__
OTU_1351	0	0	0	0.00%	k__Bacteria;p__Firmicutes;c__Bacilli;o__RF39;f__;g__;s__
OTU_1352	44	35	34	0.06%	k__Bacteria;p__Firmicutes;c__Clostridia;o__Lachnospirales;f__Lachnospiraceae;g__Blautia
OTU_1353	70	46	30	0.07%	k__Bacteria;p__Proteobacteria;c__Gammaproteobacteria;o__Pseudomonadales;f__Pseudomonadaceae;g__Pseudomonas
OTU_1354	2	0	0	0.00%	k__Bacteria;p__Verrucomicrobiota;c__Verrucomicrobiae;o__Opitutales;f__Opitutaceae;g__Opitutus;s__
OTU_1355	0	0	0	0.00%	k__Bacteria
OTU_1356	1	2	2	0.00%	k__Bacteria;p__Spirochaetota;c__Leptospirae;o__Leptospirales;f__Leptospiraceae;g__Turneriella
OTU_1357	0	1	5	0.00%	k__Bacteria;p__Desulfobacterota;c__Desulfobacteria;o__Desulfobacterales;f__Desulforegulaceae;g__Desulforegula;s__
OTU_1358	0	0	0	0.00%	k__Bacteria;p__Verrucomicrobiota;c__Verrucomicrobiae;o__Opitutales;f__Opitutaceae;g__Lacunisphaera;s__
OTU_1359	0	0	2	0.00%	k__Bacteria;p__Nitrospirota;c__Nitrospira;o__Nitrospirales;f__Nitrospiraceae
OTU_1360	0	1	0	0.00%	k__Bacteria;p__Firmicutes;c__Clostridia;o__Oscillospirales;f__Oscillospiraceae;g__Oscillibacter;s__
OTU_1361	0	0	0	0.00%	k__Bacteria;p__Firmicutes;c__Bacilli;o__Erysipelotrichales;f__Erysipelotrichaceae;g__Allobaculum;s__
OTU_1362	1	0	0	0.00%	k__Bacteria;p__Firmicutes;c__Clostridia;o__Peptostreptococcales-Tissierellales;f__Anaerovoracaceae;g__[Eubacterium]_nodatum_group;s__Aminicella_lysinilytica
OTU_1363	119	69	122	0.16%	k__Bacteria;p__Proteobacteria;c__Gammaproteobacteria;o__Burkholderiales;f__Comamonadaceae;g__Simplicispira
OTU_1364	0	0	0	0.00%	k__Bacteria;p__Proteobacteria;c__Gammaproteobacteria
OTU_1365	2	0	2	0.00%	k__Bacteria;p__unidentified_Bacteria;c__Gammaproteobacteria;o__Halothiobacillales;f__Halothiobacillaceae;g__;s__
OTU_1366	0	0	0	0.00%	k__Bacteria;p__unidentified_Bacteria;c__Bacteroidia;o__Chitinophagales;f__Saprospiraceae
OTU_1367	0	1	2	0.00%	k__Bacteria;p__unidentified_Bacteria;c__Alphaproteobacteria;o__Rhodospirillales;f__;g__;s__
OTU_1368	0	0	2	0.00%	k__Bacteria;p__Proteobacteria;c__Gammaproteobacteria;o__Burkholderiales;f__Comamonadaceae
OTU_1369	0	0	2	0.00%	k__Bacteria;p__Firmicutes;c__Desulfitobacteriia;o__Desulfitobacteriales;f__unidentified_Desulfitobacteriales;g__TC1;s__
OTU_1370	1	0	0	0.00%	k__Bacteria;p__Proteobacteria;c__Gammaproteobacteria;o__Pseudomonadales;f__Pseudomonadaceae;g__Oblitimonas;s__Oblitimonas_alkaliphila
OTU_1371	0	4	0	0.00%	k__Bacteria;p__unidentified_Bacteria;c__Vicinamibacteria;o__Vicinamibacterales;f__;g__;s__
OTU_1372	0	0	2	0.00%	k__Bacteria;p__Spirochaetota;c__Spirochaetia;o__Spirochaetales;f__Spirochaetaceae;g__Spirochaeta_2
OTU_1373	0	2	0	0.00%	k__Bacteria;p__Proteobacteria;c__Gammaproteobacteria;o__Enterobacterales;f__Succinivibrionaceae
OTU_1374	1	0	1	0.00%	k__Bacteria;p__unidentified_Bacteria
OTU_1375	0	2	0	0.00%	k__Bacteria;p__Firmicutes;c__Bacilli;o__Lactobacillales;f__Aerococcaceae;g__Facklamia
OTU_1376	0	0	0	0.00%	k__Bacteria;p__Proteobacteria;c__Alphaproteobacteria
OTU_1377	2	3	0	0.00%	k__Bacteria;p__Actinobacteriota;c__Coriobacteriia;o__Coriobacteriales
OTU_1378	0	2	0	0.00%	k__Bacteria;p__Bacteroidota;c__Bacteroidia;o__Bacteroidales;f__Marinilabiliaceae;g__[Cytophaga]_xylanolytica_group;s__
OTU_1379	2	2	2	0.00%	k__Bacteria;p__Firmicutes;c__Clostridia;o__Oscillospirales;f__UCG-010;g__;s__
OTU_1380	0	0	0	0.00%	k__Bacteria;p__Bacteroidota;c__Bacteroidia;o__Flavobacteriales;f__Flavobacteriaceae;g__Flavobacterium;s__
OTU_1381	0	0	1	0.00%	k__Bacteria;p__Bacteroidota;c__Bacteroidia;o__Cytophagales;f__Spirosomaceae;g__Lacihabitans
OTU_1382	2	0	0	0.00%	k__Bacteria;p__Firmicutes;c__Clostridia;o__Peptostreptococcales-Tissierellales;f__Family_XI;g__Peptoniphilus;s__swine_effluent_bacterium_CHNDP10
OTU_1383	0	0	0	0.00%	Unknown
OTU_1384	0	0	0	0.00%	k__Bacteria;p__Proteobacteria;c__Gammaproteobacteria;o__Enterobacterales

OTU_1385	0	2	0	0.00%	k__Bacteria;p__Firmicutes;c__Negativicutes;o__Veillonellales-Selenomonadales;f__Veillonellaceae;g__Megasphaera;s__Megasphaera_cerevisiae
OTU_1386	0	0	0	0.00%	k__Bacteria;p__Proteobacteria;c__Gammaproteobacteria;o__Burkholderiales;f__Sutterellaceae;g__Sutterella;s__Sutterella_sp_6FBBBH3
OTU_1387	0	0	1	0.00%	k__Bacteria;p__Proteobacteria;c__Gammaproteobacteria;o__unidentified_Gammaproteobacteria;f__unidentified_Gammaproteobacteria;g__;s__
OTU_1388	0	0	0	0.00%	k__Bacteria;p__Bacteroidota;c__Bacteroidia;o__Sphingobacteriales;f__Sphingobacteriaceae;g__Pedobacter;s__
OTU_1389	0	0	2	0.00%	k__Bacteria;p__Proteobacteria;c__Gammaproteobacteria;o__Burkholderiales
OTU_1390	0	0	0	0.00%	k__Bacteria;p__Proteobacteria;c__Gammaproteobacteria;o__Xanthomonadales;f__Xanthomonadaceae;g__Arenimonas;s__Arenimonas_malthae
OTU_1391	3	1	0	0.00%	k__Bacteria;p__Actinobacteria;c__unidentified_Actinobacteria;o__Propionibacteriales;f__Propionibacteriaceae;g__Tessaracoccus;s__
OTU_1392	2	0	0	0.00%	k__Bacteria;p__Bacteroidota;c__Bacteroidia;o__Cytophagales;f__Hymenobacteraceae;g__Pontibacter;s__Pontibacter_sp
OTU_1393	1	0	0	0.00%	k__Bacteria;p__Firmicutes;c__Clostridia;o__Peptostreptococcales-Tissierellales;f__Family_XI;g__Tissierella;s__
OTU_1394	0	0	0	0.00%	k__Bacteria;p__Proteobacteria;c__Gammaproteobacteria;o__Pseudomonadales;f__Pseudomonadaceae;g__Pseudomonas
OTU_1395	2	2	2	0.00%	k__Bacteria;p__Bacteroidota;c__Bacteroidia;o__Bacteroidales;f__Paludibacteraceae;g__Paludibacter;s__
OTU_1396	1	0	3	0.00%	k__Bacteria;p__Desulfobacterota;c__Desulfuromonadia;o__Desulfuromonadales;f__Desulfuromonadaceae;g__Trichloromonas;s__
OTU_1397	0	2	0	0.00%	k__Bacteria;p__Proteobacteria;c__Alphaproteobacteria;o__Acetobacterales;f__Acetobacteraceae;g__Acidiphilium;s__
OTU_1398	0	0	0	0.00%	k__Bacteria
OTU_1399	168	192	166	0.27%	k__Bacteria
OTU_1400	2	0	1	0.00%	k__Bacteria;p__Bacteroidota;c__Bacteroidia;o__Cytophagales;f__Cyclobacteriaceae;g__Marinoscillum;s__
OTU_1401	2	0	0	0.00%	k__Bacteria;p__Firmicutes;c__Clostridia;o__Oscillospirales;f__[Eubacterium]_coprostanoligenes_group;g__;s__
OTU_1402	1	0	0	0.00%	k__Bacteria;p__Proteobacteria;c__Gammaproteobacteria;o__Burkholderiales;f__Chitinibacteraceae;g__Chitinilyticum;s__Chitinilyticum_aquatile
OTU_1403	0	0	2	0.00%	k__Bacteria;p__unidentified_Bacteria;c__Alphaproteobacteria;o__Rhizobiales;f__;g__;s__
OTU_1404	0	0	2	0.00%	k__Bacteria;p__Firmicutes;c__Clostridia
OTU_1405	0	0	0	0.00%	k__Bacteria;p__unidentified_Bacteria;c__Clostridia;o__Oscillospirales;f__Ruminococcaceae;g__unidentified_Ruminococcaceae;s__[Clostridium]_leptum
OTU_1406	0	0	1	0.00%	k__Bacteria;p__Firmicutes;c__Negativicutes;o__Veillonellales-Selenomonadales;f__Selenomonadaceae;g__Schwartzia;s__
OTU_1407	0	0	0	0.00%	k__Bacteria;p__unidentified_Bacteria;c__Acidimicrobiia;o__Microtrichales;f__Ilumatobacteraceae;g__;s__
OTU_1408	0	0	0	0.00%	k__Bacteria;p__Bacteroidota;c__Bacteroidia
OTU_1409	0	0	0	0.00%	k__Bacteria;p__Proteobacteria;c__Gammaproteobacteria;o__Burkholderiales;f__Neisseriaceae;g__Vitreoscilla;s__Neisseria_sp_BEG238
OTU_1410	1	0	0	0.00%	k__Bacteria;p__Bacteroidota;c__Bacteroidia;o__Bacteroidales;f__Paludibacteraceae
OTU_1411	0	1	0	0.00%	k__Bacteria;p__Proteobacteria;c__Gammaproteobacteria;o__Methylococcales;f__Methylomonadaceae;g__Methylovulum;s__Methylocucumis_oryzae
OTU_1412	0	0	0	0.00%	k__Bacteria;p__Bacteroidota;c__Bacteroidia;o__Sphingobacteriales;f__NS11-12_marine_group;g__;s__
OTU_1413	1	0	0	0.00%	k__Bacteria;p__Proteobacteria;c__Gammaproteobacteria;o__Legionellales;f__Legionellaceae;g__Legionella;s__
OTU_1414	2	2	2	0.00%	k__Bacteria;p__Bacteroidota;c__Bacteroidia;o__Bacteroidales;f__Prevotellaceae;g__Prevotellaceae_Ga6A1_group;s__
OTU_1415	7	1	0	0.00%	k__Bacteria
OTU_1416	0	2	0	0.00%	k__Bacteria;p__Bacteroidota;c__Bacteroidia;o__Bacteroidales;f__Bacteroidaceae;g__Bacteroides
OTU_1417	0	2	0	0.00%	k__Bacteria;p__Firmicutes;c__Negativicutes;o__Veillonellales-Selenomonadales;f__Veillonellaceae;g__Megasphaera
OTU_1418	0	0	0	0.00%	k__Bacteria;p__Desulfobacterota;c__Desulfobacteria;o__Desulfobacterales;f__Desulfobacteraceae;g__Desulfobotulus;s__Desulfobotulus_sapovorans
OTU_1419	0	0	0	0.00%	k__Bacteria;p__Bacteroidota;c__Bacteroidia;o__Bacteroidales;f__Prevotellaceae;g__Prevotella_7
OTU_1420	2	0	0	0.00%	k__Bacteria;p__Proteobacteria;c__Gammaproteobacteria;o__Competibacterales;f__Competibacteraceae;g__Plasticicumulans;s__
OTU_1421	0	0	3	0.00%	k__Bacteria;p__Bacteroidota;c__Bacteroidia;o__Chitinophagales;f__Chitinophagaceae
OTU_1422	1	0	3	0.00%	k__Bacteria;p__Myxococcota;c__Polyangia;o__Haliangiales;f__Haliangiaceae;g__Haliangium;s__
OTU_1423	2	0	0	0.00%	k__Bacteria;p__Firmicutes;c__Clostridia;o__Lachnospirales;f__Lachnospiraceae;g__Lachnospiraceae_NK4A136_group
OTU_1424	2	1	1	0.00%	k__Bacteria;p__Bacteroidota;c__Bacteroidia;o__Flavobacteriales;f__Flavobacteriaceae;g__Flavobacterium;s__
OTU_1425	0	0	2	0.00%	k__Bacteria;p__Desulfobacterota;c__Desulfobulbia;o__Desulfobulbales;f__Desulfocapsaceae;g__[Desulfobacterium]_catecholicum_group;s__
OTU_1426	0	1	0	0.00%	k__Bacteria;p__unidentified_Bacteria;c__Verrucomicrobiae;o__Verrucomicrobiales;f__Verrucomicrobiaceae;g__;s__
OTU_1427	0	4	0	0.00%	k__Bacteria;p__Actinobacteriota;c__Thermoleophilia;o__Gaiellales;f__Gaiellaceae;g__Gaiella;s__
OTU_1428	0	0	3	0.00%	k__Bacteria;p__Cyanobacteria;c__Sericytochromatia;o__;f__;g__;s__
OTU_1429	4	0	0	0.00%	k__Bacteria;p__Firmicutes;c__Bacilli;o__Bacillales;f__Bacillaceae;g__Bacillus;s__Bacillus_funiculus
OTU_1430	5	5	2	0.01%	k__Bacteria;p__Firmicutes;c__Negativicutes;o__Veillonellales-Selenomonadales;f__Selenomonadaceae;g__Selenomonas;s__Selenomonas_lacticifex
OTU_1431	0	0	0	0.00%	k__Bacteria;p__unidentified_Bacteria;c__Bacteroidia;o__Chitinophagales;f__Chitinophagaceae;g__;s__
OTU_1432	2	2	1	0.00%	k__Bacteria;p__unidentified_Bacteria;c__Bacteroidia;o__Bacteroidales;f__Barnesiellaceae;g__unidentified_Barnesiellaceae;s__Bacteroidales_bacterium_36-12
OTU_1433	0	0	0	0.00%	k__Bacteria;p__Proteobacteria;c__Gammaproteobacteria;o__Legionellales;f__Legionellaceae;g__Legionella;s__
OTU_1434	1	2	1	0.00%	k__Bacteria;p__Firmicutes;c__Clostridia;o__Oscillospirales;f__Butyricicoccaceae;g__Butyricicoccus;s__
OTU_1435	0	0	0	0.00%	k__Bacteria;p__Firmicutes;c__Clostridia;o__Clostridia_vadinBB60_group;f__;g__;s__
OTU_1436	0	1	1	0.00%	k__Bacteria;p__Firmicutes;c__Clostridia;o__Lachnospirales;f__Lachnospiraceae;g__Lachnospiraceae_NK3A20_group;s__
OTU_1437	1	3	0	0.00%	k__Bacteria;p__Proteobacteria;c__Alphaproteobacteria;o__Rhizobiales;f__Devosiaceae;g__Devosia;s__Devosia_sp
OTU_1438	2	4	11	0.01%	k__Bacteria;p__Proteobacteria;c__Gammaproteobacteria;o__Thiotrichales;f__Thiotrichaceae;g__Thiothrix;s__Thiothrix_eikelboomii
OTU_1439	0	0	2	0.00%	k__Bacteria;p__Actinobacteria;c__unidentified_Actinobacteria;o__Micrococcales;f__Microbacteriaceae;g__Aurantimicrobium;s__
OTU_1440	0	0	4	0.00%	k__Bacteria;p__Bacteroidota;c__Bacteroidia;o__Bacteroidales;f__Prevotellaceae

OTU_1441	0	3	0	0.00%	k__Bacteria
OTU_1442	0	1	2	0.00%	k__Bacteria;p__Gracilibacteria;c__unidentified_Gracilibacteria;o__Absconditabacteriales_(SR1);f__;g__;s__
OTU_1443	0	0	0	0.00%	k__Bacteria;p__Gracilibacteria;c__unidentified_Gracilibacteria;o__Absconditabacteriales_(SR1);f__;g__;s__
OTU_1444	0	2	0	0.00%	k__Bacteria;p__Firmicutes;c__Bacilli;o__RsaHf231;f__;g__;s__
OTU_1445	1	0	1	0.00%	k__Bacteria;p__unidentified_Bacteria;c__Alphaproteobacteria;o__Paracaedibacterales;f__Paracaedibacteraceae;<br>g__unidentified_Paracaedibacteraceae;s__Candidatus_Intestinusbacter_nucleariae
OTU_1446	0	0	1	0.00%	k__Bacteria;p__Firmicutes;c__Clostridia;o__Clostridia_UCG-014;f__;g__;s__
OTU_1447	1	0	1	0.00%	k__Bacteria;p__unidentified_Bacteria;c__Bacteroidia;o__Bacteroidales;f__Paludibacteraceae;g__;s__
OTU_1448	4	1	0	0.00%	k__Bacteria;p__Bacteroidota;c__Bacteroidia;o__Cytophagales;f__Hymenobacteraceae;g__Adhaeribacter
OTU_1449	0	1	1	0.00%	k__Bacteria;p__unidentified_Bacteria;c__Bacteroidia;o__Bacteroidales;f__;g__;s__
OTU_1450	0	0	3	0.00%	k__Bacteria;p__Firmicutes;c__Clostridia;o__Lachnospirales;f__Lachnospiraceae
OTU_1451	1	0	0	0.00%	k__Bacteria
OTU_1452	0	0	0	0.00%	k__Bacteria
OTU_1453	164	149	167	0.24%	k__Bacteria;p__Proteobacteria;c__Gammaproteobacteria;o__Pseudomonadales;f__Moraxellaceae;g__Acinetobacter
OTU_1454	0	0	0	0.00%	k__Bacteria;p__Proteobacteria;c__Gammaproteobacteria;o__Pseudomonadales;f__Moraxellaceae;g__Acinetobacter
OTU_1455	2	0	0	0.00%	k__Bacteria;p__unidentified_Bacteria;c__unidentified_Bacteria;o__Gemmatimonadales;f__Gemmatimonadaceae;g__;s__
OTU_1456	0	1	0	0.00%	k__Bacteria;p__unidentified_Bacteria;c__Clostridia;o__Oscillospirales;f__Butyricicoccaceae;g__;s__
OTU_1457	0	0	0	0.00%	k__Bacteria
OTU_1458	2	1	2	0.00%	k__Bacteria;p__Proteobacteria;c__Gammaproteobacteria;o__Burkholderiales
OTU_1459	1	1	0	0.00%	k__Bacteria;p__Hydrogenedentes;c__Hydrogenedentia;o__Hydrogenedentiales;f__Hydrogenedensaceae;g__;s__
OTU_1460	6	2	4	0.01%	k__Bacteria
OTU_1461	2	0	0	0.00%	k__Bacteria;p__Proteobacteria;c__Gammaproteobacteria;o__Competibacterales;f__Competibacteraceae;g__Candidatus_Competibacter;s__
OTU_1462	0	0	0	0.00%	k__Bacteria;p__Gracilibacteria;c__unidentified_Gracilibacteria;o__JGI_0000069-P22
OTU_1463	0	0	0	0.00%	k__Bacteria;p__Firmicutes;c__Clostridia;o__Clostridia_UCG-014;f__;g__;s__
OTU_1464	1	0	0	0.00%	k__Bacteria;p__Firmicutes;c__Clostridia;o__Oscillospirales;f__Oscillospiraceae;g__Colidextribacter
OTU_1465	125	107	157	0.20%	k__Bacteria;p__Proteobacteria;c__Gammaproteobacteria;o__Pseudomonadales;f__Moraxellaceae;g__Acinetobacter
OTU_1466	0	1	0	0.00%	k__Bacteria;p__Firmicutes;c__Clostridia;o__Lachnospirales;f__Lachnospiraceae
OTU_1467	0	0	1	0.00%	k__Bacteria;p__Firmicutes;c__Negativicutes;o__Veillonellales-Selenomonadales;f__Sporomusaceae;g__Pelosinus;s__
OTU_1468	3	0	0	0.00%	k__Bacteria;p__Proteobacteria;c__Gammaproteobacteria;o__unidentified_Gammaproteobacteria;f__unidentified_Gammaproteobacteria;g__;s__
OTU_1469	0	3	1	0.00%	k__Bacteria;p__Synergistota;c__Synergistia;o__Synergistales;f__Synergistaceae;g__Cloacibacillus;s__
OTU_1470	0	0	0	0.00%	k__Bacteria;p__Proteobacteria;c__Alphaproteobacteria;o__Rickettsiales;f__Mitochondria;g__;s__
OTU_1471	0	0	0	0.00%	k__Bacteria;p__Firmicutes;c__Bacilli;o__RF39;f__;g__;s__
OTU_1472	0	0	2	0.00%	k__Bacteria;p__unidentified_Bacteria;c__Fibrobacteria;o__Fibrobacterales;f__Fibrobacteraceae;g__;s__
OTU_1473	0	0	0	0.00%	k__Bacteria;p__Cyanobacteria;c__Vampirivibrionia;o__Gastranaerophilales;f__;g__;s__
OTU_1474	0	0	2	0.00%	k__Bacteria;p__Firmicutes;c__Clostridia;o__Christensenellales;f__Christensenellaceae;g__Christensenellaceae_R-7_group;s__
OTU_1475	133	190	163	0.25%	k__Bacteria;p__Proteobacteria;c__Gammaproteobacteria;o__Enterobacterales;f__Enterobacteriaceae
OTU_1476	28	49	37	0.06%	k__Bacteria;p__Firmicutes;c__Clostridia;o__Lachnospirales;f__Lachnospiraceae
OTU_1477	0	0	0	0.00%	k__Bacteria;p__Firmicutes;c__Clostridia;o__Lachnospirales;f__Lachnospiraceae;g__[Eubacterium]_xylanophilum_group;s__
OTU_1478	4	0	0	0.00%	k__Bacteria;p__Cyanobacteria;c__Sericytochromatia;o__;f__;g__;s__
OTU_1479	2	0	0	0.00%	k__Bacteria
OTU_1480	2	0	0	0.00%	k__Bacteria;p__Firmicutes;c__Clostridia;o__Oscillospirales;f__[Eubacterium]_coprostanoligenes_group;g__;s__
OTU_1481	285	140	266	0.35%	k__Bacteria;p__Proteobacteria;c__Gammaproteobacteria;o__Burkholderiales;f__Comamonadaceae;g__Hydrogenophaga
OTU_1482	1	1	2	0.00%	k__Bacteria;p__Proteobacteria;c__Gammaproteobacteria;o__Xanthomonadales;f__Xanthomonadaceae;g__Thermomonas;s__Thermomonas_fusca
OTU_1483	1	1	0	0.00%	k__Bacteria;p__Desulfobacterota;c__Desulfovibrionia;o__Desulfovibrionales;f__Desulfovibrionaceae;g__Desulfovibrio;s__bacterium_S4531
OTU_1484	3	0	0	0.00%	k__Bacteria;p__Kapabacteria;c__unidentified_Kapabacteria;o__Kapabacteriales;f__;g__;s__
OTU_1485	0	3	0	0.00%	k__Bacteria;p__Myxococcota;c__Polyangia;o__Polyangiales;f__Polyangiaceae;g__Pajaroellobacter;s__
OTU_1486	0	0	2	0.00%	k__Bacteria;p__Bacteroidota;c__Bacteroidia;o__Chitinophagales;f__Chitinophagaceae;g__Taibaiella;s__
OTU_1487	1	1	0	0.00%	k__Bacteria;p__Firmicutes;c__Negativicutes;o__Veillonellales-Selenomonadales;f__Veillonellaceae;g__Megasphaera;s__
OTU_1488	0	0	0	0.00%	k__Bacteria
OTU_1489	0	0	3	0.00%	k__Bacteria;p__Proteobacteria;c__Gammaproteobacteria;o__Burkholderiales;f__Chitinibacteraceae;g__Formivibrio;s__
OTU_1490	0	0	0	0.00%	k__Bacteria;p__Firmicutes;c__Bacilli;o__RF39;f__;g__;s__
OTU_1491	0	0	0	0.00%	k__Bacteria;p__Bacteroidota;c__Bacteroidia;o__Bacteroidales;f__Bacteroidaceae;g__Bacteroides
OTU_1492	2	0	0	0.00%	k__Bacteria;p__Proteobacteria;c__Alphaproteobacteria;o__Rhizobiales
OTU_1493	0	2	1	0.00%	k__Bacteria;p__Myxococcota;c__Polyangia;o__Nannocystales;f__Nannocystaceae
OTU_1494	1	0	0	0.00%	k__Bacteria;p__Firmicutes;c__Clostridia;o__Peptostreptococcales-Tissierellales

OTU_1495	2	0	0	0.00%	k__Bacteria;p__Proteobacteria;c__Gammaproteobacteria;o__Burkholderiales
OTU_1496	0	0	0	0.00%	k__Bacteria
OTU_1497	2	4	0	0.00%	k__Bacteria;p__Proteobacteria;c__Gammaproteobacteria;o__Xanthomonadales;f__Xanthomonadaceae;g__Arenimonas
OTU_1498	7	1	1	0.00%	k__Bacteria;p__Actinobacteria;c__unidentified_Actinobacteria;o__Micrococcales;f__Cellulomonadaceae;g__Actinotalea;s__
OTU_1499	0	0	0	0.00%	k__Bacteria;p__Proteobacteria;c__Gammaproteobacteria;o__Pseudomonadales;f__Porticoccaceae;g__C1-B045
OTU_1500	0	1	0	0.00%	k__Bacteria;p__Proteobacteria;c__Gammaproteobacteria;o__Burkholderiales;f__Nitrosomonadaceae;g__MND1;s__
OTU_1501	0	0	3	0.00%	k__Bacteria;p__Firmicutes;c__Bacilli;o__Erysipelotrichales;f__Erysipelotrichaceae;g__Faecalibaculum;s__Faecalibaculum_rodentium
OTU_1502	2	0	0	0.00%	k__Bacteria;p__Myxococcota;c__Polyangia;o__Haliangiales;f__Haliangiaceae;g__Haliangium;s__
OTU_1503	0	0	0	0.00%	k__Bacteria;p__Firmicutes;c__Clostridia;o__Clostridia_UCG-014;f__;g__;s__
OTU_1504	2	0	1	0.00%	k__Bacteria;p__unidentified_Bacteria;c__Alphaproteobacteria;o__Rhodospirillales;f__;g__;s__
OTU_1505	2	0	0	0.00%	k__Bacteria;p__Proteobacteria;c__Alphaproteobacteria;o__Rhizobiales;f__Xanthobacteraceae;g__Pseudorhodoplanes
OTU_1506	4	3	0	0.00%	k__Bacteria;p__Proteobacteria;c__Alphaproteobacteria;o__Reyranellales;f__Reyranellaceae;g__Reyranella;s__
OTU_1507	358	145	343	0.43%	k__Bacteria;p__Proteobacteria;c__Gammaproteobacteria;o__Burkholderiales;f__Comamonadaceae
OTU_1508	1	0	1	0.00%	k__Bacteria;p__Kapabacteria;c__unidentified_Kapabacteria;o__Kapabacteriales;f__unidentified_Kapabacteriales;g__unidentified_Kapabacteriales
OTU_1509	0	0	0	0.00%	k__Bacteria;p__Bdellovibrionota;c__Bdellovibrionia;o__Bdellovibrionales;f__Bdellovibrionaceae;g__Bdellovibrio
OTU_1510	0	0	0	0.00%	k__Bacteria;p__unidentified_Bacteria;c__Bacilli;o__Erysipelotrichales;f__Erysipelotrichaceae;g__;s__
OTU_1511	0	0	0	0.00%	k__Bacteria;p__unidentified_Bacteria;c__Bacteroidia;o__Chitinophagales;f__Saprospiraceae;g__unidentified_Saprospiraceae;s__bacterium_enrichment_culture_clone_SRAO_34
OTU_1512	0	0	2	0.00%	k__Bacteria;p__Actinobacteria;c__unidentified_Actinobacteria;o__Frankiales;f__Nakamurellaceae;g__Nakamurella;s__
OTU_1513	0	0	0	0.00%	k__Bacteria;p__Firmicutes;c__Clostridia;o__Oscillospirales;f__Ruminococcaceae;g__Ruminococcus;s__
OTU_1514	0	2	1	0.00%	k__Bacteria;p__Actinobacteria;c__unidentified_Actinobacteria;o__Micromonosporales;f__Micromonosporaceae;g__Micromonospora;s__Micromonospora_coxensis
OTU_1515	0	0	0	0.00%	k__Bacteria;p__Bacteroidota;c__Bacteroidia;o__Bacteroidales;f__Prolixibacteraceae;g__Roseimarinus;s__
OTU_1516	0	0	0	0.00%	k__Bacteria;p__Bdellovibrionota;c__Bdellovibrionia;o__Bdellovibrionales;f__Bdellovibrionaceae;g__Bdellovibrio;s__
OTU_1517	0	0	2	0.00%	k__Bacteria;p__Bacteroidota;c__Bacteroidia
OTU_1518	0	3	0	0.00%	k__Bacteria;p__Proteobacteria;c__Gammaproteobacteria;o__Enterobacterales
OTU_1519	0	2	0	0.00%	k__Bacteria;p__Bacteroidota;c__Bacteroidia;o__Cytophagales;f__Hymenobacteraceae;g__Hymenobacter;s__Hymenobacter_gelipurpurascens
OTU_1520	0	0	2	0.00%	k__Bacteria;p__Proteobacteria;c__Gammaproteobacteria;o__Enterobacterales;f__Alteromonadaceae;g__Rheinheimera
OTU_1521	0	0	2	0.00%	k__Bacteria;p__unidentified_Bacteria;c__Bacteroidia;o__Chitinophagales;f__;g__;s__
OTU_1522	0	0	0	0.00%	k__Bacteria;p__SAR324_clade(Marine_group_B);c__;o__;f__;g__;s__
OTU_1523	5	0	0	0.00%	k__Bacteria;p__unidentified_Bacteria;c__Campylobacteria;o__Campylobacterales;f__Arcobacteraceae;g__;s__
OTU_1524	0	0	0	0.00%	k__Bacteria
OTU_1525	2	0	0	0.00%	k__Bacteria;p__Acidobacteriota;c__Acidobacteriae;o__Bryobacterales;f__Bryobacteraceae;g__Bryobacter;s__
OTU_1526	0	2	0	0.00%	k__Bacteria;p__Firmicutes;c__Clostridia;o__Lachnospirales;f__Lachnospiraceae
OTU_1527	0	0	0	0.00%	k__Bacteria;p__Firmicutes;c__Clostridia;o__Oscillospirales;f__[Clostridium]_methylpentosum_group;g__;s__
OTU_1528	3	0	0	0.00%	k__Bacteria;p__Firmicutes;c__Clostridia;o__Christensenellales;f__Christensenellaceae;g__Christensenellaceae_R-7_group
OTU_1529	2	0	0	0.00%	k__Bacteria;p__Proteobacteria;c__Alphaproteobacteria;o__Rhizobiales;f__Beijerinckiaceae;g__Bosea;s__
OTU_1530	0	0	0	0.00%	k__Bacteria;p__unidentified_Bacteria;c__Bacteroidia;o__Bacteroidales;f__Prolixibacteraceae;g__;s__
OTU_1531	0	2	0	0.00%	k__Bacteria
OTU_1532	0	0	0	0.00%	k__Bacteria;p__Proteobacteria;c__Alphaproteobacteria;o__Reyranellales;f__Reyranellaceae;g__Reyranella;s__
OTU_1533	0	0	0	0.00%	k__Bacteria;p__Firmicutes;c__Clostridia
OTU_1534	0	2	0	0.00%	k__Bacteria;p__Proteobacteria;c__Alphaproteobacteria;o__Rhizobiales;f__Xanthobacteraceae;g__Pseudoxanthobacter;s__
OTU_1535	0	0	0	0.00%	k__Bacteria;p__Firmicutes;c__Negativicutes;o__Veillonellales-Selenomonadales;f__Selenomonadaceae;g__Selenomonas
OTU_1536	0	4	0	0.00%	k__Bacteria;p__Actinobacteria;c__unidentified_Actinobacteria;o__Pseudonocardiales;f__Pseudonocardiaceae;g__Pseudonocardia;s__
OTU_1537	0	2	0	0.00%	k__Bacteria;p__Acidobacteriota;c__Holophagae;o__Subgroup_7;f__;g__;s__
OTU_1538	0	1	2	0.00%	k__Bacteria;p__Desulfobacterota;c__Desulfovibrionia;o__Desulfovibrionales;f__Desulfovibrionaceae;g__Desulfovibrio
OTU_1539	0	0	0	0.00%	k__Bacteria;p__unidentified_Bacteria;c__Bacteroidia;o__Bacteroidales;f__Marinilabiliaceae;g__;s__
OTU_1540	0	0	0	0.00%	k__Bacteria;p__Firmicutes;c__Bacilli;o__Erysipelotrichales;f__Erysipelotrichaceae;g__Faecalitalea;s__Absiella_argi
OTU_1541	3	0	0	0.00%	k__Bacteria;p__unidentified_Bacteria;c__Alphaproteobacteria;o__Micavibrionales;f__Micavibrionaceae;g__;s__
OTU_1542	0	1	4	0.00%	k__Bacteria;p__Firmicutes;c__Clostridia;o__Oscillospirales;f__Butyricicoccaceae;g__UCG-009;s__
OTU_1543	5	1	0	0.00%	k__Bacteria;p__Myxococcota;c__Myxococcia;o__Myxococcales;f__Myxococcaceae
OTU_1544	0	0	0	0.00%	k__Bacteria;p__Firmicutes;c__Clostridia;o__Peptostreptococcales-Tissierellales;f__Acidaminobacteraceae;g__Acidaminobacter;s__
OTU_1545	0	0	3	0.00%	k__Bacteria;p__unidentified_Bacteria;c__Bacteroidia;o__Bacteroidales;f__Marinilabiliaceae;g__;s__
OTU_1546	0	1	0	0.00%	k__Bacteria;p__unidentified_Bacteria;c__Bacteroidia;o__Bacteroidales;f__Dysgonomonadaceae;g__;s__
OTU_1547	0	2	0	0.00%	k__Bacteria;p__Proteobacteria;c__Alphaproteobacteria;o__Rickettsiales;f__SM2D12;g__;s__
OTU_1548	1	1	2	0.00%	k__Bacteria;p__Firmicutes;c__Clostridia;o__Oscillospirales;f__UCG-010;g__;s__
OTU_1549	0	0	0	0.00%	k__Bacteria;p__Bacteroidota;c__Bacteroidia;o__Bacteroidales;f__Williamwhitmaniaceae;g__Blvii28_wastewater-sludge_group;s__
OTU_1550	1	1	2	0.00%	k__Bacteria;p__unidentified_Bacteria;c__unidentified_Bacteria;o__Actinomycetales;f__Actinomycetaceae;g__;s__

OTU_1551	0	0	2	0.00%	k__Bacteria;p__unidentified_Bacteria;c__Acidimicrobiia;o__Actinomarinales;f__;g__;s__
OTU_1552	1	0	1	0.00%	k__Bacteria;p__unidentified_Bacteria;c__Gammaproteobacteria;o__Xanthomonadales;f__Rhodanobacteraceae;g__;s__
OTU_1553	2	0	0	0.00%	k__Bacteria;p__Actinobacteriota;c__Rubrobacteria;o__Rubrobacterales;f__Rubrobacteriaceae;g__Rubrobacter;s__
OTU_1554	1	0	1	0.00%	k__Bacteria;p__Gracilibacteria
OTU_1555	2	0	0	0.00%	k__Bacteria;p__Proteobacteria;c__Gammaproteobacteria;o__Pseudomonadales;f__Pseudohongiellaceae;g__Pseudohongiella
OTU_1556	2	3	1	0.00%	k__Bacteria;p__Proteobacteria;c__Alphaproteobacteria;o__Rhodobacterales;f__Rhodobacteraceae;g__Thioclava;s__
OTU_1557	34	9	26	0.04%	k__Bacteria;p__Proteobacteria;c__Gammaproteobacteria;o__Burkholderiales;f__Comamonadaceae
OTU_1558	1	0	1	0.00%	k__Bacteria;p__Bacteroidota;c__Bacteroidia;o__Chitinophagales;f__Chitinophagaceae;g__Edaphobaculum;s__
OTU_1559	2	0	0	0.00%	k__Bacteria;p__unidentified_Bacteria;c__Bacteroidia;o__Cytophagales;f__Cyclobacteriaceae
OTU_1560	0	0	0	0.00%	k__Bacteria;p__unidentified_Bacteria;c__Bacteroidia;o__Bacteroidales;f__Paludibacteraceae;g__;s__
OTU_1561	0	2	0	0.00%	k__Bacteria;p__Chloroflexi;c__Chloroflexia;o__Kallotenuales;f__AKIW781;g__;s__
OTU_1562	2	0	0	0.00%	k__Bacteria;p__Proteobacteria;c__Gammaproteobacteria;o__Burkholderiales;f__Comamonadaceae
OTU_1563	1	1	0	0.00%	k__Bacteria;p__Synergistota;c__Synergistia;o__Synergistales;f__Synergistaceae
OTU_1564	2	0	0	0.00%	k__Bacteria;p__Spirochaetota;c__Spirochaetia;o__Spirochaetales;f__Spirochaetaceae;g__Treponema
OTU_1565	0	0	2	0.00%	k__Bacteria;p__Firmicutes;c__Negativicutes;o__Veillonellales-Selenomonadales;f__Veillonellaceae;g__Dialister
OTU_1566	0	0	3	0.00%	k__Bacteria;p__Verrucomicrobiota;c__Verrucomicrobiae;o__Verrucomicrobiales;f__Rubritaleaceae;g__Luteolibacter;s__Verrucomicrobia_bacterium_SCGC_AAA027-I19
OTU_1567	1	0	0	0.00%	k__Bacteria;p__unidentified_Bacteria;c__Bacteroidia;o__Chitinophagales;f__Saprospiraceae;g__;s__
OTU_1568	0	1	0	0.00%	k__Bacteria;p__Synergistota;c__Synergistia;o__Synergistales;f__Synergistaceae;g__Thermovirga;s__
OTU_1569	0	0	2	0.00%	k__Bacteria;p__Bacteroidota;c__Bacteroidia;o__Bacteroidales;f__Rikenellaceae;g__dgA-11_gut_group;s__
OTU_1570	0	1	1	0.00%	k__Bacteria;p__Bacteroidota;c__Bacteroidia;o__Flavobacteriales;f__Flavobacteriaceae;g__Flavobacterium
OTU_1571	0	0	4	0.00%	k__Bacteria;p__unidentified_Bacteria;c__Thermoleophilia;o__Solirubrobacterales;f__Solirubrobacteraceae;g__unidentified_Solirubrobacteraceae;s__Solirubrobacterales_bacterium
OTU_1572	0	1	4	0.00%	k__Bacteria;p__Actinobacteriota;c__Rubrobacteria;o__Rubrobacterales;f__Rubrobacteriaceae;g__Rubrobacter;s__
OTU_1573	0	2	0	0.00%	k__Bacteria;p__Firmicutes;c__Clostridia;o__Lachnospirales;f__Lachnospiraceae
OTU_1574	0	0	2	0.00%	k__Bacteria;p__Firmicutes;c__Clostridia;o__Peptostreptococcales-Tissierellales;f__Family_XI;g__Anaerococcus;s__
OTU_1575	0	0	0	0.00%	k__Bacteria;p__Proteobacteria;c__Gammaproteobacteria;o__Burkholderiales;f__Rhodocyclaceae
OTU_1576	2	0	0	0.00%	k__Bacteria;p__Firmicutes;c__Clostridia;o__Oscillospirales;f__Ruminococcaceae;g__Pygmaiobacter;s__
OTU_1577	2	0	0	0.00%	k__Bacteria;p__Bacteroidota;c__Bacteroidia;o__Cytophagales;f__Cytophagaceae;g__Cytophaga;s__
OTU_1578	0	0	0	0.00%	k__Bacteria
OTU_1579	0	0	2	0.00%	k__Bacteria;p__Gracilibacteria;c__unidentified_Gracilibacteria;o__Absconditabacteriales_(SR1)
OTU_1580	2	0	0	0.00%	k__Bacteria;p__Bacteroidota;c__Bacteroidia;o__Cytophagales;f__Hymenobacteraceae;g__Hymenobacter
OTU_1581	0	0	2	0.00%	k__Bacteria;p__Proteobacteria;c__Gammaproteobacteria;o__Burkholderiales;f__Comamonadaceae
OTU_1582	0	0	2	0.00%	k__Bacteria;p__Bdellovibrionota;c__Bdellovibrionia;o__Bdellovibrionales;f__Bdellovibrionaceae;g__Bdellovibrio;s__
OTU_1583	0	1	1	0.00%	k__Bacteria;p__Bacteroidota;c__Bacteroidia;o__Bacteroidales;f__Rikenellaceae;g__U29-B03;s__
OTU_1584	0	2	0	0.00%	k__Bacteria;p__Proteobacteria;c__Alphaproteobacteria;o__Azospirillales;f__Azospirillaceae;g__Desertibacter;s__Desertibacter_xinjiangensis
OTU_1585	0	1	2	0.00%	k__Bacteria;p__Firmicutes;c__Clostridia;o__Peptostreptococcales-Tissierellales;f__Family_XI;g__Gallicola;s__
OTU_1586	0	0	0	0.00%	k__Bacteria;p__Verrucomicrobiota;c__Verrucomicrobiae;o__Opitutales;f__Opitutaceae;g__Lacunisphaera;s__
OTU_1587	2	0	0	0.00%	k__Bacteria;p__unidentified_Bacteria;c__Alphaproteobacteria
OTU_1588	0	0	0	0.00%	k__Bacteria;p__Firmicutes;c__Clostridia;o__Clostridia_UCG-014;f__;g__;s__
OTU_1589	2	3	1	0.00%	k__Bacteria;p__unidentified_Bacteria;c__Coriobacteriia;o__Coriobacteriales;f__;g__;s__
OTU_1590	2	0	1	0.00%	k__Bacteria;p__Synergistota;c__Synergistia;o__Synergistales;f__Synergistaceae;g__Candidatus_Tammella;s__
OTU_1591	0	0	1	0.00%	k__Bacteria;p__Firmicutes;c__Clostridia;o__Oscillospirales;f__UCG-010;g__;s__
OTU_1592	0	2	0	0.00%	k__Bacteria;p__Proteobacteria;c__Gammaproteobacteria;o__Burkholderiales;f__Alcaligenaceae;g__Parapusillimonas;s__
OTU_1593	2	0	0	0.00%	k__Bacteria;p__Bacteroidota;c__Bacteroidia;o__Bacteroidales;f__M2PB4-65_termite_group;g__;s__
OTU_1594	2	1	2	0.00%	k__Bacteria;p__Firmicutes;c__Clostridia;o__Lachnospirales;f__Lachnospiraceae
OTU_1595	0	2	0	0.00%	k__Bacteria;p__Cyanobacteria;c__Cyanobacteriia;o__unidentified_Cyanobacteriia;f__unidentified_Cyanobacteriia;g__Leptolyngbya_EcFYyyy-00;s__
OTU_1596	0	2	1	0.00%	k__Bacteria;p__Proteobacteria;c__Gammaproteobacteria;o__unidentified_Gammaproteobacteria;f__unidentified_Gammaproteobacteria;g__Candidatus_Ovatusbacter;s__
OTU_1597	0	0	0	0.00%	k__Bacteria;p__Bacteroidota;c__Bacteroidia;o__Sphingobacteriales;f__S15A-MN91;g__;s__
OTU_1598	0	0	4	0.00%	k__Bacteria;p__Proteobacteria;c__Alphaproteobacteria;o__Caedibacterales;f__Caedibacteraceae;g__Candidatus_Nucleicultrix;s__
OTU_1599	0	0	0	0.00%	k__Bacteria;p__Campylobacterota;c__Campylobacteria;o__Campylobacterales;f__Helicobacteraceae;g__Helicobacter
OTU_1600	0	0	0	0.00%	k__Bacteria;p__Proteobacteria;c__Gammaproteobacteria;o__Burkholderiales;f__Nitrosomonadaceae;g__Nitrosomonas
OTU_1601	2	0	0	0.00%	k__Bacteria;p__Bacteroidota;c__Bacteroidia;o__Bacteroidales;f__Dysgonomonadaceae;g__Proteiniphilum;s__
OTU_1602	4	0	0	0.00%	k__Bacteria;p__Bdellovibrionota;c__Oligoflexia;o__0319-6G20
OTU_1603	0	0	0	0.00%	k__Bacteria;p__Proteobacteria;c__Gammaproteobacteria;o__Enterobacterales;f__Psychromonadaceae;g__Psychromonas;s__
OTU_1604	0	0	0	0.00%	k__Bacteria;p__Proteobacteria;c__Gammaproteobacteria;o__Competibacterales;f__Competibacteraceae;g__Candidatus_Competibacter;s__
OTU_1605	4	0	0	0.00%	k__Bacteria;p__Cyanobacteria;c__Vampirivibrionia;o__Obscuribacterales;f__Obscuribacteraceae;g__;s__
OTU_1606	0	2	0	0.00%	k__Bacteria;p__Deinococcota;c__Deinococci;o__Deinococcales;f__Deinococcaceae;g__Deinococcus;s__Deinococcus_hohokamensis

OTU_1607	2	0	0	0.00%	k__Bacteria;p__Firmicutes;c__Clostridia;o__Clostridiales;f__Clostridiaceae;g__Clostridium_sensu_stricto_11
OTU_1608	0	0	1	0.00%	k__Bacteria;p__unidentified_Bacteria;c__Bacteroidia;o__Chitinophagales;f__;g__;s__
OTU_1609	3	0	0	0.00%	k__Bacteria;p__Firmicutes;c__Clostridia;o__Oscillospirales;f__UCG-010;g__;s__
OTU_1610	7	2	0	0.00%	k__Bacteria;p__Firmicutes;c__Bacilli;o__Bacillales;f__Bacillaceae;g__Bacillus;s__Bacillus_niabensis
OTU_1611	0	0	2	0.00%	k__Bacteria;p__Firmicutes;c__Clostridia;o__Oscillospirales
OTU_1612	0	2	0	0.00%	k__Bacteria;p__unidentified_Bacteria;c__Bacteroidia;o__Bacteroidales;f__Rikenellaceae;g__;s__
OTU_1613	5	3	5	0.01%	k__Bacteria;p__Proteobacteria;c__Gammaproteobacteria;o__Enterobacterales;f__Succinivibrionaceae;g__Succinivibrio;s__
OTU_1614	11	13	9	0.02%	k__Bacteria;p__Proteobacteria;c__Gammaproteobacteria;o__Pseudomonadales;f__Pseudomonadaceae;g__Pseudomonas;s__Pseudomonas_sp_C23
OTU_1615	0	1	0	0.00%	k__Bacteria;p__Verrucomicrobiota;c__Verrucomicrobiae;o__Opitutales;f__Opitutaceae;g__Cephaloticoccus;s__
OTU_1616	0	3	0	0.00%	k__Bacteria;p__Gemmatimonadetes;c__unidentified_Gemmatimonadetes;o__Gemmatimonadales;f__Gemmatimonadaceae;g__Gemmatimonas;s__
OTU_1617	0	0	2	0.00%	k__Bacteria;p__Bacteroidota;c__Bacteroidia;o__Bacteroidales;f__Tannerellaceae
OTU_1618	1	2	1	0.00%	k__Bacteria;p__Firmicutes;c__Clostridia;o__Oscillospirales;f__Oscillospiraceae;g__NK4A214_group;s__
OTU_1619	0	1	1	0.00%	k__Bacteria;p__Gracilibacteria;c__unidentified_Gracilibacteria;o__Absconditabacteriales_(SR1);f__;g__;s__
OTU_1620	0	3	0	0.00%	k__Bacteria;p__Bacteroidota;c__Bacteroidia;o__Bacteroidales;f__Rikenellaceae;g__Alistipes;s__Alistipes_sp_N15.MGS-157
OTU_1621	0	2	0	0.00%	k__Bacteria;p__Actinobacteria;c__unidentified_Actinobacteria;o__0319-7L14;f__;g__;s__
OTU_1622	0	2	0	0.00%	k__Bacteria;p__FCPU426;c__;o__;f__;g__;s__
OTU_1623	161	167	146	0.24%	k__Bacteria;p__Proteobacteria;c__Gammaproteobacteria;o__Enterobacterales;f__Aeromonadaceae;g__Aeromonas
OTU_1624	0	3	0	0.00%	k__Bacteria
OTU_1625	0	2	0	0.00%	k__Bacteria;p__Actinobacteriota;c__Coriobacteriia;o__Coriobacteriales;f__Atopobiaceae;g__Olsenella;s__Olsenella_sp_oral_taxon_807
OTU_1626	1	2	0	0.00%	k__Bacteria;p__Proteobacteria;c__Alphaproteobacteria;o__Rhizobiales;f__Rhizobiaceae
OTU_1627	2	0	0	0.00%	k__Bacteria;p__Gemmatimonadetes;c__unidentified_Gemmatimonadetes;o__Gemmatimonadales;f__Gemmatimonadaceae;g__Gemmatimonas;s__
OTU_1628	0	1	0	0.00%	k__Bacteria;p__unidentified_Bacteria;c__Alphaproteobacteria;o__Rhodospirillales;f__unidentified_Rhodospirillales;g__unidentified_Rhodospirillales;s__Azospirillum_sp_47_25
OTU_1629	0	3	0	0.00%	k__Bacteria;p__Chloroflexi;c__Anaerolineae;o__RBG-13-54-9;f__;g__;s__
OTU_1630	0	0	2	0.00%	k__Bacteria;p__Firmicutes;c__Clostridia;o__Lachnospirales;f__Lachnospiraceae;g__Butyrivibrio;s__Butyrivibrio_crossotus
OTU_1631	0	0	0	0.00%	k__Bacteria
OTU_1632	0	2	0	0.00%	k__Bacteria;p__Firmicutes;c__Clostridia;o__Oscillospirales;f__[Eubacterium]_coprostanoligenes_group;g__;s__
OTU_1633	0	0	2	0.00%	k__Bacteria;p__unidentified_Bacteria;c__Acidimicrobiia;o__Microtrichales;f__Microtrichaceae;g__;s__
OTU_1634	0	1	2	0.00%	k__Bacteria;p__Bacteroidota;c__Bacteroidia;o__Bacteroidales;f__Bacteroidaceae;g__Bacteroides;s__
OTU_1635	3	3	3	0.00%	k__Bacteria;p__Proteobacteria;c__Alphaproteobacteria;o__Sphingomonadales;f__Sphingomonadaceae;g__Sphingorhabdus;s__
OTU_1636	0	0	0	0.00%	k__Bacteria;p__Proteobacteria;c__Gammaproteobacteria;o__Beggiatoales;f__Beggiatoaceae;g__Beggiatoa;s__
OTU_1637	2	0	0	0.00%	k__Bacteria;p__Firmicutes;c__Bacilli;o__RF39;f__;g__;s__
OTU_1638	3	0	0	0.00%	k__Bacteria;p__Bacteroidota;c__Bacteroidia;o__Sphingobacteriales;f__env.OPS_17;g__;s__
OTU_1639	0	0	0	0.00%	k__Bacteria;p__Bacteroidota;c__Bacteroidia;o__Bacteroidales;f__Rikenellaceae;g__Alistipes;s__Alistipes_putredinis
OTU_1640	0	0	0	0.00%	k__Bacteria;p__Firmicutes;c__Negativicutes;o__Veillonellales-Selenomonadales;f__Selenomonadaceae;g__Selenomonas
OTU_1641	0	0	3	0.00%	k__Bacteria;p__Bacteroidota;c__Bacteroidia;o__Bacteroidales;f__Marinifilaceae;g__Odoribacter;s__
OTU_1642	0	0	1	0.00%	k__Bacteria;p__Firmicutes;c__Clostridia;o__Clostridia_UCG-014;f__;g__;s__
OTU_1643	0	3	2	0.00%	k__Bacteria;p__Desulfobacterota;c__Desulfovibrionia;o__Desulfovibrionales;f__Desulfovibrionaceae;g__Desulfovibrio
OTU_1644	0	2	0	0.00%	k__Bacteria
OTU_1645	0	0	2	0.00%	k__Bacteria;p__Bacteroidota;c__Bacteroidia;o__Bacteroidales;f__Rikenellaceae;g__Rikenellaceae_RC9_gut_group;s__
OTU_1646	0	0	0	0.00%	k__Bacteria;p__unidentified_Bacteria;c__Babeliae;o__Babeliales
OTU_1647	0	3	0	0.00%	k__Bacteria;p__Firmicutes;c__Clostridia;o__Peptostreptococcales-Tissierellales;f__Family_XI;g__Tissierella;s__
OTU_1648	2	0	0	0.00%	k__Bacteria;p__Fibrobacterota;c__Fibrobacteria;o__Fibrobacterales;f__Fibrobacteraceae;g__Fibrobacter
OTU_1649	0	2	0	0.00%	k__Bacteria;p__Gemmatimonadota;c__Longimicrobia;o__Longimicrobiales;f__Longimicrobiaceae;g__;s__
OTU_1650	0	0	2	0.00%	k__Bacteria;p__Bacteroidota;c__Bacteroidia;o__Bacteroidales;f__Paludibacteraceae;g__Paludibacter;s__
OTU_1651	1	1	0	0.00%	k__Bacteria;p__Bacteroidota;c__Bacteroidia;o__Bacteroidales;f__Muribaculaceae;g__;s__
OTU_1652	2	0	0	0.00%	k__Bacteria;p__Bacteroidota;c__Bacteroidia;o__Cytophagales;f__Bernardetiaceae;g__Bernardetia
OTU_1653	0	0	2	0.00%	k__Bacteria
OTU_1654	4	2	0	0.00%	k__Bacteria;p__Proteobacteria;c__Gammaproteobacteria;o__Burkholderiales;f__Alcaligenaceae;g__Castellaniella;s__
OTU_1655	0	0	2	0.00%	k__Bacteria
OTU_1656	1	0	1	0.00%	k__Bacteria;p__Firmicutes;c__Clostridia;o__Oscillospirales;f__Hungateiclostridiaceae;g__Fastidiosipila;s__
OTU_1657	3	0	0	0.00%	k__Bacteria;p__Proteobacteria;c__Gammaproteobacteria;o__Xanthomonadales;f__Rhodanobacteraceae;g__Tahibacter;s__
OTU_1658	0	0	0	0.00%	k__Bacteria;p__Spirochaetota;c__Spirochaetia;o__Spirochaetales;f__Spirochaetaceae;g__Treponema
OTU_1659	0	3	0	0.00%	k__Bacteria;p__Cyanobacteria;c__Cyanobacteriia;o__Leptolyngbyales;f__Leptolyngbyaceae;g__CENA518;s__
OTU_1660	0	2	0	0.00%	k__Bacteria;p__unidentified_Bacteria;c__Saccharimonadia;o__Saccharimonadales;f__;g__;s__
OTU_1661	2	0	1	0.00%	k__Bacteria
OTU_1662	0	2	0	0.00%	k__Bacteria;p__Bacteroidota;c__Bacteroidia;o__Cytophagales;f__Spirosomaceae;g__Rudanella;s__

OTU_1663	0	0	0	0.00%	k__Bacteria;p__Bacteroidota;c__Bacteroidia;o__Bacteroidales
OTU_1664	0	0	0	0.00%	k__Bacteria;p__Proteobacteria;c__Gammaproteobacteria;o__Burkholderiales;f__Alcaligenaceae;g__GKS98_freshwater_group;s__Bordetella_sp
OTU_1665	3	2	1	0.00%	k__Bacteria;p__Firmicutes;c__Clostridia;o__Oscillospirales;f__Oscillospiraceae
OTU_1666	1	0	0	0.00%	k__Bacteria;p__Proteobacteria;c__Gammaproteobacteria;o__Burkholderiales;f__Nitrosomonadaceae;g__Ellin6067;s__
OTU_1667	1	4	1	0.00%	k__Bacteria;p__Firmicutes;c__Clostridia;o__Lachnospirales;f__Lachnospiraceae;g__Lachnospiraceae_UCG-003;s__
OTU_1668	2	0	0	0.00%	k__Bacteria;p__Proteobacteria;c__Gammaproteobacteria;o__Cardiobacteriales;f__Wohlfahrtiimonadaceae;g__Ignatzschineria;s__swine_effluent_bacterium_CHNDP41
OTU_1669	0	0	2	0.00%	k__Bacteria;p__Proteobacteria;c__Gammaproteobacteria;o__Enterobacterales;f__Aeromonadaceae;g__Oceanisphaera;s__
OTU_1670	0	0	0	0.00%	k__Bacteria;p__Bacteroidota;c__Bacteroidia;o__Flavobacteriales;f__Flavobacteriaceae;g__Flavobacterium;s__
OTU_1671	0	2	1	0.00%	k__Bacteria;p__Bdellovibrionota;c__Bdellovibrionia;o__Bacteriovoracales;f__Bacteriovoracaceae;g__Peredibacter;s__
OTU_1672	0	0	0	0.00%	k__Bacteria;p__unidentified_Bacteria;c__Gammaproteobacteria;o__Burkholderiales;f__Neisseriaceae;g__;s__
OTU_1673	2	0	0	0.00%	k__Bacteria;p__Firmicutes;c__Clostridia;o__Clostridiales;f__Caloramatoraceae;g__Fonticella;s__
OTU_1674	2	0	0	0.00%	k__Bacteria
OTU_1675	0	0	0	0.00%	k__Bacteria;p__Firmicutes;c__Negativicutes;o__Veillonellales-Selenomonadales;f__Sporomusaceae;g__Sporomusa
OTU_1676	0	1	1	0.00%	k__Bacteria;p__Nitrospirota;c__Nitrospira;o__Nitrospirales;f__Nitrospiraceae
OTU_1677	0	3	0	0.00%	k__Bacteria;p__Desulfobacterota;c__Desulfovibrionia;o__Desulfovibrionales;f__Desulfovibrionaceae;g__Desulfovibrio
OTU_1678	2	0	0	0.00%	k__Bacteria;p__Firmicutes;c__Clostridia;o__Peptostreptococcales-Tissierellales;f__Sedimentibacteraceae;g__Sedimentibacter;s__
OTU_1679	2	1	0	0.00%	k__Bacteria;p__Bacteroidota;c__Bacteroidia;o__Bacteroidales;f__Muribaculaceae;g__;s__
OTU_1680	3	0	0	0.00%	k__Bacteria;p__Cyanobacteria;c__Sericytochromatia
OTU_1681	0	0	2	0.00%	k__Bacteria;p__Proteobacteria;c__Gammaproteobacteria;o__Burkholderiales;f__Rhodocyclaceae;g__Candidatus_Accumulibacter;s__
OTU_1682	0	0	0	0.00%	k__Bacteria;p__Proteobacteria
OTU_1683	2	0	0	0.00%	k__Bacteria;p__Chloroflexi;c__KD4-96;o__;f__;g__;s__
OTU_1684	2	0	0	0.00%	k__Bacteria;p__Firmicutes;c__Clostridia;o__Lachnospirales;f__Lachnospiraceae
OTU_1685	0	2	1	0.00%	k__Bacteria;p__unidentified_Bacteria;c__Gammaproteobacteria;o__Halothiobacillales;f__Halothiobacillaceae;g__;s__
OTU_1686	2	0	0	0.00%	k__Bacteria;p__unidentified_Bacteria;c__Polyangia;o__Polyangiales;f__Sandaracinaceae;g__;s__
OTU_1687	6	0	0	0.00%	k__Bacteria;p__Bacteroidota;c__Bacteroidia;o__Bacteroidales;f__Prevotellaceae;g__Alloprevotella;s__
OTU_1688	7	5	2	0.01%	k__Bacteria;p__Proteobacteria;c__Gammaproteobacteria;o__Xanthomonadales;f__Xanthomonadaceae;g__Arenimonas;s__
OTU_1689	0	2	1	0.00%	k__Bacteria;p__Desulfobacterota;c__Desulfuromonadia;o__Bradymonadales;f__;g__;s__
OTU_1690	1	3	0	0.00%	k__Bacteria;p__Firmicutes;c__Bacilli;o__Exiguobacterales;f__Exiguobacteraceae;g__Exiguobacterium
OTU_1691	2	0	0	0.00%	k__Bacteria;p__Proteobacteria;c__Alphaproteobacteria;o__Acetobacterales;f__Acetobacteraceae;g__Rhodovastum;s__
OTU_1692	0	2	1	0.00%	k__Bacteria;p__Firmicutes;c__Clostridia;o__Peptococcales;f__Peptococcaceae;g__Peptococcus;s__
OTU_1693	0	0	5	0.00%	k__Bacteria;p__Bacteroidota;c__Bacteroidia;o__Bacteroidales;f__Muribaculaceae;g__;s__
OTU_1694	0	2	0	0.00%	k__Bacteria;p__Spirochaetota;c__MVP-15;o__;f__;g__;s__
OTU_1695	0	0	2	0.00%	k__Bacteria;p__Bacteroidota;c__Bacteroidia;o__Bacteroidales;f__Tannerellaceae;g__Parabacteroides;s__Parabacteroides_sp_CT06
OTU_1696	2	2	0	0.00%	k__Bacteria;p__unidentified_Bacteria;c__Gammaproteobacteria;o__Xanthomonadales;f__;g__;s__
OTU_1697	0	2	0	0.00%	k__Bacteria;p__Bacteroidota;c__Bacteroidia;o__Bacteroidales;f__Bacteroidetes_BD2-2
OTU_1698	0	0	2	0.00%	k__Bacteria;p__Firmicutes;c__Clostridia;o__Oscillospirales;f__[Eubacterium]_coprostanoligenes_group;g__;s__
OTU_1699	2	2	1	0.00%	k__Bacteria;p__Proteobacteria;c__Gammaproteobacteria;o__Pseudomonadales;f__Moraxellaceae;g__Acinetobacter
OTU_1700	2	0	0	0.00%	k__Bacteria;p__Bacteroidota;c__Bacteroidia;o__Chitinophagales;f__Saprospiraceae;g__Phaeodactylibacter;s__
OTU_1701	2	0	0	0.00%	k__Bacteria;p__Bacteroidota;c__Bacteroidia;o__Chitinophagales;f__Chitinophagaceae;g__Taibaiella;s__
OTU_1702	4	2	0	0.00%	k__Bacteria;p__Proteobacteria;c__Alphaproteobacteria;o__Rhizobiales;f__Rhizobiaceae;g__Aureimonas;s__
OTU_1703	0	0	0	0.00%	k__Bacteria;p__unidentified_Bacteria;c__Clostridia;o__Oscillospirales;f__Ruminococcaceae;g__;s__
OTU_1704	9	9	8	0.01%	k__Bacteria;p__Bacteroidota;c__Bacteroidia;o__Flavobacteriales;f__Flavobacteriaceae;g__Flavobacterium
OTU_1705	0	0	0	0.00%	k__Bacteria;p__unidentified_Bacteria;c__Alphaproteobacteria;o__Rhodospirillales;f__unidentified_Rhodospirillales;g__unidentified_Rhodospirillales;s__Azospirillum_sp_47_25
OTU_1706	2	2	1	0.00%	k__Bacteria;p__Proteobacteria;c__Gammaproteobacteria;o__Legionellales;f__Legionellaceae;g__Legionella;s__
OTU_1707	0	2	0	0.00%	k__Bacteria;p__unidentified_Bacteria;c__Saccharimonadia;o__Saccharimonadales;f__;g__;s__
OTU_1708	0	0	2	0.00%	k__Bacteria;p__Bacteroidota;c__Bacteroidia;o__Bacteroidales;f__Dysgonomonadaceae;g__Dysgonomonas;s__
OTU_1709	0	2	0	0.00%	k__Bacteria;p__Bacteroidota;c__Bacteroidia;o__Bacteroidales;f__Bacteroidaceae;g__Bacteroides;s__Bacteroides_acidifaciens
OTU_1710	1	1	2	0.00%	k__Bacteria;p__unidentified_Bacteria;c__Bacteroidia;o__Bacteroidales;f__Prolixibacteraceae;g__;s__
OTU_1711	1	0	0	0.00%	k__Bacteria;p__Cyanobacteria;c__Vampirivibrionia;o__Obscuribacterales;f__Obscuribacteraceae;g__;s__
OTU_1712	0	0	0	0.00%	k__Bacteria
OTU_1713	0	0	0	0.00%	k__Bacteria
OTU_1714	0	0	0	0.00%	k__Bacteria;p__Actinobacteria;c__unidentified_Actinobacteria;o__Corynebacteriales;f__Corynebacteriaceae;g__Corynebacterium
OTU_1715	2	0	0	0.00%	k__Bacteria;p__Firmicutes;c__Clostridia;o__Lachnospirales;f__Lachnospiraceae
OTU_1716	0	1	0	0.00%	k__Bacteria;p__Myxococcota;c__Polyangia;o__Polyangiales
OTU_1717	0	2	0	0.00%	k__Bacteria;p__Proteobacteria
OTU_1718	0	2	0	0.00%	k__Bacteria;p__Bacteroidota;c__Bacteroidia;o__Bacteroidales;f__Bacteroidaceae;g__Bacteroides

OTU_1719	2	3	1	0.00%	k__Bacteria;p__Firmicutes;c__Negativicutes;o__Veillonellales-Selenomonadales;f__Selenomonadaceae;g__Mitsuokella;s__
OTU_1720	0	2	0	0.00%	k__Bacteria;p__Gracilibacteria;c__unidentified_Gracilibacteria;o__JGI_0000069-P22;f__;g__;s__
OTU_1721	0	1	2	0.00%	k__Bacteria;p__Firmicutes;c__Clostridia
OTU_1722	3	0	1	0.00%	k__Bacteria;p__Firmicutes;c__Clostridia;o__Peptostreptococcales-Tissierellales;f__Anaerovoracaceae;g__Family_XIII_AD3011_group;s__
OTU_1723	0	2	0	0.00%	k__Bacteria;p__unidentified_Bacteria;c__unidentified_Bacteria;o__Gemmatimonadales;f__Gemmatimonadaceae;g__;s__
OTU_1724	0	0	0	0.00%	k__Bacteria;p__Bacteroidota;c__Bacteroidia;o__Cytophagales;f__Spirosomaceae;g__Leadbetterella;s__
OTU_1725	0	0	0	0.00%	k__Bacteria;p__Bacteroidota;c__Bacteroidia;o__Bacteroidales;f__Bacteroidaceae;g__Bacteroides;s__
OTU_1726	0	0	0	0.00%	k__Bacteria;p__Firmicutes;c__Negativicutes;o__Veillonellales-Selenomonadales;f__Veillonellaceae;g__Veillonella;s__Veillonella_ratti
OTU_1727	0	0	0	0.00%	k__Bacteria
OTU_1728	5	7	14	0.01%	k__Bacteria;p__Proteobacteria;c__Gammaproteobacteria;o__Thiotrichales;f__Thiotrichaceae;g__Thiothrix;s__
OTU_1729	0	0	1	0.00%	k__Bacteria;p__unidentified_Bacteria;c__Synergistia;o__Synergistales;f__Synergistaceae
OTU_1730	0	0	2	0.00%	k__Bacteria;p__Proteobacteria;c__Gammaproteobacteria;o__Pseudomonadales;f__Moraxellaceae;g__Acinetobacter
OTU_1731	0	0	2	0.00%	k__Bacteria;p__Kapabacteria;c__unidentified_Kapabacteria;o__Kapabacteriales;f__;g__;s__
OTU_1732	2	0	0	0.00%	k__Bacteria;p__Proteobacteria;c__Alphaproteobacteria;o__Azospirillales;f__unidentified_Azospirillales;g__Stella;s__
OTU_1733	1	0	0	0.00%	k__Bacteria;p__Cyanobacteria;c__Cyanobacteriia;o__Chloroplast;f__;g__;s__
OTU_1734	1	1	0	0.00%	k__Bacteria
OTU_1735	2	1	1	0.00%	k__Bacteria;p__unidentified_Bacteria;c__Bacilli;o__Lactobacillales;f__Carnobacteriaceae;g__;s__
OTU_1736	0	0	0	0.00%	k__Bacteria;p__Proteobacteria;c__Gammaproteobacteria;o__Burkholderiales;f__Neisseriaceae;g__Neisseria;s__Neisseria_sp_TID-16
OTU_1737	1	0	0	0.00%	k__Bacteria;p__Synergistota;c__Synergistia;o__Synergistales;f__Synergistaceae;g__Syner-01;s__
OTU_1738	0	6	5	0.01%	k__Bacteria;p__Firmicutes;c__Clostridia;o__Clostridia_UCG-014;f__;g__;s__
OTU_1739	0	0	1	0.00%	k__Bacteria;p__unidentified_Bacteria;c__Bacteroidia;o__Chitinophagales;f__unidentified_Chitinophagales;g__unidentified_Chitinophagales;s__bacterium_episymbiont_of_Kiwa_sp
OTU_1740	0	0	0	0.00%	k__Bacteria;p__Firmicutes;c__Bacilli;o__Lactobacillales;f__Lactobacillaceae;g__Lactobacillus
OTU_1741	0	0	2	0.00%	k__Bacteria;p__unidentified_Bacteria;c__Desulfovibrionia;o__Desulfovibrionales;f__Desulfovibrionaceae;g__;s__
OTU_1742	0	3	0	0.00%	k__Bacteria;p__Actinobacteria;c__unidentified_Actinobacteria;o__Micrococcales;f__Bogoriellaceae;g__Georgenia
OTU_1743	0	0	3	0.00%	k__Bacteria
OTU_1744	0	2	1	0.00%	k__Bacteria;p__unidentified_Bacteria;c__Gammaproteobacteria;o__Diplorickettsiales;f__Diplorickettsiaceae;g__;s__
OTU_1745	0	2	0	0.00%	k__Bacteria;p__Bacteroidota;c__Bacteroidia;o__Bacteroidales;f__Paludibacteraceae
OTU_1746	3	0	0	0.00%	k__Bacteria;p__Firmicutes;c__Clostridia;o__Oscillospirales;f__UCG-010;g__;s__
OTU_1747	0	3	0	0.00%	k__Bacteria;p__Proteobacteria;c__Gammaproteobacteria;o__Burkholderiales;f__Nitrosomonadaceae;g__Nitrosospira;s__
OTU_1748	1	1	0	0.00%	k__Bacteria;p__Proteobacteria;c__Gammaproteobacteria;o__Burkholderiales;f__Oxalobacteraceae;g__Oxalobacter;s__Oxalobacter_formigenes
OTU_1749	3	0	0	0.00%	k__Bacteria;p__Proteobacteria;c__Alphaproteobacteria;o__Rhodobacterales;f__Rhodobacteraceae;g__Palleronia-Pseudomaribius;s__Palleronia_marisminoris
OTU_1750	2	0	0	0.00%	k__Bacteria;p__Firmicutes;c__Clostridia;o__Peptostreptococcales-Tissierellales;f__Anaerovoracaceae;g__Family_XIII_UCG-001;s__
OTU_1751	0	0	1	0.00%	k__Bacteria;p__Firmicutes;c__Clostridia;o__Oscillospirales;f__UCG-010;g__;s__
OTU_1752	0	2	2	0.00%	k__Bacteria;p__Desulfobacterota;c__Desulfovibrionia;o__Desulfovibrionales;f__Desulfovibrionaceae;g__Desulfovibrio
OTU_1753	0	2	0	0.00%	k__Bacteria;p__unidentified_Bacteria;c__Bacteroidia;o__Bacteroidales;f__Barnesiellaceae;g__;s__
OTU_1754	0	0	0	0.00%	k__Bacteria;p__Proteobacteria;c__Alphaproteobacteria;o__Rickettsiales;f__unidentified_Rickettsiales;g__Candidatus_Jidaibacter;s__
OTU_1755	0	0	0	0.00%	k__Bacteria;p__Kapabacteria;c__unidentified_Kapabacteria;o__Kapabacteriales;f__;g__;s__
OTU_1756	0	0	2	0.00%	k__Bacteria;p__Proteobacteria;c__Alphaproteobacteria;o__Acetobacterales;f__Acetobacteraceae;g__Roseomonas
OTU_1757	0	0	2	0.00%	k__Bacteria;p__Firmicutes;c__Negativicutes;o__Veillonellales-Selenomonadales;f__Veillonellaceae;g__Dialister
OTU_1758	0	0	0	0.00%	k__Bacteria;p__Bacteroidota;c__Bacteroidia;o__Bacteroidales;f__Muribaculaceae;g__;s__
OTU_1759	1	2	0	0.00%	k__Bacteria
OTU_1760	0	0	1	0.00%	k__Bacteria;p__Synergistota;c__Synergistia;o__Synergistales;f__Synergistaceae;g__Pyramidobacter
OTU_1761	0	1	2	0.00%	k__Bacteria;p__unidentified_Bacteria;c__Spirochaetia;o__Spirochaetales;f__Spirochaetaceae
OTU_1762	0	3	0	0.00%	k__Bacteria;p__Proteobacteria;c__Gammaproteobacteria;o__Steroidobacterales;f__Woeseiaceae;g__Woeseia;s__
OTU_1763	0	2	0	0.00%	k__Bacteria;p__Proteobacteria;c__Alphaproteobacteria;o__Dongiales;f__Dongiaceae;g__Dongia;s__
OTU_1764	2	0	0	0.00%	k__Bacteria;p__Bacteroidota;c__Bacteroidia;o__Sphingobacteriales;f__Sphingobacteriaceae;g__Arcticibacter;s__
OTU_1765	0	0	0	0.00%	k__Bacteria;p__Firmicutes;c__Clostridia;o__Clostridia_vadinBB60_group;f__;g__;s__
OTU_1766	1	4	2	0.00%	k__Bacteria;p__Firmicutes;c__Clostridia;o__Lachnospirales;f__Lachnospiraceae;g__GCA-900066575;s__
OTU_1767	0	2	0	0.00%	k__Bacteria;p__Proteobacteria;c__Gammaproteobacteria;o__Burkholderiales;f__Chromobacteriaceae;g__Paludibacterium;s__
OTU_1768	0	3	0	0.00%	k__Bacteria;p__Acidobacteriota;c__Vicinamibacteria;o__Vicinamibacterales;f__Vicinamibacteraceae;g__;s__
OTU_1769	0	0	2	0.00%	k__Bacteria;p__Proteobacteria;c__Alphaproteobacteria;o__Azospirillales;f__unidentified_Azospirillales;g__Stella;s__
OTU_1770	2	0	0	0.00%	k__Bacteria;p__unidentified_Bacteria;c__Vicinamibacteria;o__Vicinamibacterales;f__;g__;s__
OTU_1771	0	0	1	0.00%	k__Bacteria;p__unidentified_Bacteria;c__Desulfovibrionia;o__Desulfovibrionales;f__Desulfovibrionaceae;g__;s__
OTU_1772	0	0	1	0.00%	k__Bacteria;p__unidentified_Bacteria;c__Bacteroidia;o__Bacteroidales;f__Dysgonomonadaceae;g__;s__
OTU_1773	0	0	0	0.00%	k__Bacteria;p__Spirochaetota;c__Leptospirae;o__Leptospirales;f__Leptospiraceae;g__;s__
OTU_1774	0	0	0	0.00%	k__Bacteria;p__Proteobacteria;c__Gammaproteobacteria;o__Competibacterales;f__Competibacteraceae;g__Candidatus_Competibacter;s__

OTU_1775	0	0	0	0.00%	k__Bacteria;p__unidentified_Bacteria;c__Gammaproteobacteria;o__Diplorickettsiales;f__Diplorickettsiaceae;g__;s__
OTU_1776	0	2	0	0.00%	Unknown
OTU_1777	0	0	1	0.00%	k__Bacteria;p__unidentified_Bacteria;c__Clostridia;o__Oscillospirales;f__Ruminococcaceae;g__;s__
OTU_1778	0	0	0	0.00%	k__Bacteria;p__Proteobacteria;c__Gammaproteobacteria;o__Beggiatoales;f__Beggiatoaceae;g__Beggiatoa;s__Beggiatoa_sp_1401-13
OTU_1779	0	0	0	0.00%	k__Bacteria;p__Actinobacteriota;c__Thermoleophilia;o__Solirubrobacterales
OTU_1780	0	4	0	0.00%	k__Bacteria;p__Gemmatimonadetes;c__unidentified_Gemmatimonadetes;o__Gemmatimonadales;f__Gemmatimonadaceae;g__Gemmatimonas;s__
OTU_1781	0	0	0	0.00%	k__Bacteria;p__Bacteroidota;c__Ignavibacteria;o__Ignavibacteriales;f__Ignavibacteriaceae;g__Ignavibacterium
OTU_1782	2	2	2	0.00%	k__Bacteria;p__Proteobacteria;c__Gammaproteobacteria;o__Pseudomonadales;f__Pseudomonadaceae;g__Pseudomonas;s__Pseudomonas_sp_76
OTU_1783	0	1	0	0.00%	k__Bacteria;p__Proteobacteria;c__Alphaproteobacteria;o__Acetobacterales;f__Acetobacteraceae;g__Roseomonas;s__Roseomonas_lacus
OTU_1784	0	0	0	0.00%	k__Bacteria;p__Myxococcota;c__Polyangia;o__mle1-27;f__;g__;s__
OTU_1785	2	0	0	0.00%	k__Bacteria;p__Firmicutes;c__Clostridia;o__Peptostreptococcales-Tissierellales;f__unidentified_Peptostreptococcales-Tissierellales;g__Brassicibacter;s__
OTU_1786	1	2	0	0.00%	k__Bacteria;p__Bacteroidota;c__Bacteroidia;o__Sphingobacteriales
OTU_1787	2	2	11	0.01%	k__Bacteria;p__Proteobacteria;c__Gammaproteobacteria;o__Pseudomonadales;f__Moraxellaceae;g__Acinetobacter
OTU_1788	20	10	14	0.02%	k__Bacteria;p__Proteobacteria;c__Gammaproteobacteria;o__Burkholderiales;f__Comamonadaceae;g__Sphaerotilus;s__
OTU_1789	2	0	0	0.00%	k__Bacteria
OTU_1790	0	0	2	0.00%	k__Bacteria;p__Firmicutes;c__Desulfotomaculia;o__Desulfotomaculales;f__Desulfotomaculaceae;g__Desulfohalotomaculum
OTU_1791	0	0	0	0.00%	k__Bacteria;p__Gracilibacteria;c__unidentified_Gracilibacteria;o__Absconditabacteriales_(SR1);f__;g__;s__
OTU_1792	0	0	0	0.00%	k__Bacteria
OTU_1793	0	0	0	0.00%	k__Bacteria;p__Firmicutes;c__Bacilli;o__RF39;f__;g__;s__
OTU_1794	0	0	2	0.00%	k__Bacteria
OTU_1795	2	0	0	0.00%	k__Bacteria;p__Firmicutes;c__Clostridia;o__Oscillospirales
OTU_1796	2	0	0	0.00%	k__Bacteria
OTU_1797	0	0	0	0.00%	k__Bacteria;p__Firmicutes;c__Clostridia;o__Lachnospirales;f__Lachnospiraceae;g__Catonella;s__
OTU_1798	0	1	4	0.00%	k__Bacteria;p__Firmicutes;c__Bacilli;o__Lactobacillales;f__Lactobacillaceae;g__Pediococcus;s__Pediococcus_acidilactici
OTU_1799	0	3	0	0.00%	k__Bacteria;p__Proteobacteria;c__Gammaproteobacteria;o__Xanthomonadales;f__Xanthomonadaceae;g__Lysobacter
OTU_1800	0	2	1	0.00%	k__Bacteria;p__Firmicutes;c__Clostridia;o__Oscillospirales;f__[Eubacterium]_coprostanoligenes_group;g__;s__
OTU_1801	1	0	2	0.00%	k__Bacteria;p__Proteobacteria;c__Alphaproteobacteria;o__Rhodobacterales;f__Rhodobacteraceae;g__Roseobacter_clade_CHAB-I-5_lineage;s__
OTU_1802	0	3	0	0.00%	k__Bacteria;p__Firmicutes;c__Clostridia;o__Oscillospirales;f__Ruminococcaceae
OTU_1803	0	0	2	0.00%	k__Bacteria;p__Firmicutes;c__Clostridia;o__Oscillospirales;f__Hungateiclostridiaceae;g__Fastidiosipila;s__
OTU_1804	2	0	0	0.00%	k__Bacteria;p__Proteobacteria;c__Gammaproteobacteria
OTU_1805	0	0	0	0.00%	k__Bacteria;p__Bacteroidota;c__Bacteroidia;o__Flavobacteriales;f__Flavobacteriaceae;g__Flavobacterium
OTU_1806	0	0	2	0.00%	k__Bacteria;p__Kapabacteria;c__unidentified_Kapabacteria;o__Kapabacteriales;f__;g__;s__
OTU_1807	1	1	1	0.00%	k__Bacteria;p__Firmicutes;c__Bacilli;o__Lactobacillales;f__Aerococcaceae;g__Aerococcus;s__Aerococcus_urinaeequi
OTU_1808	0	0	2	0.00%	k__Bacteria;p__unidentified_Bacteria;c__Bacteroidia;o__Chitinophagales;f__Saprospiraceae;g__;s__
OTU_1809	0	3	0	0.00%	k__Bacteria;p__Actinobacteria;c__unidentified_Actinobacteria;o__Corynebacteriales;f__Nocardiaceae;g__Rhodococcus;s__Rhodococcus_corynebacterioides
OTU_1810	3	0	0	0.00%	k__Bacteria;p__Verrucomicrobiota;c__Verrucomicrobiae;o__Verrucomicrobiales;f__Verrucomicrobiaceae;g__Verrucomicrobium;s__
OTU_1811	0	2	0	0.00%	k__Bacteria;p__Firmicutes;c__Clostridia;o__Peptostreptococcales-Tissierellales;f__Family_XI;g__Tepidimicrobium;s__Tepidimicrobium_ferriphilum
OTU_1812	1	1	0	0.00%	k__Bacteria;p__Proteobacteria;c__Gammaproteobacteria;o__Coxiellales;f__Coxiellaceae;g__Coxiella;s__Gammaproteobacteria_bacterium_RBG_16_37_9
OTU_1813	0	0	2	0.00%	k__Bacteria;p__unidentified_Bacteria;c__Bacteroidia;o__Bacteroidales;f__Paludibacteraceae;g__;s__
OTU_1814	0	0	2	0.00%	k__Bacteria;p__Bacteroidota;c__Bacteroidia;o__Cytophagales;f__Hymenobacteraceae;g__Adhaeribacter;s__
OTU_1815	0	2	0	0.00%	k__Bacteria;p__Sumerlaeota;c__Sumerlaeia;o__Sumerlaeales;f__Sumerlaeaceae;g__Sumerlaea;s__
OTU_1816	0	1	2	0.00%	k__Bacteria;p__Firmicutes;c__Clostridia;o__Peptostreptococcales-Tissierellales;f__Anaerovoracaceae;g__Family_XIII_UCG-001;s__
OTU_1817	0	1	0	0.00%	k__Bacteria
OTU_1818	0	0	2	0.00%	k__Bacteria;p__Actinobacteria;c__unidentified_Actinobacteria;o__Propionibacteriales;f__Nocardioidaceae;g__Nocardioides
OTU_1819	0	0	2	0.00%	k__Bacteria;p__Proteobacteria;c__Alphaproteobacteria;o__Rhizobiales;f__unidentified_Rhizobiales;g__Alsobacter;s__
OTU_1820	0	0	3	0.00%	k__Bacteria;p__Chloroflexi;c__Chloroflexia;o__Thermomicrobiales;f__JG30-KF-CM45;g__;s__
OTU_1821	0	2	0	0.00%	k__Bacteria;p__unidentified_Bacteria;c__Anaerolineae;o__Ardenticatenales;f__Ardenticatenaceae;g__;s__
OTU_1822	0	0	2	0.00%	k__Bacteria;p__Proteobacteria;c__Alphaproteobacteria;o__Rhodobacterales;f__Rhodobacteraceae;g__Rubellimicrobium;s__
OTU_1823	4	7	4	0.01%	k__Bacteria;p__Firmicutes;c__Clostridia;o__Clostridia_UCG-014;f__;g__;s__
OTU_1824	0	0	2	0.00%	k__Bacteria;p__Bacteroidota;c__Bacteroidia;o__Bacteroidales
OTU_1825	0	0	0	0.00%	k__Bacteria;p__Proteobacteria;c__Gammaproteobacteria;o__Legionellales;f__Legionellaceae;g__Legionella;s__
OTU_1826	0	0	1	0.00%	k__Bacteria;p__Bdellovibrionota;c__Oligoflexia;o__053A03-B-DI-P58;f__;g__;s__
OTU_1827	0	1	0	0.00%	k__Bacteria;p__Proteobacteria;c__Gammaproteobacteria;o__Methylococcales;f__Methylococcaceae
OTU_1828	0	0	0	0.00%	k__Bacteria;p__Firmicutes;c__Bacilli;o__Erysipelotrichales;f__Erysipelotrichaceae;g__Holdemania;s__
OTU_1829	0	0	0	0.00%	k__Bacteria;p__Bacteroidota;c__Bacteroidia;o__Bacteroidales;f__Rikenellaceae;g__Alistipes;s__
OTU_1830	7	7	3	0.01%	k__Bacteria;p__Proteobacteria;c__Gammaproteobacteria;o__Burkholderiales;f__Rhodocyclaceae;g__Zoogloea;s__

OTU_1831	1	0	0	0.00%	k__Bacteria;p__Proteobacteria;c__Gammaproteobacteria;o__Pseudomonadales;f__Hahellaceae;g__Hahella;s__
OTU_1832	2	3	0	0.00%	k__Bacteria;p__Firmicutes;c__Clostridia;o__Lachnospirales;f__Lachnospiraceae
OTU_1833	1	0	1	0.00%	k__Bacteria;p__unidentified_Bacteria;c__Bacteroidia;o__Chitinophagales;f__;g__;s__
OTU_1834	7	1	2	0.01%	k__Bacteria;p__Bacteroidota;c__Bacteroidia;o__Cytophagales;f__Hymenobacteraceae;g__Pontibacter;s__Pontibacter_populi
OTU_1835	0	2	0	0.00%	k__Bacteria;p__unidentified_Bacteria;c__Rhodothermia;o__Rhodothermales;f__Rhodothermaceae;g__;s__
OTU_1836	0	2	0	0.00%	k__Bacteria;p__Proteobacteria;c__Gammaproteobacteria;o__Burkholderiales;f__Chromobacteriaceae;g__Aquitalea;s__Aquitalea_magnusonii
OTU_1837	2	2	0	0.00%	k__Bacteria;p__Actinobacteria;c__unidentified_Actinobacteria;o__Corynebacteriales;f__Corynebacteriaceae;g__Lawsonella
OTU_1838	0	0	2	0.00%	k__Bacteria;p__Verrucomicrobiota;c__Verrucomicrobiae;o__Pedosphaerales;f__Pedosphaeraceae;g__DEV114
OTU_1839	0	2	0	0.00%	k__Bacteria;p__Firmicutes;c__Clostridia;o__Clostridiales;f__Clostridiaceae;g__Clostridium_sensu_stricto_12;s__
OTU_1840	13	11	37	0.03%	k__Bacteria;p__Bacteroidota;c__Bacteroidia;o__Bacteroidales;f__Tannerellaceae;g__Parabacteroides
OTU_1841	0	3	2	0.00%	k__Bacteria;p__Proteobacteria;c__Gammaproteobacteria;o__Enterobacterales;f__Aeromonadaceae;g__Aeromonas
OTU_1842	0	0	2	0.00%	k__Bacteria;p__unidentified_Bacteria;c__Acidimicrobiia;o__Microtrichales;f__;g__;s__
OTU_1843	0	2	0	0.00%	k__Bacteria;p__Acidobacteriota;c__Holophagae;o__Holophagales;f__Holophagaceae;g__Holophaga;s__
OTU_1844	2	0	0	0.00%	k__Bacteria;p__Firmicutes;c__Bacilli;o__Acholeplasmatales;f__Acholeplasmataceae;g__Acholeplasma
OTU_1845	36	51	35	0.06%	k__Bacteria;p__Firmicutes;c__Clostridia;o__Lachnospirales;f__Lachnospiraceae;g__[Ruminococcus]_gauvreauii_group;s__
OTU_1846	1	0	2	0.00%	k__Bacteria;p__Proteobacteria;c__Gammaproteobacteria;o__Xanthomonadales;f__Xanthomonadaceae;g__Thermomonas
OTU_1847	0	0	1	0.00%	k__Bacteria;p__Proteobacteria;c__Alphaproteobacteria;o__Rhizobiales;f__Beijerinckiaceae;g__Qingshengfania;s__Pseudochelatococcus_lubricantis
OTU_1848	0	1	0	0.00%	k__Bacteria;p__Proteobacteria;c__Gammaproteobacteria;o__Burkholderiales;f__Burkholderiaceae;g__Lautropia;s__
OTU_1849	1	0	0	0.00%	k__Bacteria;p__unidentified_Bacteria;c__Alphaproteobacteria;o__Micavibrionales;f__;g__;s__
OTU_1850	0	0	0	0.00%	k__Bacteria;p__Bdellovibrionota;c__Bdellovibrionia;o__Bdellovibrionales;f__Bdellovibrionaceae;g__Bdellovibrio;s__Bdellovibrio_bacteriovorus
OTU_1851	2	3	1	0.00%	k__Bacteria;p__Proteobacteria;c__Gammaproteobacteria;o__Xanthomonadales;f__Rhodanobacteraceae;g__Aquimonas;s__
OTU_1852	9	6	11	0.01%	k__Bacteria;p__Campylobacterota;c__Campylobacteria;o__Campylobacterales;f__Sulfurospirillaceae;g__Sulfurospirillum
OTU_1853	0	0	0	0.00%	k__Bacteria;p__Bacteroidota;c__Bacteroidia;o__Bacteroidales;f__Marinifilaceae;g__Butyricimonas;s__
OTU_1854	0	1	2	0.00%	k__Bacteria;p__Kapabacteria;c__unidentified_Kapabacteria;o__Kapabacteriales;f__;g__;s__
OTU_1855	0	0	0	0.00%	k__Bacteria;p__Bacteroidota;c__Bacteroidia;o__Chitinophagales;f__Chitinophagaceae;g__Niabella;s__
OTU_1856	0	0	1	0.00%	k__Bacteria;p__Actinobacteriota;c__Coriobacteriia;o__Coriobacteriales;f__Eggerthellaceae;g__Enterorhabdus;s__
OTU_1857	0	1	0	0.00%	k__Bacteria;p__Proteobacteria;c__Alphaproteobacteria;o__Rickettsiales;f__Mitochondria
OTU_1858	0	0	3	0.00%	k__Bacteria;p__Cyanobacteria;c__Sericytochromatia;o__;f__;g__;s__
OTU_1859	0	0	0	0.00%	k__Bacteria;p__Bacteroidota;c__Bacteroidia;o__Sphingobacteriales;f__env.OPS_17;g__;s__
OTU_1860	0	0	0	0.00%	k__Bacteria;p__Proteobacteria;c__Gammaproteobacteria
OTU_1861	0	0	0	0.00%	k__Bacteria;p__Bacteroidota;c__Bacteroidia
OTU_1862	0	0	0	0.00%	k__Bacteria;p__Firmicutes
OTU_1863	0	1	1	0.00%	k__Bacteria;p__Proteobacteria;c__Gammaproteobacteria;o__Pseudomonadales;f__Halomonadaceae;g__Kushneria
OTU_1864	0	0	0	0.00%	k__Bacteria;p__Proteobacteria;c__Gammaproteobacteria
OTU_1865	0	2	1	0.00%	k__Bacteria;p__Bacteroidota;c__Bacteroidia;o__Bacteroidales;f__Prevotellaceae
OTU_1866	1	0	2	0.00%	k__Bacteria;p__Desulfobacterota
OTU_1867	3	0	0	0.00%	k__Bacteria;p__Chloroflexi;c__Anaerolineae;o__SBR1031;f__;g__;s__
OTU_1868	0	2	0	0.00%	k__Bacteria;p__NB1-j;c__;o__;f__;g__;s__
OTU_1869	0	2	0	0.00%	k__Bacteria;p__Firmicutes;c__Bacilli;o__Erysipelotrichales;f__Erysipelatoclostridiaceae;g__Asteroleplasma;s__
OTU_1870	0	3	1	0.00%	k__Bacteria;p__Acidobacteriota;c__Aminicenantia;o__Aminicenantales;f__;g__;s__
OTU_1871	0	2	0	0.00%	k__Bacteria;p__Firmicutes;c__Clostridia;o__Lachnospirales;f__Lachnospiraceae
OTU_1872	26	17	11	0.03%	k__Bacteria;p__Firmicutes;c__Clostridia;o__Lachnospirales;f__Lachnospiraceae
OTU_1873	0	0	2	0.00%	k__Bacteria;p__Proteobacteria;c__Gammaproteobacteria;o__Pseudomonadales;f__Spongiibacteraceae;g__;s__
OTU_1874	0	2	0	0.00%	k__Bacteria;p__Firmicutes;c__Clostridia;o__Lachnospirales;f__Lachnospiraceae;g__A2;s__
OTU_1875	3	0	3	0.00%	k__Bacteria;p__Actinobacteria;c__unidentified_Actinobacteria;o__Micrococcales;f__Dermabacteraceae;g__Brachybacterium;s__Brachybacterium_paraconglomeratum
OTU_1876	6	2	10	0.01%	k__Bacteria;p__Proteobacteria;c__Gammaproteobacteria;o__Burkholderiales;f__Comamonadaceae
OTU_1877	2	0	0	0.00%	k__Bacteria;p__Planctomycetota;c__Phycisphaerae;o__Tepidisphaerales;f__WD2101_soil_group;g__;s__
OTU_1878	2	0	0	0.00%	k__Bacteria;p__Firmicutes;c__Clostridia;o__Lachnospirales;f__Lachnospiraceae
OTU_1879	0	3	0	0.00%	k__Bacteria;p__Cyanobacteria;c__Sericytochromatia
OTU_1880	2	0	0	0.00%	k__Bacteria;p__Firmicutes;c__Bacilli;o__Aneurinibacillales;f__Aneurinibacillaceae;g__Aneurinibacillus;s__
OTU_1881	0	2	0	0.00%	k__Bacteria;p__Proteobacteria;c__Gammaproteobacteria;o__Burkholderiales;f__Alcaligenaceae;g__GKS98_freshwater_group;s__
OTU_1882	0	1	1	0.00%	k__Bacteria;p__Proteobacteria;c__Alphaproteobacteria;o__Rhizobiales;f__A0839;g__;s__
OTU_1883	0	2	0	0.00%	k__Bacteria
OTU_1884	0	0	0	0.00%	k__Bacteria;p__Proteobacteria;c__Gammaproteobacteria;o__Pseudomonadales;f__Pseudomonadaceae
OTU_1885	0	0	0	0.00%	k__Bacteria
OTU_1886	0	0	0	0.00%	k__Bacteria

OTU_1887	2	0	0	0.00%	k__Bacteria;p__unidentified_Bacteria;c__Alphaproteobacteria;o__Rhodospirillales;f__;g__;s__
OTU_1888	2	0	0	0.00%	k__Bacteria;p__unidentified_Bacteria;c__Chloroflexia;o__Chloroflexales;f__Roseiflexaceae;g__;s__
OTU_1889	0	0	0	0.00%	k__Bacteria;p__Firmicutes
OTU_1890	0	0	0	0.00%	k__Bacteria;p__Firmicutes;c__Clostridia;o__Lachnospirales;f__Lachnospiraceae;g__Blautia;s__Lachnospiraceae_bacterium_615
OTU_1891	0	0	0	0.00%	k__Bacteria;p__Bacteroidota;c__Bacteroidia;o__Flavobacteriales;f__Flavobacteriaceae;g__Flavobacterium
OTU_1892	0	0	2	0.00%	k__Bacteria;p__Gracilibacteria;c__unidentified_Gracilibacteria;o__JGI_0000069-P22;f__;g__;s__
OTU_1893	2	1	0	0.00%	k__Bacteria;p__Acidobacteriota;c__Blastocatellia;o__Blastocatellales;f__Blastocatellaceae;g__Aridibacter;s__
OTU_1894	0	1	3	0.00%	k__Bacteria;p__Firmicutes;c__Negativicutes;o__Veillonellales-Selenomonadales;f__Veillonellaceae;g__Megasphaera;s__Megasphaera_hexanoica
OTU_1895	0	2	0	0.00%	k__Bacteria
OTU_1896	0	0	0	0.00%	k__Bacteria
OTU_1897	1	1	0	0.00%	k__Bacteria
OTU_1898	0	0	2	0.00%	k__Bacteria;p__Proteobacteria;c__Gammaproteobacteria;o__Enterobacterales;f__Succinivibrionaceae;g__Ruminobacter;s__Ruminobacter_amylophilus
OTU_1899	5	0	0	0.00%	k__Bacteria;p__Firmicutes;c__Clostridia;o__Monoglobales;f__Monoglobaceae;g__Monoglobus;s__
OTU_1900	1	0	2	0.00%	k__Bacteria;p__Proteobacteria;c__Alphaproteobacteria;o__Caulobacterales;f__Caulobacteraceae;g__PMMR1;s__
OTU_1901	0	1	1	0.00%	k__Bacteria
OTU_1902	0	0	2	0.00%	k__Bacteria;p__Proteobacteria;c__Gammaproteobacteria;o__Burkholderiales;f__Nitrosomonadaceae;g__MND1;s__
OTU_1903	0	1	0	0.00%	k__Bacteria;p__unidentified_Bacteria;c__Gammaproteobacteria;o__Diplorickettsiales;f__Diplorickettsiaceae;g__;s__
OTU_1904	0	2	0	0.00%	k__Bacteria;p__Actinobacteria;c__unidentified_Actinobacteria;o__Kineosporiales;f__Kineosporiaceae;g__Kineococcus
OTU_1905	0	0	2	0.00%	k__Bacteria;p__Firmicutes;c__Clostridia;o__Oscillospirales;f__Butyricicoccaceae;g__UCG-009
OTU_1906	0	0	0	0.00%	k__Bacteria;p__Actinobacteria;c__unidentified_Actinobacteria;o__Actinomycetales;f__Actinomycetaceae;g__Actinomyces
OTU_1907	0	1	0	0.00%	k__Bacteria;p__Proteobacteria;c__Gammaproteobacteria;o__Burkholderiales;f__Nitrosomonadaceae;g__Ellin6067;s__
OTU_1908	0	1	0	0.00%	k__Bacteria;p__Proteobacteria;c__Gammaproteobacteria;o__Burkholderiales;f__Rhodocyclaceae
OTU_1909	0	0	0	0.00%	k__Bacteria;p__Firmicutes;c__Clostridia;o__Oscillospirales;f__Oscillospiraceae;g__NK4A214_group;s__
OTU_1910	0	0	0	0.00%	k__Bacteria;p__unidentified_Bacteria;c__Bacteroidia;o__Bacteroidales;f__Rikenellaceae;g__;s__
OTU_1911	0	0	0	0.00%	k__Bacteria;p__Firmicutes;c__Clostridia;o__Clostridia_vadinBB60_group;f__;g__;s__
OTU_1912	0	0	1	0.00%	k__Bacteria;p__Firmicutes;c__Clostridia;o__Peptococcales;f__Peptococcaceae;g__Dehalobacterium
OTU_1913	1	0	0	0.00%	k__Bacteria;p__Bacteroidota;c__Bacteroidia;o__Chitinophagales;f__Saprospiraceae;g__Lewinella;s__
OTU_1914	2	0	0	0.00%	k__Bacteria
OTU_1915	0	0	0	0.00%	k__Bacteria

Supplementary file 2. The 16S rRNA genes of the successfully cultured organic-PSM cells derived via scRACS-Culture.

>F4_ *Comamonas* spp._16S ribosomal RNA gene

TGGGGGGGCTGGCCTTACCATGCAAGTCGAACGGTAACAGGTCTTCGGATGCTGACGAGTGGCG
AACGGGTGAGTAATACATCGGAACGTGCCTAGTAGTGGGGGATAACTACTCGAAAGAGTGGCTA
ATACCGCATGAGATCTACGGATGAAAGCAGGGGATCGCAAGACCTTGTGCTACTAGAGCGGCCG
ATGGCAGATTAGGTAGTTGGTGGGATAAAAGCTTACCAAGCCGACGATCTGTAGCTGGTCTGAG
AGGACGATCAGCCACACTGGGACTGAGACACGGCCCAGACTCCTACGGGAGGCAGCAGTGGGG
AATTTTGGACAATGGGCGCAAGCCTGATCCAGCAATGCCGCGTGCAGGATGAAGGCCTTCGGGT
TGTAAACTGCTTTTGTACGGAACGAAAAGTCCCGGGTTAATACCCTGGGGTCATGACGGTACCGT
AAGAATAAGCACCGGCTAACTACGTGCCAGCAGCCGCGGTAATACGTAGGGTGCGAGCGTTAAT
CGGAATTACTGGGCGTAAAGCGTGCGCAGGCGGTTTTGTAAGACAGAGGTGAAATCCCCGGGCT
CAACCTGGGAACTGCCTTTGTGACTGCAAGGCTAGAGTACGGCAGAGGGGGATGGAATTCCGCG
TGTAGCAGTGAAATGCGTAGATATGCGGAGGAACACCGATGGCGAAGGCAATCCCCTGGGCCTG
TACTGACGCTCATGCACGAAAGCGTGGGGAGCAAACAGGATTAGATACCCTGGTAGTCCACGCC
CTAAACGATGTCAACTGGTTGTTGGGTCTTAACTGACTCAGTAACGAAGCTAACGCGTGAAGTTG
ACCGCCTGGGGAGTACGGCCGCAAGGTTGAAACTCAAAGGAATTGACGGGGACCCGCACAAGC
GGTGGATGATGTGGTTTAATTCGATGCAACGCGAAAAACCTTACCCACCTTTGACATGTACGGA
ATCCTTTAGAGATAGAGGAGTGCTCGAAAGAGAGCCGTAACACAGGTGCTGCATGGCTGTCGTC
AGCTCGTGTCGTGAGATGTTGGGTTAAGTCCCGCAACGAGCGCAACCCTTGCCATTAGTTGCTAC
GAAAGGGCACTCTAATGGGACTGCCGGTGACAAACCGGAGGAAGGTGGGGATGACGTCAAGTC
CTCATGGCCCTTATAGGTGGGGCTACACACGTCATACAATGGCTGGTACAAAGGGTTGCCAACC
CGCGAGGGGGAGCTAATCCCATAAAGCCAGTCGTAGTCCGGATCGCAGTCTGCAACTCGACTGC
GTGAAGTCGGAATCGCTAGTAATCGTGGATCAGAATGTCACGGTGAATACGTTCCCGGGTCTTGT
ACACACCGCCCGTCACACCATGGGAGCGGGTCTCGCCAGAAGTAGGTAGCCTAACCGCAAGGAG
GCGCTACCACGTCCGGT

>S7_ *Acinetobacter* spp._16S ribosomal RNA gene

GGGGGGGGCAGGCCTTACACATGCAAGTCGAGCGGAGATGAGGGTGGCTTCGCACACTTGATCT
CTAGCGGCGGACGGGTGAGTAATGCTTAGGAATCTGCCTATTAGTGGGGGACAACATTCCGAAA
GGAATGCTAATACCGCATACGTCCTACGGGAGAAAGCAGGGGATCTTCGGACCTTGCGCTAAAT
GATGAGCCTAAGTCGGATTAGCTAGTTGGTGGGGTAAAGGCCTACCAAGGCGACGATCTGTAGC
GGGTCTGAGAGGATGATCCGCCACACTGGGACTGAGACACGGCCCAGACTCCTACGGGAGGCA
GCAGTGGGGAATATTGGACAATGGGCGGAAGCCTGATCCAGCCATGCCGCGTGTGTGAAGAAG
GCCTTTTGGTTGTAAAGCACTTTAAGCGAGGAGGAGGCTACCTAGATTAATACTCTGGGATAGTG
GACGTTACTCGCAGAATAAGCACCGGCTAACTCTGTGCCAGCAGCCGCGGTAATACAGAGGGTG
CGAGCGTTAATCGGATTTACTGGGCGTAAAGCGTGCGTAGGCGGCTGATTAAGTCGGATGTGAA
ATCCCTGAGCTTAACTTAGGAATTGCATTCGATACTGGTCAGCTAGAGTATGGGAGAGGATGGT
AGAATTCCAGGTGTAGCGGTGAAATGCGTAGAGATCTGGAGGAATACCGATGGCGAAGGCAGC
CATCTGGCCTAATACTGACGCTGAGGTACGAAAGCATGGGGAGCAAACAGGATTAGATACCCTG
GTAGTCCATGCCGTAAACGATGTCTACTAGCCGTTGGGGCCTTTGAGGCTTTAGTGGCGCAGCTA

ACGCGATAAGTAGACCGCCTGGGGAGTACGGTCGCAAGACTAAAACTCAAATGAATTGACGGG
GGCCCGCACAAGCGGTGGAGCATGTGGTTTAATTCGATGCAACGCGAAGAACCTTACCTGGCCT
TGACATACTAGAAACTTTCCAGAGATGGATTGGTGCCTTCGGGAATCTAGATACAGGTGCTGCAT
GGCTGTCGTCAGCTCGTGTCGTGAGATGTTGGGTTAAGTCCCGCAACGAGCGCAACCCTTTTCCT
TACTTGCCAGCATTTCGGATGGGAACTTTAAGGATACTGCCAGTGACAAACTGGAGGAAGGCGG
GGACGACGTCAAGTCATCATGGCCCTTACGGCCAGGGCTACACACGTGCTACAATGGTCGGTAC
AAAGGGTTGCTACCTAGCGATAGGATGCTAATCTCAAAAAGCCGATCGTAGTCCGGATTGGAGT
CTGCAACTCGACTCCATGAAGTCGGAATCGCTAGTAATCGCGGATCAGAATGCCGCGGTGAATA
CGTTCCCGGGCCTTGTACACACCGCCCGTCACACCATGGGAGTTTGTTGCACCAGAAGTAGGTAG
TCTAACCGCAAGGAGGACGCTACCCAAACCGGGGG

>T2_ *Enterobacter* spp._16S ribosomal RNA gene

GCCCGGGGCGGGCAGGGCCCTAACACATTGCAAGTCGAACGGTAACAGGAAGCAGCTTGCTGCT
TTGCTGACGAGTGGCGGACGGGTGAGTAATGTCTGGGAAACTGCCTGATGGAGGGGGATAACTA
CTGGAAACGGTAGCTAATACCGCATAACGTCGCAAGACCAAAGAGGGGGACCTTCGGGCCTCTT
GCCATCGGATGTGCCCAGATGGGATTAGCTAGTAGGTGGGGTAACGGCTCACCTAGGCGACGAT
CCCTAGCTGGTCTGAGAGGATGACCAGCCACACTGGAACTGAGACACGGTCCAGACTCCTACGG
GAGGCAGCAGTGGGGAATATTGCACAATGGGCGCAAGCCTGATGCAGCCATGCCGCGTGTATGA
AGAAGGCCTTCGGGTTGTAAAGTACTTTCAGCGGGGAGGAAGGCGATAAGGTTAATAACCTTGT
CGATTGACGTTACCCGCAGAAGAAGCACCGGCTAACTCCGTGCCAGCAGCCGCGGTAATACGGA
GGGTGCAAGCGTTAATCGGAATTACTGGGCGTAAAGCGCACGCAGGCGGTCTGTCAAGTCGGAT
GTGAAATCCCCGGGCTCAACCTGGGAACTGCATTCGAAACTGGCAGGCTAGAGTCTTGTAGAGG
GGGGTAGAATTCCAGGTGTAGCGGTGAAATGCGTAGAGATCTGGAGGAATACCGGTGGCGAAG
GCGGCCCCCTGGACAAAGACTGACGCTCAGGTGCGAAAGCGTGGGGAGCAAACAGGATTAGAT
ACCCTGGTAGTCCACGCCGTAAACGATGTCGACTTGGAGGTTGTGCCCTTGAGGCGTGGCTTCCG
GAGCTAACGCGTTAAGTCGACCGCCTGGGGAGTACGGCCGCAAGGTTAAAACTCAAATGAATTG
ACGGGGGCCCGCACAAGCGGTGGAGCATGTGGTTTAATTCGATGCAACGCGAAGAACCTTACCT
ACTCTTGACATCCAGAGAACTTAGCAGAGATGGTTTGGTGCCTTCGGGAACTCTGAGACAGGTG
CTGCATGGCTGTCGTCAGCTCGTGTTGTGAAATGTTGGGTTAAGTCCCGCAACGAGCGCAACCCT
TATCCTTTGTTGCCAGCGGTCAGGCCGGGAACTCAAAGGAGACTGCCAGTGATAAACTGGAGGA
AGGTGGGGATGACGTCAAGTCATCATGGCCCTTACGAGTAGGGCTACACACGTGCTACAATGGC
GCATACAAAGAGAAGCGACCTCGCGAGAGCAAGCGGACCTCATAAAGTGCGTCGTAGTCCGGA
TTGGAGTCTGCAACTCGACTCCATGAAGTCGGAATCGCTAGTAATCGTGGATCAGAATGCCACG
GTGAATACGTTCCCGGGCCTTGTACACACCGCCCGTCACACCATGGGAGTGGGTTGCAAAAGAA
GTAGGTAGCTTAACCTTCGGGAGGGCGCTTACCCCCACCTTTGGGATTCCT

>T8_ *Citrobacter* spp._16S ribosomal RNA gene

TCATTGCCGGCGGGCTACACATGCAGTCGAGCGGTAGCACAGAGAGCTTGCTCTCGGGTGACGA
GCGGCGGCCGGGTGAGTAATGTCTGGGAAACTGCCTGATGGAGGGGGATAACTACTGGAAACG
GTAGCTAATACCGCATAACGTCGCAAGACCAAAGAGGGGGACCTTCGGGCCTCTTGCCATCAGA
TGTGCCCAGATGGGATTAGCTAGTAGGTGGGGTAACGGCTCACCTAGGCGACGATCCCTAGCTG
GTCTGAGAGGATGACCAGCCACACTGGAACTGAGACACGGTCCAGACTCCTACGGGAGGCAGC
AGTGGGGAATATTGCACAATGGGCGCAAGCCTGATGCAGCCATGCCGCGTGTATGAAGAAGGCC

TTCGGGTTGTAAAGTACTTTCAGCGAGGAGGAAGGTGTTGAGGTTAATAACCTCAGCAATTGAC
GTTACTCGCAGAAGAAGCACCGGCTAACTCCGTGCCAGCAGCCGCGGTAATACGGAGGGTGCAA
GCGTTAATCGGAATTACTGGGCGTAAAGCGCACGCAGGCGGTCTGTCAAGTCGGATGTGAAATC
CCCGGGCTCAACCTGGGAACTGCATTCGAAACTGGCAGGCTAGAGTCTTGTAGAGGGGGGTAGA
ATTCCAGGTGTAGCGGTGAAATGCGTAGAGATCTGGAGGAATACCGGTGGCGAAGGCGGCCCCC
TGGACAAAGACTGACGCTCAGGTGCGAAAGCGTGGGGAGCAAACAGGATTAGATACCCTGGTA
GTCCACGCCGTAAACGATGTCGACTTGGAGGTTGTTCCCTTGAGGAGTGGCTTCCGGAGCTAACG
CGTTAAGTCGACCGCCTGGGGAGTACGGCCGCAAGGTTAAAACTCAAATGAATTGACGGGGGCC
CGCACAAGCGGTGGAGCATGTGGTTTAATTCGATGCAACGCGAAGAACCTTACCTACTCTTGAC
ATCCAGAGAACTTAGCAGAGATGCTTTGGTGCCTTCGGGAACTCTGAGACAGGTGCTGCATGGC
TGTCGTCAGCTCGTGTTGTGAAATGTTG
